# Air-Stable Cobalt−Semiquinone Radical Complexes on Carbon Nanotubes: A Redox Switch for Anion Response

**DOI:** 10.1021/jacs.5c16011

**Published:** 2025-12-04

**Authors:** Sabrina L. Kleynemeyer, Alex M. Wu, Daniel Klose, Yanlin Pan, Jan Reger, Christina Moser, Máté J. Bezdek

**Affiliations:** Department of Chemistry and Applied Biosciences, 27219 ETH Zürich, Vladimir-Prelog-Weg 1, Zürich 8093, Switzerland

## Abstract

Surface-bound radicals represent an emerging frontier for controlling the physical and chemical properties of nanomaterials in applications across sensing, electrocatalysis, spintronics, and redox-switchable devices. However, air- and moisture-stable radicals are rare, and harnessing their chemical reactivity for nanomaterial function remains underexplored. Herein, we report the synthesis and characterization of a dianionic cobalt complex featuring a ligand-centered semiquinone radical and outer-sphere pyrene trimethylammonium cations (**[Pyr]**_**2**_ **[Co]**), which can be noncovalently immobilized on single-walled carbon nanotubes (SWCNTs). The resulting hybrid material (**SWCNT-[Pyr]**_**2**_ **[Co]**) features molecularly defined surface functionalization, as established by Raman and X-ray photoelectron spectroscopic characterization in combination with electron microscopy. Electron paramagnetic resonance (EPR) spectroscopy revealed an interaction between the paramagnetic cobalt complex and the SWCNT surface, with radical character persisting in the presence of air and moisture for several months. Electrochemical studies showed that the ligand-centered radical in **[Pyr]**_**2**_ **[Co]** undergoes reversible oxidation at mild potentials, triggering selective chemical reactivity with exogenous cyanide ions (CN^−^). This feature, in combination with the environmental stability of the radical, was leveraged to demonstrate proof-of-concept electrochemical CN^−^ detection using **SWCNT-[Pyr]**_**2**_ **[Co]**. Overall, our findings establish a remarkably stable radical-nanotube interface and outline a general strategy for constructing hybrid materials with redox-switchable function.

## Graphical abstract

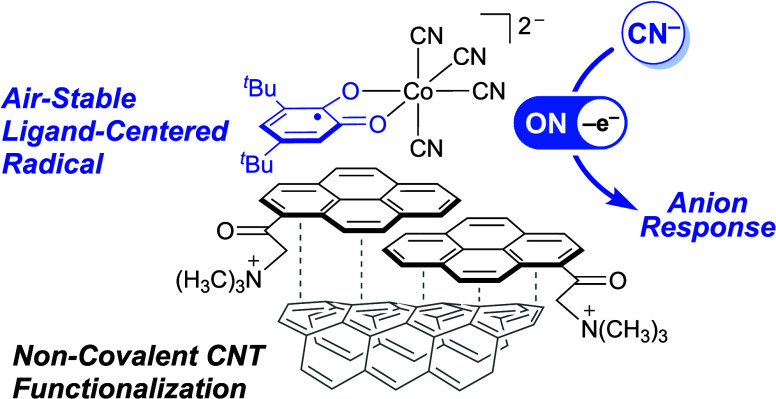

## Introduction

The controlled modification of carbon nanotubes (CNTs) with surface functionalities is crucial for their implementation as next-generation building blocks across sensing,^1–3^ bioimaging,^4–6^ energy storage,^7–9^ spintronics^10–12^ and catalysis.^13–16^ In these settings, the chemical and physical properties of CNTs can be matched to the desired application using surface functionalization approaches that include covalent bond formation,^17–19^ noncovalent interactions^20–22^ and mechanical nanotube interlocking.^23,24^ Given the broad potential utility of functional CNTs, new methods for the molecularly defined modification of their surface chemistry and electronic structure continue to attract significant research interest.^25–29^

An emerging direction in carbon nanotube functionalization involves the integration of surface groups bearing unpaired electrons. Shown in Scheme 1A, recent efforts have focused on decorating single- and multiwalled CNTs with open-shell organic molecules, transition metal complexes and lanthanides with the aim of controlling nanotube doping and spin-dependent phenomena such as magnetoresistance, spin-valve switching, Kondo effects, quantum spin transport and quantum coherence.^10,12,30–33^ While these studies highlight the effectiveness of spin-bearing molecules for tuning the electronic and magnetic properties of CNTs, they largely treat surface-bound radicals as chemically passive spin centers.^34,35^ Consequently, the potential to harness radical reactivity for functional CNT design is essentially unexplored.

An attractive application of surface-bound radicals is their use as redox switches^36–38^ for inducing reversible changes in CNT properties through electrochemical stimuli.^39,40^ Radicals are particularly well-suited for this role, as their singly occupied orbitals facilitate one-electron redox events that are often accessible at milder potentials relative to their closed-shell counterparts.^41,42^ Furthermore, initiating redox switching from a persistent radical avoids the formation of new high-energy radical intermediates upon electron transfer, which could otherwise lead to uncontrolled side reactions and compromise redox reversibility. Beyond serving as supports, conductive CNT networks provide an electronically coupled platform in which the redox activity of immobilized radicals can be directly transduced into measurable changes in charge transport.^33^ This electronic addressability renders CNT-bound radicals especially promising for sensing applications, where molecular-level redox events must be translated into electrical readouts.^3^ A key challenge, however, is the limited air- and moisture-stability of most radicals that restricts their broader utility.^43,44^ Developing CNT-bound radicals that combine environmental robustness with reversible electrochemical features is therefore essential for accessing redox-switchable radical nanomaterials with practical sensing function.

**Scheme 1:**
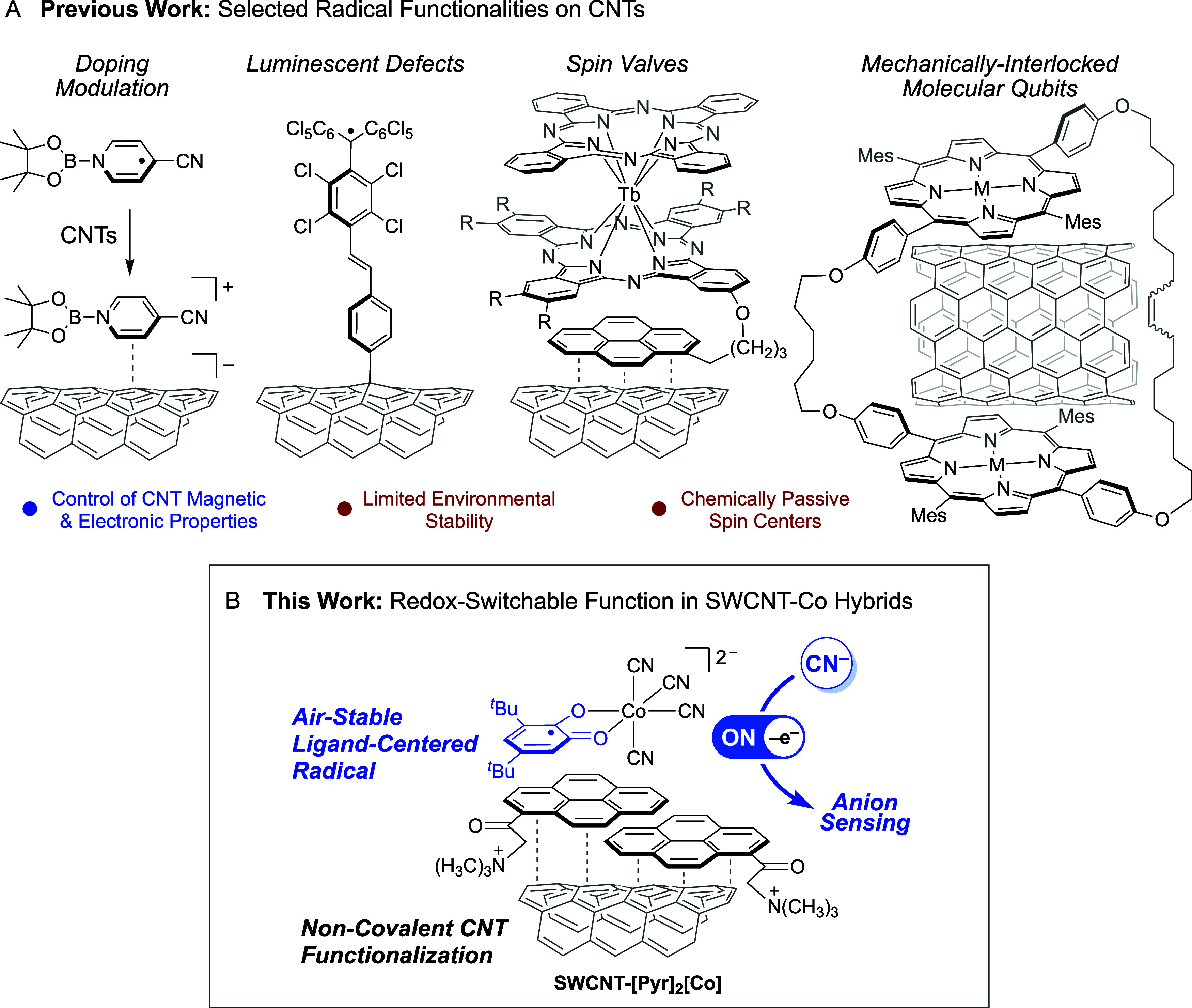
(A) Selected Examples of Carbon Nanotubes Featuring Radical Surface Functionalities; (B) The Strategy Reported in the Present Study^a^

a R = C_6_H_13_; CNT = carbon nanotube; SWCNT = single-walled CNT; Mes = 2,4,6-trimethylphenyl; M = Co or Cu.

In this work, we target the installation of air-stable complexes containing unpaired electrons on CNT surfaces to access a new class of environmentally stable, radical-bearing functional nanomaterials. Toward this goal, we were drawn to the use of metal complexes supported by redox-active ligands.^45,46^ Owing to the presence of low-lying π* orbitals relative to metal d-manifolds,^47,48^ we reasoned that ligand redox activity could be leveraged to facilitate electron delocalization and stabilize unpaired electrons. In addition, metal complexes supported by redox active ligands typically exhibit reversible electron transfer processes.^49–53^ Consequently, we aimed to utilize a ligand-based redox couple to “switch-on” chemical sensing via redox-triggered reactivity. While ligand redox activity has emerged as a powerful design element for mediating multielectron transformations in synthesis and catalysis,^54–60^ its use to control CNT function remains in its infancy.

Herein, we report that a dianionic cobalt complex supported by a redox-active *o*-semiquinone ligand^61^ can be noncovalently immobilized on single-walled carbon nanotubes (SWCNTs) to yield air-stable radical nanocomposites (**SWCNT-[Pyr]**_**2**_ **[Co]**, Scheme 1B). Immobilization is directed by outer-sphere trimethylammonium-functionalized pyrene cations that promote π−π stacking at the SWCNT surface. Electron paramagnetic resonance (EPR), X-ray photoemission spectroscopy (XPS) and electron microscopy are employed to probe the **SWCNT-[Pyr]**_**2**_ **[Co]** interface and confirm molecularly defined immobilization of the complex featuring a ligand-centered radical. Electrochemical studies show that **SWCNT-[Pyr]**_**2**_ **[Co]** exhibits reversible redox features at mild potentials, wherein single-electron oxidation triggers selective chemical reactivity with exogenous cyanide ions. Finally, we establish a quantitative correlation between the concentration of cyanide and the electrochemical response, thereby translating ligand redox activity to switchable anion sensing in a new class of radical nanomaterials.

## Results and Discussion

### Synthesis and Characterization of the Cobalt Complex [Pyr]_2_[Co]

Our studies began with the synthesis of an air- and moisture-stable complex bearing an unpaired electron, designed for immobilization on SWCNT networks. Toward this goal, we drew inspiration from previous work demonstrating that redox-active quinone ligands can support reduced metal centers and confer a degree of air stability.^62–64^ Specifically, we targeted a cobalt cyanide complex featuring a semiquinone ligand-based radical [(*n*-Bu)_4_N]_2_[(*t-*Bu-SQ)­Co­(CN)_4_] (**[(** ***n*** **-Bu)**_**4**_ **N]**_**2**_ **[Co]**; *t-*Bu-SQ = 3,5-(*t*-Bu)_2_-1,2-semiquinone) which was reported to exhibit air stability.^61^ Building on this precedent, we targeted the pairing of an outer-sphere pyrene-functionalized trimethylammonium cation with **[Co]** ^**2−**^. Our aim was to leverage π−π stacking of the pyrene counterion with SWCNT surfaces and combine this with electrostatic interactions for the successful immobilization of the anionic cobalt complex.^65,66^

Shown in Scheme 2A, the targeted cobalt complex was synthesized by salt metathesis between **[(** ***n*** **-Bu)**_**4**_ **N]**_**2**_ **[Co]** and pyrene-functionalized ammonium bromide **[Pyr]­[Br]** (**Pyr** = trimethyl-2-oxo-2­(pyren-1-yl)­ethyl-1-ammonium) in aqueous solution.^67^ After stirring at room temperature for 20 min, the desired complex **[Pyr]**_**2**_ **[Co]** precipitated as an analytically pure dark red solid in 87% yield. While the cobalt anion **[Co]** ^**2−**^ is NMR-silent due to its paramagnetic nature, peaks observed at δ = 8.92, 8.46−8.21, 5.52, and 3.47 ppm in its ^1^H NMR spectrum (DMSO-*d*_6_) were assigned to the outer sphere **[Pyr]** ^**+**^ cations (Figure S81). The presence of cyanide ligands in **[Pyr]**_**2**_ **[Co]** was confirmed by infrared (IR) spectroscopy which showed a diagnostic C≡N vibration at 2121 cm^−1^ (Figure S100). Single crystals of **[Pyr]**_**2**_ **[Co]** suitable for X-ray diffraction established that the coordination environment at cobalt remained unchanged during the anion exchange and showed the presence of two outer-sphere **[Pyr]** ^**+**^ cations per cobalt center in **[Pyr]**_**2**_ **[Co]** (Scheme 2B). In the solid-state, the **[Co]** ^**2−**^ anions and **[Pyr]** ^**+**^ cations pack into alternating layers, with π−π stacking between adjacent pyrene units (centroid−centroid distances ∼ 3.55 Å) forming slipped stacks that propagate along one crystallographic axis (Scheme 2C). The *t*-Bu-SQ ligand in **[Co]** ^**2−**^ shows elongated C−O bonds (C−O_avg_ = 1.295(4) Å), consistent with previous reports of Co complexes bearing ligand-based semiquinone radicals.^61,68^

**Scheme 2:**
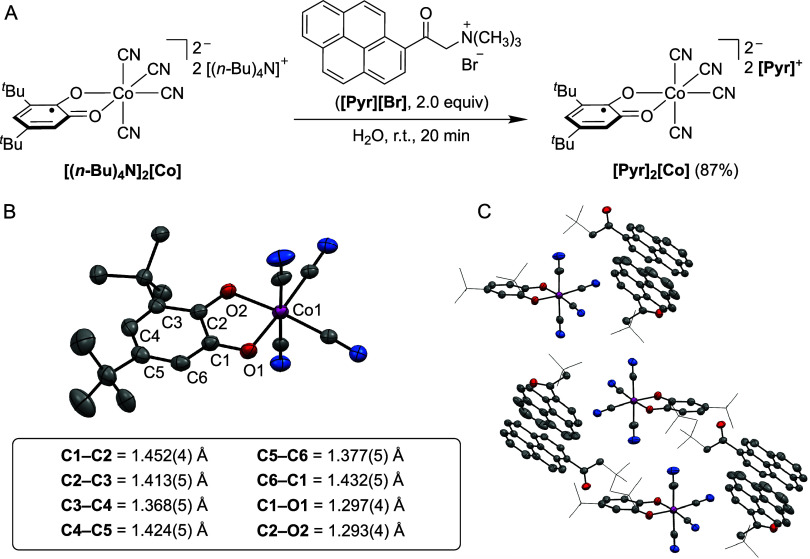
(A) Synthesis, (B) Solid-State Structure^a^,[Fn sch2-fn2] and (C) Solid-State Packing^a^,[Fn sch2-fn3] of [Pyr]_2_[Co].

a Represented with 50% probability ellipsoids. Hydrogen atoms and solvent molecules present in the crystallographic unit cell are omitted for clarity.
b
**[Pyr]** ^**+**^ counterions are omitted.
c
*t*-Bu and N­(CH_3_)_3_ substituents are represented without probability ellipsoids.

Magnetic and spectroscopic measurements were carried out to gain deeper insight into the electronic structure of **[Pyr]**_**2**_ **[Co]**. A solution-state magnetic moment of 1.95 ± 0.09 μ_*B*_ (Evans method, DMSO-*d*_6_) was measured for the complex at room temperature, consistent with the presence of a single unpaired electron. The X-band continuous-wave (CW) EPR spectrum of **[Pyr]**_**2**_ **[Co]** was recorded in PhMe/MeOH (3:1, v/v) solution at room temperature and features an isotropic signal (*g*_iso_ = 2.0024) with hyperfine coupling to cobalt [*A*_iso_(^59^Co) = 22.53 MHz, *I* = ^7^/_2_] and to an aromatic proton at the 4-position of the *t-*Bu-SQ ligand [*A*_iso_(^1^H) = 8.17 MHz, *I* = ^1^/_2_)] (Figure 1A). The proximity of the *g*-value to that of a free-electron (*g*_e_ = 2.0023)^69^ as well as the small hyperfine coupling to cobalt suggest that the singly occupied molecular orbital (SOMO) of **[Pyr]**_**2**_ **[Co]** is principally ligand-based. This view was further supported by density functional theory (DFT) computations (B3LYP/def2-TZVP), which show that spin-density is mainly delocalized across the *t-*Bu-SQ ligand with a minor contribution from the cobalt center (Figure 1B). The ligand-centered radical in **[Pyr]**_**2**_ **[Co]** also influences its optical properties. Complex **[Pyr]**_**2**_ **[Co]** is dark purple in DMF solution and exhibits a low energy absorption in its UV−vis spectrum at λ_max_ = 530 nm (ϵ = 1.08 × 10^5^ M^−1^ cm^−1^) in addition to features at λ_max_ = 288, 367, and 396 nm that are assignable to the **[Pyr]** ^+^ counterions (Figure 1C). The energy of the transition at λ_max_ = 530 nm depends strongly on the solvent medium, wherein a bathochromic shift was generally observed with decreasing solvent polarity (Figure S48).^70–72^ The parentage of this feature was assigned by time-dependent DFT computations (ωB97X-D3/def2-TZVP) to correspond to a *t-*Bu-SQ-based π → π* transition in **[Pyr]**_**2**_ **[Co]** (SOMO−1 → SOMO, Figure 1D). Overall, the structural, spectroscopic and computational data suggest that **[Pyr]**_**2**_ **[Co]** is likely best described as a low-spin Co­(III) complex bearing a *t-*Bu-SQ ligand-based “semiquinone” radical anion.^61,73^

**Figure 1:**
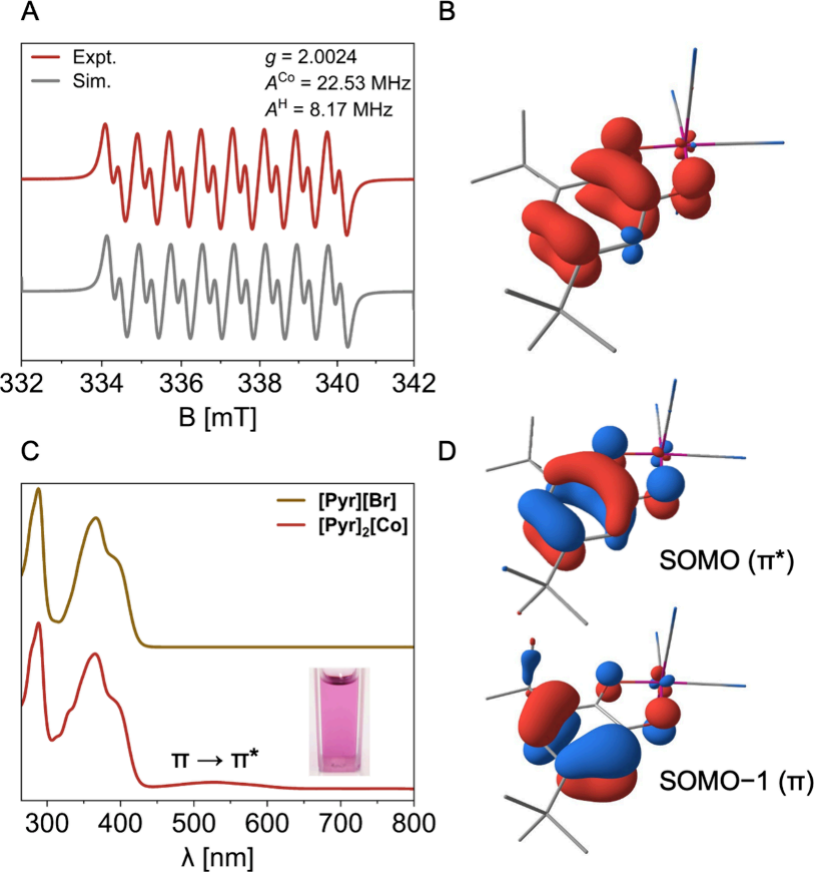
(A) X-Band CW EPR spectrum of **[Pyr]_2_[Co]** in PhMe/MeOH (3:1, v/v) at 298 K. (B) DFT-computed spin density isosurface using contour levels of ± 0.4%. (C) UV−vis spectra of **[Pyr]­[Br]** and **[Pyr]_2_[Co]** in DMF (50 μM) together with a sample photograph (inset). (D) DFT-computed frontier orbital illustrations (SOMO and SOMO−1) of **[Co]^2−^**, relevant to the low-energy transition (λ_max_ = 530 nm) observed in the UV−vis spectrum of **[Pyr]_2_[Co]**. See SI for computational details.

Having established the presence of a ligand-based radical in **[Pyr]**_**2**_ **[Co]**, we next evaluated the long-term stability of the complex, anticipating that its use in applied settings will require air and moisture tolerance. Although **[(** ***n*** **-Bu)**_**4**_ **N]**_**2**_ **[Co]** was reported to be air tolerant, the duration of this stability was not quantified.^61^ We found that solutions of **[Pyr]**_**2**_ **[Co]** degrade slowly, both in air and under inert conditions (*t*_1/2_ ≈ 24 h), as evidenced by the loss of its characteristic features by EPR and UV−vis spectroscopy (Figures S45−S46). Analysis of the resulting solution by ^1^H NMR spectroscopy (MeCN-*d*_3_) showed peaks assignable to free *t*-Bu-BQ ligand (*t-*Bu-BQ = 3,5-(*t*-Bu)_2_-1,2-benzoquinone), as well as diamagnetic **[Pyr]** ^**+**^ (Figure S47). In contrast, samples of **[Pyr]**_**2**_ **[Co]** containing excess *t-*Bu-BQ exhibited significantly higher stability (*t*_1/2_ ≈ 1 week). Similarly, **[Pyr]**_**2**_ **[Co]** showed markedly improved stability when protected from light, with no measurable impact from the presence of air and moisture (Figure S45). These findings indicate that the primary degradation pathway for **[Pyr]**_**2**_ **[Co]** in solution involves light-induced *t-*Bu-BQ dissociation, consistent with the presence of a ligand-based π → π* transition in the visible light region of its UV−vis spectrum (Figure 1D). While **[Pyr]**_**2**_ **[Co]** is relatively stable in solution when kept in the dark, it was found to be stable to air, moisture and ambient light in the solid-state and can be stored on the benchtop for >1 month. This remarkable environmental robustness renders **[Pyr]**_**2**_ **[Co]** well-suited for solid-state responsive materials applications.

### SWCNT Functionalization and Characterization of SWCNT-[Pyr]_2_[Co]

With the air-stable radical complex **[Pyr]**_**2**_ **[Co]** in hand, we proceeded with its noncovalent immobilization on SWCNTs (P3-SWCNT; purified by nitric acid treatment, sonication in *o*-DCB and multiple MeOH washings. For purification details, see SI). SWCNTs were selected as the functionalization platform owing to their high surface area, efficient charge- and spin-transport characteristics, and established sensitivity to surface-bound molecular species, properties that make them particularly attractive for sensing and electronic applications.^3,28^ In a typical procedure, a 1:1 (*w*/*w*) mixture of **[Pyr]**_**2**_ **[Co]** and SWCNTs was bath-sonicated in 1,2-dichlorobenzene (*o*-DCB) at room temperature for 1 h (Figure 2A) and thoroughly washed with MeOH. A consistent degree of noncovalent functionalization was achieved in the resulting **SWCNT-[Pyr]**_**2**_ **[Co]** material as judged by a suite of characterization methods. X-ray photoelectron spectroscopy (XPS) was performed to confirm the elemental surface composition of **SWCNT-[Pyr]**_**2**_ **[Co]**. High resolution XPS scans showed Co 2p_1/2_ and Co 2p_3/2_ binding energies at 796.8 and 781.8 eV, respectively, assignable to the presence of a Co­(III) complex on the SWCNT surface (Figure 2B, left).^74^ Additionally, N 1s peaks were observed that are characteristic for cyanide ligands (398 eV)^75^ and *N*-alkyl ammonium ions with a smaller area (403 eV),^76,77^ as expected from the elemental composition of **[Pyr]**_**2**_ **[Co]** (Figure 2B, right). Thermal gravimetric analysis (TGA) of **SWCNT-[Pyr]**_**2**_ **[Co]** revealed a mass loss of 27.7% upon heating to 800 °C (Figure S33). This is significantly greater than the 19.0% mass loss observed for unfunctionalized SWCNTs for the same temperature range and supports the successful introduction of surface functionalities in **SWCNT-[Pyr]**_**2**_ **[Co].**

**Figure 2:**
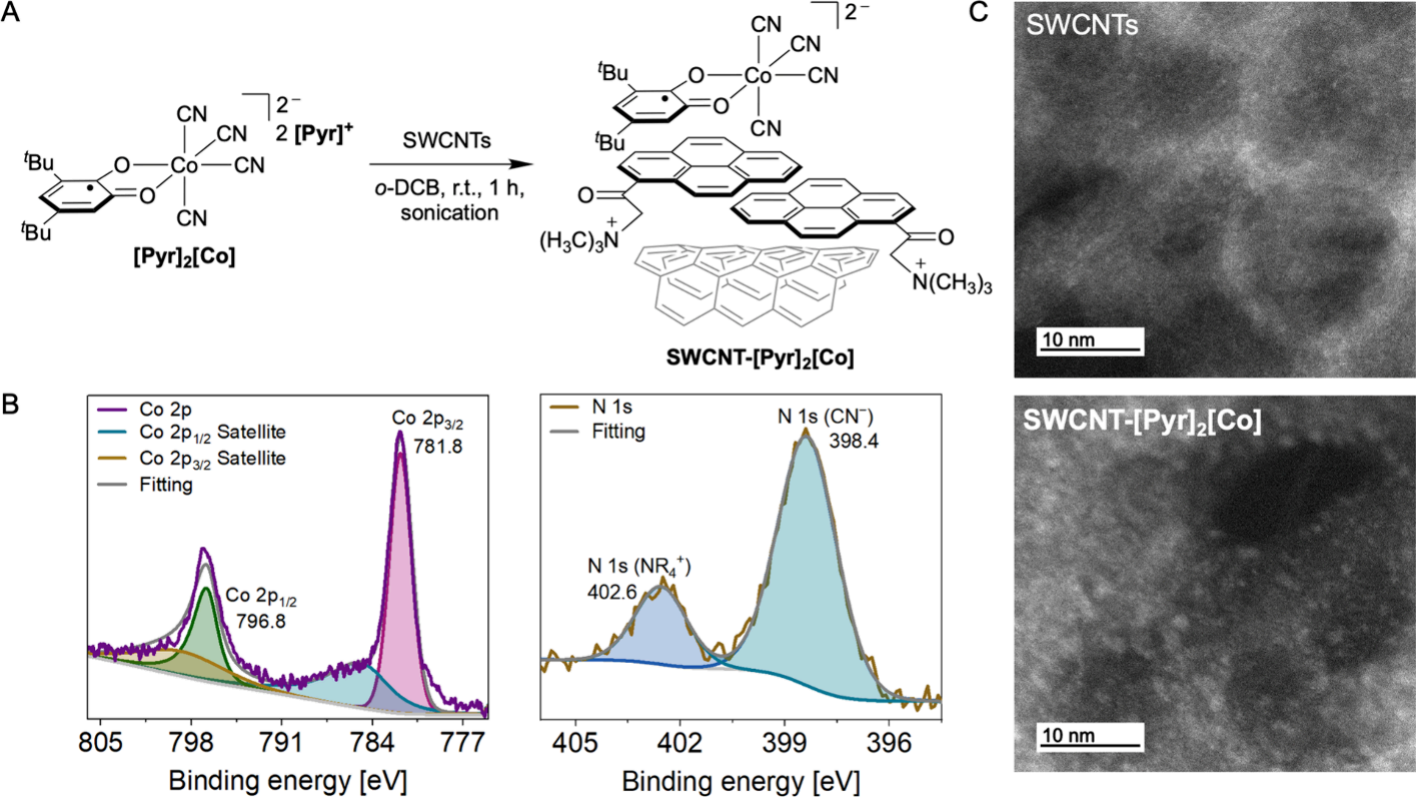
(A) Synthesis of **SWCNT-[Pyr]_2_[Co]**. (B) High-resolution Co 2p (left) and N 1s (right) XPS spectra of **SWCNT-[Pyr]_2_[Co]**. (C) High-angle annular dark field (HAADF) scanning transmission electron microscopy (STEM) micrographs of purified SWCNTs (top) and **SWCNT-[Pyr]_2_[Co]** (bottom).

To further support the presence of **[Pyr]**_**2**_ **[Co]** on the nanotubes and to evaluate the uniformity of the surface coverage, scanning transmission electron microscopy (STEM) data were collected. A control sample of SWCNTs subjected to identical processing, but without exposure to **[Pyr]**_**2**_ **[Co]** was also investigated. High-angle annular dark field (HAADF) STEM micrographs of **SWCNT-[Pyr]**_**2**_ **[Co]** revealed regularly spaced high-contrast regions that are absent in the control SWCNT sample (Figure 2C). Energy dispersive X-ray spectrometry (EDS) performed on these regions showed pronounced cobalt signals, consistent with the presence of **[Pyr]**_**2**_ **[Co]** (Figure S40). To determine whether the incorporation of complex **[Pyr]**_**2**_ **[Co]** disrupts the SWCNTs π-system via covalent sidewall modification, **SWCNT-[Pyr]**_**2**_ **[Co]** was analyzed by Raman spectroscopy (633 nm excitation wavelength, Figure S30). Particular attention was given to the D/G band area ratio, wherein an increased relative D-band intensity (1328 cm^−1^) is a diagnostic indicator of sidewall defects that give rise to symmetry-breaking Raman scattering.^78,79^ Importantly, the D/G band area ratio remained constant in **SWCNT-[Pyr]**_**2**_ **[Co]** relative to the SWCNT control, pointing to a noncovalent interaction between **[Pyr]**_**2**_ **[Co]** and the nanotube sidewalls. Taken together, these data confirm the uniform noncovalent immobilization of complex **[Pyr]**_**2**_ **[Co]** on the SWCNT surface without disruption of the nanotube π-system.

The immobilization of **[Pyr]**_**2**_ **[Co]** on SWCNTs proved highly robust toward both mechanical agitation and repeated washing. Control experiments showed that **SWCNT-[Pyr]**_**2**_ **[Co]** retained identical spectroscopic features after extended sonication and multiple washing cycles (see Supporting Information). These observations indicate that the noncovalent association between **[Pyr]**_**2**_ **[Co]** and the SWCNT surface withstands the purification procedures required for hybrid material preparation and remains intact during subsequent handling. To assess the role of the pyrene moiety, additional experiments were conducted employing cetyltrimethylammonium (**[CTA]** ^**+**^) cations in place of pyrene, where immobilization relies primarily on dispersion forces and weak electrostatic adsorption to the nanotube surface. The resulting **SWCNT-[CTA]**_**2**_ **[Co]** material displayed significantly lower cobalt surface loading, with pronounced desorption of the complex observed after washing. These results confirm that the pyrene-functionalized cation is essential for establishing the strong noncovalent interactions that yield durable and uniform cobalt coverage on the nanotube interface in **SWCNT-[Pyr]**_**2**_ **[Co].**

The presence of spin-bearing complexes on the surface of **SWCNT-[Pyr]**_**2**_ **[Co]** provided a unique opportunity to probe the nanomaterial electronic structure using electron paramagnetic resonance (EPR) spectroscopy. The X-band CW EPR spectrum of purified SWCNTs revealed a broad resonance spanning ∼500 mT, together with a sharper feature at *g* = 2.0037 (Figure 3A).^80,81^ Upon functionalization with **[Pyr]**_**2**_ **[Co]**, a distinct signal emerged at *g* = 2.001(6), consistent with the immobilization of a paramagnetic complex on the nanotube surface in **SWCNT-[Pyr]**_**2**_ **[Co]** (Figure 3A,B). To deconvolute the individual spectral contributions of each component in the hybrid material, echo-detected field sweep (EDFS) EPR spectra were acquired at 5 K. Reference samples were prepared consisting of pure SWCNTs, **[Pyr]**_**2**_ **[Co]** both in the solid state and in PhMe/MeOH glass (3:1, v/v) as well as spin-diluted^82^ solid **[Pyr]**_**2**_ **[Co]** (Figure 3C). Comparing the spectrum of **SWCNT-[Pyr]**_**2**_ **[Co]** to the reference samples revealed that its broad, anisotropic line shape with rhombic symmetry is best described as the superimposition of signals from the nanotube matrix^80,81^ as well as the spin-diluted cobalt complex. Numerical analysis using a linear combination of these components reproduced the experimental trace and indicated a composition of ∼77% spin-diluted **[Pyr]**_**2**_ **[Co]** and ∼23% nanotube-only contributions (Figure 3D). The similarity of the cobalt component in the hybrid material’s trace with the spin-diluted **[Pyr]**_**2**_ **[Co]** solid indicates a homogeneous surface distribution of **[Pyr]**_**2**_ **[Co]** on the nanotubes, with minimal interaction between individual complexes. In line with the obtained TEM images, this supports minimal aggregation of the cobalt complex in **SWCNT-[Pyr]**_**2**_ **[Co].**

**Figure 3:**
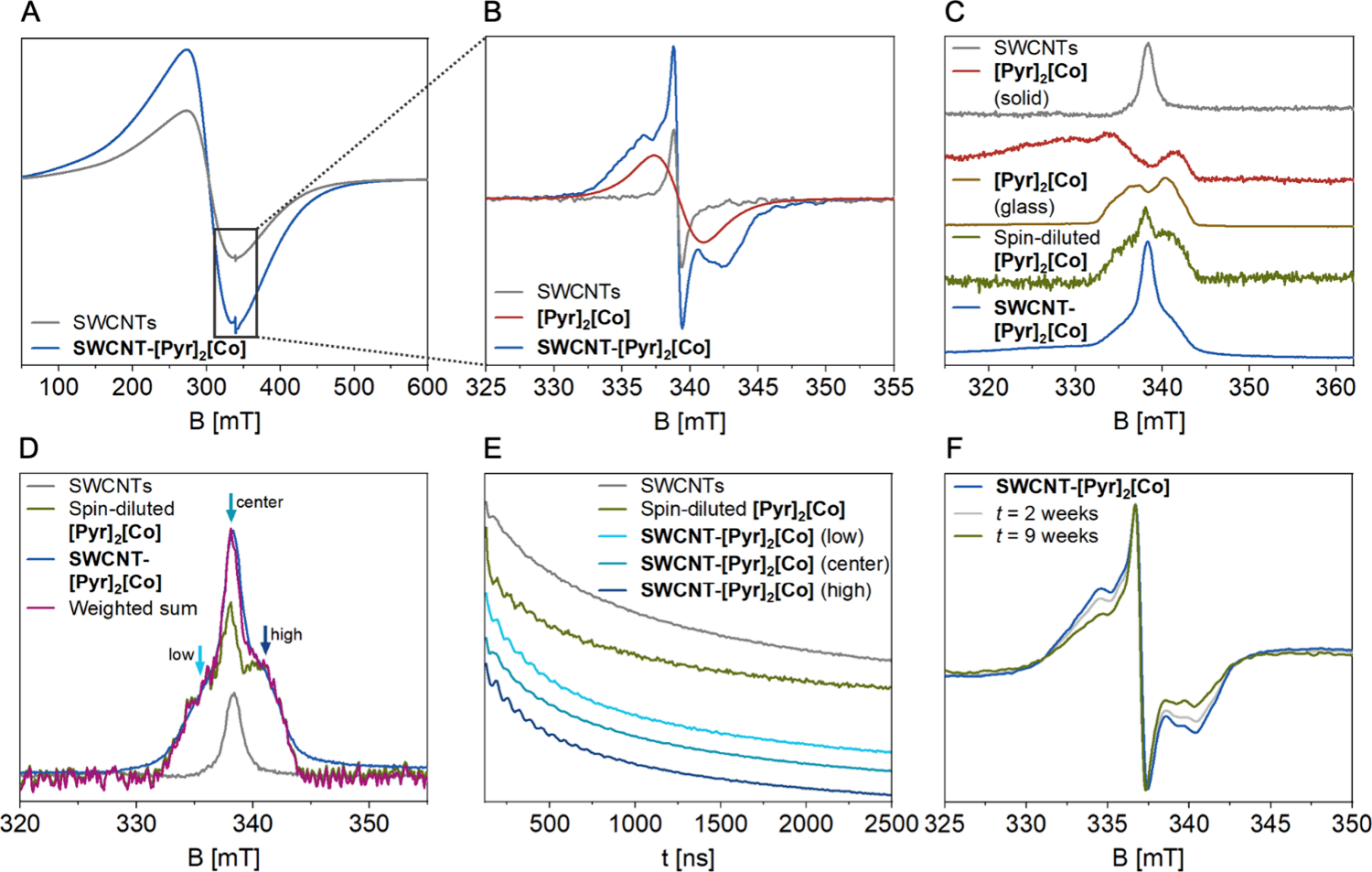
(A) X-band CW EPR spectra for SWCNTs (gray) and **SWCNT-[Pyr]_2_[Co]** (blue) collected at 298 K. (B) Zoomed-in background-corrected EPR spectra from (A) for SWCNTs (gray), and **SWCNT-[Pyr]_2_[Co]** (blue) compared to solid **[Pyr]_2_[Co]** (red). (C) X-band echo-detected field sweep (EDFS) EPR spectra of SWCNTs (gray), solid (red) and glassy (bronze) **[Pyr]_2_[Co]**, spin-diluted **[Pyr]_2_[Co]** (green), and **SWCNT-[Pyr]_2_[Co]** (blue), collected at 5 K. (D) Normalized X-band EDFS EPR difference spectra of SWCNTs (gray), spin-diluted **[Pyr]_2_[Co]** (green), **SWCNT-[Pyr]_2_[Co]** with SWCNT component subtracted (blue), and the calculated weighted-sum spectrum of all components (magenta), collected at 5 K. (E) Three-pulse electron spin echo envelope modulation (3pESEEM) time traces of SWCNTs (gray), spin-diluted **[Pyr]_2_[Co]** (green) and **SWCNT-[Pyr]_2_[Co]** at low (light-blue), center (petrol) and high (blue), magnetic field positions, collected at τ = 144 ns and 5 K. (F) X-band CW EPR spectra for a sample of **SWCNT-[Pyr]_2_[Co]** after 0 d (blue), 14 d (light-gray) and 63 d (petrol) stored under air and exposed to ambient light, collected at 298 K. For further experimental and data fitting details, see SI.

To gain deeper insight into the nature of the electronic interaction between the cobalt complex and the SWCNT support, pulsed EPR experiments were carried out. Inversion recovery experiments showed that the longitudinal electron spin relaxation time (T_1_) is shorter in **SWCNT-[Pyr]**_**2**_ **[Co]** than in pure SWCNTs, and markedly shorter than in spin-diluted **[Pyr]**_**2**_ **[Co]**. This behavior indicates stronger magnetic coupling to the surrounding molecular environment, consistent with interactions involving the unpaired electron of the cobalt complex and delocalized π-electrons of the nanotube interface (Figure S11). Two- and three-pulse electron spin echo envelope modulation (2p/3pESEEM) experiments supported this assignment. At low and high B-field positions, nuclear modulation patterns of **SWCNT-[Pyr]**_**2**_ **[Co]** matched those of the spin-diluted **[Pyr]**_**2**_ **[Co]** reference sample (Figure 3E). Concomitantly, the trace at the central magnetic field position shows minor nuclear modulation, arising from specific electron−nuclear hyperfine couplings, consistent with its assignment as the SWCNT spectral component (Figures S12 and S14). These features are most clearly distinguished when comparing the 3pESEEM traces of the samples at a pulse delay time of τ = 144 ns, indicating an interaction between **[Pyr]**_**2**_ **[Co]** and the SWCNT electronic matrix (Figure 3E).^83^ Lastly, Rabi oscillations measured by pulse EPR at 5 K confirmed an *S* = 1/2 spin state for all samples, with differences in damping behavior consistent with the spectral decomposition and the respective contributions of the SWCNT and cobalt complex. Altogether, these results establish that the ligand-centered radical in **[Pyr]**_**2**_ **[Co]** remains EPR-active upon immobilization, retaining its spin state and *g*-tensor, and is interacting with the SWCNT matrix magnetically and electronically.

Besides providing electronic structure insights, EPR experiments were utilized to establish the long-term environmental stability of **SWCNT-[Pyr]**_**2**_ **[Co]**. Accordingly, the EPR signal of the functionalized nanomaterial was monitored over an extended time period under ambient conditions. Shown in Figure 3F, prominent features attributable to the cobalt complex remained visible even after exposure of **SWCNT-[Pyr]**_**2**_ **[Co]** to air, moisture and light for 9 weeks. The remarkable long-term persistence of EPR activity highlights the environmental stability of the radical nanomaterial and underscores its potential for deployment in practical settings.

### Redox Switchable Anion Sensing

With insights in hand concerning the electronic structure and environmental robustness of **SWCNT-[Pyr]**_**2**_ **[Co]**, we next examined whether its unpaired electrons could be leveraged for redox-switchable nanomaterial function. Specifically, we aimed to trigger chemical reactivity in **SWCNT-[Pyr]**_**2**_ **[Co]** by single-electron transfer and translate this process into a sensing response. As a soluble molecular reference, the electrochemical behavior of **[(** ***n*** **-Bu)**_**4**_ **N]**_**2**_ **[Co]** was first examined. Shown in Figure 4, the cyclic voltammogram (CV) of **[(** ***n*** **-Bu)**_**4**_ **N]**_**2**_ **[Co]** in 1,2-difluorobenzene (1,2-DFB) shows a reversible anodic and a quasi-reversible cathodic wave with half-wave potentials (*E*_1/2_) of − 0.04 V and − 1.3 V vs Fc/Fc^+^, respectively (Fc = ferrocene). Square-wave voltammetry (SWV) confirmed that these correspond to single-electron processes, generating **[Co]** ^**−**^ upon oxidation and **[Co]** ^**3−**^ upon reduction (Figure S22). The associated peak currents scale with the square root of scan rate, consistent with diffusion-controlled electron transfer in solution (Figure S21).

In comparison, the CV of a **SWCNT-[Pyr]**_**2**_ **[Co]** thin film exhibits analogous redox features, shifted to *E*_1/2_ = 0.169 V and − 1.05 V (Figure 4). Here, the peak currents vary linearly with scan rate, indicating surface-confined processes (Figure S27).^84^ Thus, the potential shift and the change from square-root to linear scan-rate dependence clearly distinguish the diffusion-controlled behavior of the molecular **[(** ***n*** **-Bu)**_**4**_ **N]**_**2**_ **[Co]** complex from thin-layer electrochemistry in the immobilized **SWCNT-[Pyr]**_**2**_ **[Co]** nanomaterial. The anodic **[Co]** ^**−**^/**[Co]** ^**2−**^ redox couple occurs at accessible potentials in both systems and remains fully reversible under extended cycling. By contrast, the cathodic **[Co]** ^**2−**^/**[Co]** ^**3−**^ couple becomes quasi-reversible, consistent with its significantly smaller SWV peak area relative to the **[Co]** ^**−**^/**[Co]** ^**2−**^ couple both in solution and in the solid-state (Figures S22 and S27). This attenuation can be attributed in part to the reduced stability of **[Co]** ^**3−**^, underscoring the anodic **[Co]** ^**−**^/**[Co]** ^**2−**^ couple as the more robust handle for electrochemical sensing applications.

**Figure 4:**
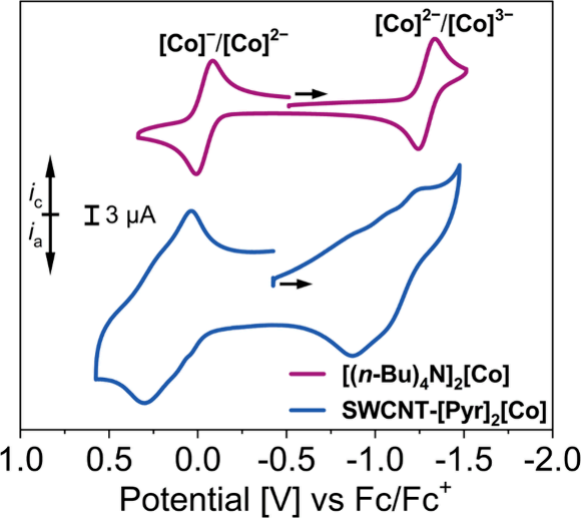
Cyclic voltammograms of **[(*n*-Bu)_4_N]_2_[Co]** (1.0 mM), and **SWCNT-[Pyr]_2_[Co]** (thin film drop-cast on working electrode). (0.10 M [(*n*-Bu)_4_N]­[PF_6_] supporting electrolyte in 1,2-DFB, 100 mV s^−1^ scan rate, r.t., glassy carbon working electrode). *i*_c_ *:* cathodic current, *i*_a_: anodic current.

The well-resolved and reversible **[Co]** ^**−**^/**[Co]** ^**2−**^ redox couple prompted us to explore whether changes in chemical environment would elicit an electrochemical response detectable by CV. Cyanide (CN^−^) was chosen as a test analyte because of its strong nucleophilicity and ubiquity as a ligand in coordination chemistry, making it a useful probe for redox-triggered reactivity. We hypothesized that oxidation of **[Co]** ^**2−**^ to the more electron-deficient **[Co]** ^**−**^ species would enhance its reactivity toward cyanide and alter its electrochemical profile in the presence of CN^−^. Indeed, upon collecting the CV of **[(** ***n*** **-Bu)**_**4**_ **N]**_**2**_ **[Co]** with increasing amounts of [(*n*-Bu)_4_N]­[CN] (0.1 to 0.9 mM), the **[Co]** ^**−**^/**[Co]** ^**2−**^ couple became irreversible with concomitant shifting of the peak anodic potential between *E*_pc_ = 0.0 and − 0.1 V (Figure 5A). These observations are consistent with an “EC” mechanism^85–87^ wherein fast electron transfer (E) first generates **[Co]** ^**−**^ *in situ* which undergoes a rate-limiting chemical reaction (C) with CN^−^. The CV of **SWCNT-[Pyr]**_**2**_ **[Co]** exhibits analogous changes upon titration with [(*n*-Bu)_4_N]­[CN], including increasing irreversibility and cathodic peak shifts of the **[Co]** ^**−**^/**[Co]** ^**2−**^ couple (Figure S57). Given the extended environmental stability of the surface-bound cobalt complex in **SWCNT-[Pyr]**_**2**_ **[Co]** (*vide supra*), these observations suggested that the functionalized nanomaterial could serve as a platform for electrochemical CN^−^ sensing.

**Figure 5:**
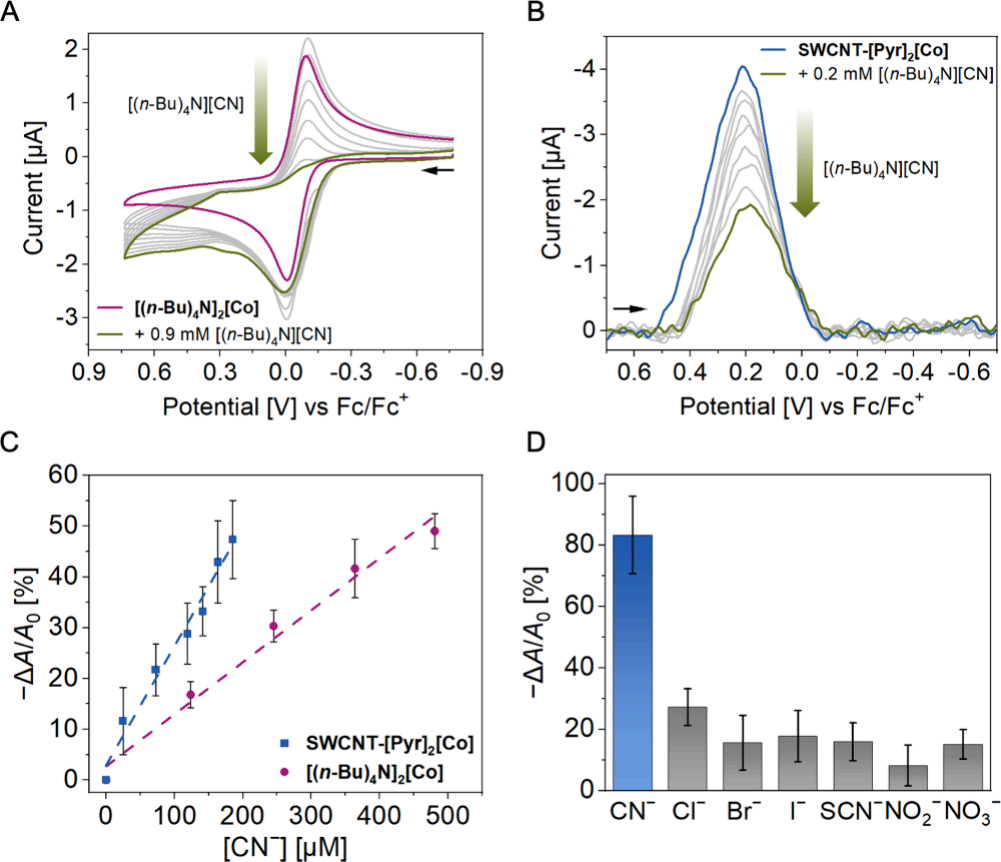
(A) Cyclic voltammograms of **[(*n*-Bu)_4_N]_2_[Co]** (1.0 mM in 1,2-DFB) upon titration with [(*n*-Bu)_4_N]­[CN] (12.5 mM in 1,2-DFB). The initial CV is shown in magenta, intermediate CVs in gray, and the final CV in green (final concentration: 0.9 mM [(*n*-Bu)_4_N]­[CN]). (0.10 M [(*n*-Bu)_4_N]­[PF_6_] supporting electrolyte in 1,2-DFB, 100 mV s^−1^ scan rate, r.t., glassy carbon working electrode). (B) Square wave voltammograms of **SWCNT-[Pyr]_2_[Co]** film upon titration with [(*n*-Bu)_4_N]­[CN] (2.5 mM in 1,2-DFB). The initial SWV is shown in blue, intermediate SWVs in gray, and the final SWV in green (final concentration: 0.2 mM [(*n*-Bu)_4_N]­[CN]). (0.10 M [(*n*-Bu)_4_N]­[PF_6_] supporting electrolyte in 1,2-DFB, 100 mV s^−1^ scan rate, r.t, glassy carbon working electrode). (C) Electrochemical sensing responses of **[(*n*-Bu)_4_N]_2_[Co]** (magenta) and **SWCNT-[Pyr]_2_[Co]** (blue) to varying concentrations of [(*n*-Bu)_4_N]­[CN]. (D) Electrochemical sensing response of **SWCNT-[Pyr]_2_[Co]** to various anions. Anions were added as their respective [(*n*-Bu)_4_N]^+^ salts with a final concentration of 1.85 mM. Error bars represent standard errors (*N* = 2).

To translate the observed CV changes for proof-of-concept cyanide detection, we turned to square wave voltammetry (SWV). This technique is especially well suited for obtaining analytically useful electrochemical responses as it offers enhanced sensitivity and improved discrimination of faradaic signals from the capacitive background currents of carbon nanomaterials.^88,89^ Similar to the CV measurements, the square wave voltammogram of **SWCNT-[Pyr]**_**2**_ **[Co]** in the presence of CN^−^ (25 to 185 μM) showed an inhibition of the cathodic component of the **[Co]** ^**−**^/**[Co]** ^**2−**^ redox couple, giving a decrease in the net peak current (Figure 5B). Accordingly, we defined the CN^−^ sensing signal as the percent inhibition of the SWV peak area [−Δ*A*/*A*_0_ (%) = (*A*_0_−*A*)/*A*_0_ × 100%; *A*_0_ = uninhibited SWV peak area], following established practices for inhibition-based electrochemical sensing, where analyte-induced suppression of the redox signal constitutes the analytical response.^90^ This approach accounts for the partial suppression of the **[Co]** ^**−**^/**[Co]** ^**2−**^ redox process, where only the cathodic component is lost due to **[Co]** ^**−**^ reactivity with CN^−^. Exposure of **SWCNT-[Pyr]**_**2**_ **[Co]** to varying CN^−^ concentrations yielded a linear change in SWV peak area inhibition in the range of 25 μM to 1.5 mM wherein the theoretical limit of detection (LOD) was calculated to be 896 nM CN^−^ (Figure 5C).^91^ Control experiments confirmed that the observed signal inhibition arises from the presence of cyanide rather than electrode degradation upon repeated scanning (Figures S67−S69). Conducting the SWV experiments with the soluble complex **[(** ***n*** **-Bu)**_**4**_ **N]**_**2**_ **[Co]** in 1,2-DFB also yielded a linear dependence of the SWV peak area inhibition on CN^−^ concentration, albeit with a higher LOD of 4.30 μM CN^−^ (Figure 5C). This comparison highlights the unique advantage of the SWCNT platform, which not only stabilizes the radical complex but also amplifies the sensing response through efficient electronic coupling (*vide supra*) and charge transport within the nanotube network.^3,28^ The ability of the SWCNT matrix to transduce^92^ local molecular redox events into macroscopic electrical signals underlies the significantly lower detection limit observed for **SWCNT-[Pyr]**_**2**_ **[Co].**

In addition to sensitivity, **SWCNT-[Pyr]**_**2**_ **[Co]** displayed pronounced selectivity for CN^−^ over a range of potentially interfering anions, including halides, SCN^−^, NO_2_^−^, and NO_3_^−^ (Figure 5D). The soluble complex **[(** ***n*** **-Bu)**_**4**_ **N]**_**2**_ **[Co]** in 1,2-DFB solution yielded a strikingly similar anion selectivity profile (Figure S62). Further, the **SWCNT-[Pyr]**_**2**_ **[Co]** film remained stable in 1,2-DFB, with no evidence of **[Pyr]**_**2**_ **[Co]** desorption or leaching upon solvent contact (Figure S67). These data support the notion that **[Co]** ^**2−**^ acts as a selector for CN^−^ on the nanotube surface upon oxidation and is responsible for the observed sensing response in the **SWCNT-[Pyr]**_**2**_ **[Co]** film.

#### Proposed Cyanide Sensing Mechanism

Our CV scan rate studies indicated that cyanide sensing proceeds by an “EC” pathway, in which electrochemical oxidation of **[Co]** ^**2−**^ (E) precedes a chemical reaction (C) with CN^−^ (*vide supra*). To gain deeper insight into the nature of the chemical step, we next examined the reactivity of **[Pyr]**_**2**_ **[Co]** under oxidizing conditions. Control experiments established that mixtures of **[Pyr]**_**2**_ **[Co]** and [(*n*-Bu)_4_N]­[CN] do not react, as judged by EPR and NMR spectroscopy (Scheme 3A top and Figure S75). By contrast, treatment of **[Pyr]**_**2**_ **[Co]** with 1 equiv of the oxidant “*Magic Blue*” **[NAr**_**3**_ **]­[SbCl**_**6**_ **]** (**[NAr**_**3**_ **] =** tris­(4-bromophenyl)­aminium) generated a transient oxidized species that underwent immediate reaction with cyanide, accompanied by *t*-Bu-BQ ligand dissociation (Scheme 3A, bottom). The IR spectrum of the resulting precipitates displayed a broad ν_C≡N_ band at 2123 cm^−1^, close to that of K_3_[Co­(CN)_6_)] (ν_C≡N_ = 2125 cm^−1^) and consistent with partial cyanide coordination. Separate stoichiometric experiments established the reactivity of free *t-*Bu-BQ ligand with CN^−^, likely involving a cascade of nucleophilic addition reactions.^93^ Taken together, these results indicate that oxidation of the semiquinone radical in **[Co]** ^**2−**^ labilizes the *t*-Bu-BQ ligand and renders the complex susceptible to additional cyanide coordination. This oxidation-induced reactivity provides a plausible basis for the attenuation of the **[Co]** ^**−**^/**[Co]** ^**2−**^ redox process in the presence of cyanide and rationalizes the pronounced selectivity for CN^−^ over other anions.

**Scheme 3:**
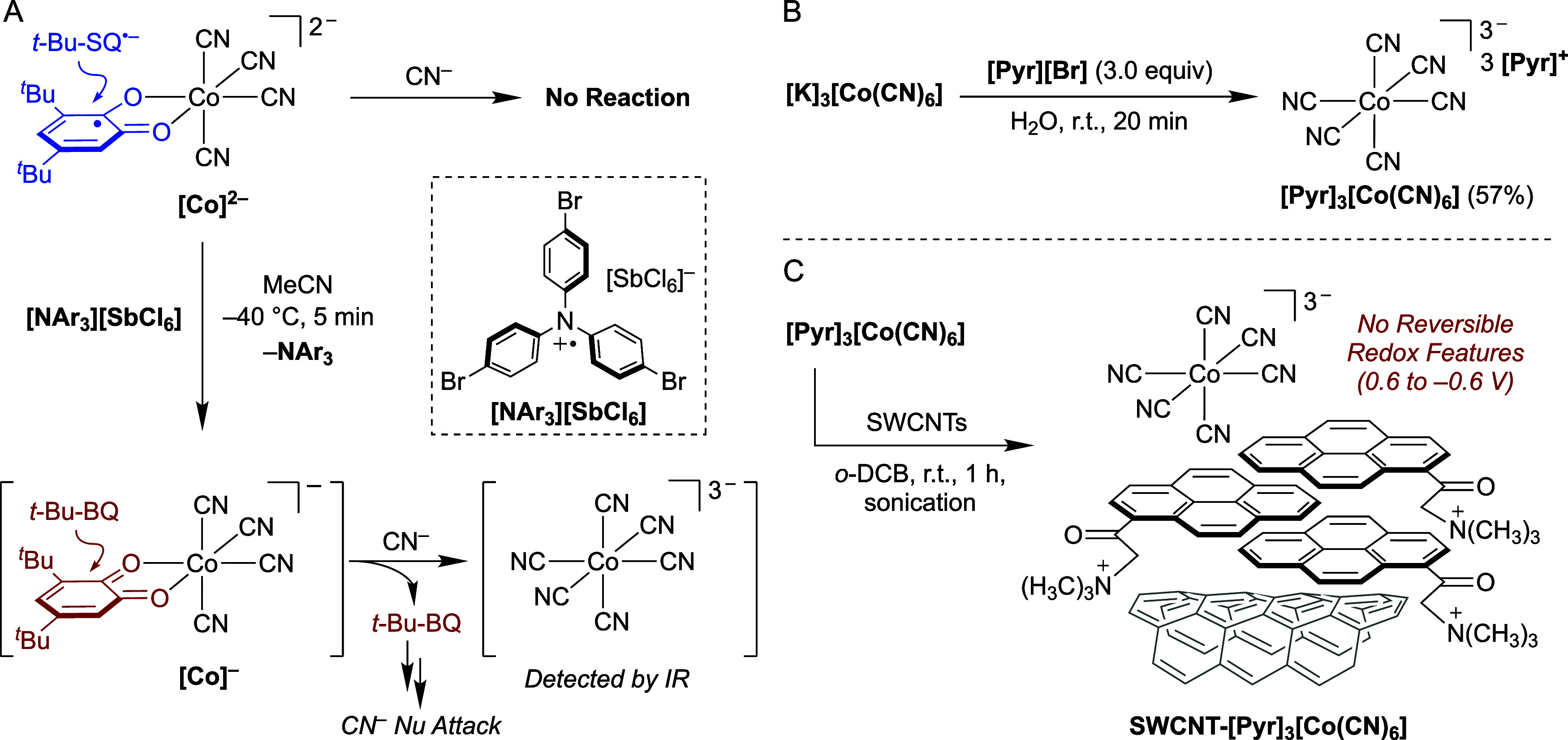
(A) Stoichiometric experiments to probe the reactivity of [Co]^2−^and [Co]^−^with cyanide. Synthesis of (B) [Pyr]_3_[Co­(CN)_6_] and (C) SWCNT-[Pyr]_3_[Co­(CN)_6_].

A notable feature of our proof-of-concept sensing demonstration is that the selector complex **[Pyr]**_**2**_ **[Co]** remains chemically stable in the presence of exogenous cyanide. Whereas many metal-based cyanide sensors operate through direct CN^−^ coordination to the metal center,^94,95^ **[Pyr]**_**2**_ **[Co]** exhibits no such spontaneous interaction, demonstrating that reactivity in this system requires prior electrochemical activation (i.e., redox-switching).^96,97^ This feature underpins a unique sensing mechanism in which electrochemical oxidation of the ligand-centered semiquinone radical (*t*-Bu-SQ^•**−**^) in **[Co]** ^**2−**^ “unmasks” a reactive intermediate that engages CN^−^ to produce a response (Scheme 3A). Importantly, the sensor remains in an inactive “OFF” state until electrochemically activated via the **[Co]** ^**−**^/**[Co]** ^**2−**^ redox couple, thereby providing temporal control over its operation. Crucially, initiating redox-switchable sensing from a persistent radical state avoids the formation of new reactive radical intermediates upon electron transfer, thereby minimizing side reactions and ensuring a selective response. This gated reactivity parallels approaches in switchable molecular catalysis^36,98^ and is expected to extend sensor lifetime by mitigating background sensor degradation. Our approach yields a nanomolar detection limit while offering an additional layer of tunability not accessible with conventional “always-on” recognition elements.^3^

Having established that CN^−^ sensing proceeds by an oxidation-triggered “EC” process involving the ligand-centered semiquinone radical, we next sought to verify that *t*-Bu-SQ^•**−**^ is required for this behavior. To this end, a closed-shell cobalt­(III) analogue lacking a semiquinone radical was prepared by salt metathesis between K_3_[Co­(CN)_6_] and **[Pyr]­[Br]** in aqueous solution, affording **[Pyr]**_**3**_ **[Co­(CN)**_**6**_ **]** as a yellow solid (Scheme 3B). The trianionic cobalt complex, paired with three **[Pyr]** ^**+**^ counter-cations, was immobilized on SWCNTs under conditions identical to those used for **SWCNT-[Pyr]**_**2**_ **[Co]**, yielding **SWCNT-[Pyr]**_**3**_ **[Co­(CN)**_**6**_ **]** (Scheme 3C). XPS, TEM and EDS analyses confirmed comparable cobalt surface coverage, and the Raman D/G band ratio of the nanotube framework remained unchanged relative to **SWCNT-[Pyr]**_**2**_ **[Co]** and pure SWCNTs, consistent with noncovalent functionalization (Figures S20, S32, S37, and S42). In stark contrast to **SWCNT-[Pyr]**_**2**_ **[Co]** however, the closed-shell **SWCNT-[Pyr]**_**3**_ **[Co­(CN)**_**6**_ **]** exhibited no reversible redox features within the electrochemical window of +0.6 to −0.6 V (vs Fc/Fc^+^) and displayed no response to CN^−^ under identical SWV conditions (Figures S29 and S70). These results demonstrate that the cobalt-bound semiquinone radical is indispensable for the reversible one-electron redox process that underlies the sensing signal (Figure 5A,B). The presence of the ligand-centered radical enables a stable, accessible redox couple that can transduce chemical binding events at relatively mild applied potentials, a behavior not observed for the closed-shell cobalt analogue. Overall, our findings underscore the unique potential of redox-switchable radical nanomaterials for highly selective, sensitive, and controllable anion sensing.

Beyond clarifying the role of the radical species in the cyanide sensing mechanism, the preparation of **SWCNT-[Pyr]**_**3**_ **[Co­(CN)**_**6**_ **]** also demonstrates the versatility of the pyrene-anchored electrostatic immobilization strategy. Specifically, its synthesis shows that a chemically distinct, closed-shell cobalt complex can be immobilized on the SWCNT surface using the same pyrene-cation pairing principle as for **SWCNT-[Pyr]**_**2**_ **[Co]**. The presence of only cyanide ligands in the coordination sphere of **[Co­(CN)**_**6**_ **]** ^**3−**^ precludes covalent pyrene attachment, rendering traditional tethering strategies^16^ unsuitable for its immobilization. Our findings highlight that the present “dual-mode” electrostatic/π−π stacking approach provides a convenient route to attach electronically diverse metal complexes on CNT surfaces. The immobilization concept could likely also be extended to other conductive supports, offering a potentially generalizable platform for expediently integrating both open- and closed-shell coordination compounds with electronic materials.

## Conclusions

In summary, a paramagnetic cobalt complex featuring a redox-active ligand and outer-sphere pyrene-functionalized cations was synthesized and noncovalently immobilized on SWCNTs. The molecularly defined surface functionalization of the resulting **SWCNT-[Pyr]**_**2**_ **[Co]** hybrid was established by an array of spectroscopic methods, electron microscopy, and thermogravimetric analysis. EPR spectroscopy revealed uniform surface coverage and electronic interaction between the ligand-centered radical in **[Pyr]**_**2**_ **[Co]** and the SWCNT network. Despite its open-shell nature, the hybrid material remained stable in the presence of air, moisture and light for several months. Electrochemical studies demonstrated the facile and reversible single-electron oxidation of the ligand-centered radical in **[Pyr]**_**2**_ **[Co]**, which was utilized for a redox-triggered reaction with cyanide ions. This reactivity enabled highly sensitive, robust and selective electrochemical cyanide sensing using **SWCNT-[Pyr]**_**2**_ **[Co]**. The ligand-centered radical plays a central role in enabling this reactivity, as its one-electron oxidation at mild potentials provides a robust electrochemical handle underlying the sensing response. Notably, the immobilized complex displayed a detection limit nearly five times lower than the soluble molecular analogue, highlighting that surface confinement on SWCNTs not only stabilizes the radical species but also uniquely enhances sensing performance.

Overall, the noncovalent SWCNT functionalization strategy reported herein enables the modular introduction of metal complexes to nanomaterial surfaces by a combination of electrostatic and π-π interactions. We therefore anticipate that this approach can be extended to access tailored hybrid nanomaterials for sensing a range of gases, ions, and biomolecules^1–3,99,100^ or for developing surface-bound heterogeneous catalysts.^13–16,101^ In addition to sensors, the observed electronic coupling between **[Pyr]**_**2**_ **[Co]** and the SWCNT framework informs the future design of electronically addressable radicals for potential applications in spintronic devices, spin valves, and quantum spin transport systems.^10–12,30–33,102^ Toward these goals, the development of materials that retain radical character upon prolonged exposure to ambient conditions is essential, as demonstrated by the air-stable **SWCNT-[Pyr]**_**2**_ **[Co]** system reported here. Taken together, our findings lay the groundwork for a broader class of redox-active functional nanomaterials that unite open-shell organometallic chemistry with electrically conductive carbon platforms.

## Supplementary Material



## Notes

Experimental details; characterization data for all new compounds; details of stability, electrochemical sensing, and chemical oxidation studies; computational data (PDF)
