# Supplementary material for: Air-Stable Cobalt−Semiquinone Radical Complexes on Carbon Nanotubes: A Redox Switch for Anion Response
Source: J Am Chem Soc. 2025 Dec 4;147(50):46406–17. doi: 10.1021/jacs.5c16011 (PMC12715801; doi:10.1021/jacs.5c16011)
Supplement: Supplementary file 1 [file ja5c16011_si_001.pdf]

Supporting Information

Air-Stable Cobalt–Semiquinone Radical Complexes on Carbon Nanotubes: A Redox Switch for Anion Response

*Sabrina L. Kleynemeyer, Alex M. Wu, Daniel Klose, Yanlin Pan, Jan Reger, Christina Moser and Máté J. Bezdek**

Department of Chemistry and Applied Biosciences, ETH Zürich, Vladimir-Prelog-Weg 1, 8093 Zürich, Switzerland.

*E–mail: mbezdek@ethz.ch

1 Experimental Procedures ... 3
1.1 General Considerations ... 3
1.2 Synthetic Procedures ... 8
2 Characterization Methods ... 19
2.1 Magnetic Moment Determination ... 19
2.2 EPR Spectroscopic Data ... 20
2.3 XPS Data ... 28
2.4 Electrochemical Data ... 34
2.5 Raman Spectroscopic Data ... 43
2.6 TGA/MS Data ... 45
2.7 TEM Imaging ... 46
2.8 EDS Spectroscopic Data ... 51
3 Stability Studies and Control Experiments ... 54
3.1 Stability Studies of **$[Pyr]_2[Co]$** ... 54
3.2 Solvatochromism of **$[Pyr]_2[Co]$** ... 58
3.3 Stability Studies of SWCNT composite materials ... 59
4 Additional Electrochemical Data ... 66
4.1 Electrochemical Cyanide Sensing with **$[Pyr]_2[Co]$** ... 66
4.2 Electrochemical Cyanide Sensing with **$[(n\text{-Bu})_4N]_2[Co]$** ... 67
4.3 Electrochemical Cyanide Sensing with **SWCNT-$[Pyr]_2[Co]$** ... 69
4.4 Selectivity studies: **$[(n\text{-Bu})_4N]_2[Co]$** ... 70
4.5 Selectivity studies: **SWCNT-$[Pyr]_2[Co]$** ... 73
4.6 Theoretical Limit of Detection (LOD) Determination ... 75
5 Optimization and Control Experiments for Electrochemical Sensing Studies ... 77
5.1 Reproducibility of Drop-Casting Technique ... 77
5.2 Robustness of **SWCNT-$[Pyr]_2[Co]$** Surface Functionalization under Electrochemical Cycling Conditions ... 79
5.3 Response of **SWCNT-$[Pyr]_2[Co]$** Electrodes to Sequential and Prolonged Cyanide Exposure ... 80
5.4 Attempted Cyanide Sensing with **SWCNT-$[Pyr]_3[Co(CN)_6]$** ... 82
6 Chemical Oxidation of $[Pyr]_2[Co]$ ... 83
6.1 Chemical Synthesis of **$[Co]^-$** ... 83
6.2 Reaction of Chemically Generated **$[Co]^-$** with $[(n\text{-Bu})_4N][CN]$ ... 84
7 Appendix ... 88
7.1 NMR Spectra ... 88
7.2 UV-Vis Spectra ... 95
7.3 IR Spectra ... 100
7.4 X-Ray Crystallographic Data ... 103
8 DFT Computations ... 107
8.1 General Considerations ... 107
8.2 Ground-State Energies ... 109
8.3 Time-Dependent (TD) DFT Calculations ... 110
9 References ... 118

1 Experimental Procedures

1.1 General Considerations

Where stated, reactions and purification procedures were performed following standard Schlenk techniques under an argon atmosphere, using an oil pump vacuum of up to 1×10^{-3} mbar and dried glassware by flame-drying under vacuum. Where necessary, an argon filled *MBraun LabMaster Pro* glovebox (H_2O < 0.1 ppm, O_2 < 0.1 ppm) was used to manipulate highly sensitive compounds. Room temperature refers to T = 23 °C, while elevated reaction temperatures are referred to the respective oil bath's temperature. SWCNT-containing samples were sonicated in a *Branson 3800 series* ultrasonic bath and centrifuged in *Eppendorf* tubes with an *Eppendorf 5418* centrifuge at 16873 g (14'000 rpm).

Solvents and Chemicals: Molecular sieves were thermically activated by heating under high vacuum prior to use. Dry tetrahydrofuran (THF), acetonitrile (MeCN) and toluene (PhMe) were collected from a solvent purification system, *MB SPS 7* (*MBraun*), and additionally stored over 3 Å (MeCN) or 4 Å (THF, PhMe) molecular sieves prior to use. THF-d_8 was dried over sodium, degassed *via* three freeze-pump-thaw cycles, distilled and stored over 4 Å molecular sieves. MeCN-d_3 was dried over CaH_2, degassed *via* three freeze-pump-thaw cycles, distilled and stored over 3 Å molecular sieves. 3,5-Di-*tert*-butyl-1,2-benzoquinone (*t*-Bu-BQ; *Tokyo Chemical Industry* (TCI)), dicobalt octacarbonyl (*Thermo Scientific – Acros Organics* (ACR)) , 1-(bromoacetyl)pyrene (*Sigma-Aldrich*) and cetyltrimethylammonium bromide (*Sigma-Aldrich*) were used without further purification. Tripotassium hexacyanocobaltate(III) (*Sigma-Aldrich*) was recrystallized from H_2O and dried *in vacuo* at 80 °C for 16 h prior to use. SWCNTs (P3-SWCNT, > 90% carbonaceous purity, 5–7 wt% metal content, 500 nm–1.5 µm bundle length, 4–5 nm bundle diameter, 1.55±0.1 nm individual tube diameter, lot #03-A036; purified by nitric acid treatment by the supplier) were purchased from *Carbon Solutions, Inc.* All experiments involving samples of pure SWCNTs were subjected to the same purification steps as the respective composite material, consisting of sonication for 1 h in *ortho*-dichlorobenzene (*o*-DCB) and several washings with MeOH, if not stated otherwise.

Nuclear Magnetic Resonance (NMR) Spectroscopy: NMR spectroscopic measurements spectra were recorded on *Bruker* (*AVANCE 400*, *AVANCE 500*). Unless otherwise stated, the spectroscopic data was obtained at T = 298 K. Acetonitrile-d_3 (MeCN-d_3), dimethylsulfoxide-d_6 (DMSO-d_6), methanol-d_4 (CD_3OD) and tetrahydrofuran-d_8 (THF-d_8) were used as deuterated solvents. The chemical shifts are given in ppm (parts per million) and are referenced in the ^{1}H NMR spectra to the respective residual protonated solvent.[1] The assignment of the obtained spectra was done using *MestReNova* v14.0.0-23239 (*MestReLab Research*). The following abbreviations were used for the description of spin multiplicities, whereby combinations were used when needed: s (singlet), d (doublet), t (triplet), q (quartet), qu (quintet), sext (sextet), m (multiplet), br (broad) for the description of (partially) paramagnetic compounds. Coupling constants J are given in Hertz (Hz). The signal

assignments refer to the numbering in the respective compounds and were corroborated by 2D NMR spectroscopy where necessary ($^{1}H/^{1}H$ COSY, $^{1}H/^{13}C$ HMQC/HMBC).

Electron Paramagnetic Resonance (EPR) Spectroscopy: Continuous wave (CW) X-Band EPR spectroscopic data at room temperature were collected either on a *Magnettech MS-5000* benchtop EPR spectrometer or on a *Bruker* ELEXSYS E500 using a *Bruker* Super-High-Q resonator, and simulations were calculated using the *EasySpin* toolbox for *MATLAB*.[2,3] Spectra used for quantitative comparisons were recorded using identical measurement parameters. Identical samples measured at different timepoints to probe for decomposition processes in the hybrid materials were normalized to the intrinsic SWCNT signal (*vide infra*). For solution-state measurements at room temperature, a solvent mixture of PhMe/MeOH (3:1) and 3 mm or 5 mm (outer diameter) quartz tubes were used.
Pulse EPR experiments in X-band (ca. 9.8 GHz) were performed at 5 K using a *Bruker ELEXSYS E580 EPR* spectrometer equipped with a *Bruker Dielectric MD-5* microwave resonator and an *Oxford Instruments* helium flow cryostat. Echo-detected EPR spectra were acquired using the standard Hahn echo pulse sequence π/2–τ–π–τ-echo with 16/32 ns pulse lengths and an interpulse delay of 200 ns with a shot repetition time of 2 ms and a 2-step phase cycle to cancel out receiver offsets. 2-pulse ESEEM experiments were recorded with the same Hahn echo sequence by incrementing the interpulse delay τ from 128 ns in 512 steps of 8 ns. 3-pulse ESEEM data were acquired with the sequence π/2–τ–π/2–T–π/2–τ–echo, while incrementing the delay T from 128 ns in 1024 steps of 8 ns, using the standard 4-step phase cycle to remove unwanted coherence transfer pathways. Inversion recovery data were recorded using the standard pulse sequence π–T–π/2–τ–π–τ–echo using a 200 ns interpulse delay τ and incrementing the delay after inversion, T, from 1 µs in 512 steps of 12 µs at a shot repetition time of 8 ms. From 2-pulse ESEEM and inversion recovery time traces the phenomenological relaxation times T_2 and T_1, respectively, were determined as the 1/e decay times of the data. Nutation experiments, also known as Rabi Oscillations, were acquired using the pulse sequence tp-T-π/2–τ–π–τ-echo by incrementing the pulse length tp from 0 ns in 100 steps of 4 ns followed by a fixed delay T = 1 µs with a shot repetition time of 8 ms. The pulse EPR data was processed and visualized using the *EasySpin* toolbox for *MATLAB*[2] using custom-written Matlab scripts. Solution-state samples were measured in a solvent mixture of PhMe/MeOH (3:1), filled into 3 mm (outer diameter) quartz tubes and shock frozen in liquid nitrogen. All solid-state samples were measured in 3 mm (outer diameter) quartz tubes.

Infrared (IR) Spectroscopy: IR spectra were recorded using a *Bruker Alpha Platinum-ATR* and *Bruker Tensor II* IR spectrometer. All IR spectra shown represent attenuated total reflection (ATR) spectra measured on solid samples and were baseline corrected using the *Peak Analyzer* Tool in *Origin2025*, using 2nd derivative anchor point finding, adjacent-averaging smoothing and interpolation.

Ultraviolet-Visible (UV-Vis) Spectroscopy: UV-Vis spectra were recorded using an *Agilent Cary5000* using Teflon-capped quartz cuvettes (10 mm path length), at concentrations ranging 25–60 µM.

High-Resolution Mass Spectrometry (HRMS): High-resolution mass spectrometric analyses were conducted by the Molecular and Biomolecular Analysis Service (MoBiAS) in the Laboratory of Organic Chemistry (LOC) at ETH Zürich on a *Varian IonSpec Spectrometer* for electrospray ionization (ESI). A detailed assignment of the fragmentation was not undertaken and solely the peaks of the molecule ions or a characteristic fragment are noted herein. Molecular ions $[M]^+$ are reported in m/z (mass-to-charge ratio).

X-Ray Photoelectron Spectroscopy (XPS): XPS measurements were performed on a *Sigma II* instrument by *Thermo Electron*, equipped with an *Alpha 110* hemispherical analyzer. The large area XPS mode, using a non-monochromatic Al Kα X-ray source (1486.6 eB) at 200 W, was applied. All samples were prepared under ambient conditions, using cleaned p-doped Si wafer substrates (10 x 10 mm, *Electron Microscope Sciences*, 71893-12). The pressure in the XPS analysis chamber was kept below 5.0 x 10^{-8} mbar during all measurements. Survey scan spectra were collected at the beginning of each measurement, up to a binding energy of 1100 eV, using a pass energy of 50 eV, a step size of 1.0 eV, and a dwell time of 50 ms. Narrow region scans were collected using a pass energy of 20 eV, a step size of 0.10 eV, and a dwell time of 50 ms. The raw data was analyzed with the *CasaXPS* software.[4] Spectra collected of cobalt complex **$[Pyr]_2[Co]$** were calibrated to the C 1s peak at 284.6 eV. Due to their high conductivity, spectra collected of samples containing SWCNTs were not charge corrected.

Transmission Electron Microscopy (TEM) Imaging and Energy Dispersive Spectroscopy (EDS): A double Cs-corrected *Jeol ltd. JEM-ARM300F GRAND ARM* with cold field emission gun was used for TEM imaging and EDS were used for samples of pure SWCNTs, **SWCNT-$[Pyr]_2[Co]$** and **SWCNT-[Pyr][Br]**. Images were recorded on a *Gatan 1View* camera (16M pixel CMOS@4kx4k). In the case of **SWCNT-$[CTA]_2[Co]$** and **SWCNT-$[Pyr]_3[Co(CN)_6]$**, atomic-resolution images were acquired on a double-aberration-corrected *Jeol ltd. GRAND ARM “Vortex”* microscope operated at 300 kV. EDS mapping was performed on a *Joel ltd. JEM-F200* microscope operated at 200 kV and equipped with a dual EDS system consisting of two large-area SDD detectors (100 mm^2 active area each; total solid angle: 1.7 sr).

Raman Spectroscopy: Raman spectra were recorded using a *Thermo Scientific DXR3* Raman microscope with a 633 nm excitation LASER. Five individual spectra were recorded at different locations on the sample surface, which were averaged to yield the final spectra, normalized to G-band intensity. All SWCNT-containing samples were recorded as powdery films on nylon membrane filters (0.2 µm).

Thermogravimetric Analysis/Mass Spectrometry (TGA/MS) and Differential Thermo-gravimetric Analysis (DTG): TGA/MS data was collected on a *Netzch STA 449 F5 Jupiter*

System coupled to a *QMS403 D Aëolos Quadro* mass spectrometer. Measurements were carried out under an argon flow of 30 mL/min. All samples were dried *in vacuo* prior to measurements, and then heated at a rate of 10 °C/min, from T = 23 °C to T = 800 °C. TGA/MS data was processed using the *Proteus* software for thermal analyses.

X-ray diffraction (XRD) analysis: The intensities for the molecular structure determinations in the single crystals of **$[Pyr]_2[Co]$** and **$[Pyr]_3[Co(CN)_6]$** were collected on a *Bruker D8 Venture Dual Source* diffractometer with a *Photon II* detector at a temperature of T = 100 K. A CuK_α (λ = 1.54178 Å) X-ray source with a graphite monochromator was used. For data reduction and absorption correction, standard procedures were applied. All structure determinations were calculated using Olex2.[5] The structure solution was performed with *SHELXT* and the structure refinement with *SHELXL*. Disorders were treated with *PART* and *EADP*. Hydrogen atoms were calculated for idealized positions and treated with the "riding model" option of *SHELXL*.[6] Ellipsoid representations were drawn using *Mercury* 2022.2.0 for Windows.

General Methods for Electrochemical Experiments: Electrochemical-grade MeCN purchased from *Sigma-Aldrich* was stored over 3 Å molecular sieves. 1,2-Difluorobenzene (1,2-DFB) was dried over CaH_2, distilled, degassed *via* three freeze-pump-thaw cycles and stored over 4 Å molecular sieves. $[(n\text{-Bu})_4N][PF_6]$ was purchased from *Fluorochem*, recrystallized from hot ethanol (EtOH) twice and dried *in vacuo* at 80 °C for 48 h. All $[(n\text{-Bu})_4N][X]$ (X = CN, Cl, Br, I, SCN, NO_2, NO_3) salts were purchased from *Sigma-Aldrich*. $[(n\text{-Bu})_4N][CN]$ was recrystallized from hot ethyl acetate (EtOAc) twice and dried *in vacuo* at r.t. for 16 h. $[(n\text{-Bu})_4N][I]$ and $[(n\text{-Bu})_4N][SCN]$ were recrystallized from hot EtOH and dried *in vacuo* at r.t. for 16 h. All other tetrabutylammonium (TBA) salts were used as purchased without further purification, but equally dried *in vacuo* at r.t. for 16 h. Glassy carbon electrodes for thin-layer CV studies of **SWCNT-$[Pyr]_2[Co]$** were prepared by drop-casting a single drop of an *o*-DCB dispersion of the composite (washed with MeOH once; see more details in Sections 1.2.7–1.2.8) on top of the electrode *via* a Pasteur pipette and drying the electrode *in vacuo* at r.t. for 1 h. This procedure was repeated once, yielding an even layer of the composite on top of the electrode. The acquired CV data was referenced against the Fc/Fc^+ couple and smoothened using the Savitzky-Golay method where necessary.

Cyclic Voltammetry (CV): CV studies were performed in an argon-filled *MBraun UniLab* glovebox using a *Gamry Interface 1010E* potentiostat/galvanostat/ZRA with a three-electrode electrochemical cell. Unless stated otherwise, an $Ag/AgNO_3$ (0.10 M $[(n\text{-Bu})_4N][PF_6]$ in MeCN, *BASi*) reference electrode, a glassy carbon disk (1.6 mm^2, *eDAQ*) working electrode (GCE) and a Pt-wire counter electrode were used. The working electrode was thoroughly polished before each solution-state experiment or drop-casting procedure with abrasive papers (P2400, P4000, P8000, P12000; in that order) and finally an alumina slurry (0.05 µm) and rinsed sequentially with *o*-DCB, *i*-PrOH, acetone and *Millipore* water. CV studies were conducted in 1,2-dimethoxyethane (DME) (**$[Pyr]_2[Co]$ and $[CTA]_2[Co]$**), or 1,2-DFB (**$[(n\text{-Bu})_4N]_2[Co]$**, **$[Pyr]_3[Co(CN)_6]$** and **SWCNT-$[Pyr]_2[Co]$**) in a $[(n\text{-Bu})_4N][PF_6]$ (0.10 M) supporting electrolyte

solution in the respective solvent. At the beginning of each experiment, a background scan of a blank sample containing electrolyte and solvent was performed to determine the solvent window and ensure a stable potential and the absence of contaminants. Next, the respective compound (1.0 mM) was dissolved in the electrolyte solution, or the hybrid material was drop-cast onto the working electrode (for a detailed procedure, see Section 1.2.8). To determine the half wave potentials of complexes **[(*n*-Bu)$_4$N]$_2$[Co]** and **[Pyr]$_2$[Co]** and the hybrid material **SWCNT-[Pyr]$_2$[Co]**, two scans of the full window starting at the open circuit potential (OCP) were recorded. The first scan is reported.

Electrochemical Sensing Studies: At the beginning of all electrochemical sensing studies, a background scan of [(*n*-Bu)$_4$N][PF$_6$] (0.10 M in 1,2-DFB) supporting electrolyte solution was recorded. For CV studies, one scan of the oxidation window per addition of analyte (12.5 mM in 1,2-DFB for studies with **[(*n*-Bu)$_4$N]$_2$[Co]**; 25 mM in 1,2-DFB for studies with **SWCNT-[Pyr]$_2$[Co]**) was recorded. Additionally, square wave voltammetry (SWV) studies were conducted on the anodic feature of the respective compound. Prior to all measurements, (for both solution-state and thin-film) the potential was held at 1.00 V (vs Ag/AgNO$_3$ (0.10 M [(*n*-Bu)$_4$N][PF$_6$] in MeCN, *BASi*) reference electrode) for 1 min, before scanning to −0.5 V vs ref with a pulse height (P_H) of 25.0 mV, a pulse width (P_W) of 50.0 ms and a scan height (S_H) of −10.0 mV, resulting in a scan rate of 100 mV s^{-1}. The obtained SW voltammograms were baseline corrected and integrated using the *Peak Analyzer* Tool in *Origin2025*, using 2nd derivative anchor point finding, adjacent-averaging smoothing and interpolation for the baseline creation. Within one data set, the anchor points were kept constant to ensure comparability of the integration results between individual runs.

1.2 Synthetic Procedures

1.2.1 *N,N,N*-Trimethyl-2-oxo-2-(pyren-1-yl)ethan-1-ammonium bromide (**[Pyr][Br]**)

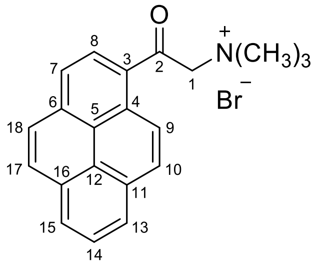

[Pyr][Br]
$C_{21}H_{20}NOBr$
M = 382.30 g/mol

[Pyr][Br] was prepared according to a modified literature procedure.[7] A 250 mL round-bottomed flask was charged with a magnetic stir bar and 2-bromo-1-(pyren-1-yl)ethan-1-one (1.90 g, 5.88 mmol, 1.00 equiv) was dissolved in THF (150 mL). Trimethylamine (4.20 M in EtOH, 2.10 mL, 8.82 mmol, 1.50 equiv) was added to the stirring solution, leading to the immediate precipitation of a pale-yellow solid. The reaction mixture was subsequently stirred for 16 h at room temperature. The obtained suspension was filtered and the residue was washed with ice-cold THF (2 x 15 mL) and dried *in vacuo*. **[Pyr][Br]** (2.25 g, 5.88 mmol, 99%) was obtained as a pale yellow powder.

^{1}H NMR (500 MHz, DMSO-d_6, 298 K): δ = 8.92 (d, $^3J_{H17–H18}$ = 9.4 Hz, 1H, *H*17), 8.65 (d, $^3J_{H8–H7}$ = 8.2 Hz, 1H, *H*8), 8.49–8.46 (m, 3H, overlapping *H*7, *H*13, *H*15), 8.46 (d, $^3J_{H18–H17}$ = 9.6 Hz, 1H, *H*18), 8.42 (d, $^3J_{H9–H10}$ = 8.9 Hz, 1H, *H*9), 8.32 (d, $^3J_{H10–H9}$ = 9.0 Hz, 1H, *H*10), 8.21 (t, $^3J_{H14–H13/H15}$ = 7.6 Hz, 1H, *H*14), 5.55 (s, 2H, *H*1), 3.46 (s, 9H, C*H*$_3$) ppm.

^{13}C{^{1}H} NMR (126 MHz, DMSO-d_6, 298 K): δ = 194.9 (*C*2), 134.4 (*C*3), 130.6 (*C*11), 130.5 (*C*9), 130.3 (*C*18), 129.8 (*C*16), 129.2 (*C*5), 128.4 (*C*6), 127.3 (*C*10), 127.2 (*C*13 or *C*15), 127.1 (*C*14), 127.0 (*C*8), 126.7 (*C*13 or *C*15), 124.3 (*C*7), 124.0 (*C*4), 124.0 (*C*17), 123.2 (*C*12), 68.8 (*C*1), 53.5 (3C, *C*H$_3$) ppm.

UV-Vis: λ_{max} (CH_2Cl_2) = 204, 234, 288, 367 nm.

IR (ATR): ṽ = 3010 (w), 2873 (w), 1675 (m), 1593 (m), 1389 (m), 1213 (m), 917 (s), 849 (s) cm^{-1}.

HRMS-ESI: *m/z* calculated for $C_{21}H_{20}NO^+$ $[M-Br]^+$: 302.1540. Found: 302.1538.

1.2.2 [(*t*-Bu-SQ)$_2$Co]$_4$

The cobalt complex [(*t*-Bu-SQ)$_2$Co]$_4$ was prepared according to a modified literature procedure.[8] In an argon-filled glovebox, a 250 mL round-bottomed flask was charged with a magnetic stir bar, [$Co_2(CO)_8$] (1.25 g, 3.67 mmol, 1.00 equiv) and PhMe (40 mL). A solution containing 3,5-di-*tert*-butyl-*o*-benzoquinone (*t*-Bu-BQ) (3.23 g, 14.7 mmol, 4.00 equiv) dissolved in PhMe (40 mL) was slowly added over the course of 30 min. The reaction mixture was subsequently stirred at room temperature for 4 h. The resulting suspension was filtered, the residue was washed once with cold PhMe (−35 °C, 15 mL) and dried *in vacuo* to afford the product [(*t*-Bu-SQ)$_2$Co]$_4$ as a blue powder (3.36 g, 1.68 mmol, 92%).

^{1}H NMR (300 MHz, THF-d_8, 298 K): δ = 5.35 (br) ppm.

UV-Vis: λ_{max} (THF) = 233, 310 nm.

IR (ATR): ṽ = 2949 (m), 2905 (w), 2865 (w), 1581 (m), 1474 (m), 1441 (s), 1357 (m) cm^{-1}.

1.2.3 $[(n\text{-Bu})_4N]_2[(t\text{-Bu-SQ})Co(CN)_4]$ (**$[(n\text{-Bu})_4N]_2[Co]$**)

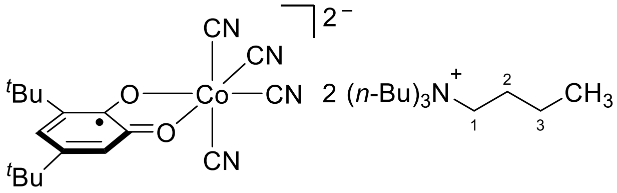

$[(n\text{-Bu})_4N]_2[Co]$
$C_{50}H_{92}N_6O_2Co^{\bullet}$
M = 868.26 g/mol

The air- and moisture-stable complex **$[(n\text{-Bu})_4N]_2[Co]$** was prepared according to a literature procedure.[9] In an argon-filled glovebox, a 100 mL round-bottomed flask was charged with a magnetic stir bar, $[(t\text{-Bu-SQ})_2Co]_4$ (2.95 g, 1.48 mmol, 1.00 equiv) and THF (40 mL). While stirring, $[(n\text{-Bu})_4N][CN]$ (3.17 g, 11.8 mmol, 8.00 equiv) was added in one portion. The reaction mixture was subsequently stirred for 16 h at room temperature. The resulting dark purple suspension was filtered, the residue was washed once with cold THF (−35 °C, 15 mL) and then dried *in vacuo*. **$[(n\text{-Bu})_4N]_2[Co]$** (1.93 g, 2.23 mmol, 38%) was obtained as a purple, crystalline powder.

^{1}H NMR (300 MHz, DMSO-d_6, 298 K): δ = 3.17 (s, 8H, *H*1), 1.57 (s, 8H, *H*2), 1.32 (s, 8H, *H*3), 0.94 (s, 12H, CH_3) ppm.

^{13}C{^{1}H} NMR (300 MHz, DMSO-d_6, 298 K): δ = 57.5 (4C, *C*1), 23.0 (4C, *C*2), 19.2 (4C, *C*3), 13.5 (4C, CH_3) ppm.

EPR (PhMe/MeOH 3:1, 298 K): g = 2.0024; A^{Co} = 22.73 MHz, A^{H} = 8.15 MHz.

UV-Vis: λ_{max} (CH_2Cl_2) = 229 (ε = 3540 $M^{-1}\,cm^{-1}$), 321, 503 nm.

IR (ATR): ṽ = 2959 (s), 2873 (m), 2121 (m), 1580 (w), 1484 (s), 1452 (s), 1439 (s) cm^{-1}.

HRMS-ESI: *m/z* calculated for $C_{16}H_{36}N^+$ [M−**$[(n\text{-Bu})_4N][Co]$**]$^+$: 242.2843. Found: 242.2842.

1.2.4 [Pyr]$_2$[(*t*-Bu-SQ)Co(CN)$_4$] (**[Pyr]$_2$[Co]**)

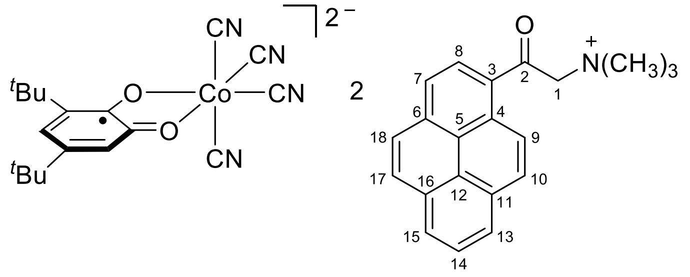

[Pyr]$_2$[Co]
$C_{60}H_{60}N_6O_4Co^{\bullet}$
M = 988.11 g/mol

A 2 L round-bottomed flask was charged with a magnetic stir bar, **[Pyr][Br]** (880 mg, 2.30 mmol, 2.00 equiv) and deionized water (650 mL). A separate 1 L round-bottomed flask was charged with a magnetic stir bar, **[(*n*-Bu)$_4$N]$_2$[Co]** (1.00 g, 1.15 mmol, 1.00 equiv) and deionized water (750 mL). Both suspensions were stirred at room temperature until the solids were fully dissolved. Then, the solution of **[(*n*-Bu)$_4$N]$_2$[Co]** was added to the stirring solution of **[Pyr][Br]**, leading to the immediate precipitation of a maroon-colored solid. The suspension was stirred for an additional 15 min before being filtered over a sintered-glass frit (P4). The red, pasty residue was washed with deionized water (3 x 100 mL) and dried *in vacuo*, to yield **[Pyr]$_2$[Co]** (900 mg, 908 µmol, 87%) as a red powder. Single crystals suitable for X-ray diffraction studies were obtained by slow evaporation of a concentrated MeCN solution at room temperature.

^{1}H NMR (300 MHz, DMSO-d_6, 298 K): δ = 8.95–8.92 (br m, 1H, *H*17), 8.49–8.41 (br m, 6H, overlapping *H*7, *H*8, *H*9, *H*13, *H*15, *H*18), 8.32–8.30 (br m, 1H, *H*10), 8.21 (br t, 1H, *H*14), 5.52 (br s, 2H, *H*1), 3.47 (br s, 9H, C*H*$_3$) ppm.

^{13}C{^{1}H} NMR (101 MHz, DMSO-d_6, 298 K): δ = 194.8 (*C*2), 134.3 (*C*3), 130.5 (*C*11), 130.4 (*C*9), 130.3 (*C*18), 129.7 (*C*16), 129.1 (*C*5), 128.3 (*C*6), 127.2 (2C, *C*10, *C*13 or *C*15), 127.1 (*C*14), 126.7 (2C, *C*8, *C*13 or *C*15), 124.5 (*C*7), 123.9 (2C, *C*4, *C*17), 123.0 (*C*12), 68.7 (*C*1), 53.8 (3C, *C*H$_3$) ppm.

EPR (PhMe/MeOH 3:1, 298 K): g = 2.0024; A^{Co} = 22.53 MHz, A^{H} = 8.17 MHz.

UV-Vis: λ_{max} (CH_2Cl_2) = 203, 231 (ε = 1.16 x 10^5 M^{-1} cm^{-1}), 288, 313, 366, 475 nm.

IR (ATR): ṽ = 3042 (w), 2957 (m), 2868 (w), 2121 (m), 1454 (s), 913 (s), 846 (s) cm^{-1}.

HRMS-ESI: *m/z* calculated for $C_{21}H_{20}NO^+$ [M–**[Pyr][Co]**]$^+$: 302.1539. Found: 302.1538.

1.2.5 [CTA]$_2$[(*t*-Bu-SQ)Co(CN)$_4$] (**[CTA]$_2$[Co]**)

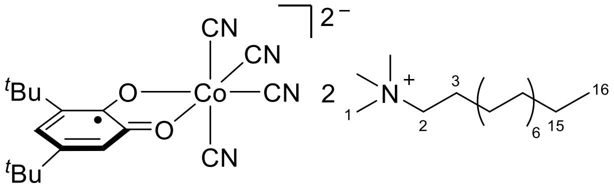

[CTA]$_2$[Co]
$C_{56}H_{104}N_6O_2Co^{\bullet}$
M = 952.42 g/mol

A 500 mL round-bottomed flask was charged with a magnetic stir bar, **[(*n*-Bu)$_4$N]$_2$[Co]** (786 mg, 0.905 mmol, 1.00 equiv), cetyltrimethylammonium bromide (CTAB; 660 mg, 1.81 mmol, 2.00 equiv) and CH_2Cl_2 (100 mL). The mixture was stirred for 20 min at room temperature, after which deionized water (100 mL) was added. After an additional 10 min of stirring, the mixture was transferred into a separatory funnel and the phases were separated. The organic phase was washed with deionized water (5 x 100 mL), dried over $MgSO_4$ and filtered. Evaporation of the solvent *in vacuo* afforded **[CTA]$_2$[Co]** (840 mg, 0.882 mmol, 97%) as a deep red-purple, flakey solid.

^{1}H NMR (500 MHz, DMSO-*d*$_6$, 298 K): δ = 3.07 (br s, 11H, *H*1, *H*2), 1.67 (br s, 2H, *H*3), 1.24 (br s, 26H, *H*4–*H*15), 0.85 (br s,3H, *H*16) ppm.

^{13}C{^{1}H} NMR (101 MHz, DMSO-*d*$_6$, 298 K): δ = 65.3 (*C*2), 52.4 (*C*1), 31.2 (*C*14), 29.0, 28.9, 28.8, 28.6 (*C*5–*C*13), 25.9 (*C*4), 22.1 (*C*3 or *C*15), 22.0 (*C*3 or *C*15), 13.9 (*C*16) ppm.

EPR (PhMe/MeOH 3:1, 298 K): g = 2.0024; A^{Co} = 22.60 MHz, A^H = 8.15 MHz.

UV-Vis: λ_{max} (CH_2Cl_2) = 319 (ε = 1.19 x 10^6 M^{-1} cm^{-1}), 495 nm.

IR (ATR): ṽ = 2957 (m), 2920 (s), 2851 (s), 2121 (m) 1576 (w), 1466 (m), 1445 (s) cm^{-1}.

HRMS-ESI: *m/z* calculated for $C_{19}H_{42}N^+$ [M–**[CTA][Co]**]$^+$: 284.3312. Found: 284.3310.

1.2.6 **[Pyr]$_3$[Co(CN)$_6$]**

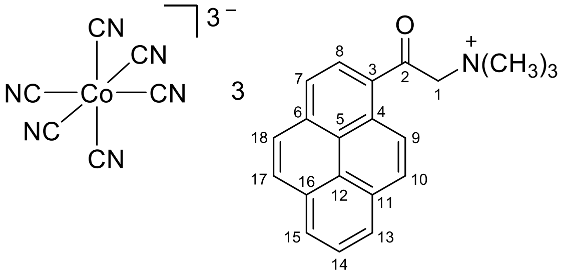

[Pyr]$_3$[Co(CN)$_6$]
$C_{69}H_{60}N_9O_3Co$
M = 1122.23 g/mol

A 1 L round-bottomed flask was charged with a magnetic stir bar, **[Pyr][Br]** (1.00 g, 2.62 mmol, 3.00 equiv) and distilled water (600 mL). The mixture was stirred for 20 min at room temperature, until all solids were dissolved. In a second 25 mL round-bottomed flask, $K_3[Co(CN)_6]$ (290 mg, 0.872 mmol, 1.00 equiv) was dissolved in distilled water (20 mL). This solution was added to the first one, leading to the immediate precipitation of a bright yellow solid. The mixture was stirred for 20 min at r.t. and then centrifuged at 19872 g (15'000 rpm) for 10 min. The supernatant was replaced with fresh H_2O, the dispersion was mixed by vigorous shaking, and centrifuged again. This step was repeated once, after which the residues were dissolved in DMF and combined in a 50 mL round-bottomed flask. The solvent was removed *in vacuo* and the residue was additionally dried *in vacuo* at 100 °C for 16 h, yielding **[Pyr]$_3$[Co(CN)$_6$]** (516 mg, 0.459 mmol, 57%) as a bright-yellow solid. Single crystals suitable for X-ray diffraction studies were obtained by slow evaporation of a concentrated DMF solution at room temperature.

^{1}H NMR (300 MHz, DMSO-d_6, 298 K): δ = δ = 8.90 (d, $^3J_{H17–H18}$ = 9.4 Hz, 1H, *H*17), 8.68 (d, $^3J_{H8–H7}$ = 8.2 Hz, 1H, *H*8), 8.48–8.43 (m, 4H, overlapping *H*7, *H*13, *H*15, *H*18), 8.39 (d, $^3J_{H9–H10}$ = 9.4 Hz, 1H, *H*9), 8.27 (d, $^3J_{H10–H9}$ = 8.9 Hz, 1H, *H*10), 8.19 (t, $^3J_{H14–H13/H15}$ = 7.7 Hz, 1H, *H*14), 5.56 (s, 2H, *H*1), 3.49 (s, 9H, C*H*$_3$) ppm.

^{13}C{^{1}H} NMR (101 MHz, DMSO-d_6, 298 K): δ = 195.3 (*C*2), 134.5 (*C*3), 130.8 (*C*11), 130.6 (*C*9), 130.5 (*C*18), 130.1 (*C*16), 129.4 (*C*5), 128.7 (*C*6), 127.5 (*C*10), 127.4 (2C, *C*13 or *C*15, *C*14), 127.3 (*C*8), 126.9 (*C*13 or *C*15), 124.7 (*C*7), 124.3 (2C, *C*4, *C*17), 123.5 (*C*12), 69.1 (*C*1), 53.9 (3C, *C*H$_3$) ppm.

UV-Vis: λ_{max} (DMSO) = 289 (ε = 5.7 x 10^6 M^{-1} cm^{-1}), 368 nm.

IR (ATR): ṽ = 3437 (br), 3038 (w), 2967 (w), 2127 (s), 1670 (s), 1592 (m), 918 (s), 846 (s) cm^{-1}.

HRMS-ESI: *m/z* calculated for $C_{21}H_{20}NO^+$ [M–**[Pyr]$_2$[Co(CN)$_6$]**]$^+$: 302.1539. Found: 302.1534.

1.2.7 SWCNT-[Pyr]$_2$[Co]

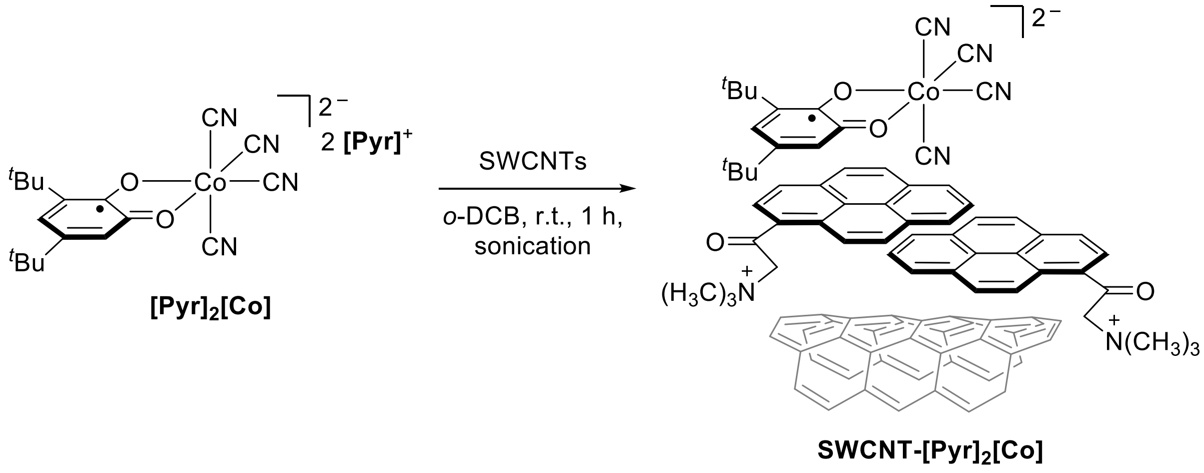

A 5 mL scintillation vial was charged with SWCNTs (1.00 mg), **[Pyr]$_2$[Co]** (1.00 mg, 1.00 µmol) and *o*-DCB (2.0 mL). The vial was sealed with *Parafilm*® and then mounted by its cap into an ultrasonic bath. The dispersion was sonicated for 60 min, transferred into an *Eppendorf* tube and centrifuged for 10 min. The supernatant was decanted away.

Purification: The residue was washed with MeOH (1.5 mL). The sample was then centrifuged again and the washing steps were repeated until the supernatant MeOH solution remained colorless (min. 3x).

Note: SWCNT-[CTA]$_2$[Co] and **SWCNT-[Pyr]$_3$[Co(CN)$_6$]** were prepared according to an identical procedure, using **[CTA]$_2$[Co]** (1.00 mg, 1.05 µmol) or **[Pyr]$_3$[Co(CN)$_6$]** (1.00 mg, 0.891 µmol), respectively. Samples of **SWCNT-[CTA]$_2$[Co]** were only washed twice during the purification step to avoid material loss, as the surface functionalization was found to be labile. Control samples containing SWCNTs (1.00 mg) and either **[Pyr][Br]** (1.00 mg, 3.00 µmol) or **[(*n*-Bu)$_4$N]$_2$[Co]** (1.00 mg, 1.15 µmol) were also prepared according to an identical procedure.

1.2.8 Sample Preparation Procedures

EPR Spectroscopy:

Room Temperature (298 K) Measurements: SWCNT-containing samples for EPR spectroscopic measurements were prepared by flushing 1.00 mg of the respective material into a quartz EPR tube (3.00 or 5.00 mm outer diameter) using CH_2Cl_2 (0.25 or 0.50 mL). If needed, the CH_2Cl_2 was then removed *in vacuo*.

Low Temperature (5 K) Measurements: For an in-depth EPR spectroscopic investigation of the composite material at low temperature, five different samples were prepared and compared (Table S1). The spin-diluted sample (entry 3) was used to mimic the (mostly diamagnetic) cobalt environment in the composite material. The spin-diluted sample consisted of a solid mixture of 99 wt% diamagnetic compounds (**[Pyr][Br]**/*t*-Bu-BQ, 2:1 (w/w)) and 1 wt% **[Pyr]$_2$[Co]**. SWCNT-containing samples were prepared by flushing 1.00 mg of the respective material into a quartz EPR tube (3.00 mm outer diameter) using CH_2Cl_2 (0.25 mL). The freshly prepared spin-diluted, solid sample (see Table S1 for details) was flushed into a 3.00 mm quartz EPR tube with *n*-pentane. The solvent of all samples, except the pure complex **[Pyr]$_2$[Co]** in solution, were then removed *in vacuo* and the residues thoroughly dried.

Table S1: EPR sample compositions and methods of preparation for low *T* measurements.

Sample Description	Composition	Solvent
**[Pyr]$_2$[Co]** (solid)	**[Pyr]$_2$[Co]** (5.00 mg, 5.06 µmol)	-
**[Pyr]$_2$[Co]** (glass)	**[Pyr]$_2$[Co]** (199 µg, 200 nmol)	PhMe/MeOH (3:1), V_{tot} = 400 µL
Spin-diluted sample[a]	**[Pyr]$_2$[Co]** (50.0 µg, 51.0 nmol), **[Pyr][Br]** (4.00 mg, 10.5 µmol), *t*-Bu-BQ (1.00 mg, 4.54 µmol)	-
**SWCNT-[Pyr]$_2$[Co]**[b]	**SWCNT-Co** (~ 5 mg)	-
Purified SWCNTs[c]	SWCNTs (5.00 mg)	-

[a]Sample preparation procedure: A solution containing *t*-Bu-BQ and **[Pyr][Br]** dissolved in MeOH (600 µL) was prepared. **[Pyr]$_2$[Co]** (1.30 mg, 1.32 µmol) was dissolved in MeOH (2.60 mL), and an aliquot (100 µL) of this solution was added the solution containing *t*-Bu-BQ and **[Pyr][Br]**. The mixture was dried *in vacuo*. The yellow residue was suspended in *n*-pentane (1.0 mL) and sonicated for 5 min. [b]Five batches of **SWCNT-[Pyr]$_2$[Co]** were prepared according to the general procedure above and washed with MeOH (3 x 1.5 mL). All obtained dispersions were combined, the solvent was removed *in vacuo* and the residue was re-dispersed in CH_2Cl_2 (0.5 mL). [c]To ensure a comparable manipulation of the SWCNT control sample, the SWCNTs were sonicated in *o*-DCB (4.0 mL) as described in the general procedure above and washed with MeOH (3 x 1.5 mL). The solvent was removed *in vacuo* and the residue was re-dispersed in CH_2Cl_2 (0.5 mL).

XPS Measurements:

Preparation of XPS substrates: *p*-Doped silicon wafers in pieces of ca. 10 x 10 mm were positioned into a PTFE wafer-holder using blunt tweezers. The holder was submerged into a beaker containing *Millipore* water and the wafer was rinsed by gently swiveling the holder. This procedure was repeated with MeOH and acetone, after which the wafers were gently dried in a stream of N_2.

SWCNTs: A dispersion of the SWCNTs (1.00 mg) without **[Pyr]$_2$[Co]** was prepared according to the general procedure (see Section 1.2.7) until the first centrifugation step. A cleaned silicon wafer was placed into a small glass Petri dish and a single drop (ca. 10 µL) of the prepared dispersion was drop-cast onto the substrate *via* a Pasteur pipette. The Petri dish was placed into a vacuum chamber and the solvent was removed *in vacuo*. This procedure was repeated 20 times, after which the substrate was evenly covered in a thin layer of SWCNTs.

SWCNT-[Pyr]$_2$[Co]: The hybrid material was prepared according to the general procedure (see Section 1.2.7), including the purification step. The solid residue was then re-dispersed in *o*-DCB (1.5 mL), the suspension transferred to a scintillation vial and sonicated again for 15 min. A cleaned silicon wafer was placed into a small glass Petri dish and a single drop (ca. 10 µL) of the **SWCNT-[Pyr]$_2$[Co]** dispersion was drop-cast onto the substrate using a Pasteur pipette. The Petri dish was placed into a vacuum chamber and the solvent was removed *in vacuo*. This procedure was repeated 20 times, after which the substrate was evenly covered in a thin layer of **SWCNT-[Pyr]$_2$[Co]**.

Samples of **SWCNT-[CTA]$_2$[Co]** and **SWCNT-[Pyr]$_3$[Co(CN)$_6$]** for XPS analysis were prepared according to the same procedure.

CV Studies:

SWCNT-[Pyr]$_2$[Co] *on GCE*: For all CV studies of the composite material, fresh dispersions according to the general procedure (see Section 1.2.7) were prepared, using SWCNTs (1.00 mg) and **[Pyr]$_2$[Co]** (10.0 mg, 10.1 µmol), but only washing the material with MeOH (1.5 mL) once. The composite was then re-dispersed in *o*-DCB (1.5 mL), transferred to a scintillation vial and sonicated for another 15 min. The glassy carbon electrodes were thoroughly cleaned (see Section 1.1) and dried under an N_2-stream. The **SWCNT-[Pyr]$_2$[Co]** dispersion was then carefully drop-cast on the electrode using a Pasteur pipette (10 µL) and the electrode was dried *in vacuo* for >1 h (Figure S1, left). This procedure was repeated once, ensuring a uniform, thin coverage of the electrode surface (Figure S1, right). The electrode was then immersed into the electrolyte solution and allowed to equilibrate for 10 min, before CV measurements.

SWCNTs on GCE: For control CV studies on the nanotubes, SWCNTs (1.00 mg) were dispersed in *o*-DCB and sonicated as described in Section 1.2.7. The resulting dispersions

were directly used for the drop-casting procedure, without additional purification steps. The glassy carbon electrodes were thoroughly cleaned (see Section 1.1) and dried under an N_2-stream. The SWCNT dispersion was then drop-cast on the electrode using a Pasteur pipette (10 µL) and the electrode was dried *in vacuo* for >1 h. This procedure was repeated once, ensuring a uniform, thin coverage of the electrode surface. The electrode was then immersed into the electrolyte solution for 10 min before CV measurements.

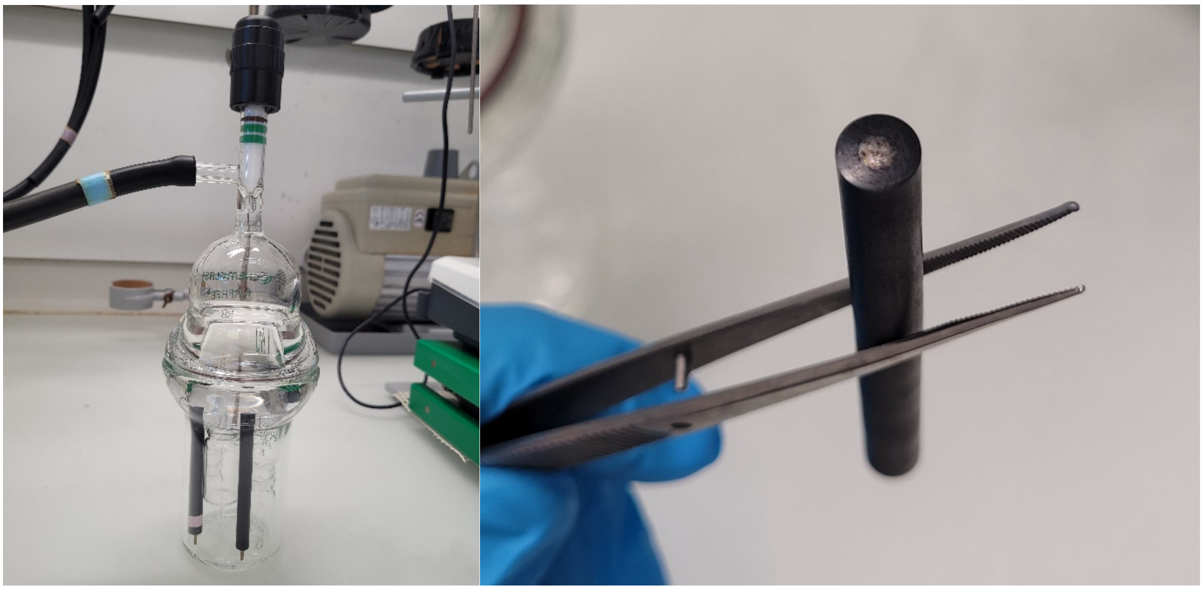

Figure S1: Vacuum chamber for electrode drying (left) and an example of a glassy carbon electrode (GCE) covered with a thin layer of **SWCNT-[Pyr]$_2$[Co]** after two drop-casting steps (right).

Raman Spectroscopy:

Samples for Raman spectroscopy were prepared according to the general procedure described above (see Section 1.2.7), after which an alternative purification method was applied. Here, the crude hybrid material **SWCNT-[Pyr]$_2$[Co]** was suspended in MeOH (1.5 mL) and the suspension was filtered through a nylon filter membrane (0.2 µm). The film was washed once with MeOH and the filter was then dried *in vacuo* for 16 h. SWCNT control samples were prepared according to an identical procedure.

Samples of **SWCNT-[CTA]$_2$[Co]** and **SWCNT-[Pyr]$_3$[Co(CN)$_6$]** were prepared according to the same procedure.

TEM Imaging:

A dispersion of **SWCNT-[Pyr]$_2$[Co]** was prepared according to the general procedure (see Section 1.2.7), stopping before the purification step. A copper TEM grid with a holey carbon support film was placed onto a microscope slide. A single drop of the **SWCNT-[Pyr]$_2$[Co]** dispersion (ca. 10 µL) was drop-cast onto the grid using a Pasteur pipette. The slide was then dried *in vacuo*. The TEM grid was carefully picked up using flat-tip tweezers and swirled in a beaker containing MeOH to wash the composite.

The rest of the dispersion was subjected to the purification steps described in the general procedure (see Section 1.2.7). A TEM sample of the purified composite was then prepared in the same manner as described above.

Control TEM samples containing purified **SWCNT-[Pyr][Br]**, **SWCNT-[CTA]$_2$[Co]** and **SWCNT-[Pyr[$_3$[Co(CN)$_6$]** (see Section 1.2.7) were also prepared as described above.

2 Characterization Methods

2.1 Magnetic Moment Determination

The magnetic moment of complex **[Pyr]$_2$[Co]** was determined using the Evans method in triplicate.[10] For each measurement, a J. Young NMR tube was charged with a weighed amount of **[Pyr]$_2$[Co]**. A weighed amount of dried, degassed DMSO-d_6, was then directly added to the tube. Ferrocene ($FeCp_2$) in DMSO-d_6, contained in a flame-sealed glass capillary, was used as an internal standard. The tube was sealed and shaken until all solids were dissolved. The magnetic moment was determined from the shift difference (Δδ) of the ferrocene Cp-signal in the recorded ^{1}H NMR spectra, measured at 298 K in a temperature-controlled NMR probe, by comparing the sample with a blank DMSO-d_6 reference containing the same ferrocene capillary. The measured molar susceptibilities were corrected for the diamagnetic susceptibility of **[Pyr]$_2$[Co]**.[11] An average of three trials was used to determine the effective magnetic moment of **[Pyr]$_2$[Co]** in Bohr magnetons (μ_B; Table S2).

Table S2: Evans measurements and calculated effective magnetic moment of **[Pyr]$_2$[Co]** in DMSO-d_6.

Trial	Δδ FeCp_2 [ppm]	μ_{eff} [μ_B]
1	0.0284	1.852
2	0.0357	2.016
3	0.0591	1.981
		1.95±0.09

2.2 EPR Spectroscopic Data

2.2.1 **[(*n*-Bu)$_4$N]$_2$[Co]**

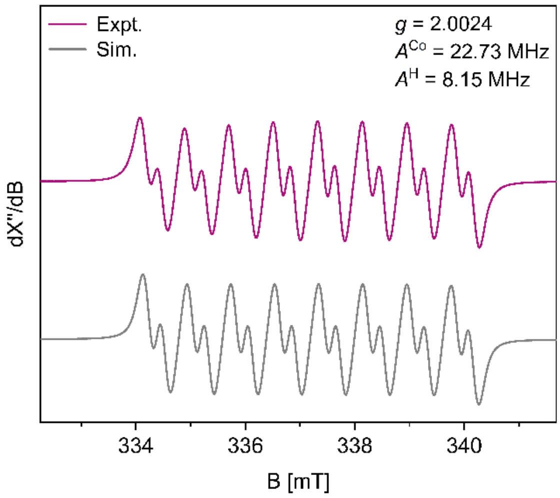

Figure S2: X-Band CW EPR spectrum of **[(*n*-Bu)$_4$N]$_2$[Co]** in PhMe/MeOH (3:1, v/v), recorded at 298 K.

2.2.2 **[CTA]$_2$[Co]**

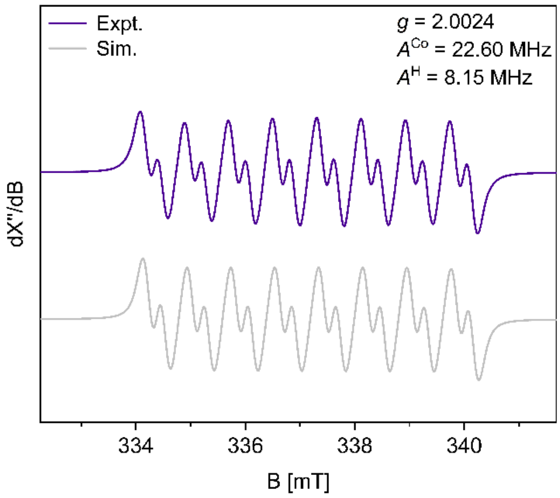

Figure S3: X-Band CW EPR spectrum of **[CTA]$_2$[Co]** in PhMe/MeOH (3:1, v/v), recorded at 298 K.

2.2.3 **[Pyr]₂[Co]**

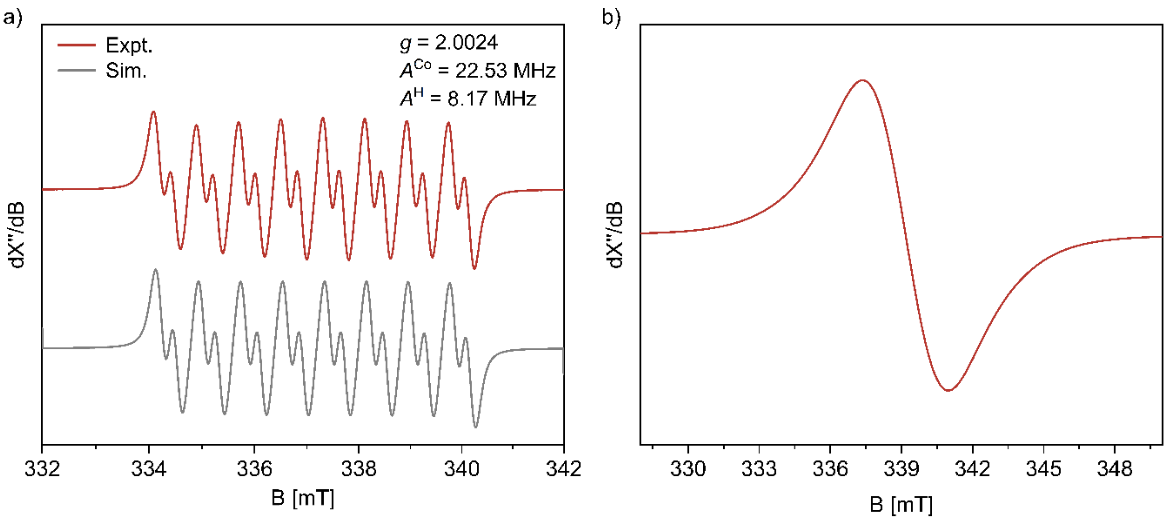

Figure S4: X-Band CW EPR spectra of (a) **$[Pyr]_2[Co]$** in PhMe/MeOH (3:1, v:v) and (b) solid **$[Pyr]_2[Co]$**, both recorded at 298 K.

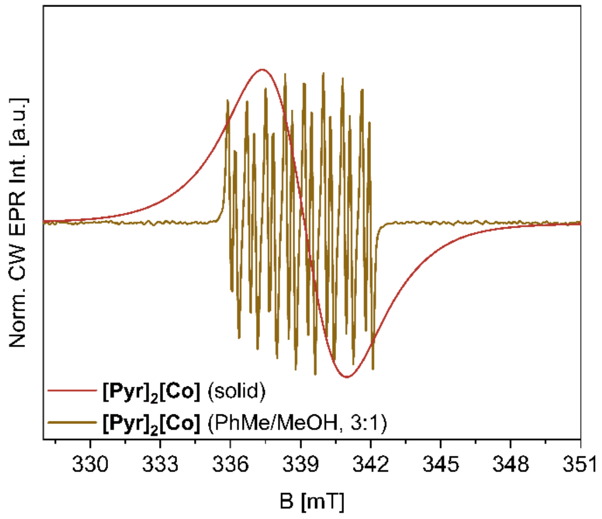

Figure S5: Normalized overlay of X-Band CW EPR spectra of **$[Pyr]_2[Co]$** in the solid state (bronze) and in solution (red), recorded at 298 K.

2.2.4 **SWCNT-$[CTA]_2[Co]$**

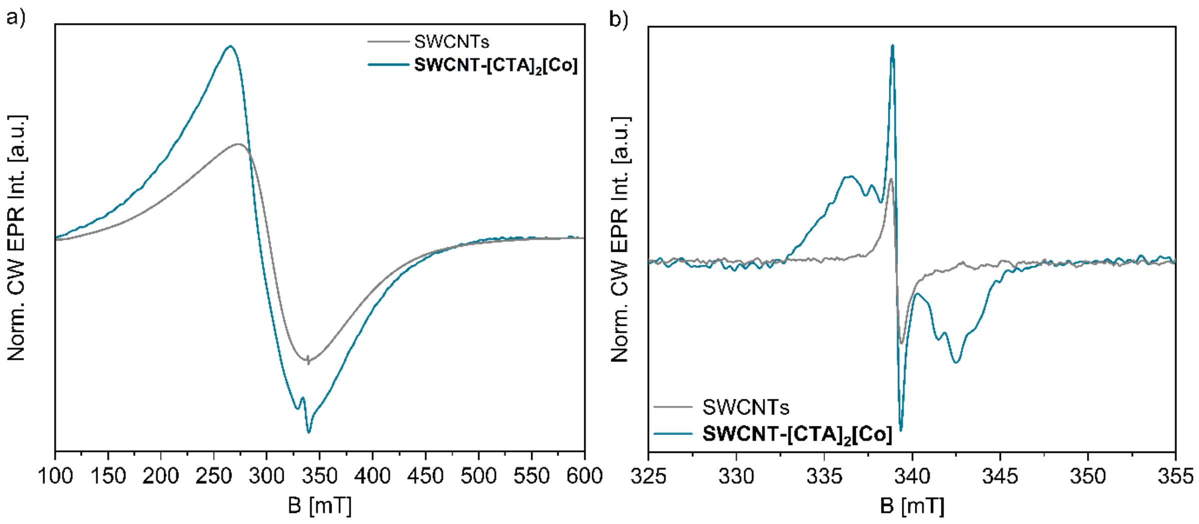

Figure S6: Normalized X-Band CW EPR spectra of SWCNTs (gray) and **SWCNT-$[CTA]_2[Co]$** (petrol), recorded at 298 K, with magnetic field sweeps of (a) 550 mT and (b) 30 mT. In (b), the broad line was removed by subtracting a local third-order (**SWCNT-$[CTA]_2[Co]$**) or sixth-order (SWCNTs) polynomial fit.

2.2.5 **SWCNT-$[Pyr]_2[Co]$**

Room Temperature EPR Data:

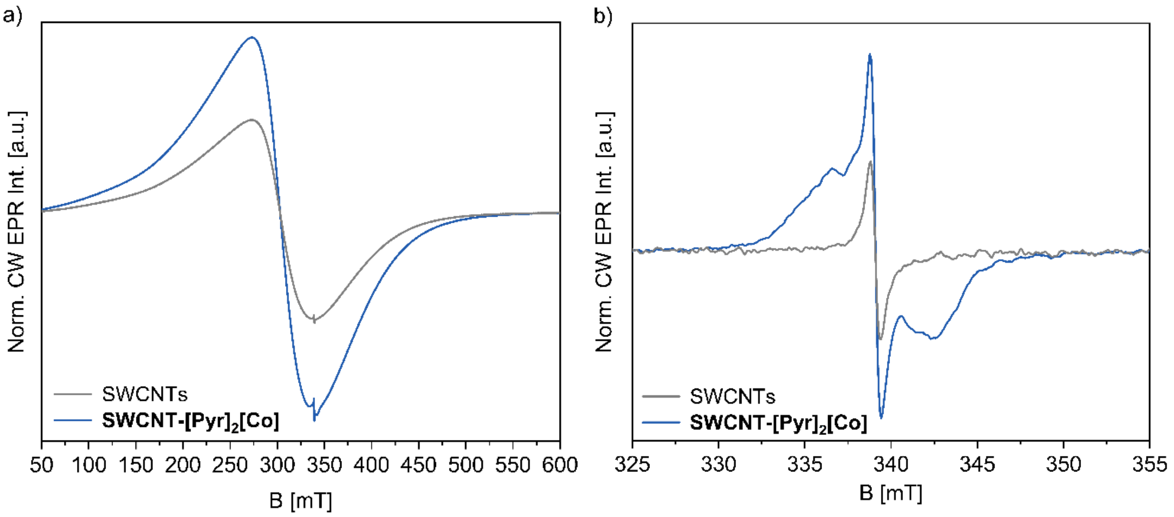

Figure S7: Normalized X-Band CW EPR spectra of SWCNTs (gray) and SWCNT-$[Pyr]_2[Co]$ (blue), recorded at 298 K, with magnetic field sweeps of (a) 550 mT and (b) 30 mT. In (b), the broad line was removed by subtracting a local sixth-order polynomial fit.

Low Temperature (5 K) EPR Data:

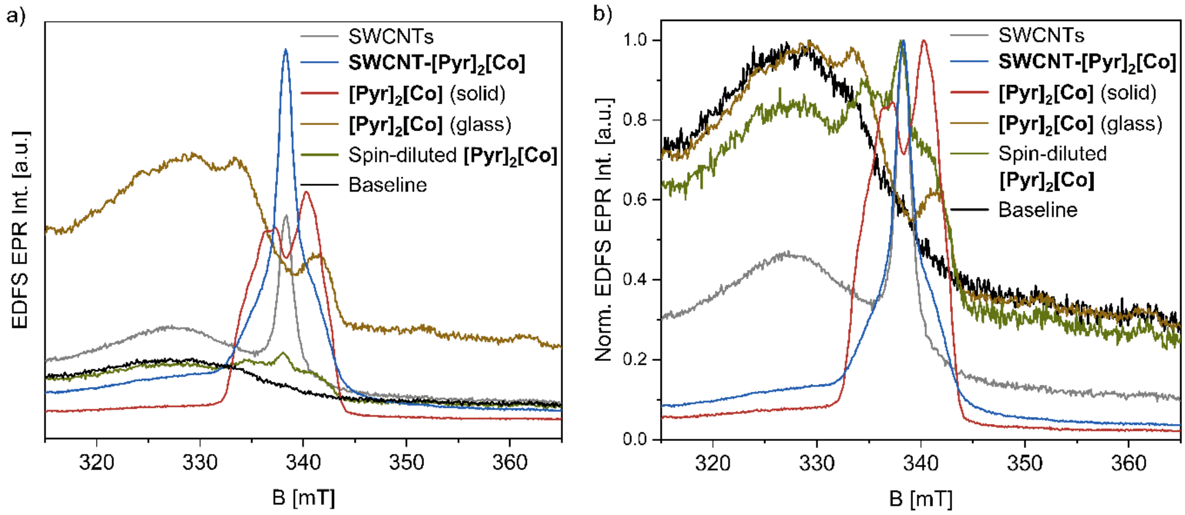

Figure S8: X-Band echo-detected field sweep (EDFS) EPR spectra of SWCNTs (gray), **SWCNT-[Pyr]$_2$[Co]** (blue), solid **[Pyr]$_2$[Co]** (red), glassy **[Pyr]$_2$[Co]** (bronze), spin-diluted **[Pyr]$_2$[Co]** (green) and the intrinsic baseline of the resonator (black; empty 3 mm capillary), collected at 5 K. Spectra are shown at their relative intensities (a) and normalized to an intensity maximum of 1.0 (b).

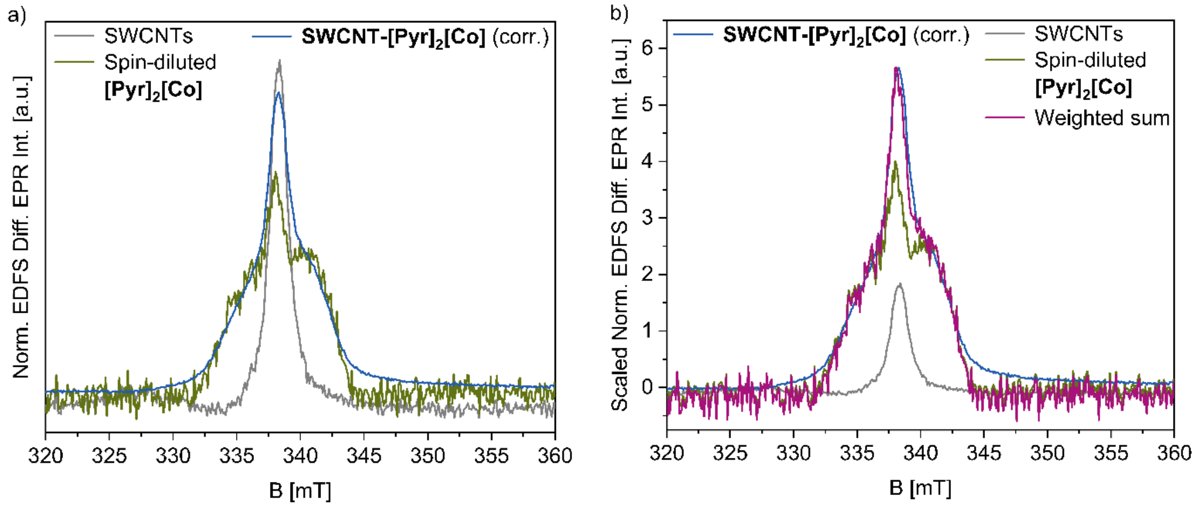

Figure S9: (a) Baseline-corrected EDFS EPR difference spectra (baseline: black trace in Fig. S8) of SWCNTs (gray), **SWCNT-[Pyr]$_2$[Co]** (blue), and spin-diluted **[Pyr]$_2$[Co]** (green), normalized to the spectral intensity at the peripheral magnetic field positions of **SWCNT-[Pyr]$_2$[Co]** (corrected for intrinsic SWCNT signal), collected at 5 K. (b) Baseline-corrected EDFS EPR difference spectra of SWCNTs (gray), **SWCNT-[Pyr]$_2$[Co]** (blue), and spin-diluted **[Pyr]$_2$[Co]** (green), with least-squares fitting of scaling coefficients to produce a weighted sum spectrum (magenta; 77% spin diluted **[Pyr]$_2$[Co]** + 23% SWCNTs) for fitting the spectrum of **SWCNT-[Pyr]$_2$[Co]**.

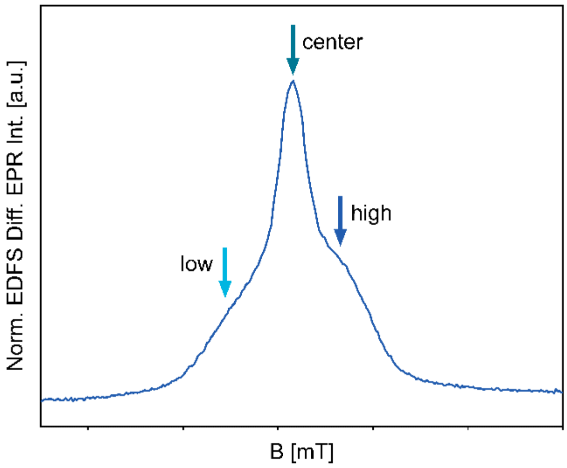

Figure S10: Schematic showing the three magnetic field positions in the EDFS EPR spectrum of **SWCNT-[Pyr]$_2$[Co]** analyzed in the pulsed EPR measurements below. Because the absolute magnetic field values vary between samples due to the slight variations of the resonator center frequency, these positions are denoted as *low* (light-blue), *center* (petrol) and *high* (blue).

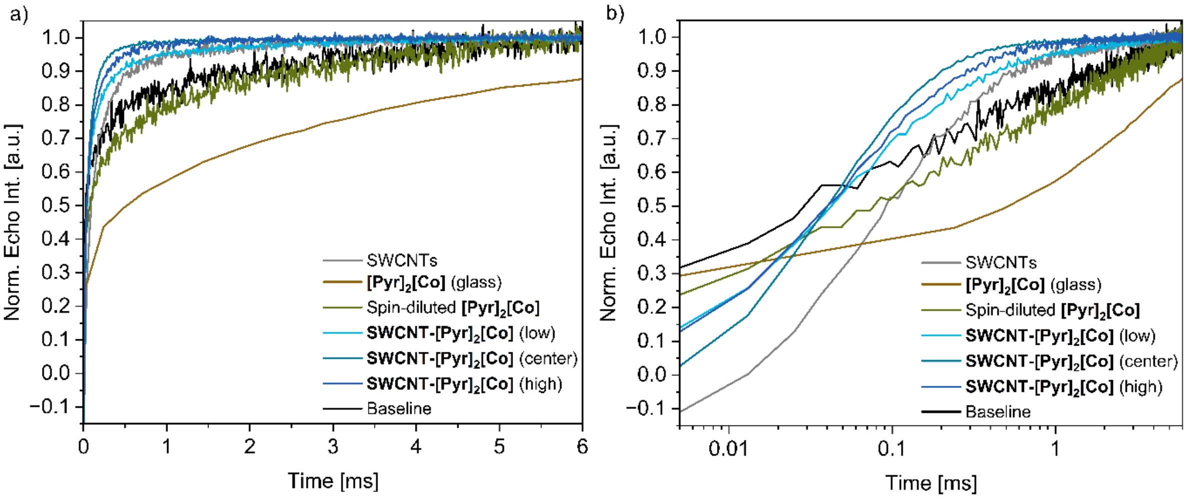

Figure S11: Normalized T_1 relaxation time traces from inversion-recovery EPR experiments for SWCNTs (gray; T_1(1/e) = 0.037 ms), glassy **[Pyr]$_2$[Co]** (bronze; T_1(1/e) = 0.721 ms), spin-diluted **[Pyr]$_2$[Co]** (green, T_1(1/e) = 0.049 ms) and **SWCNT-[Pyr]$_2$[Co]** (T_1(1/e) = 0.025 ms), measured at low (light-blue), center (petrol) and high (blue) magnetic field positions. Data are shown on (a) a linear time scale and (b) a logarithmic time scale, collected at 5 K.

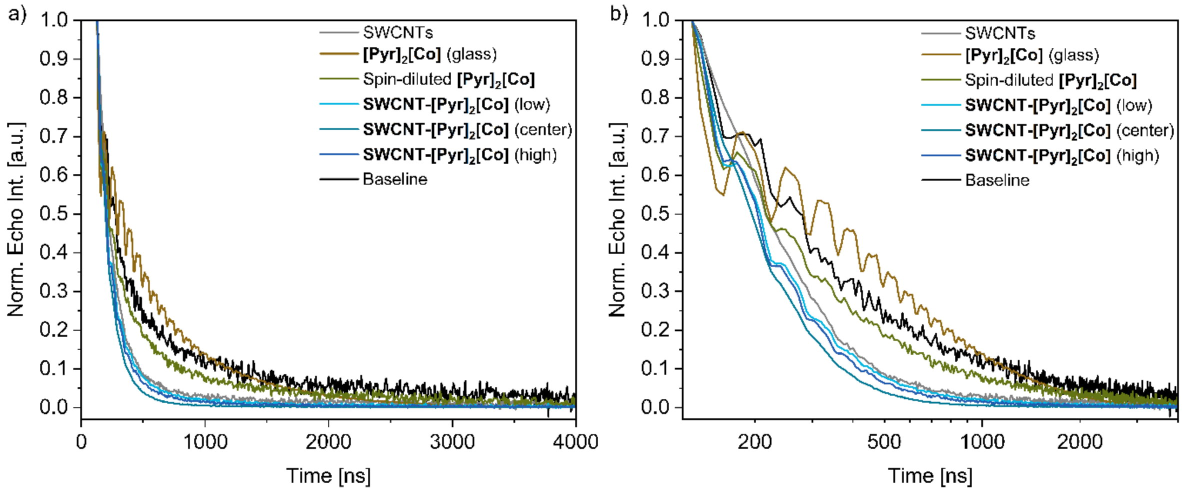

Figure S12: Normalized two-pulse electron spin echo envelope modulation (2pESEEM) time traces for SWCNTs (gray; T_2(1/e) = 264 ns), glassy **[Pyr]₂[Co]** (bronze; T_2(1/e) = 448 ns), spin-diluted **[Pyr]₂[Co]** (green, T_2(1/e) = 288 ns) and **SWCNT-[Pyr]₂[Co]** measured at low (light-blue; T_2(1/e) = 224 ns), center (petrol; T_2(1/e) = 224 ns) and high (blue; T_2(1/e) = 248 ns) magnetic field positions. Data are shown on (a) a linear time scale and (b) a logarithmic time scale, collected at 5 K.

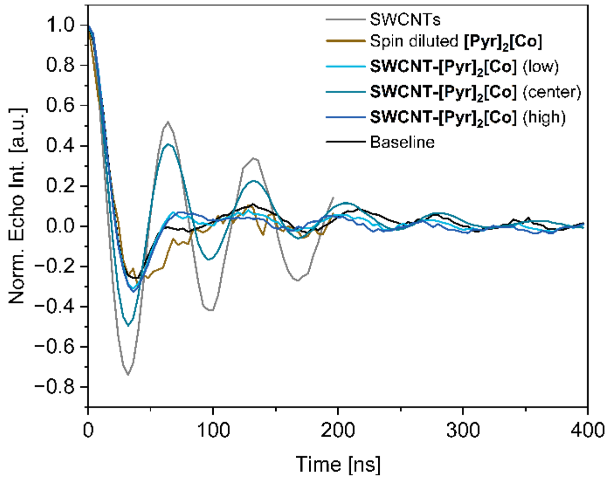

Figure S13: EPR-detected Rabi oscillations for SWCNTs (gray), glassy **[Pyr]₂[Co]** (bronze), spin-diluted **[Pyr]₂[Co]** (green) and **SWCNT-[Pyr]₂[Co]** measured at low (light-blue), center (petrol) and high (blue) magnetic field positions, collected at 5 K.

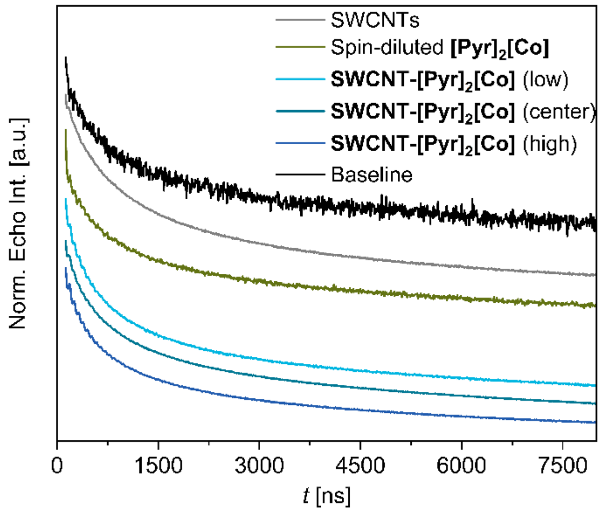

Figure S14: Three-pulse electron spin echo envelope modulation (3pESEEM) time traces for SWCNTs (gray), glassy **[Pyr]$_2$[Co]** (bronze), spin-diluted **[Pyr]$_2$[Co]** (green) and **SWCNT-[Pyr]$_2$[Co]** at low (light-blue), center (petrol) and high (blue) magnetic field positions, with τ°= 144 ns, collected at 5 K.

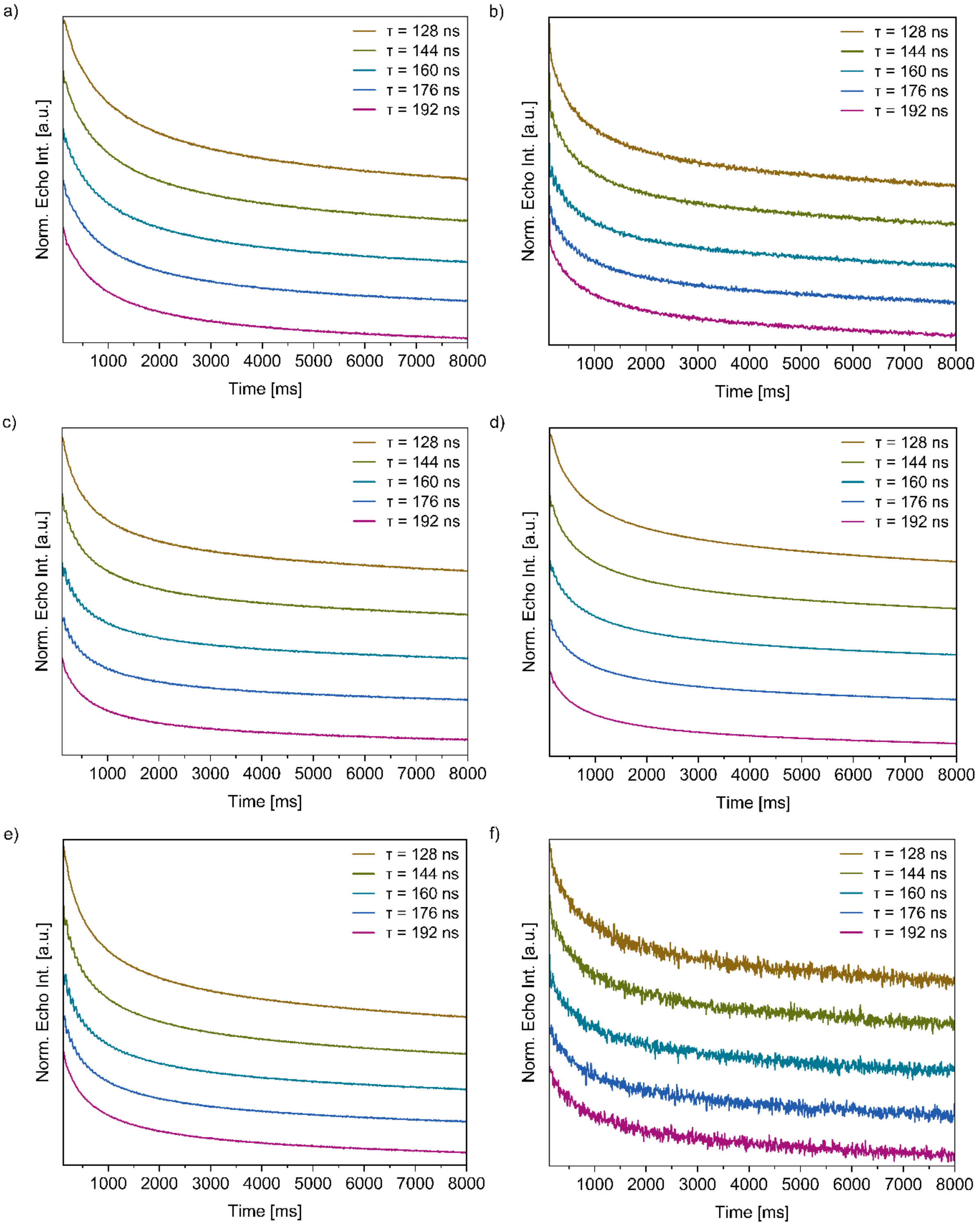

Figure S15: Three-pulse electron spin echo envelope modulation (3pESEEM) time traces for (a) SWCNTs, (b) spin-diluted **[Pyr]$_2$[Co]**, (c–e) **SWCNT-[Pyr]$_2$[Co]** at low, center, and high magnetic field positions, respectively, and (f) the resonator baseline signal. Data were collected at 5 K with pulse delay times of τ°= 128 ns (bronze), 144 ns (green), 160 ns (petrol), 176 ns (blue), and 192 ns (magenta).

2.3 XPS Data

2.3.1 **[Pyr]₂[Co]**

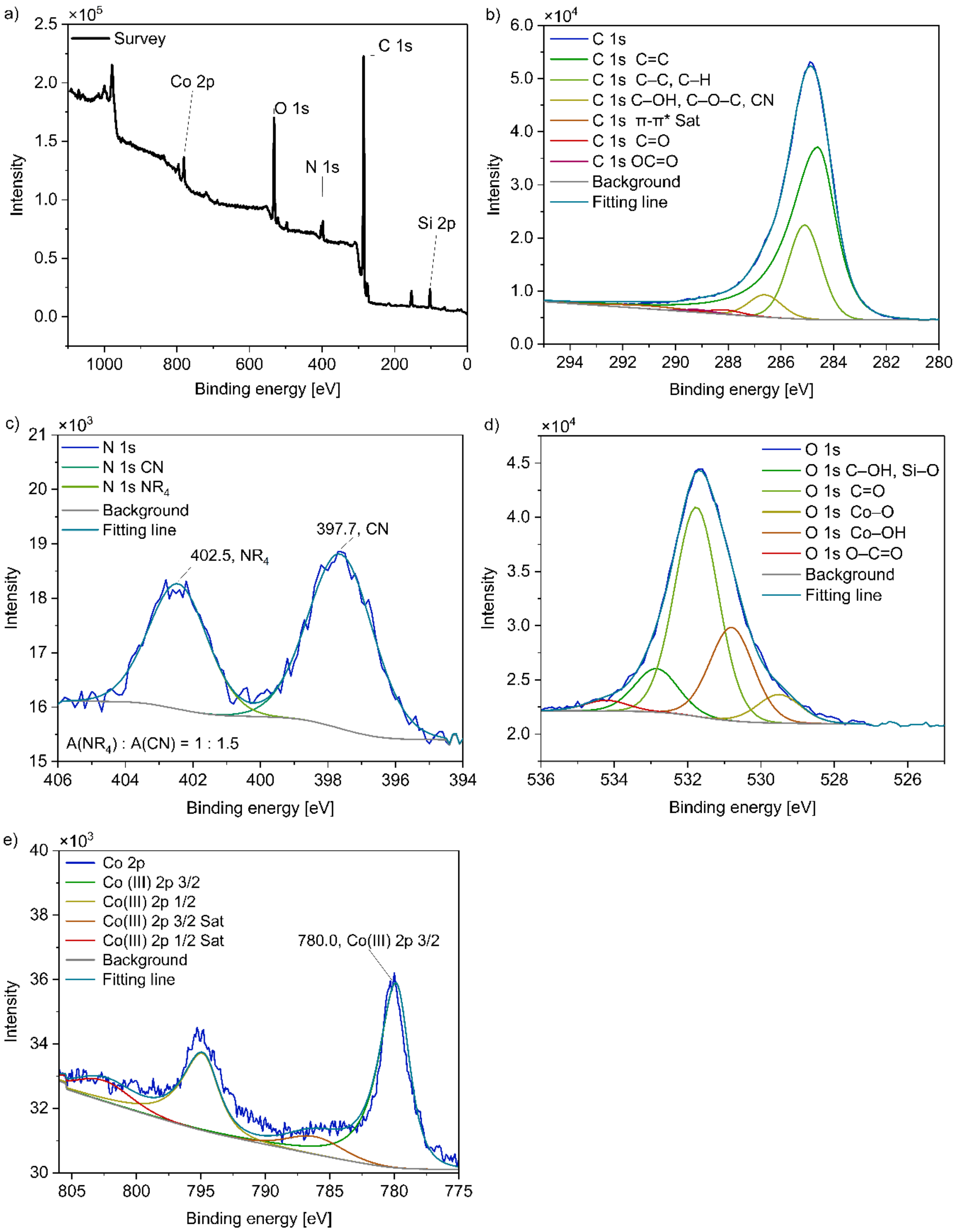

Figure S16: (a) Survey scan and high-resolution XPS spectra of **[Pyr]₂[Co]**: (b) C 1s, (c) N 1s, (d) O 1s, and (e) Co 2p regions.

2.3.2 SWCNTs

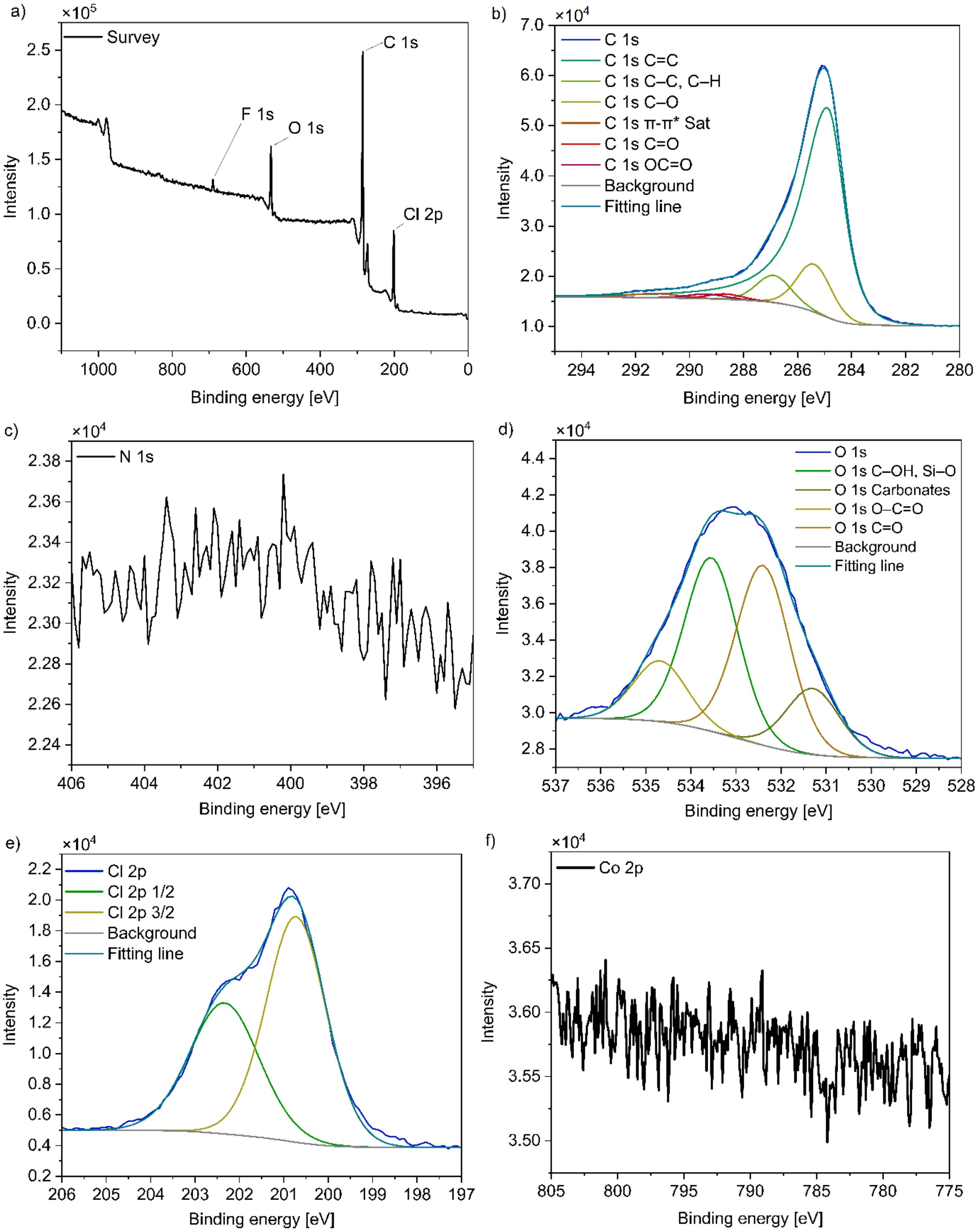

Figure S17: (a) Survey scan and high-resolution XPS spectra of pure SWCNTs: (b) C 1s, (c) N 1s, (d) O 1s, (e) Cl 2p, and (f) Co 2p regions. The more intense Cl 2p signal in the survey scan, relative to functionalized SWCNTs, is attributed to residual *o*-DCB on the substrate. *o*-DCB is the solvent used to disperse SWCNTs, which was removed during purification of **SWCNT-[Pyr]$_2$[Co]**.

2.3.3 SWCNT-[Pyr]₂[Co]

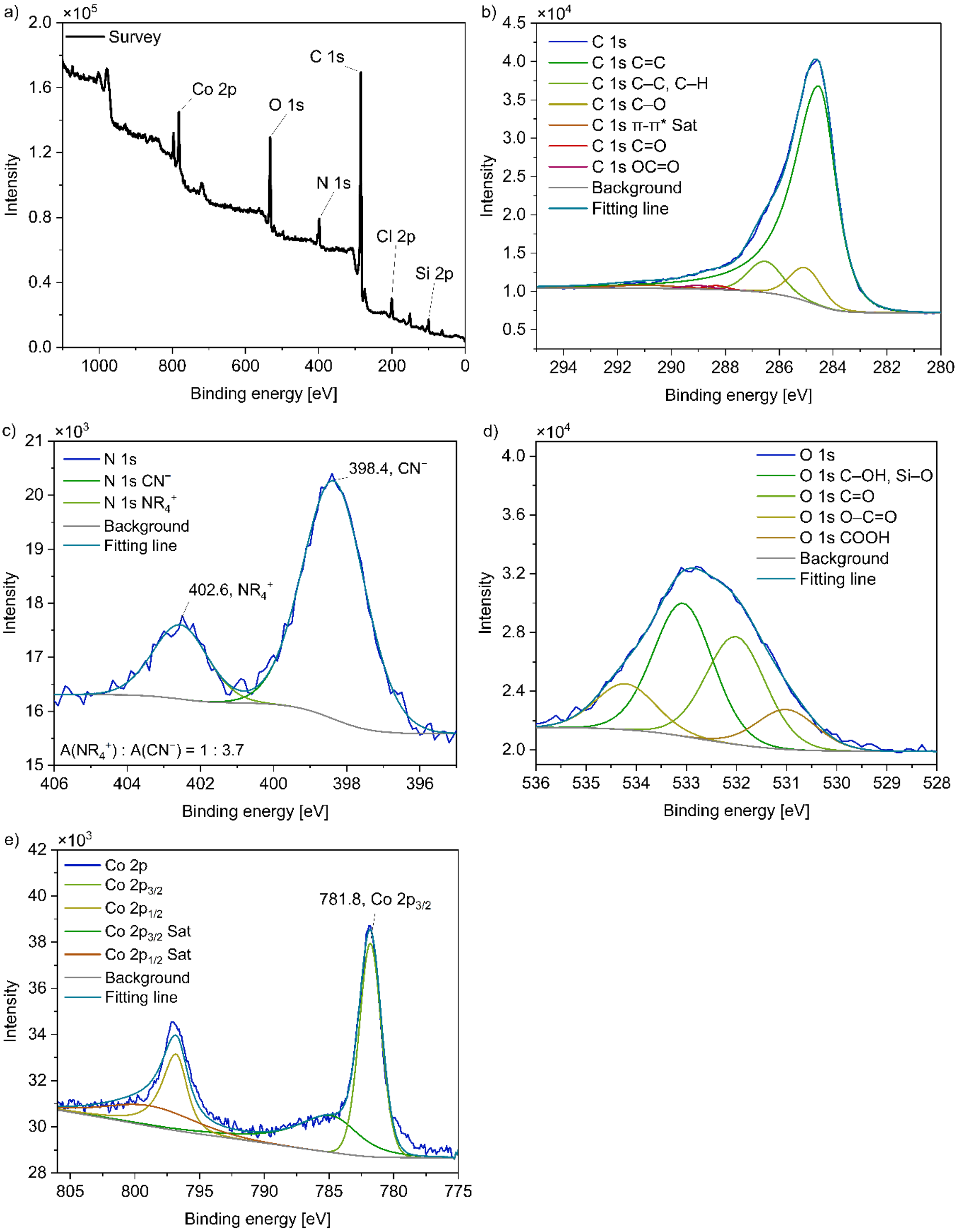

Figure S18: (a) Survey scan and high-resolution XPS spectra of **SWCNT-[Pyr]₂[Co]**: (b) C 1s, (c) N 1s, (d) O 1s, and (e) Co 2p regions.

2.3.4 **SWCNT-$[CTA]_2[Co]$**

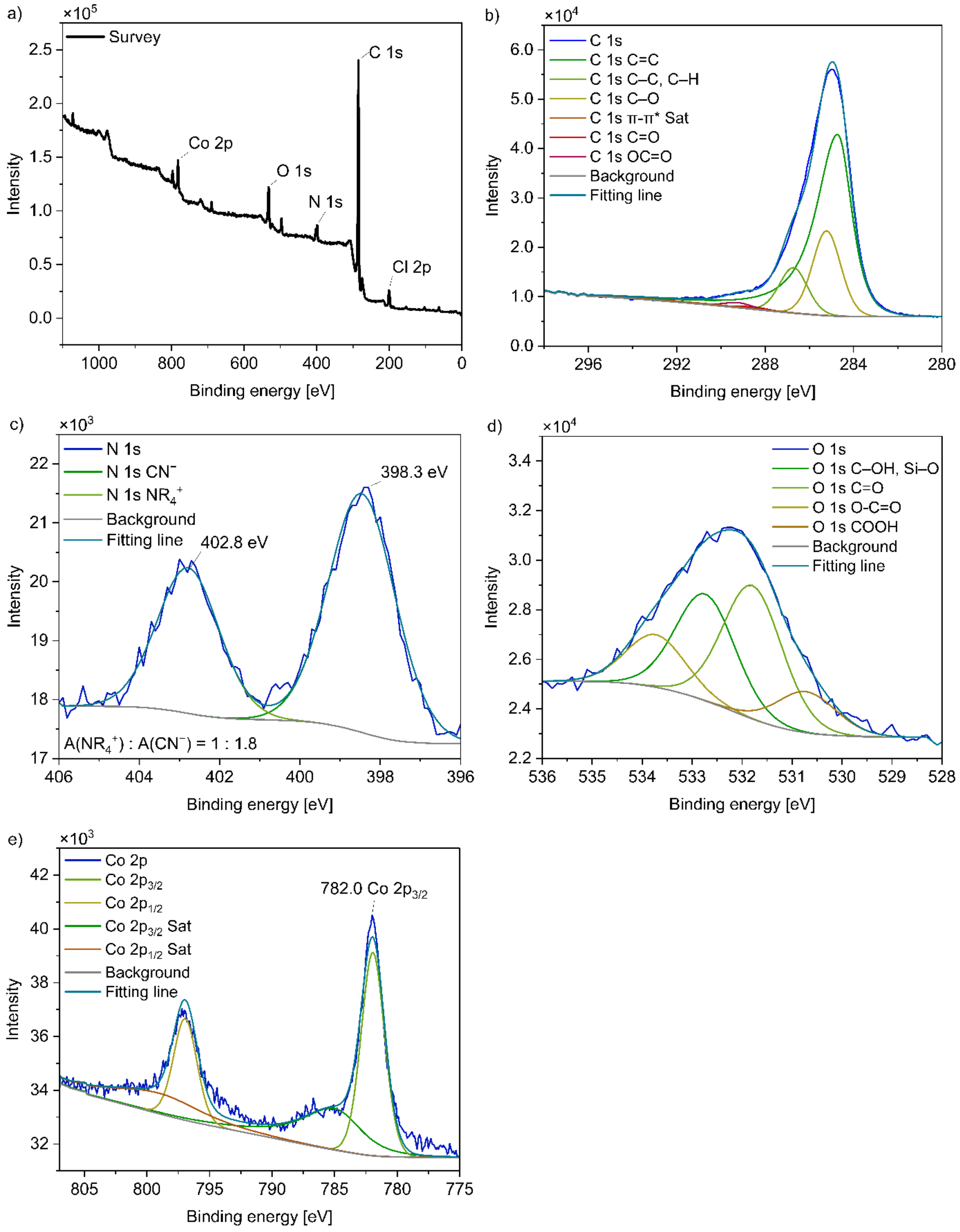

Figure S19: (a) Survey scan and high-resolution XPS spectra of **SWCNT-$[CTA]_2[Co]$**: (b) C 1s, (c) N 1s, (d) O 1s, and (e) Co 2p regions.

2.3.5 SWCNT-$[Pyr]_3[Co(CN)_6]$

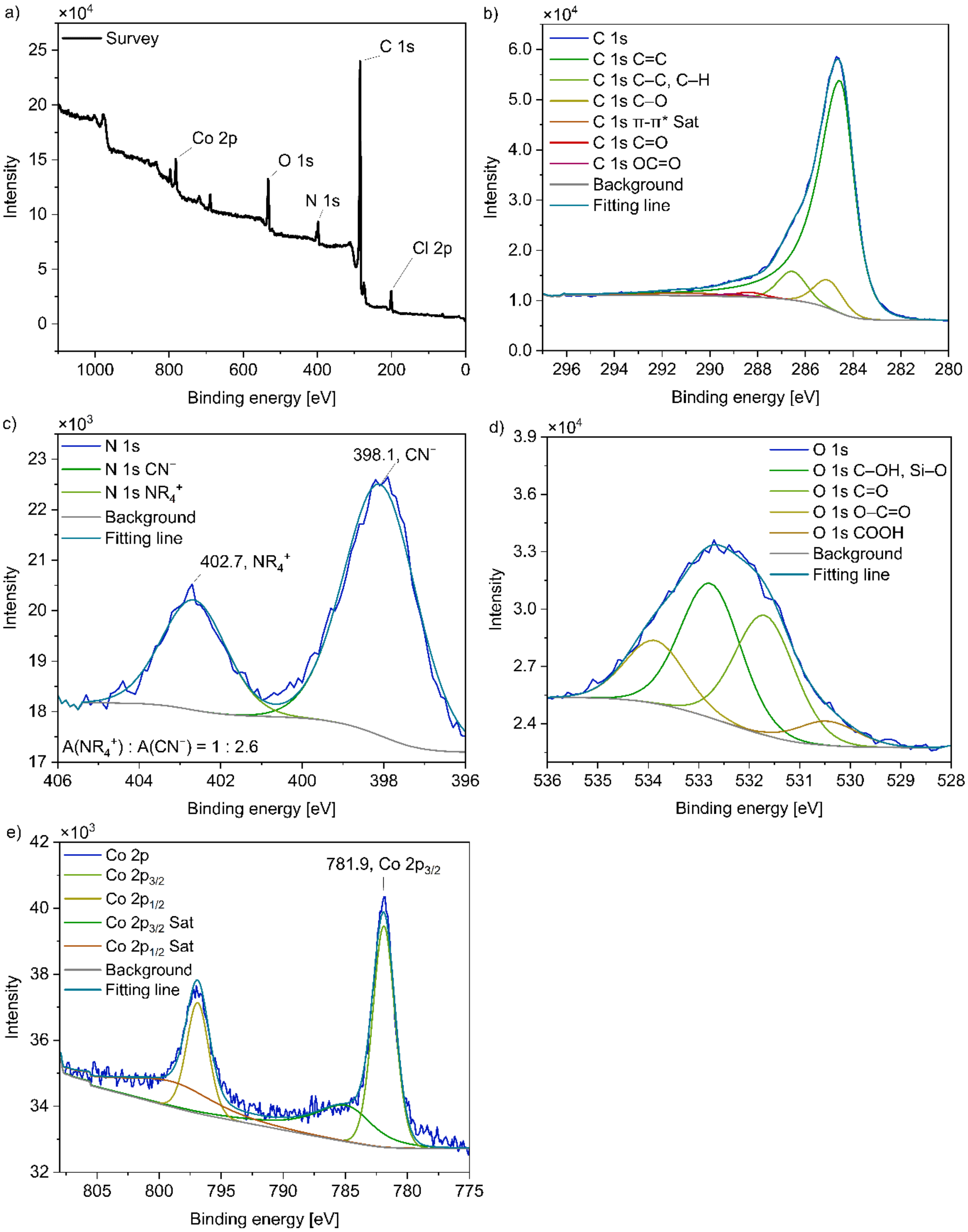

Figure S20: (a) Survey scan and high-resolution XPS spectra of **SWCNT-$[Pyr]_3[Co(CN)_6]$**: (b) C 1s, (c) N 1s, (d) O 1s, and (e) Co 2p regions.

Note: Comparison of the N 1s XPS spectra for **$[Pyr]_2[Co]$** and **SWCNT-$[Pyr]_2[Co]$** shows an increase in the NR_4^+:CN^- peak area ratio from 1:1.5 to 1:3.7, deviating from the expected 1:2 stoichiometry. This discrepancy may result from (i) X-ray- or neutraliser-induced decomposition of the quaternary ammonium cations, reducing their signal intensity,[12–14] and/or (ii) differences in depth distribution, with NR_4^+ groups more deeply embedded in the SWCNT matrix and thus underrepresented in the surface-sensitive XPS measurement.[15] Both effects would lead to a shift of the NR_4^+:CN^- area ratio in favor of CN^-. A similar deviation was found for **SWCNT-$[Pyr]_3[Co(CN)_6]$** (1:2.6 vs stoichiometrically expected 1:2 NR_4^+:CN^- area ratio).

To estimate the atomic ratio between C and Co of **SWCNT-$[Pyr]_2[Co]$**, an external calibration using the pure **$[Pyr]_2[Co]$** complex was carried out.[16] By extracting the sensitivity factor for the complex from the experimentally found XPS atomic ratio vs the theoretical one, and applying it to the composite material, a weight percentage of 4.2% Co was found for **SWCNT-$[Pyr]_2[Co]$**.

2.4 Electrochemical Data

2.4.1 **[(*n*-Bu)$_4$N]$_2$[Co]** in 1,2-DFB

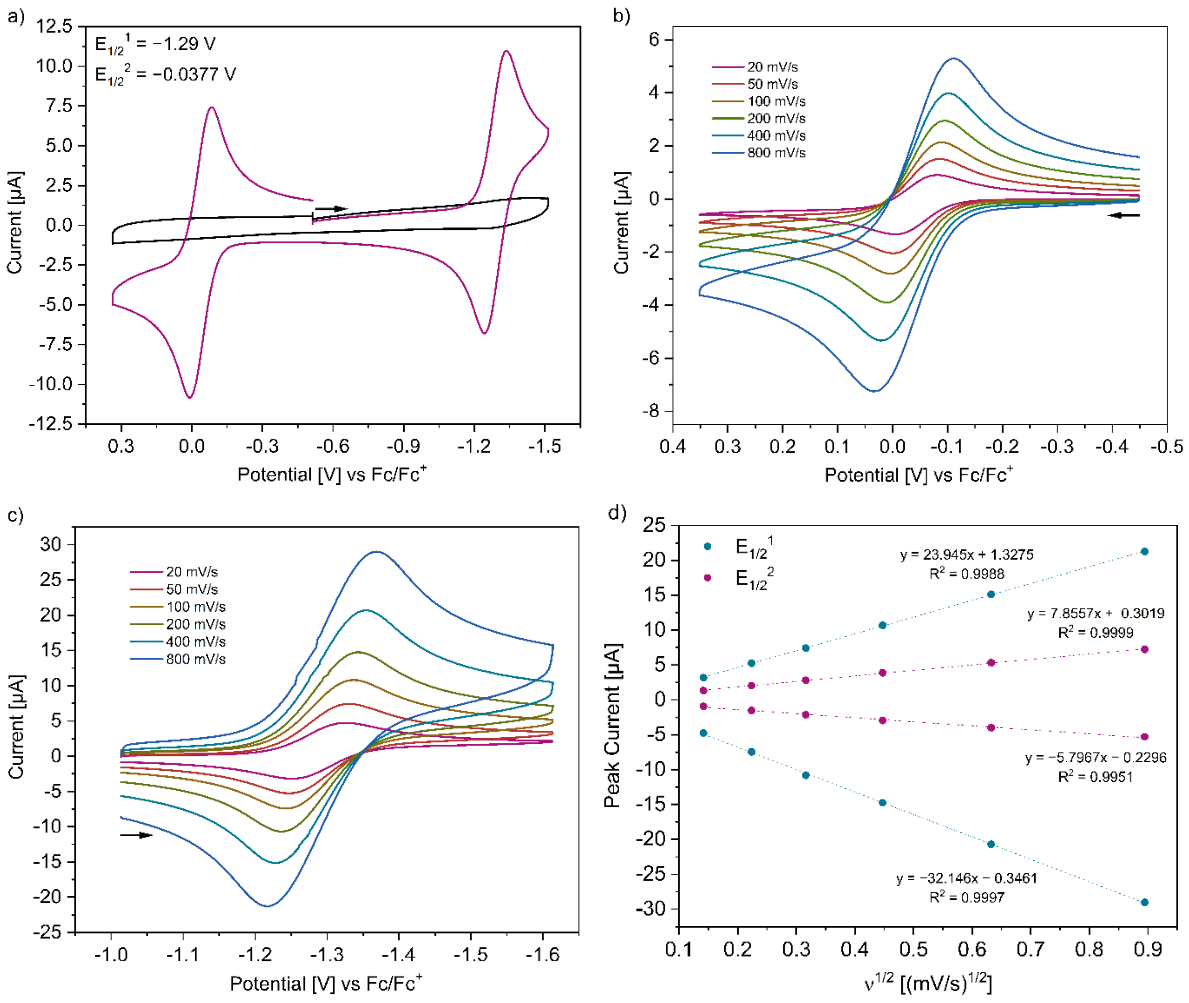

Figure S21: (a) Cyclic voltammogram and (b–d) scan rate study of **[(*n*-Bu)$_4$N]$_2$[Co]** (1.0 mM) with [(*n*-Bu)$_4$N][PF$_6$] (0.10 M) as supporting electrolyte in 1,2-DFB, referenced to Fc/Fc$^+$. The full scan (magenta) was recorded at 100 mV/s and is shown alongside the background (black) of the supporting electrolyte.

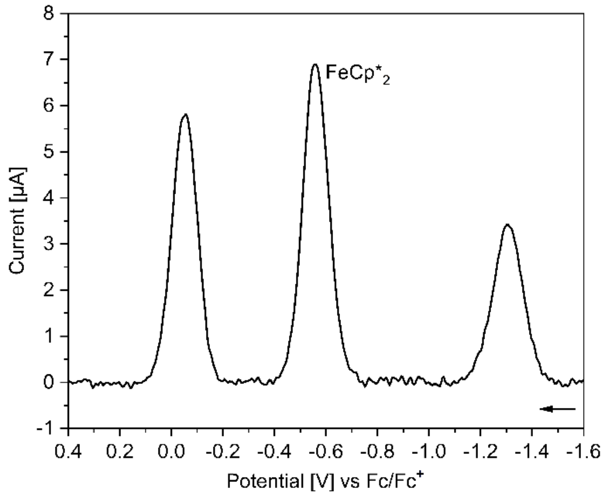

Figure S22: Square-wave voltammogram of **[(*n*-Bu)$_4$N]$_2$[Co]** (1.0 mM) in 1,2-DFB in the presence of decamethylferrocene (FeCp*$_2$, 1.0 mM).

Note: Peak areas were referenced to FeCp*$_2$ to confirm single-electron transfer for both redox couples. The anodic feature shows the expected 1:1 ratio to FeCp*$_2$, consistent with its reversible character determined by CV. By contrast, the cathodic feature displays a smaller relative peak area, attributed to quasi-reversible behavior.

2.4.2 **[Pyr]$_2$[Co]** in DME

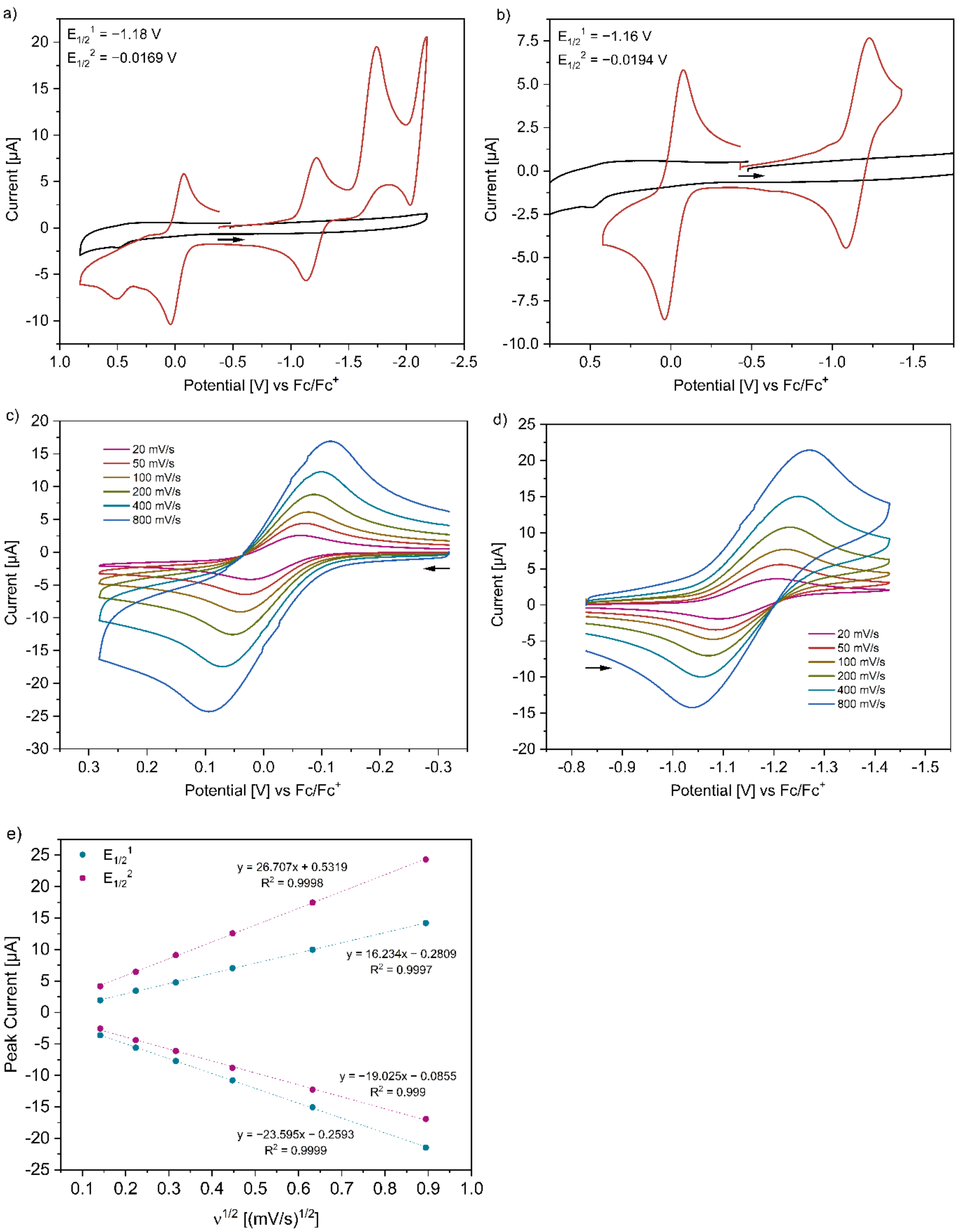

Figure S23: (a,b) Cyclic voltammograms and (c–e) scan rate study of **[Pyr]$_2$[Co]** (1.0 mM) with [(n-Bu)$_4$N][PF$_6$] (0.10 M) as supporting electrolyte in DME, referenced to Fc/Fc$^+$. The full scans (red) in (a) and (b) were recorded at 100 mV/s and are shown alongside the background trace (black) of the supporting electrolyte.

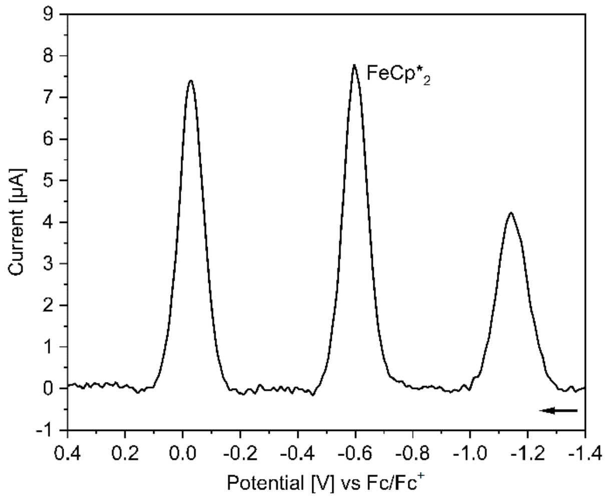

Figure S24: Square-wave voltammogram of **[Pyr]$_2$[Co]** (1.0 mM) in MeCN in the presence of decamethylferrocene (FeCp*$_2$, 1.0 mM).

Note: Peak areas were referenced to FeCp*$_2$ to confirm single-electron transfer for both redox couples. The anodic feature shows the expected 1:1 ratio to FeCp*$_2$, consistent with its reversible character determined by CV. By contrast, the cathodic feature displays a smaller relative peak area, attributed to quasi-reversible behavior.

2.4.3 **[Pyr]$_3$[Co(CN)$_6$]** in 1,2-DFB

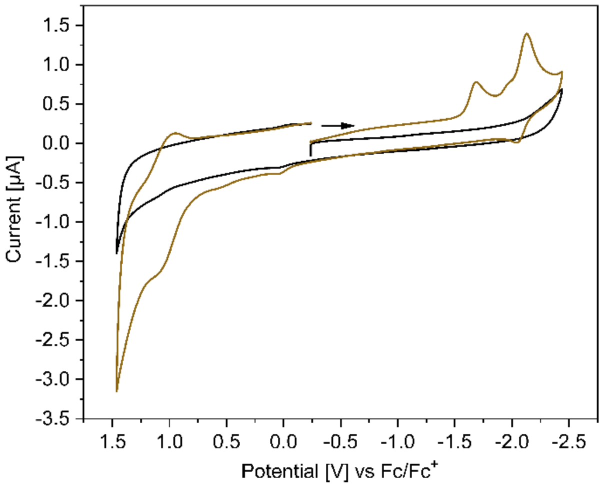

Figure S25: Cyclic voltammogram of **[Pyr]$_3$[Co(CN)$_6$]** (1.0 mM) with [(*n*-Bu)$_4$N][PF$_6$] (0.10 M) as supporting electrolyte in 1,2-DFB, referenced to Fc/Fc$^+$. The full scan (bronze) was recorded at 100 mV/s and is shown alongside the background (black) of the supporting electrolyte.

2.4.4 SWCNTs

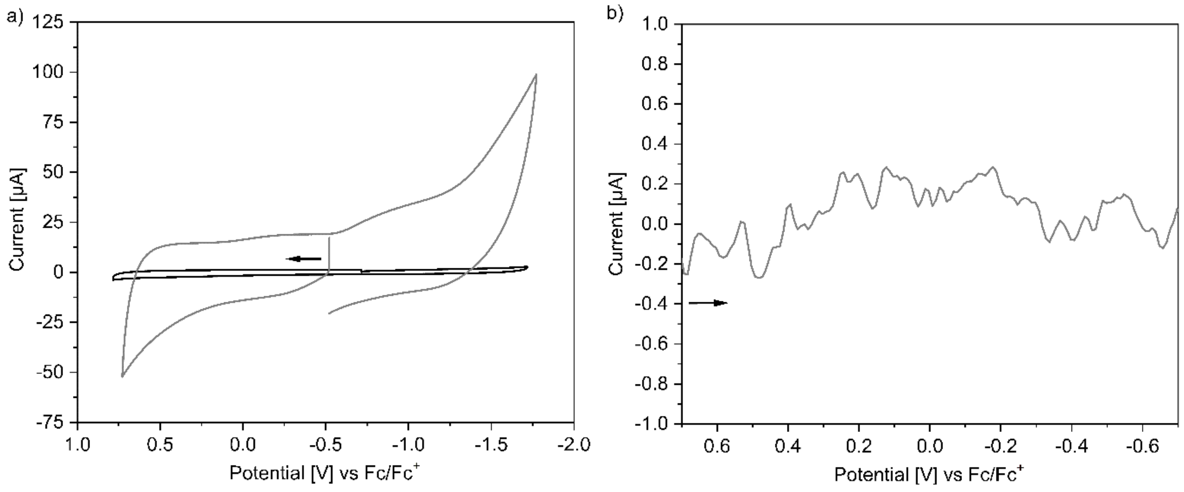

Figure S26: (a) Cyclic voltammogram and (b) square-wave voltammogram of SWCNTs drop-cast onto a glassy carbon working electrode, with [(*n*-Bu)$_4$N][PF$_6$] (0.10 M) as supporting electrolyte in 1,2-DFB, referenced to Fc/Fc$^+$. The full scan (gray) in (a) was recorded at 100 mV/s and is shown alongside the background trace (black) of the supporting electrolyte.

2.4.5 SWCNT-[Pyr]₂[Co]

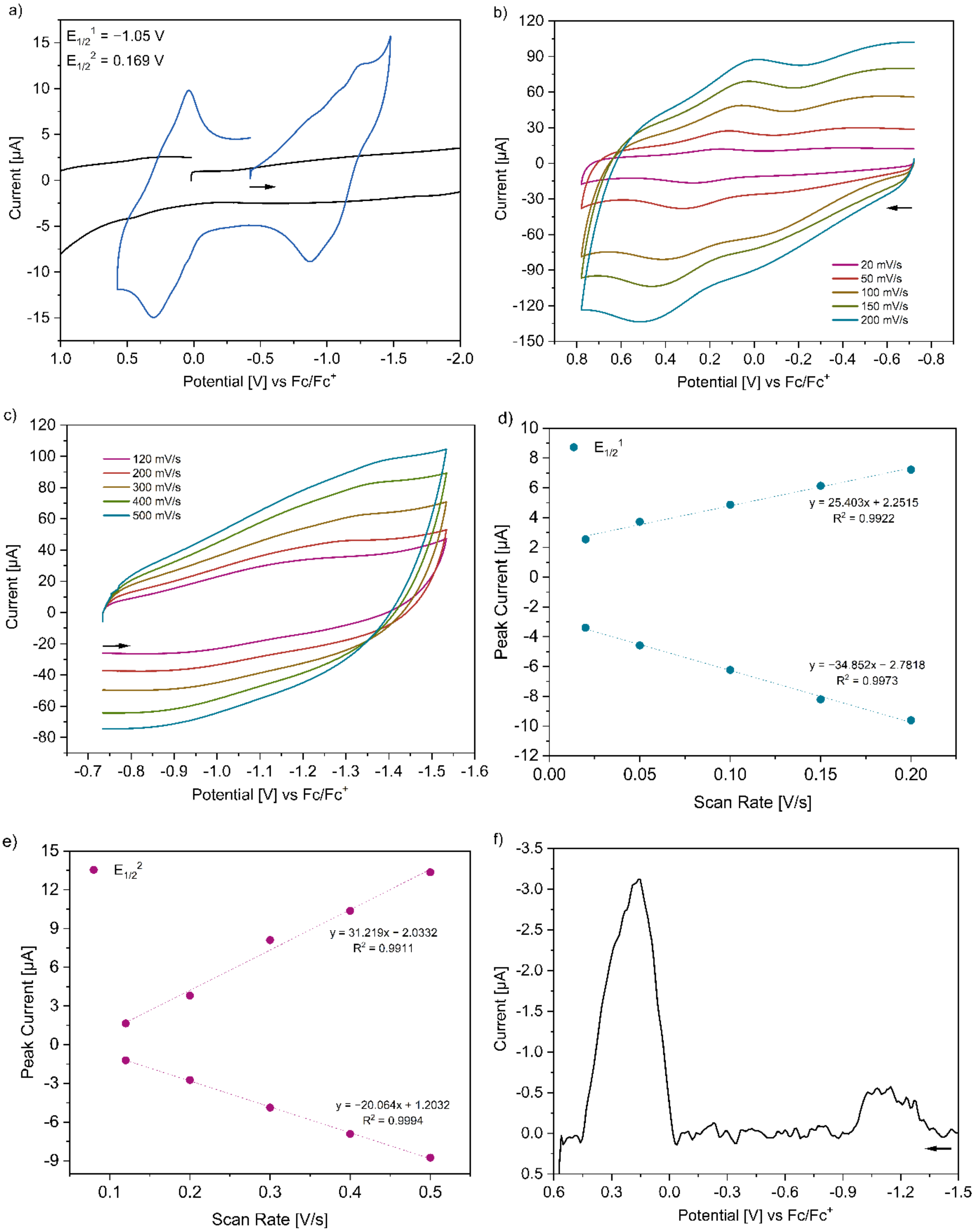

Figure S27: (a) Cyclic voltammogram, (b–e) scan rate study, and (f) square wave voltammogram of **SWCNT-[Pyr]₂[Co]** drop-cast onto a glassy carbon working electrode, with $[(n\text{-Bu})_4N][PF_6]$ (0.10 M) as supporting electrolyte in 1,2-DFB, referenced to Fc/Fc^+. The full scan (blue) in (a) was recorded at 100 mV/s and is shown alongside the background trace (black) of the supporting electrolyte. The current minima of the reduction event were

determined by identifying the onset potential as the inflection point of the cathodic current, using *EC-Lab V11.52*.

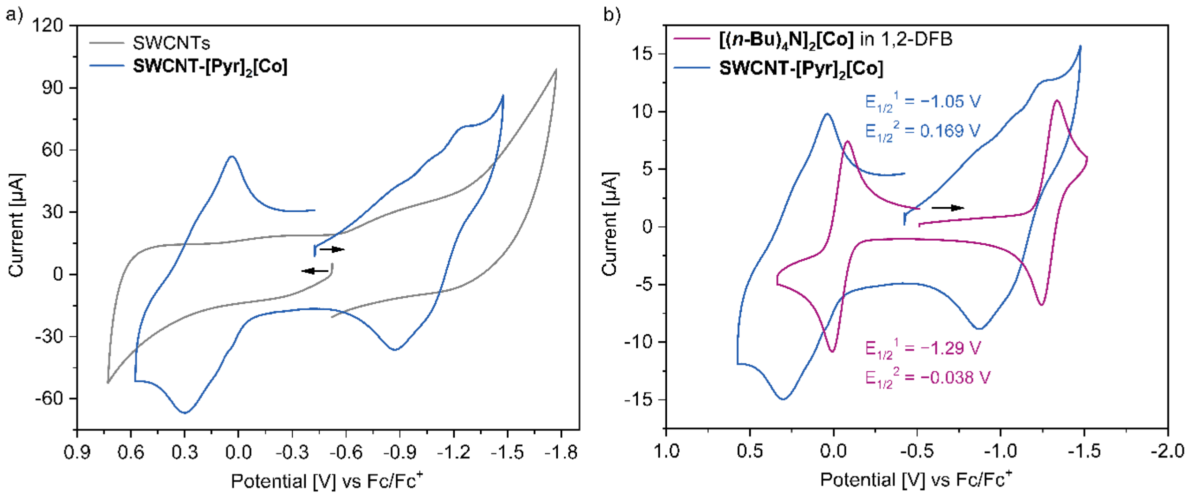

Figure S28: Comparison of the cyclic voltammogram of **SWCNT-[Pyr]$_2$[Co]** with (a) pure SWCNTs and (b) **[Pyr]$_2$[Co]** in 1,2-DFB.

2.4.6 SWCNT-$[Pyr]_3[Co(CN)_6]$

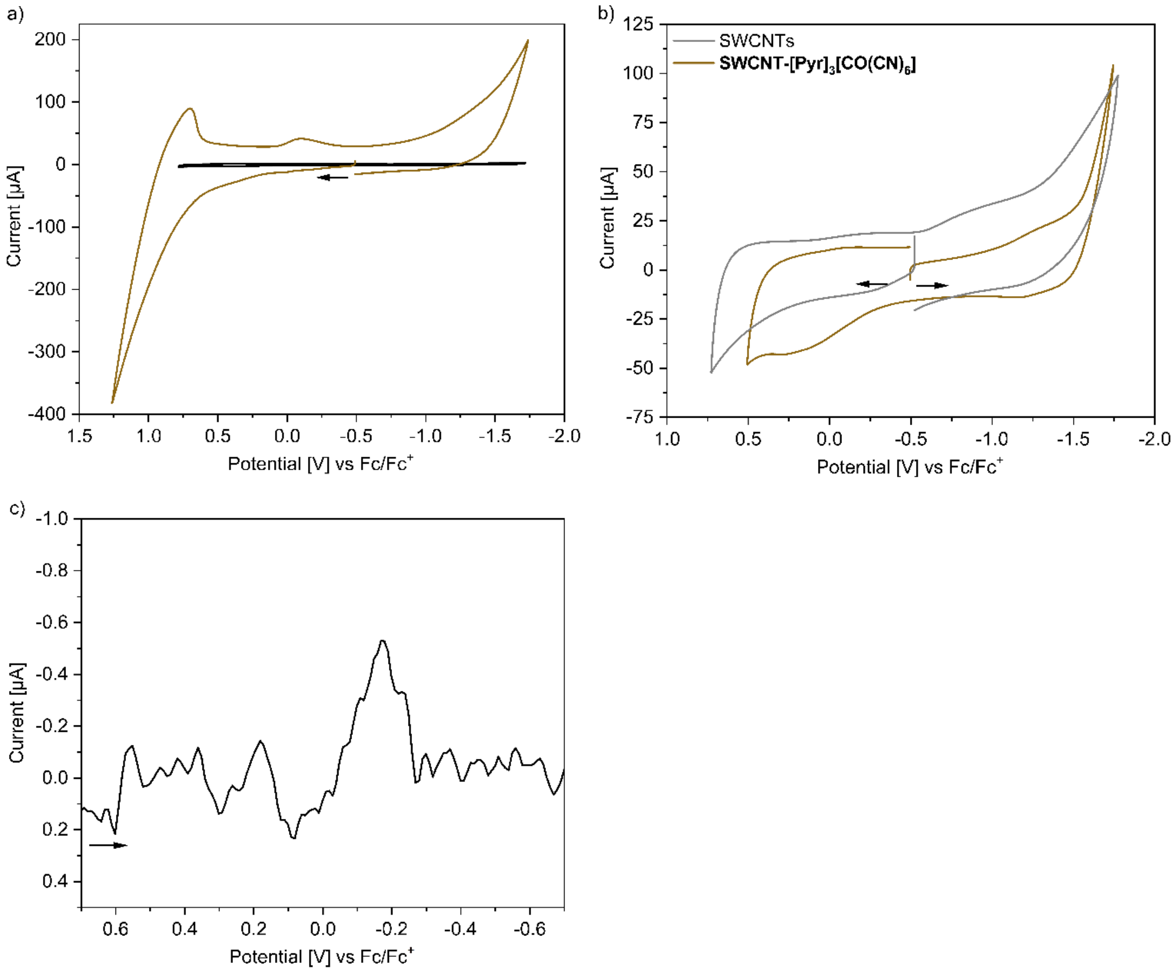

Figure S29: (a) Cyclic voltammogram of **SWCNT-$[Pyr]_3[Co(CN)_6]$** drop-cast onto a glassy carbon working electrode, with $[(n\text{-Bu})_4N][PF_6]$ (0.10 M) as supporting electrolyte in 1,2-DFB, referenced to Fc/Fc^+. (b) Comparison of the cyclic voltammograms of **SWCNT-$[Pyr]_3[Co(CN)_6]$** (bronze) with pure SWCNTs (grey). (c) Square wave voltammogram of **SWCNT-$[Pyr]_3[Co(CN)_6]$**. The full scan (bronze) in (a) was recorded at 100 mV/s and is shown alongside the background trace (black) of the supporting electrolyte.

2.5 Raman Spectroscopic Data

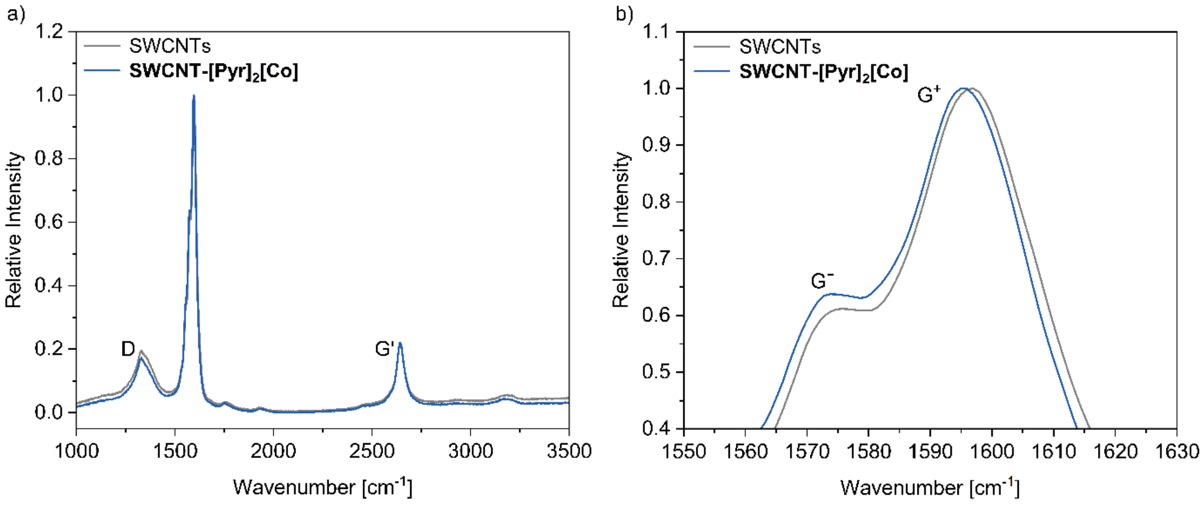

Figure S30: Raman spectra of SWCNTs (gray) and **SWCNT-[Pyr]$_2$[Co]** (blue) over (a) the full scan window and (b) the G-band region, normalized to the G-band maximum.

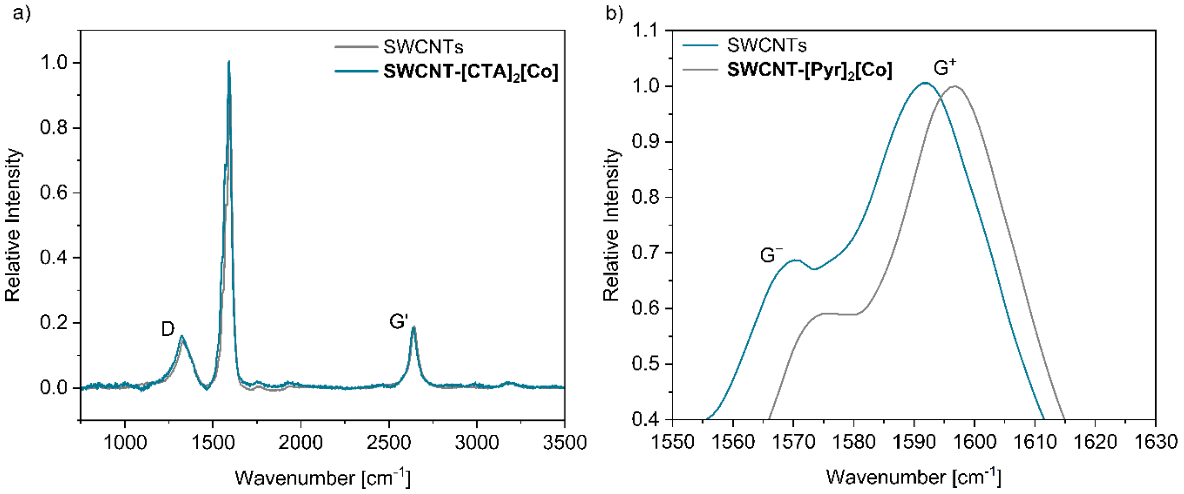

Figure S31: Raman spectra of SWCNTs (gray) and **SWCNT-[CTA]$_2$[Co]** (petrol) over (a) the full scan window and (b) the G-band region, both normalized to the G-band maximum.

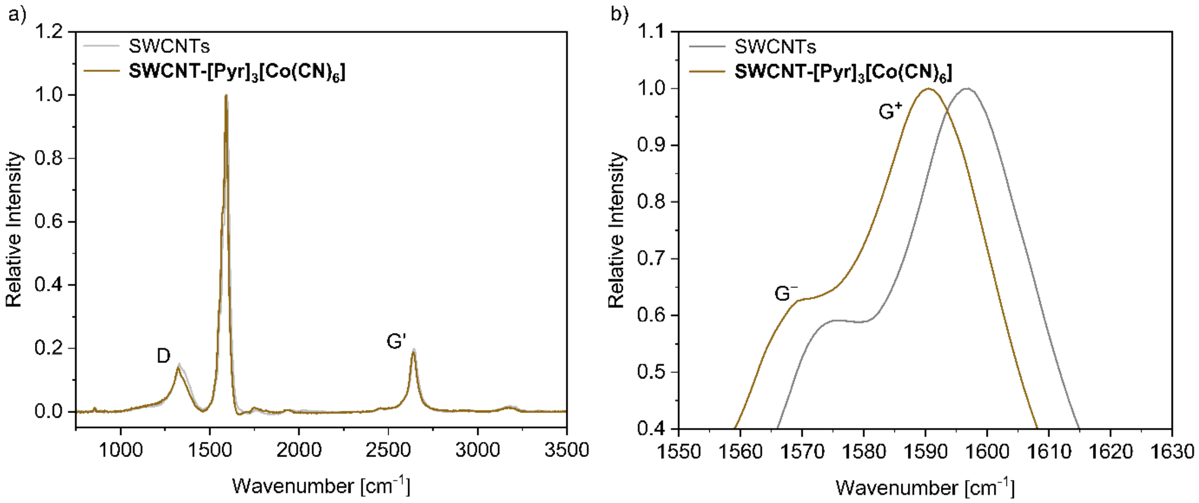

Figure S32: Raman spectra of SWCNTs (gray) and **SWCNT-[Pyr]$_3$[Co(CN)$_6$]** (bronze) over (a) the full scan window and (b) the G-band region, both normalized to the G-band maximum.

2.6 TGA/MS Data

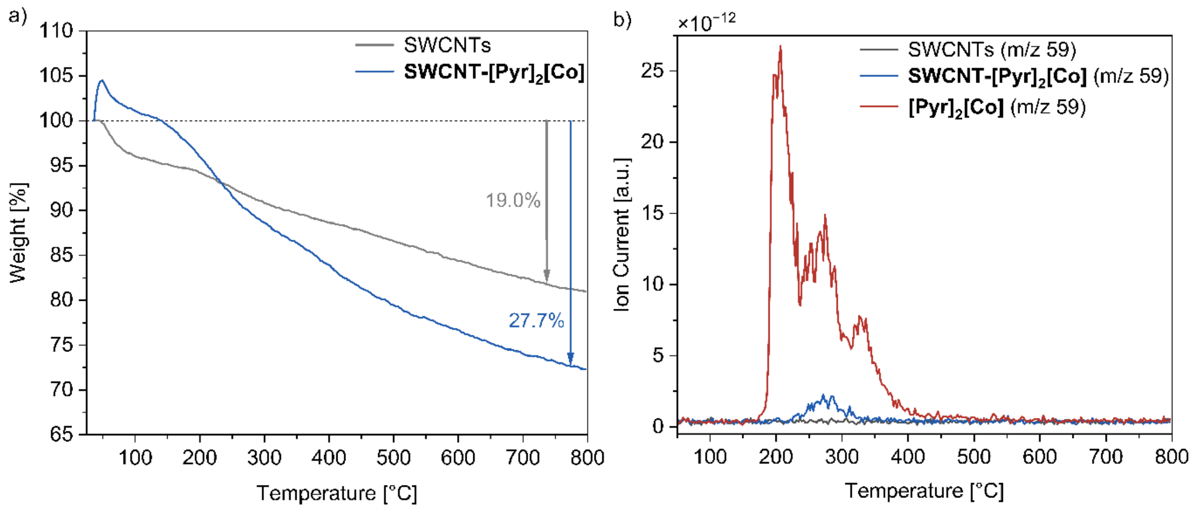

Figure S33: (a) Thermogravimetric analysis (TGA) curves of SWCNTs (gray) and **SWCNT-[Pyr]$_2$[Co]** (blue). (b) TGA/mass spectrometry (MS) ion current curves for the NMe_3^+ fragment (m/z 59) of SWCNTs (gray), **SWCNT-[Pyr]$_2$[Co]** (blue), and **[Pyr]$_2$[Co]** (red).

2.7 TEM Imaging

SWCNTs:

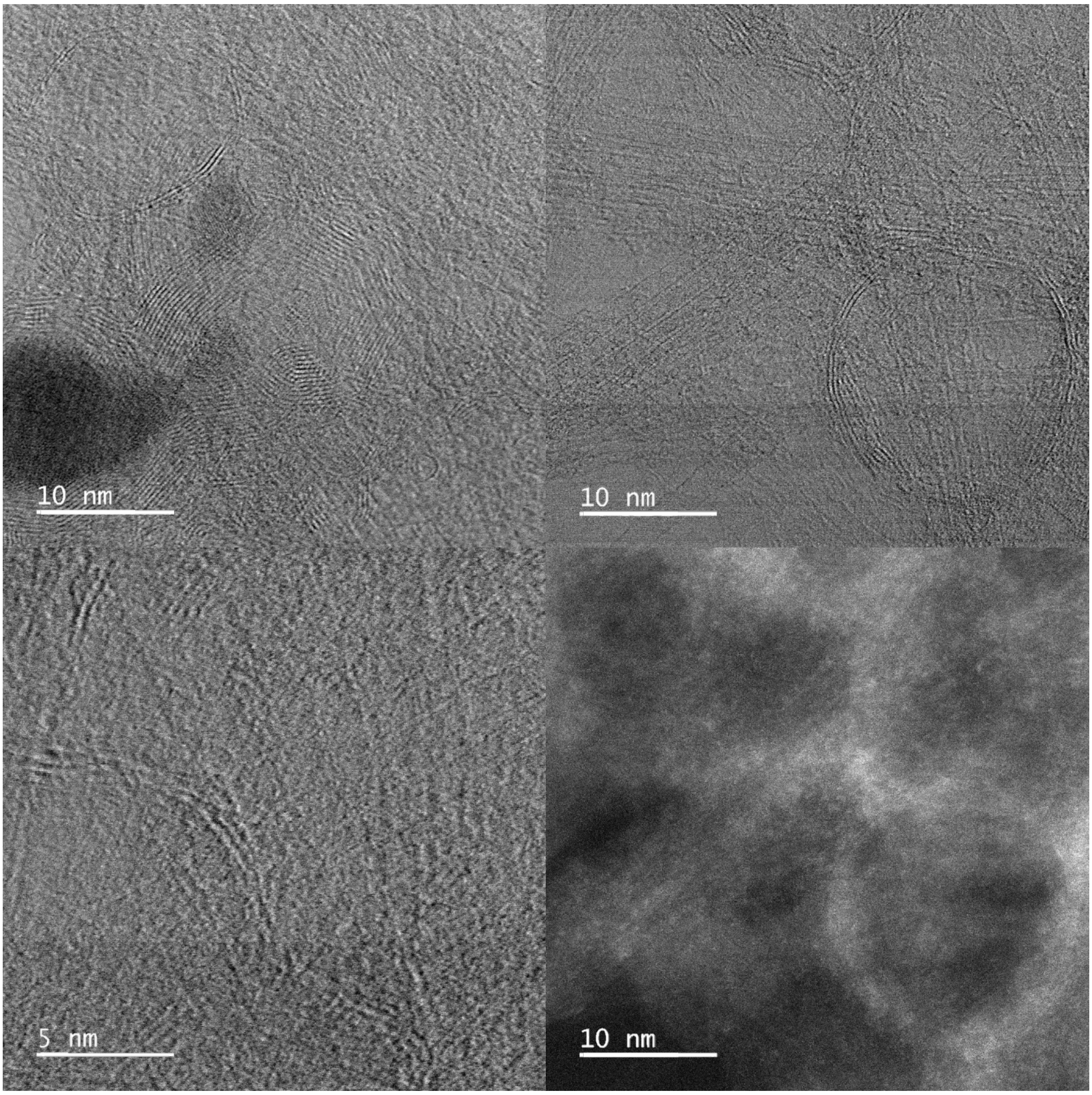

Figure S34: TEM micrographs (top, bottom left), and HAADF STEM micrograph (bottom right) of SWCNTs (see Section 1.2.8 for preparative details).

SWCNT-[Pyr]$_2$[Co]:

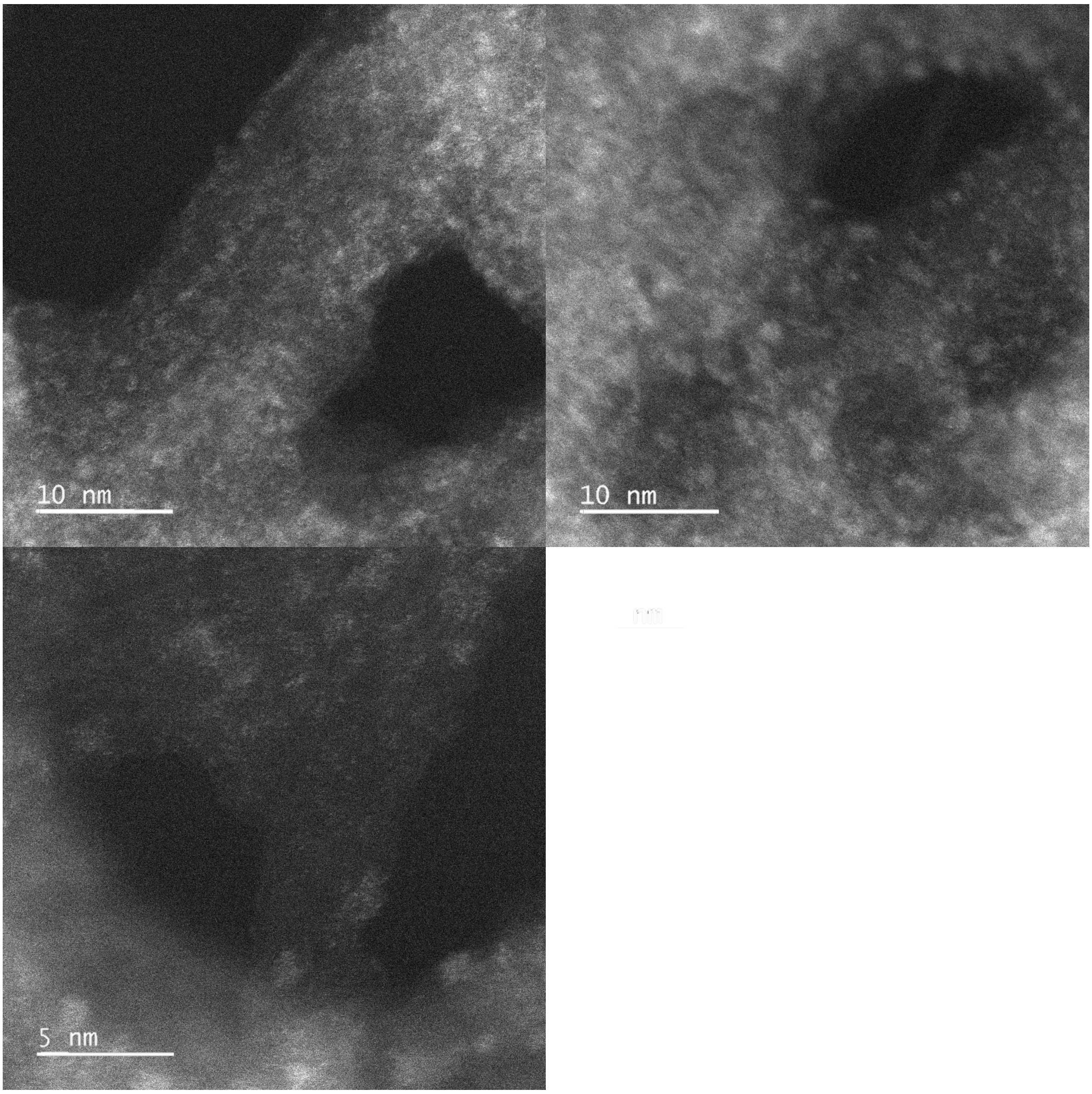

Figure S35: High-angle annular dark-field (HAADF) scanning transmission electron microscopy (STEM) micrographs of purified **SWCNT-[Pyr]$_2$[Co]** (see Section 1.2.8 for preparative details).

SWCNT-[CTA]$_2$[Co]:

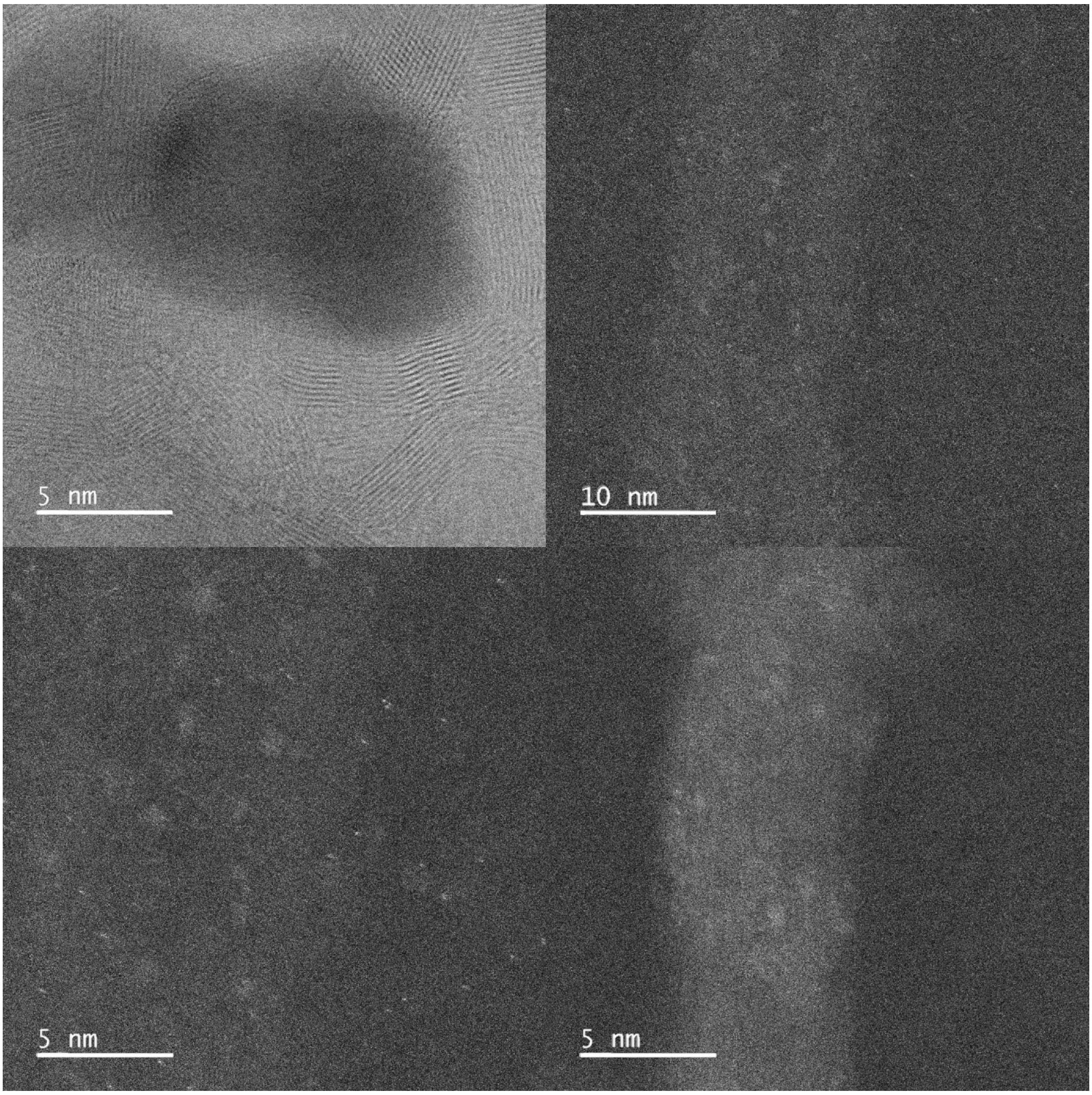

Figure S36: TEM micrographs (top left), and HAADF STEM micrographs (top right, bottom) of **SWCNT-[CTA]$_2$]Co]** (see Section 1.2.8 for preparative details).

SWCNT-$[Pyr]_3[Co(CN)_6]$:

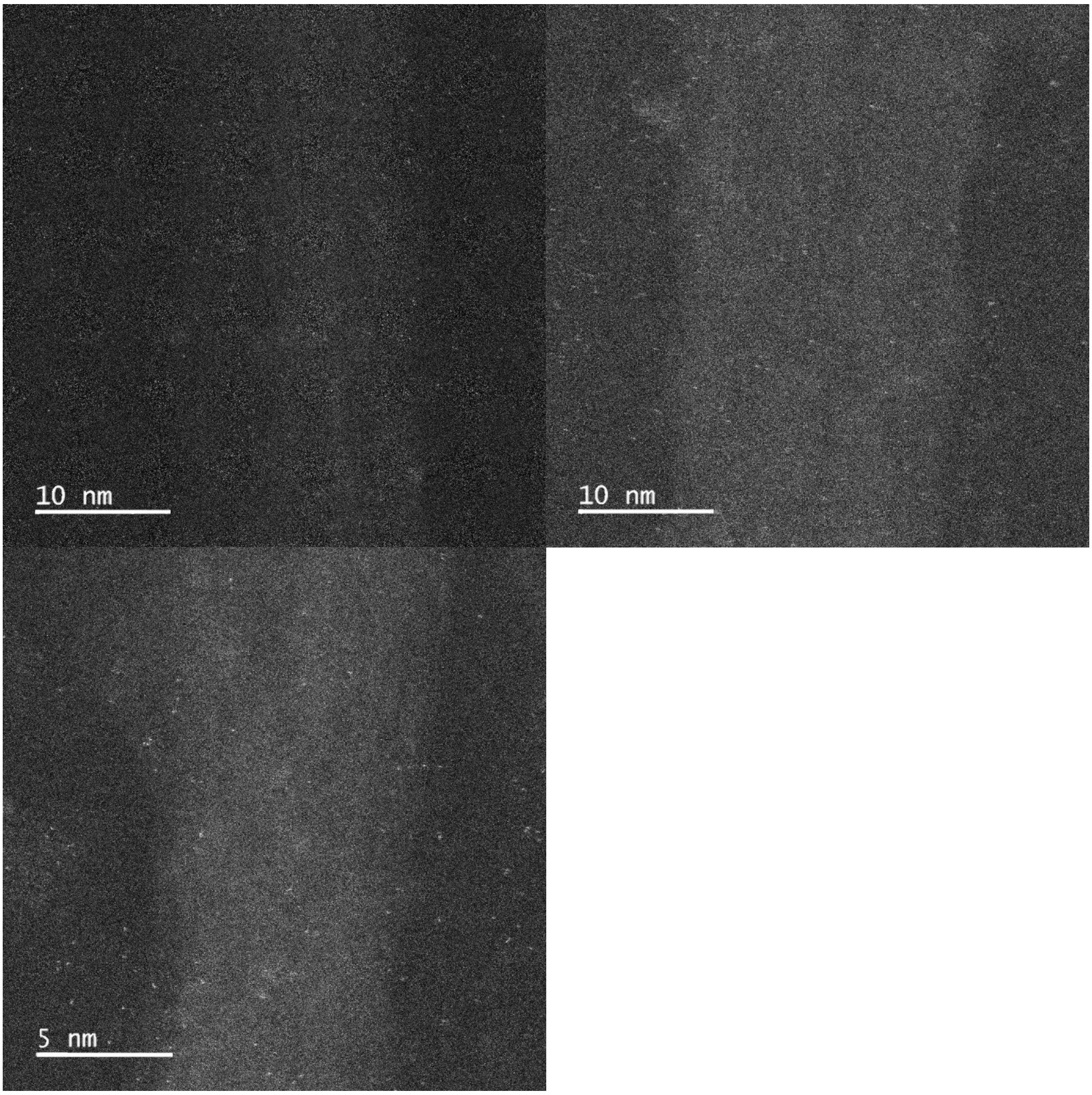

Figure S37: TEM micrographs (top left), and HAADF STEM micrographs (top right, bottom) of **SWCNT-$[Pyr]_3[Co(CN)_6]$** (see Section 1.2.8 for preparative details).

SWCNT-[Pyr][Br]:

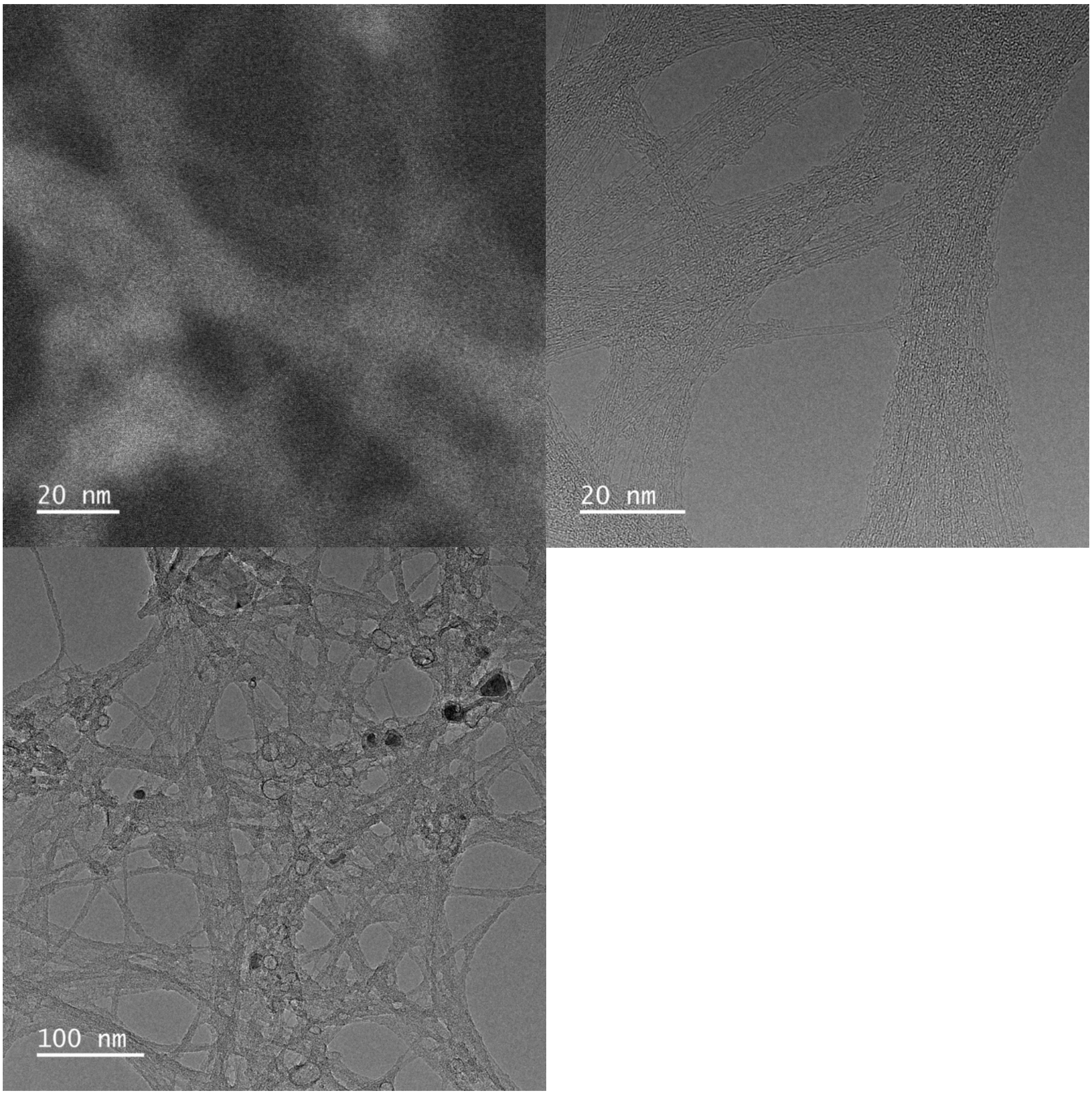

Figure S38: HAADF STEM micrograph (top left), and TEM micrographs (top right, bottom) of purified **SWCNT-[Pyr][Br]** (see Section 1.2.8 for preparative details).

2.8 EDS Spectroscopic Data

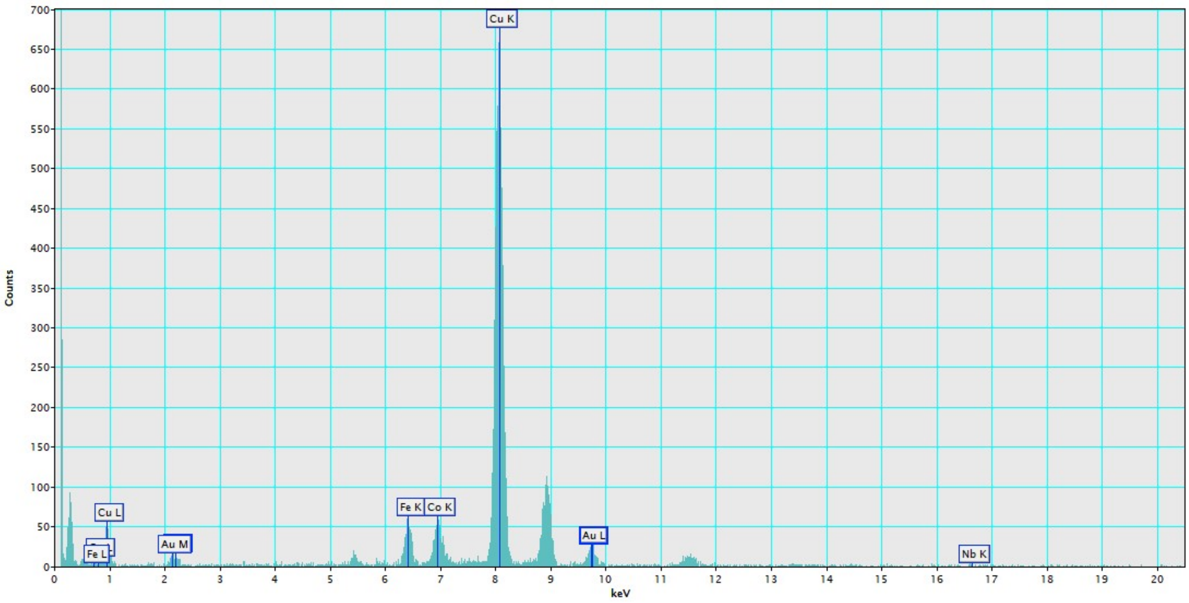

Figure S39: EDS counts measured in the bulk region of SWCNTs. The Cu signal originates from the sample holder; Co and Fe background signals are present in a 1:1 ratio.

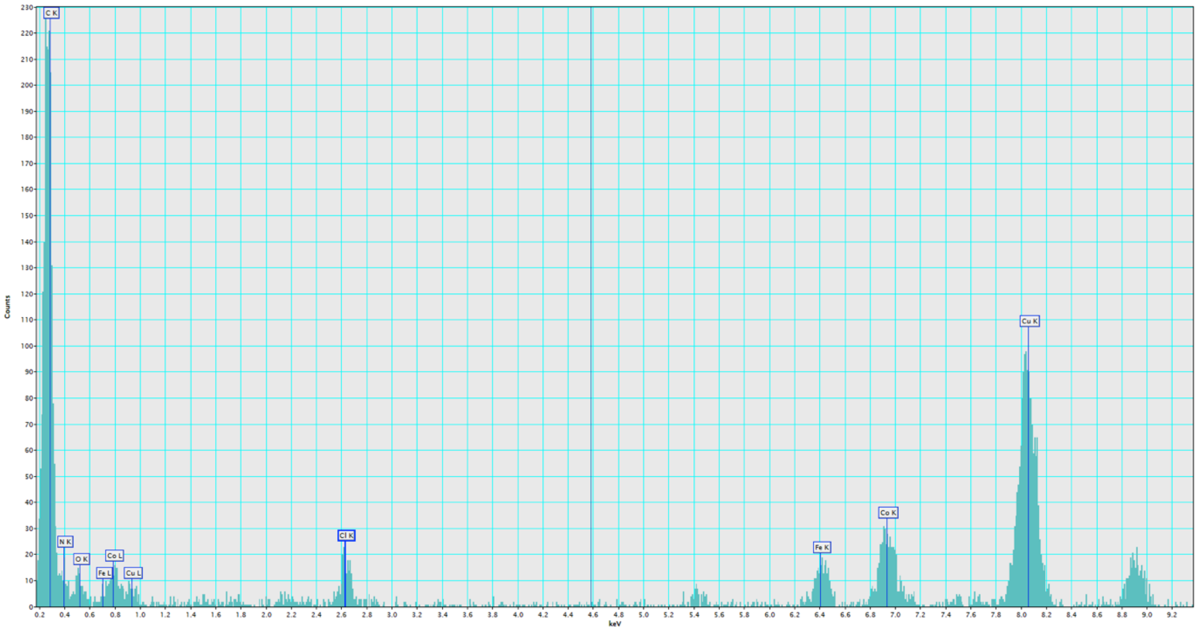

Figure S40: EDS counts measured on a high-contrast agglomerate of purified **SWCNT-[Pyr]$_2$[Co]**. The Cu signal originates from the sample holder; Co and Fe background signals are present in a 1:1 signal ratio.

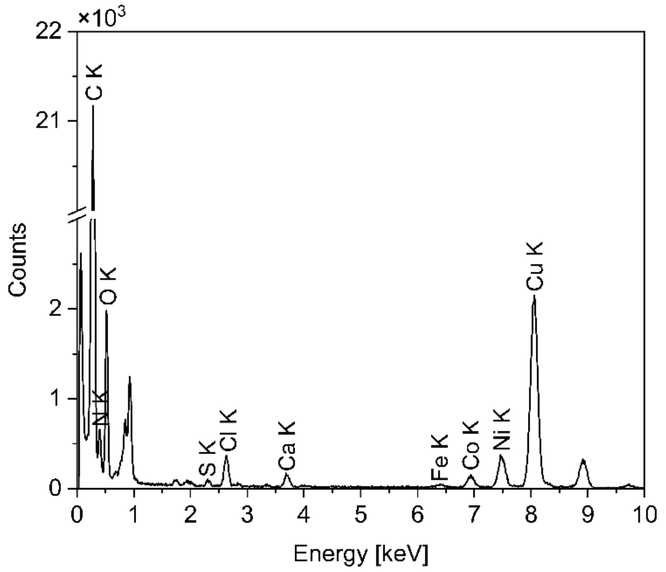

Figure S41: EDS counts measured on a high-contrast agglomerate of purified **SWCNT-[CTA]$_2$[Co]**. The Cu signal originates from the sample holder; Co and Fe background signals are present in a 1:1 signal ratio.

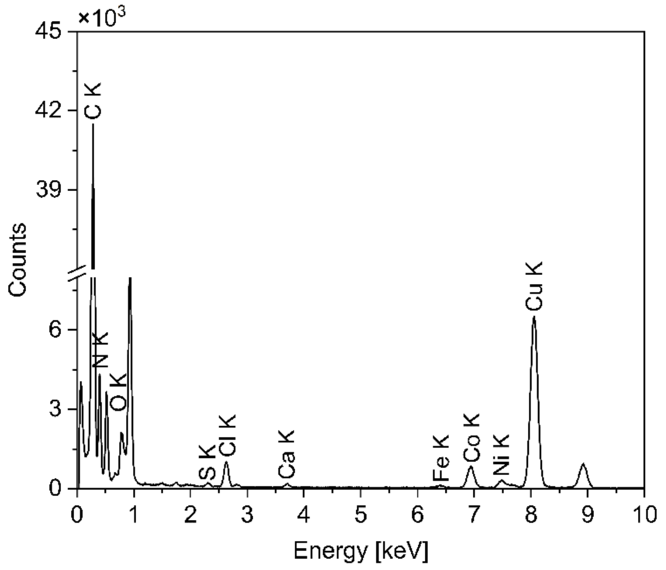

Figure S42: EDS counts measured on a high-contrast agglomerate of purified **SWCNT-[Pyr]$_3$[Co(CN)$_6$]**. The Cu signal originates from the sample holder; Co and Fe background signals are present in a 1:1 signal ratio.

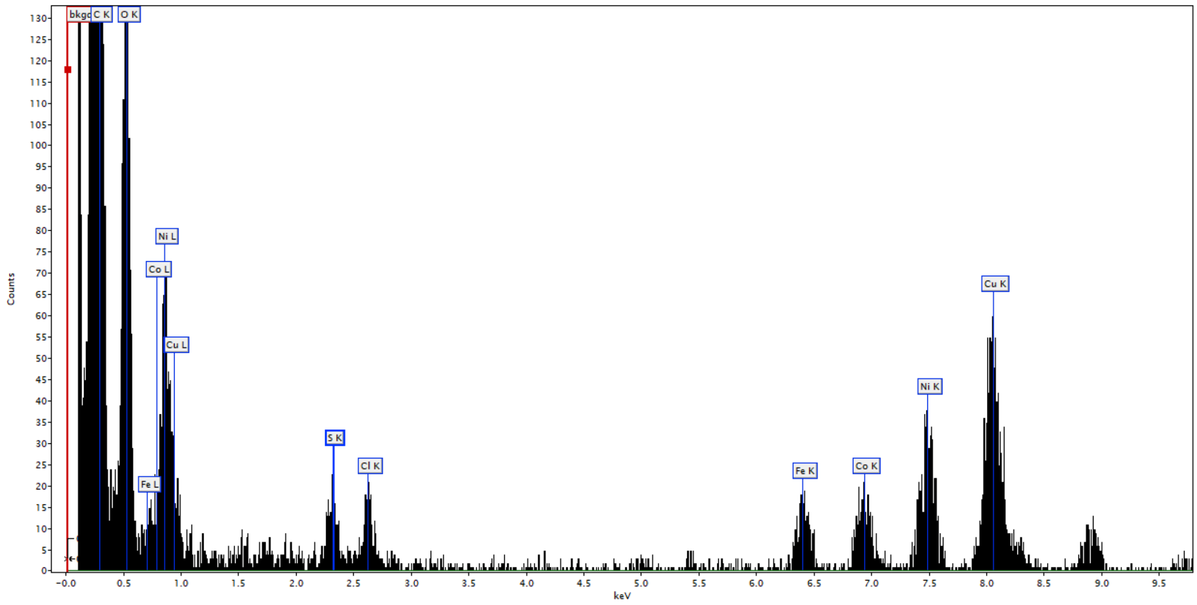

Figure S43: EDS counts measured in the bulk region of purified **SWCNT-[Pyr][Br]**. The Cu signal originates from the sample holder; Co and Fe background signals are present in a 1:1 ratio.

3 Stability Studies and Control Experiments

3.1 Stability Studies of **[Pyr]$_2$[Co]**

Stability Studies in the Solid State

For solid state IR measurements, a dried sample of **[Pyr]$_2$[Co]** (10.0 mg, 10.1 µmol) was stored in a small vial under ambient conditions, unprotected from light (Figure S44a). Spectra were recorded at t = 0 d and after 28 d, with no noticeable changes in the intensities or positions of the absorption bands in the IR spectrum.

For long-term stability monitoring by solid-state EPR spectroscopy, a solution of **[Pyr]$_2$[Co]** (3.00 mg, 3.04 µmol) in CH_2Cl_2 (0.5 mL) was added to an EPR tube. The solvent was subsequently evaporated to produce a homogeneous thin film on the tube walls. After the initial measurement (t = 0 d), the sample's position in the EPR spectrometer was noted to ensure identical positioning for subsequent measurements (Figure S44b). The sample was stored under ambient conditions, unprotected from light. After 28 d, no significant changes in intensity of the EPR spectrum were observed.

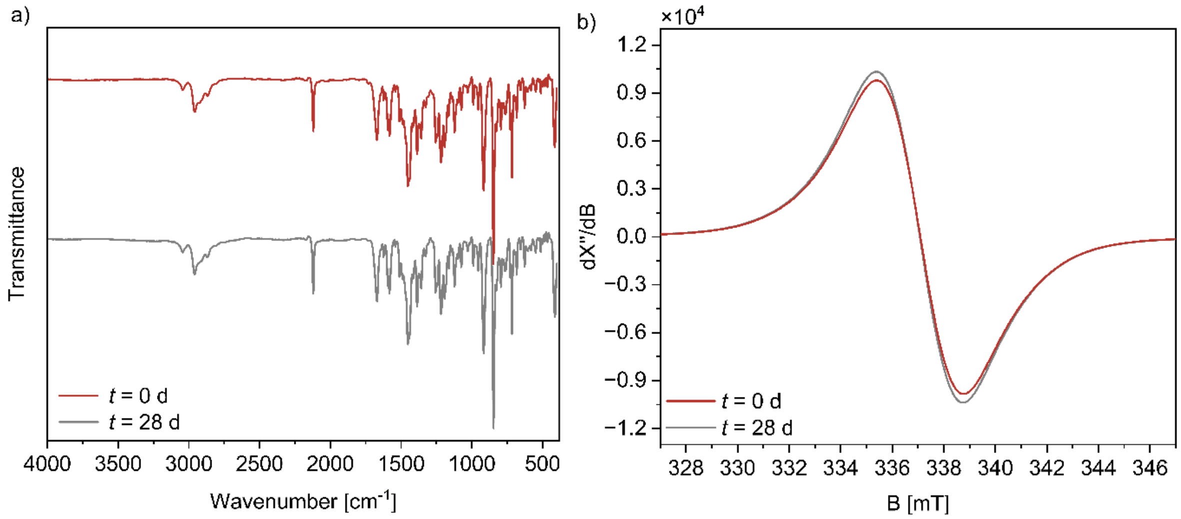

Figure S44: Long-term stability monitoring of **[Pyr]$_2$[Co]** by (a) IR and (b) EPR spectroscopy under ambient conditions, unprotected from light.

Stability Studies in Solution

[Pyr]₂[Co] (6.00 mg, 6.08 µmol, 1.00 equiv) was dissolved in PhMe/MeOH (3:1, 8.00 mL), and the resulting solution was distributed among four EPR tubes. To one tube, $[(n\text{-Bu})_4N][CN]$ (815 µg, 3.04 µmol, 2.00 equiv) was added; to another, *t*-Bu-BQ (669 µg, 3.04 µmol, 2.00 equiv). All four samples were sealed with Parafilm® to minimize solvent evaporation. Three of the samples were stored under ambient light exposure, while one pure **$[Pyr]_2[Co]$** solution was protected from light. All samples were stored under ambient conditions and their decomposition processes monitored by EPR spectroscopy (Figure S45).

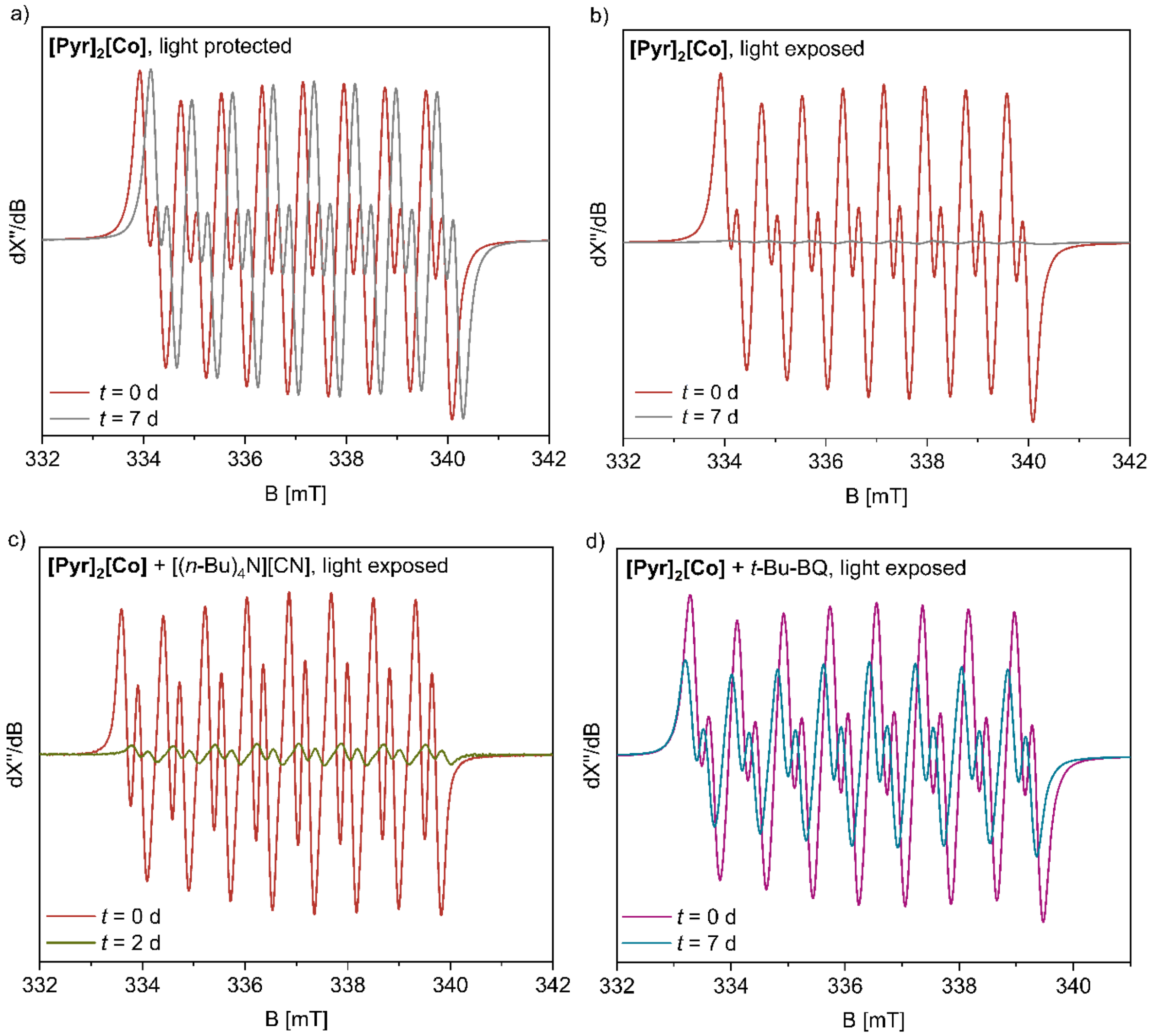

Figure S45: **$[Pyr]_2[Co]$** in PhMe/MeOH (3:1, v/v) after storage for 7 d under (a) light protection, (b) ambient light exposure, (c) ambient light exposure in the presence of $[(n\text{-Bu})_4N][CN]$ and (d) ambient light exposure in the presence of *t*-Bu-BQ.

To evaluate the influence of oxygen- and moisture-exposure on the stability of **[Pyr]₂[Co]** in solution, a 25.0 μM stock solution of **[Pyr]₂[Co]** in MeOH (V_{tot} = 20 mL) was prepared, and its UV-Vis spectrum was recorded (Figure S46). The solution was then divided into two portions, each stored for 3 d under ambient light: one in an argon-filled glovebox and the other under ambient atmosphere. Both solutions were analyzed again by UV-Vis spectroscopy, showing a significant decrease in signal intensity indicative of decomposition, with no discernible difference between the two samples.

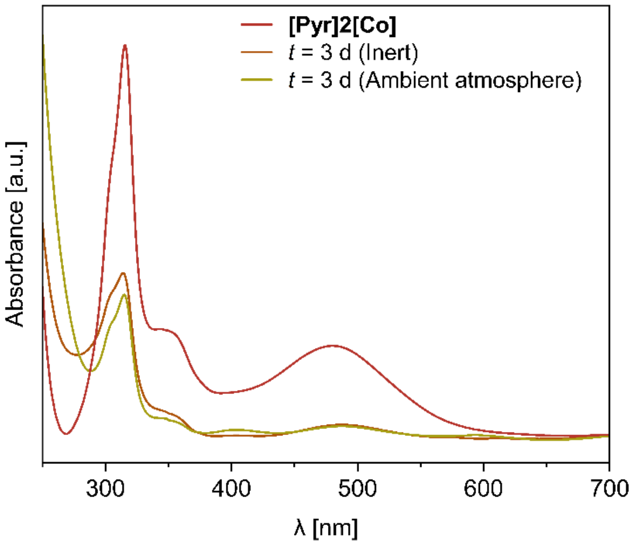

Figure S46: UV-Vis spectra of **[Pyr]$_2$[Co]** in MeOH (12.5 μM) at *t* = 0 d (red) and after storage for 4 d under ambient light in an inert atmosphere (orange) or ambient atmosphere (green).

The solvent of the solution stored under inert conditions was then removed *in vacuo*, and the residue was dissolved in MeCN-d_3. The subsequently recorded ^{1}H NMR spectrum (Figure S47) displayed all expected resonances for the free *t*-Bu-BQ ligand (red), and for the pyrene cation **[Pyr]$^+$** (bronze). Given the diamagnetic character of the spectrum, the paramagnetic cobalt anion **[Co]$^{2-}$** likely decomposed.

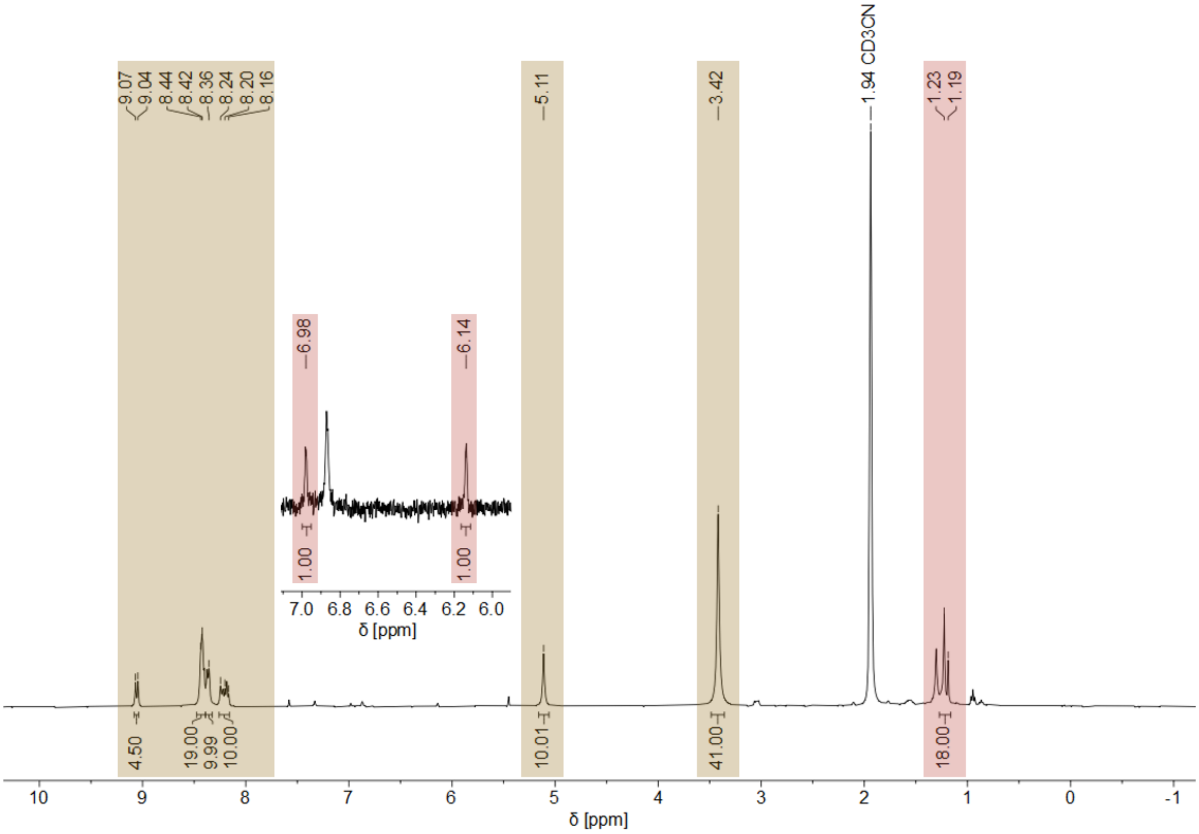

Figure S47: ^{1}H NMR spectrum (300 MHz, MeCN-d_3, 298 K) of a decomposed sample of **$[Pyr]_2[Co]$** after storage under inert conditions, exposed to ambient light, for 3 d. The signal integrals are referenced to *t*-Bu-BQ (red), which is present in a 1:4.5 ratio relative to **$[Pyr]^+$** (bronze).

3.2 Solvatochromism of **[Pyr]$_2$[Co]**

The complex **[Pyr]$_2$[Co]** exhibits pronounced negative solvatochromism (Figure S48, left),[17] showing a blue shift in the absorption maxima of its UV-Vis spectrum with increasing solvent polarity (Figure S48, right), an effect commonly reported for Co(III) complexes.[18–20]

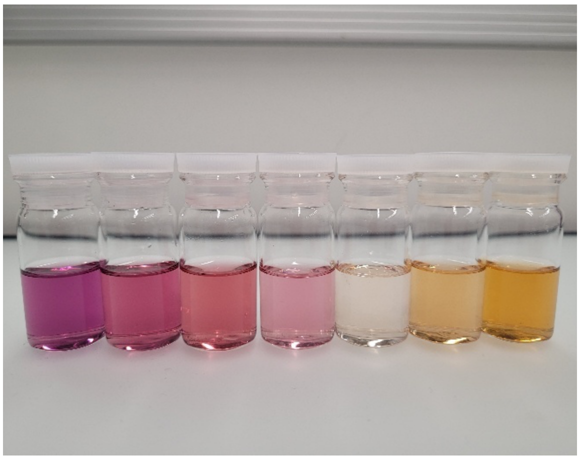

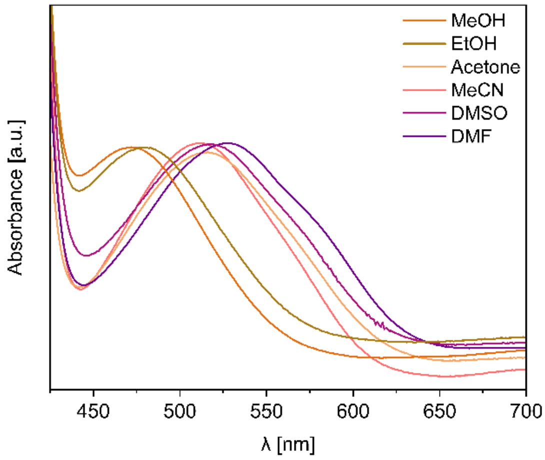

Figure S48: Photograph (left) of **[Pyr]$_2$[Co]** (250 µM) in DMF, DMSO, MeCN, acetone, CH_2Cl_2, EtOH and MeOH (from left to right) and UV-Vis spectra (right) of **[Pyr]$_2$[Co]** (25 µM) in MeOH (orange), EtOH (orange), acetone (peach), MeCN (melon), DMSO (magenta) and DMF (purple), zoomed in on the lowest-energy absorption band.

3.3 Stability Studies of SWCNT composite materials

3.3.1 Solid-State Stability of **SWCNT-[Pyr]$_2$[Co]**

A fresh batch of the material was prepared according to the general procedure (see Section 1.2.7), using SWCNTs (2.00 mg) and **[Pyr]$_2$[Co]** (2.00 mg, 2.02 µmol). After purification, the residue was suspended in CH_2Cl_2 (1.0 mL) and the suspension was distributed into two EPR tubes. The solvent was removed *in vacuo* and the composite was dried *in vacuo* for 1 h. The samples were stored under ambient conditions, one exposed to light, and the other protected from light.

EPR spectra were collected according to the general procedure described above and the traces were normalized to the intrinsic SWCNT-signal (assumed to remain constant over time and across measurements) according to the following equation:[21,22]

$$y(t_n)_{norm} = (y(t_n) - \bar{y}(t_n)) \cdot k + \bar{y}(t_0)$$

where:

- $y(t_n)_{norm}$: normalized y-axis values at timepoint $t \neq 0$
- $y(t_n)$: y-axis values at timepoint t
- $\bar{y}(t_n)$: mean of y-axis values at timepoint t
- $\bar{y}(t_0)$: mean of y-axis values at timepoint t_0
- k (scaling factor), defined as:

$$k = \frac{y(t_0)_{max} - y(t_0)_{min}}{y(t_n)_{max} - y(t_n)_{min}}$$

with $y(t_n)_{min/max}$ being the minimum/maximum y-value of the SWCNT signal at g = 2.0037 at timepoint t. Y- and x-offsets were applied as needed. Lastly, the spectra were broad line-corrected by subtracting fourth-order polynomial fits from the respective EPR traces.

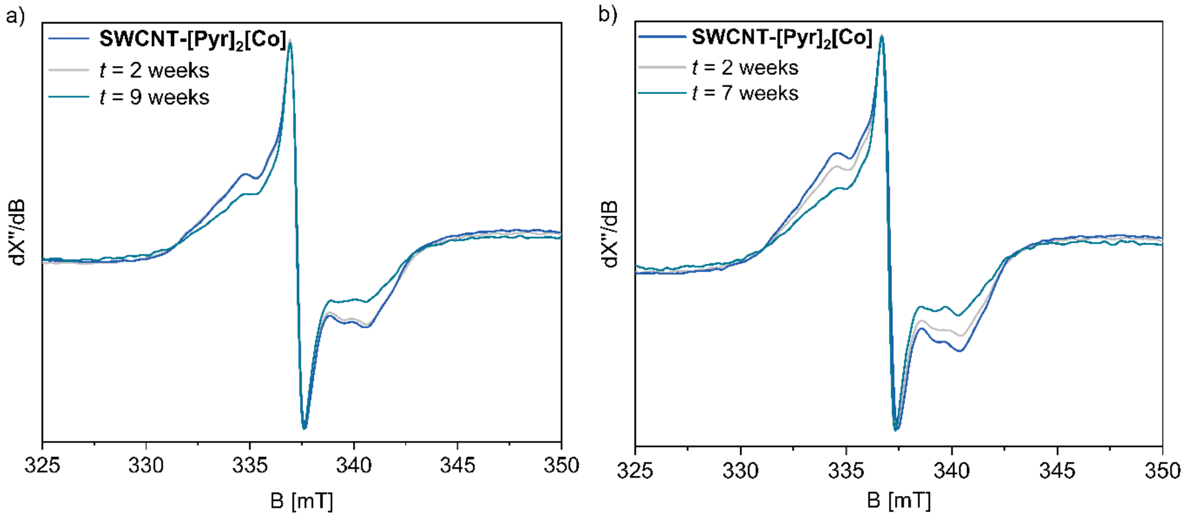

Figure S49: X-band CW EPR spectra of **SWCNT-[Pyr]$_2$[Co]** collected at 298 K over a 7-week storage period under (a) light protection and (b) light exposure at ambient conditions. All spectra were broad-line–corrected by subtracting fourth-order polynomial fits.

3.3.2 Robustness of Functionalization of **SWCNT-[Pyr]$_2$[Co]**

The robustness of the functionalization of SWCNTs with **[Pyr]$_2$[Co]** was evaluated by subjecting the hybrid material to a series of sonication and washing steps.

A scintillation vial was charged with SWCNTs (1.00 mg), **[Pyr]$_2$[Co]** (500 µg, 500 nmol) and *o*-DCB (1.0 mL). The vial was sealed with Parafilm®, mounted by its cap and sonicated for 1 h. The resulting suspension was filtered through a nylon membrane filter (0.2 µm) and washed with CH_2Cl_2 until the filtrate was colorless. The product was then scraped from the filter, transferred into an EPR tube using CH_2Cl_2 (0.5 mL), and analyzed by EPR spectroscopy (Figure S50b, blue trace).

The supernatant CH_2Cl_2 was then decanted and replaced with fresh CH_2Cl_2, and the EPR tube was sonicated again for 1 h. The supernatant was transferred to a second EPR tube and analyzed by EPR spectroscopy (Figure S50a, rose trace). The solid residue in the first EPR tube was also analyzed (Figure S50b, petrol trace).

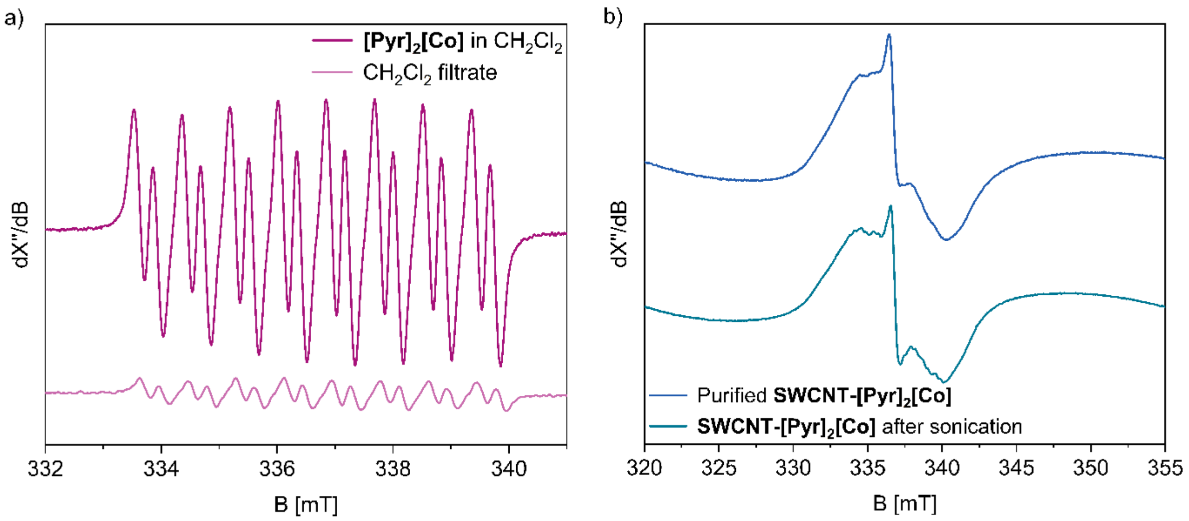

Figure S50: (a) X-band CW EPR spectra of a saturated solution of **[Pyr]$_2$[Co]** in CH_2Cl_2 (magenta) and the decanted CH_2Cl_2 supernatant of the sonicated hybrid material (rose). (b) X-band CW EPR spectra of the purified hybrid material directly after synthesis (blue) and after additional sonication and washing (petrol). All spectra were collected at 298 K. **SWCNT-[Pyr]$_2$[Co]** spectra were broad-line–corrected by subtracting second-order polynomial fits from the respective EPR spectra.

Notably, the EPR spectrum of the CH_2Cl_2 filtrate indicates a **[Pyr]$_2$[Co]** concentration well below saturation, while the broad EPR signal of the cobalt complex in **SWCNT-[Pyr]$_2$[Co]** showed no discernible change after additional sonication of the hybrid material. These results confirm both the stability of **SWCNT-[Pyr]$_2$[Co]** and the robustness of **[Pyr]$_2$[Co]** immobilization on the nanotubes, even under harsh manipulation conditions.

3.3.3 Robustness of Functionalization of **SWCNT-[CTA]$_2$[Co]** and **SWCNT-[Pyr]$_3$[Co(CN)$_6$]**

The robustness of the functionalization of SWCNTs with **[CTA]$_2$[Co]** was evaluated according to an identical procedure as the one used for **SWCNT-[Pyr]$_2$[Co]**. However, **[CTA]$_2$[Co]** was found to be unable to withstand these tests, with the EPR feature corresponding to the complex on the SWCNTs having disappeared entirely after the first washing and sonication step.

The robustness of the functionalization of SWCNTs with **[Pyr]$_3$[Co]** was evaluated in a manner similar to the above.

A scintillation vial was charged with SWCNTs (1.00 mg), **[Pyr]$_3$[Co(CN)$_6$]** (1.00 mg, 891 nmol) and *o*-DCB (1.5 mL). The vial was sealed with Parafilm®, mounted by its cap and sonicated for 1 h. The resulting dispersion was transferred into an *Eppendorf* tube and centrifuged for 10 min. The supernatant was decanted away. The residue was washed with MeOH (1.5 mL). The sample was then centrifuged again and the washing steps were repeated twice.

The residue was suspended in CD_2Cl_2 (0.8 mL) and the mixture transferred into a scintillation vial. The vial was sealed with Parafilm®, mounted by its cap and sonicated for 30 min. The resulting dispersion was transferred into an *Eppendorf* tube and centrifuged for 10 min. The supernatant was decanted into an NMR tube and analyzed by NMR spectroscopy. The resulting NMR spectrum did not show any peaks corresponding to free **[Pyr]$_3$[Co(CN)$_6$]**. Repetition of the experiment in CD_3CN instead of CD_2Cl_2 during the initial sonication gave the same result. These experiments thus highlight the robustness of functionalization of **[Pyr]$_3$[Co(CN)$_6$]** on SWCNTs, even when subjected to a series of manipulations.

To control for the presence of **[Pyr]$_3$[Co(CN)$_6$]** in **SWCNT-[Pyr]$_3$[Co(CN)$_6$]**, a final extraction with DMSO-d_6 was performed. Specifically, **SWCNT-[Pyr]$_3$[Co(CN)$_6$]** was suspended in DMSO-d_6 (0.8 mL), and the mixture transferred into a scintillation vial. The vial was sealed with Parafilm®, mounted by its cap and sonicated for 30 min. The resulting dispersion was transferred into an *Eppendorf* tube and centrifuged for 10 min. The supernatant was decanted into an NMR tube and analyzed by NMR spectroscopy. The resulting NMR spectrum (Figure S51) showed all expected peaks corresponding to free **[Pyr]$_3$[Co(CN)$_6$]**, demonstrating its presence of in the material.

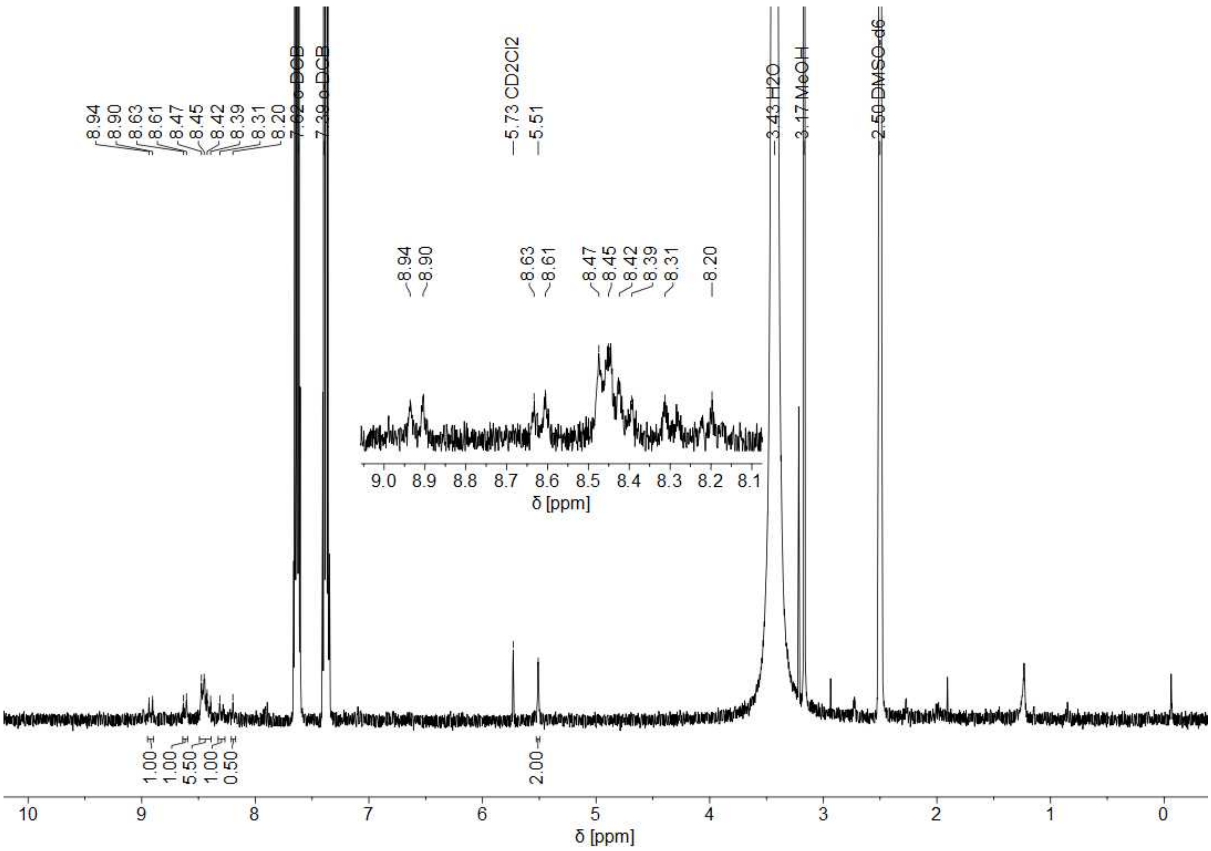

Figure S51: ^{1}H NMR spectrum (300 MHz, DMSO-d_6, 298 K) of **[Pyr]$_3$[Co(CN)$_6$]** after immobilization on SWCNTs and subsequent extraction with DMSO-d_6. The CH_3 signal of the trimethylammonium moiety is obscured by the H_2O peak.

3.3.4 Functionalization Attempts with **[(*n*-Bu)$_4$N]$_2$[Co]** and **[Pyr][Br]**

To showcase the crucial role of the pyrene cations in **[Pyr]$_2$[Co]** for immobilization on SWCNTs, a control experiment was performed using **[(*n*-Bu)$_4$N]$_2$[Co]**. A 5 mL scintillation vial was charged with SWCNTs (1.00 mg), **[(*n*-Bu)$_4$N]$_2$[Co]** (1.00 mg, 1.00 µmol) and *o*-DCB (1.5 mL). The vial was sealed with *Parafilm*®, the dispersion was sonicated for 60 min and then transferred to an *Eppendorf* tube. To remove residual **[(*n*-Bu)$_4$N]$_2$[Co]**, the mixture was centrifuged for 10 min at 16 873 g. The supernatant was decanted, the residual solid was washed with MeOH (3 x 1.0 mL) and then dried *in vacuo*, before being transferred into an EPR tube with CH_2Cl_2 (0.5 mL).

As shown in Figure S52, the characteristic EPR trace assigned to **[Pyr]$_2$[Co]** in **SWCNT-[Pyr]$_2$[Co]** is absent from the spectrum of the material obtained in this control experiment, underscoring the importance of the pyrene cations for successful immobilization.

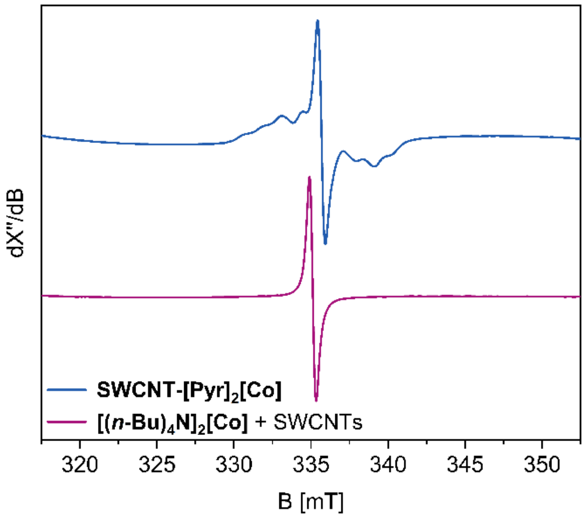

Figure S52: X-band CW EPR spectra of **SWCNT-[Pyr]$_2$[Co]** (blue) and the product from the functionalization attempt of SWCNTs with **[(*n*-Bu)$_4$N]$_2$[Co]** (magenta), collected at 298 K. Both spectra were broad-line-corrected by subtracting third-order polynomial fits. The apparent *g*-value difference between spectra stems from a lack of referencing, not an actual *g*-shift difference.

To confirm that the EPR signal assigned to **SWCNT-[Pyr]$_2$[Co]** is not due to the immobilization of the pyrene moiety alone on the nanotube surface, a control experiment was performed using **[Pyr][Br]**. A 5 mL scintillation vial was charged with SWCNTs (1.00 mg), **[Pyr][Br]** (1.00 mg, 1.00 µmol) and *o*-DCB (1.5 mL). The vial was sealed with *Parafilm*®, the dispersion was sonicated for 60 min and then transferred to an *Eppendorf* tube. To remove residual **[Pyr][Br]**, the mixture was centrifuged for 10 min at 16 873 g. The supernatant was decanted, and the residual solid was washed with MeOH (3 x 1.0 mL) and dried *in vacuo* before being transferred into an EPR tube with CH_2Cl_2 (0.5 mL).

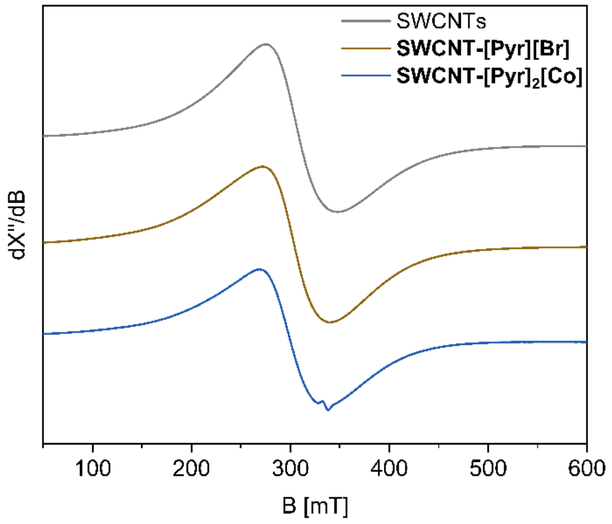

Figure S53: X-band CW EPR spectra of SWCNTs (gray), **SWCNT-[Pyr][Br]** (bronze), and **SWCNT-[Pyr]$_2$[Co]** (blue), collected at 298 K.

As shown in Figure S53, the characteristic EPR feature assigned to **[Pyr]$_2$[Co]** in **SWCNT-[Pyr]$_2$[Co]** is absent from the spectrum of the obtained material, corroborating its correct assignment.

4 Additional Electrochemical Data

Note: For electrode preparation procedures, see Section 1.2.8 (CV Studies). Prior to all anion sensing experiments, one full-window scan was performed, to ensure the integrity of the complex in the composite. This was followed by a scan of the anodic window, again in the neat electrolyte solution, before the respective $[(n\text{-Bu})_4N]^+$ salt (25 mM in 1,2-DFB, 50 µL, 1.25 µmol) was added. The solutions were mixed by gentle shaking and another CV of the anodic window was recorded. This procedure was repeated seven times, until a total of 10.0 µmol of the respective $[(n\text{-Bu})_4N]^+$ salt had been added. The reported final concentrations of the $[(n\text{-Bu})_4N]^+$ salts account for dilution from stock solution additions. Thus, all titrations of the cobalt complexes in solution correspond to a total addition of 1.0 equiv of $[(n\text{-Bu})_4N][X]$.

4.1 Electrochemical Cyanide Sensing with **[Pyr]₂[Co]**

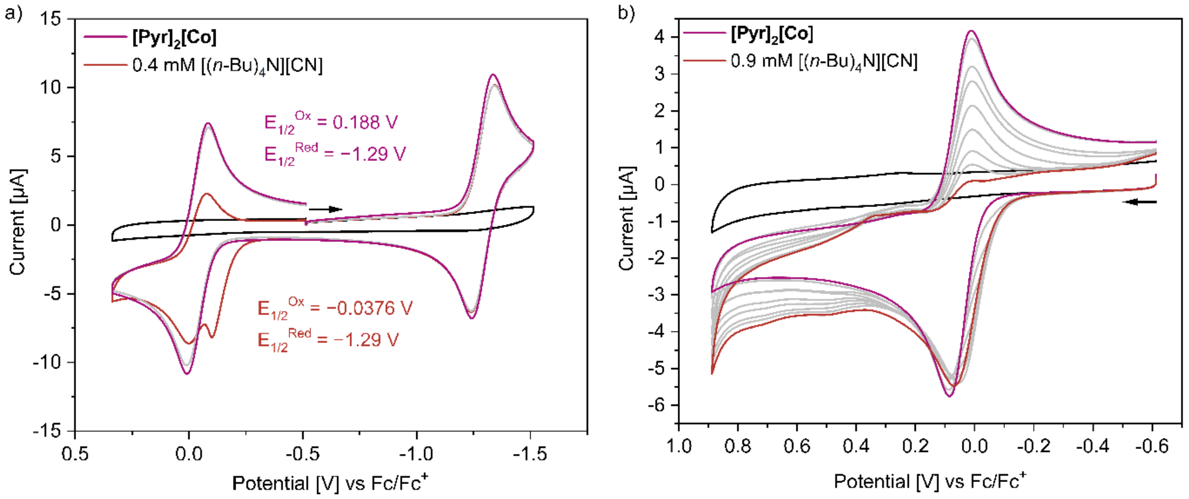

Figure S54: (a) Cyclic voltammograms of **[Pyr]₂[Co]** (1.0 mM) during titration with $[(n\text{-Bu})_4N][CN]$ (12.5 mM in MeCN). The initial CV is shown in magenta, intermediate traces (0.2 equiv additions, total 0.4 equiv) in gray, and the final CV in red. (b) Cyclic voltammogram of the anodic window of **[Pyr]₂[Co]** under the same conditions with smaller stepwise $[(n\text{-Bu})_4N][CN]$ additions (0.125 equiv) showing the initial trace in magenta, intermediates in gray, and the final CV (1.0 equiv total) in red. All scans were recorded at a scan rate of 100 mV/s and are shown alongside the background trace (black) of pure electrolyte. Measurements were performed with 0.10 M $[(n\text{-Bu})_4N][PF_6]$ as supporting electrolyte in MeCN using a glassy carbon working electrode, referenced to Fc/Fc^+.

4.2 Electrochemical Cyanide Sensing with **[(*n*-Bu)$_4$N]$_2$[Co]**

MeCN Solvent:

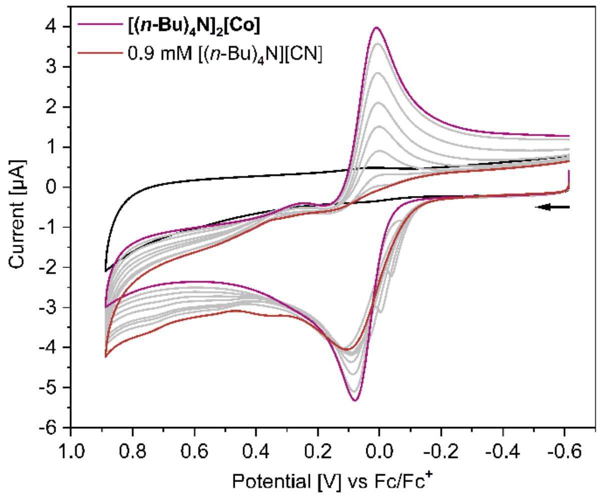

Figure S55: Cyclic voltammogram of **[(*n*-Bu)$_4$N]$_2$[Co]** (1.0 mM in MeCN) upon titration with [(*n*-Bu)$_4$N][CN] (12.5 mM in MeCN). The initial CV is shown in magenta, intermediate CVs (0.125 equiv additions) in gray, and the final CV (after addition of 1.0 equiv [(*n*-Bu)$_4$N][CN]; final concentration: 925 µM) in red. All scans were recorded at a scan rate of 100 mV/s and are shown alongside the background (black) of pure electrolyte. Measurements were performed with 0.10 M [(*n*-Bu)$_4$N][PF$_6$] as supporting electrolyte in MeCN using a glassy carbon working electrode, referenced to Fc/Fc$^+$.

1,2-DFB Solvent:

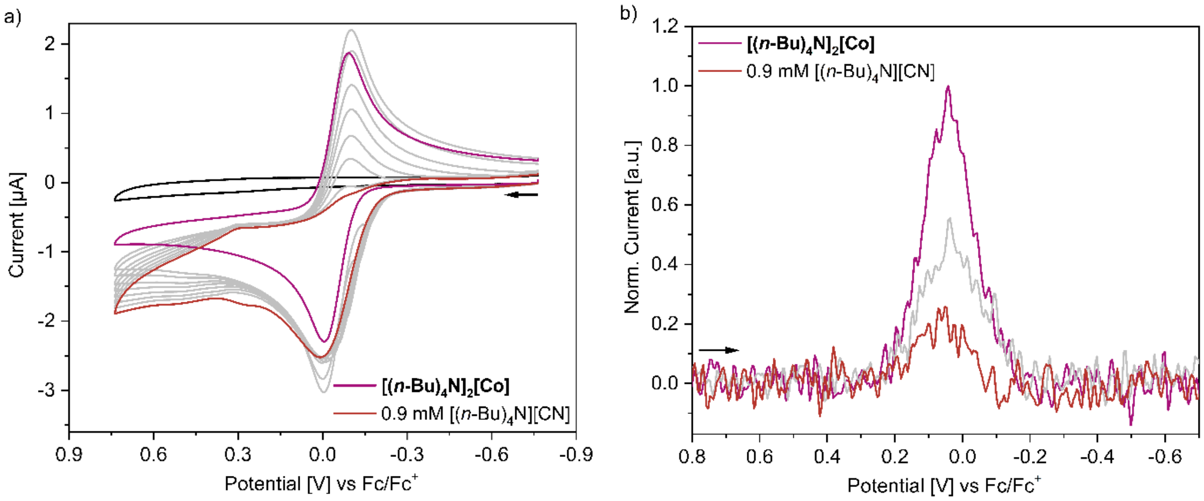

Figure S56: (a) Cyclic voltammogram of **[(*n*-Bu)$_4$N]$_2$[Co]** (1.0 mM in 1,2-DFB) upon titration with [(*n*-Bu)$_4$N][CN] (12.5 mM in 1,2-DFB). The initial CV is shown in magenta, intermediate CVs (0.125 equiv additions) in gray, and the final CV (after addition of 1.0 equiv [(*n*-Bu)$_4$N][CN]; final concentration: 925 µM) in red. All scans were recorded at a scan rate of

100 mV/s and are shown alongside the background (black) of pure supporting electrolyte. (b) Square wave voltammogram of **[(*n*-Bu)$_4$N]$_2$[Co]** (1.0 mM in 1,2-DFB) upon addition of 0.5 equiv (gray) and 1.0 equiv (red) [(*n*-Bu)$_4$N][CN] (12.5 mM in 1,2-DFB; final concentration: 925 µM). 0.10 M [(*n*-Bu)$_4$N][PF_6] supporting electrolyte in 1,2-DFB, r.t., glassy carbon working electrode, referenced to Fc/Fc^+.

Note: Identical electrochemical reactivity was observed for **[(*n*-Bu)$_4$N]$_2$[Co]** and **[Pyr]$_2$[Co]** with [(*n*-Bu)$_4$N][CN], with no detectable influence of the counterion identity within the investigated electrochemical window. **[(*n*-Bu)$_4$N]$_2$[Co]** was therefore selected as the soluble reference complex for all subsequent electrochemical experiments and selectivity studies, due to its favorable solubility in 1,2-DFB.

4.3 Electrochemical Cyanide Sensing with **SWCNT-[Pyr]$_2$[Co]**

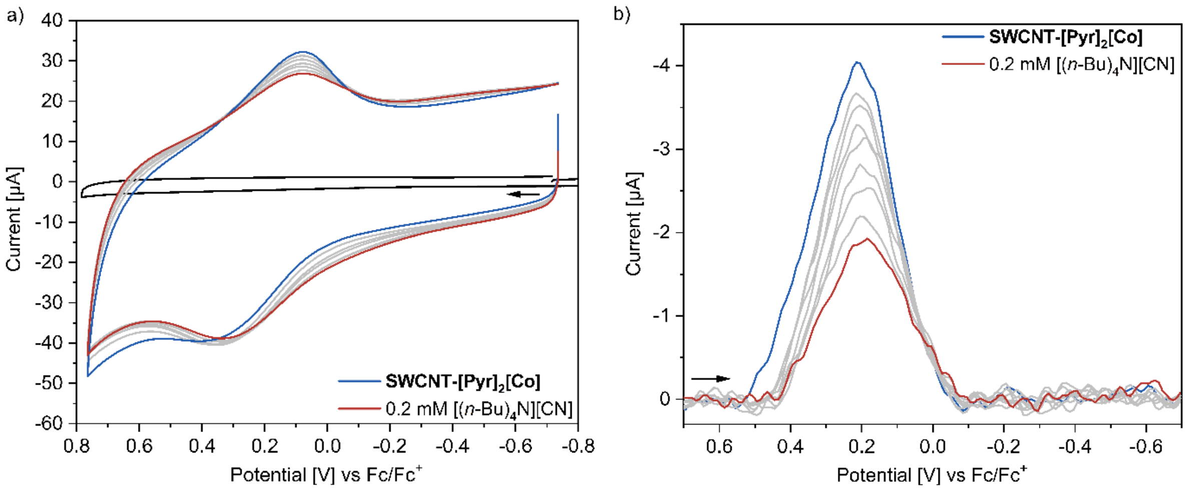

Figure S57: (a) Cyclic voltammogram of **SWCNT-[Pyr]$_2$[Co]** (blue; drop-cast on glassy carbon working electrode), upon titration with [(*n*-Bu)$_4$N][CN] (2.50 mM in 1,2-DFB). The initial CV is shown in blue, intermediate CVs (0.125 µmol additions) in gray, and the final CV (after addition of 1.00 µmol; final concentration: 185 µM) in red. All scans were recorded at a scan rate of 100 mV/s and are shown alongside the background (black) of pure supporting electrolyte. (b) Square wave voltammograms of **SWCNT-[Pyr]$_2$[Co]** after titration with [(*n*-Bu)$_4$N][CN] (2.50 mM in 1,2-DFB). The initial SWV is shown in blue, intermediate SWVs (0.125 equiv additions) in gray, and the final SWV (after addition of 1.00 µmol; final concentration: 185 µM) in red. 0.10 M [(*n*-Bu)$_4$N][PF_6] supporting electrolyte in 1,2-DFB, r.t., glassy carbon working electrode, referenced to Fc/Fc$^+$.

4.4 Selectivity studies: **[(*n*-Bu)$_4$N]$_2$[Co]**

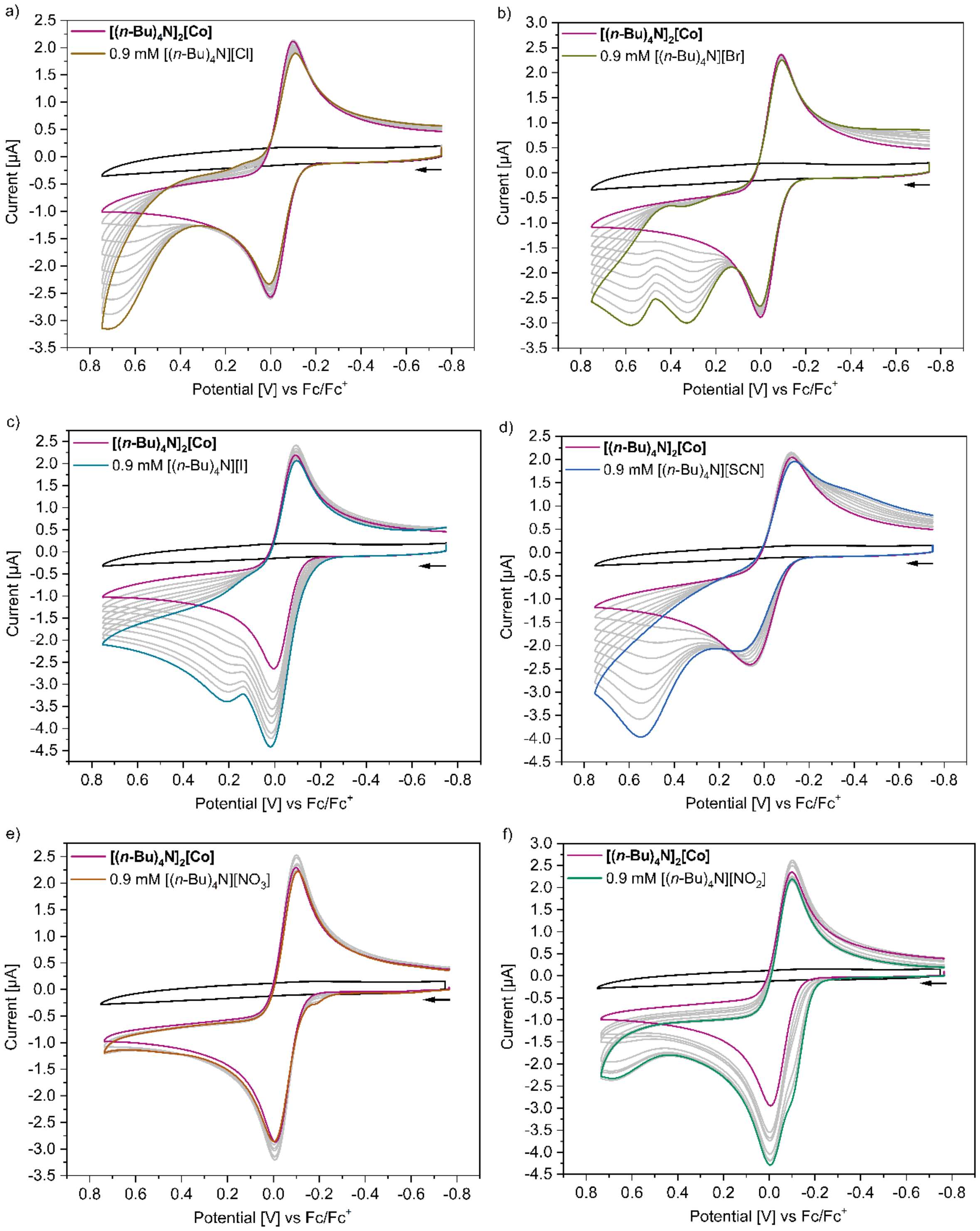

Figure S58: Cyclic voltammograms of **[(*n*-Bu)$_4$N]$_2$[Co]** (1.0 mM in 1,2-DFB), upon titration with (a) [(*n*-Bu)$_4$N][Cl], (b) [(*n*-Bu)$_4$N][Br], (c) [(*n*-Bu)$_4$N][I], (d) [(*n*-Bu)$_4$N][SCN], (e) [(*n*-Bu)$_4$N][NO_3], and (f) [(*n*-Bu)$_4$N][NO_2] (12.5 mM in 1,2-DFB). The initial CV is shown in magenta, intermediate CVs (0.125 equiv additions) in gray and the final CV (after addition of 1.0 equiv [(*n*-Bu)$_4$N][X]; final concentration: 925 µM) in various colors. All scans were recorded at a scan rate of 100 mV/s and are shown alongside the background (black) of pure supporting electrolyte.

0.10 M [(n-Bu)$_4$N][PF_6] supporting electrolyte in 1,2-DFB, r.t., glassy carbon working electrode, referenced to Fc/Fc^+.

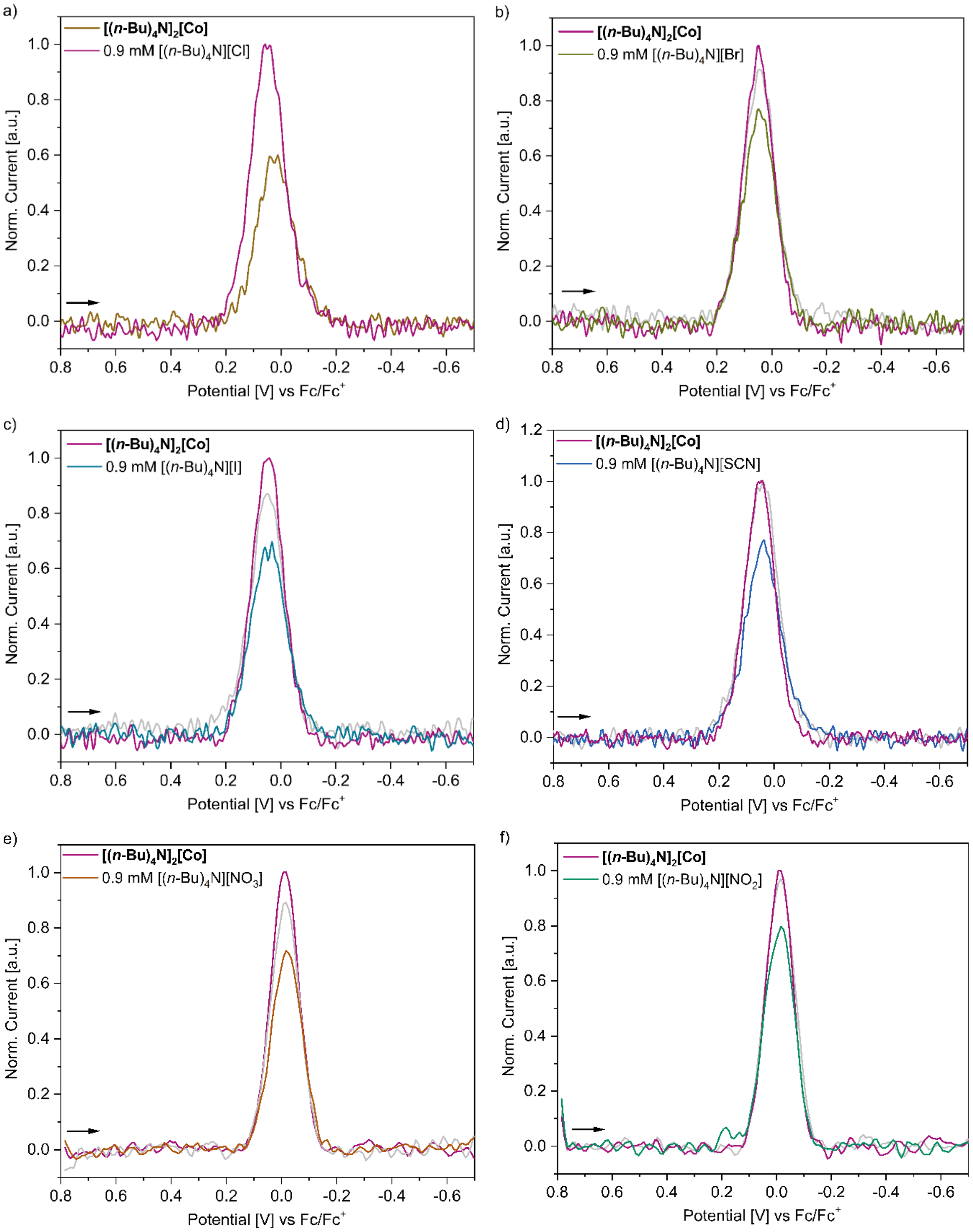

Figure S59: Square wave voltammogram of **[(*n*-Bu)$_4$N]$_2$[Co]** before (magenta; 1.0 mM in 1,2-DFB) and after addition of 0.5 equiv (gray) and 1.0 equiv (various colors) of (a) [(*n*-Bu)$_4$N][Cl], (b) [(*n*-Bu)$_4$N][Br], (c) [(*n*-Bu)$_4$N][I], (d) [(*n*-Bu)$_4$N][SCN], (e) [(*n*-Bu)$_4$N][NO_3], and (f) [(*n*-Bu)$_4$N][NO_2] (12.5 mM in 1,2-DFB; final concentration: 925 µM). 0.10 M [(*n*-Bu)$_4$N][PF_6] supporting electrolyte in 1,2-DFB, r.t., glassy carbon working electrode, referenced to Fc/Fc$^+$.

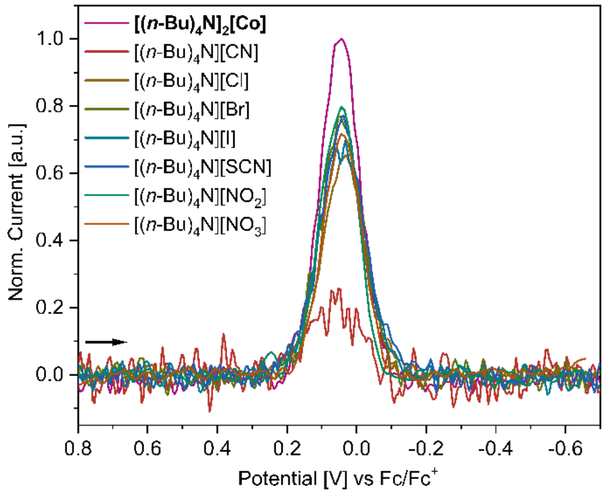

Figure S60: Comparison of square wave voltammograms of **[(*n*-Bu)$_4$N]$_2$[Co]** (magenta) after addition of 1.0 equiv of [(*n*-Bu)$_4$N][CN] (red), [(*n*-Bu)$_4$N][Cl] (bronze), [(*n*-Bu)$_4$N][Br] (green), [(*n*-Bu)$_4$N][I] (petrol), [(*n*-Bu)$_4$N][SCN] (blue), [(*n*-Bu)$_4$N][NO$_3$] (forest green) and [(*n*-Bu)$_4$N][NO$_2$] (orange). For conditions, see Figure S59.

4.5 Selectivity studies: **SWCNT-[Pyr]$_2$[Co]**

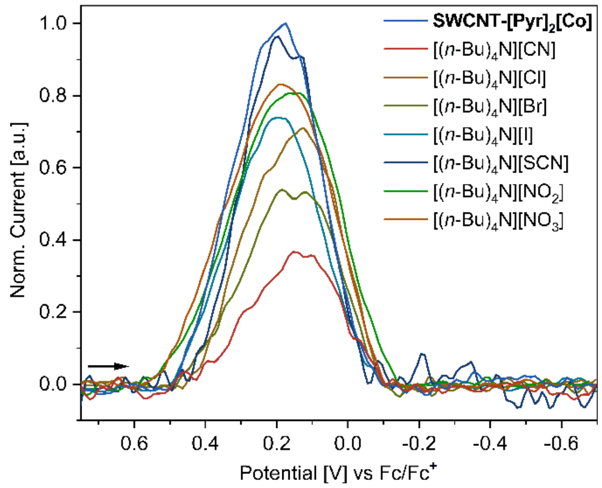

Figure S61: Comparison of square wave voltammograms of **SWCNT-[Pyr]$_2$[Co]** (blue) after addition of 10.0 µmol (25.0 mM [(*n*-Bu)$_4$N][X] in 1,2-DFB; final concentration: 1.85 mM) of [(*n*-Bu)$_4$N][CN] (red), [(*n*-Bu)$_4$N][Cl] (bronze), [(*n*-Bu)$_4$N][Br] (green), [(*n*-Bu)$_4$N][I] (petrol), [(*n*-Bu)$_4$N][SCN] (blue), [(*n*-Bu)$_4$N][NO$_3$] (forest green) and [(*n*-Bu)$_4$N][NO$_2$] (orange). 0.10 M [(*n*-Bu)$_4$N][PF$_6$] supporting electrolyte in 1,2-DFB, r.t., **SWCNT-[Pyr]$_2$[Co]** dispersion drop-cast on glassy carbon working electrode, referenced to Fc/Fc$^+$.

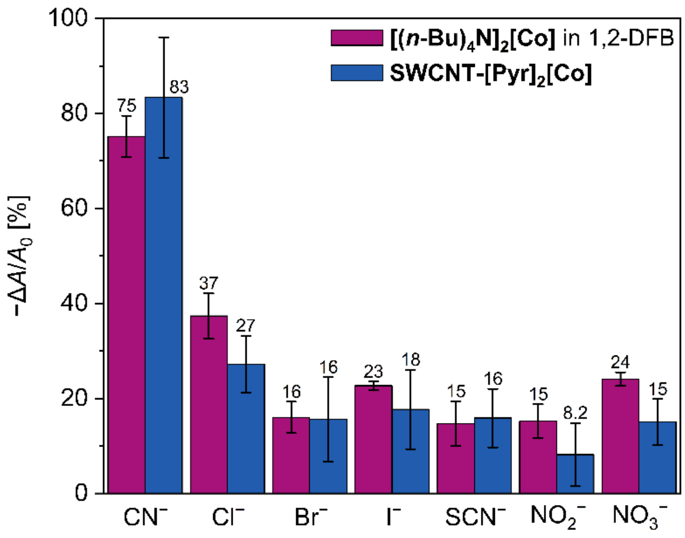

Figure S62: Comparison of normalized sensing response of **[Pyr]$_2$[Co]** and **SWCNT-[Pyr]$_2$[Co]** to the exposure of various [(*n*-Bu)$_4$N] salts (**[Pyr]$_2$[Co]**: 5.00 µmol [(*n*-Bu)$_4$N][X]; **SWCNT-[Pyr]$_2$[Co]**: 10.0 µmol [(*n*-Bu)$_4$N][X]). Error bars represent standard errors (N = 2).

4.6 Theoretical Limit of Detection (LOD) Determination

The theoretical LOD (Table S3) was determined using modified literature procedures.[23,24] For both **[(*n*-Bu)$_4$N]$_2$[Co]** and **SWCNT-[Pyr]$_2$[Co]**, the baseline noise in the SWV studies was determined by fitting the baseline signal between 0.0 and −0.6 V vs Fc/Fc^+ (free of faradaic features) with a fifth-order polynomial (50 data points). The root-mean square noise (rms_{noise}) of the baseline was calculated from this fit as:

$$V_{X^2} = \sum (y_i - y_{Fit})^2$$

$$rms_{noise} = \sqrt{\frac{V_{X^2}}{N}}$$

where:

- y_i: experimentally determined baseline data point
- y_{Fit}: corresponding *y*-value from polynomial fit
- N: number of fitted data points (here $N = 50$).

Following the literature definition of a true signal (signal-to-noise ratio, SNR > 3), the LOD was calculated according to:

$$LOD\ [\mu M] = 3 \times \frac{rms_{noise}}{m}$$

where:

- m: the slope of the linear fit of the in SWV peak-area inhibition [$-\Delta A/A_0$ (%) = $(A_0 - A)/A_0 \times 100\%$; A_0 = uninhibited SWV peak area] versus cyanide concentration (Figure S63).

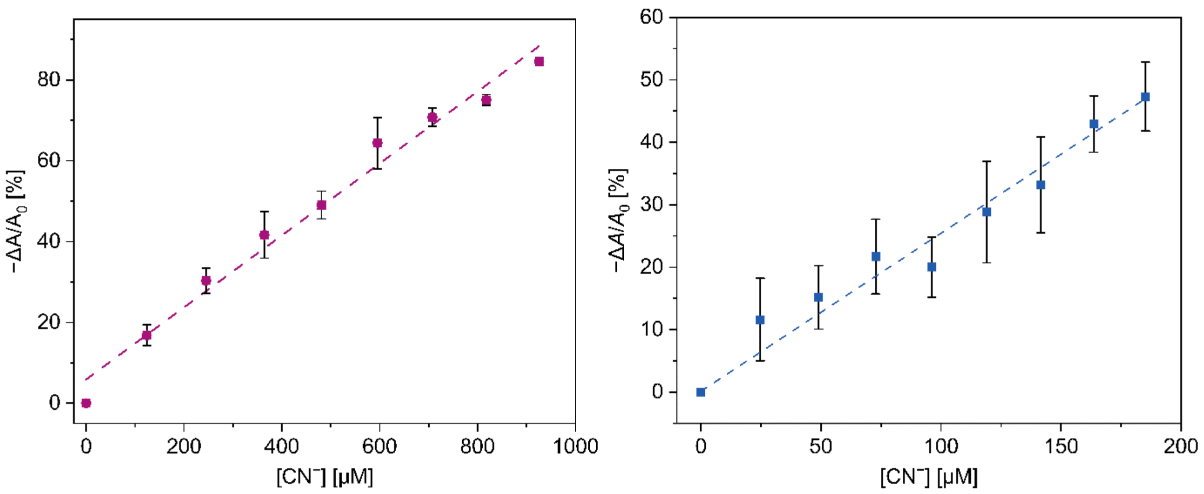

Figure S63: Electrochemical sensing response of **[(*n*-Bu)$_4$N]$_2$[Co]** (left) and **SWCNT-[Pyr]$_2$[Co]** (right) to varying concentrations of [(*n*-Bu)$_4$N][CN], shown as a scatter plots with linear fits (dashed lines) for LOD determination. Error bars represent standard errors (N = 2).

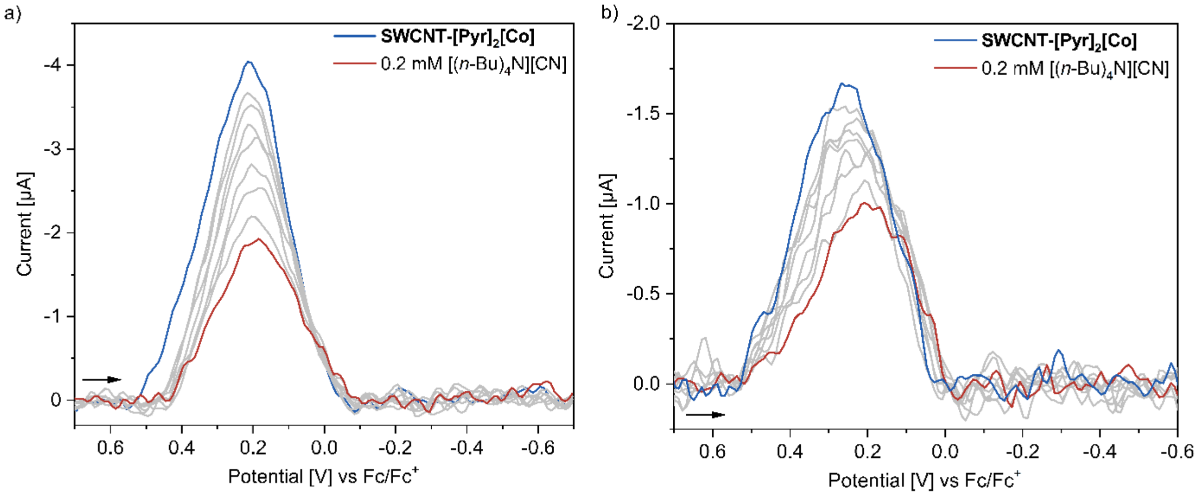

Figure S64: Comparison of the square wave voltammograms collected on two individual **SWCNT-[Pyr]$_2$[Co]** electrodes after titration with [(*n*-Bu)$_4$N][CN] (2.5 mM in 1,2-DFB). The initial SWV is shown in blue, intermediate SWVs (0.125 equiv additions) in gray, and the final SWV (after addition of 1.00 µmol; final concentration: 185 µM) in red. 0.10 M [(*n*-Bu)$_4$N][PF$_6$] supporting electrolyte in 1,2-DFB, r.t., glassy carbon working electrode, referenced to Fc/Fc$^+$.

Table S3: Values obtained in the LOD calculation of **[(*n*-Bu)$_4$N]$_2$[Co]** and **SWCNT-[Pyr]$_2$[Co]**.

**Compound**	***rms*$_{noise}$**	***m***	**LOD [µM]**
**[(*n*-Bu)$_4$N]$_2$[Co]**	0.1276	0.0891	4.296
**SWCNT-[Pyr]$_2$[Co]**	0.0702	0.2348	0.8964

5 Optimization and Control Experiments for Electrochemical Sensing Studies

5.1 Reproducibility of Drop-Casting Technique

To assess the reproducibility of the **SWCNT-[pyr]$_2$[Co]** electrode preparation, the drop-casting procedure was evaluated both within one dispersion and across individually prepared dispersions. Within a single dispersion, nearly identical SWV responses were obtained from several electrodes, indicating consistent thin-film formation (Figure S65).

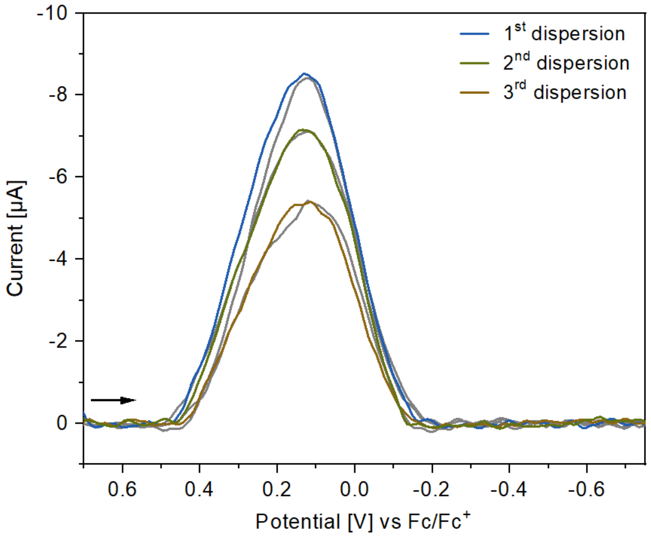

Figure S65: Comparison of three separately prepared dispersions of **SWCNT-[Pyr]$_2$[Co]** for SWV measurements. The dark grey SWV traces correspond to a second electrode prepared from the respective identical dispersion. 0.10 M [(*n*-Bu)$_4$N][PF$_6$] supporting electrolyte in 1,2-DFB, r.t., glassy carbon working electrode, referenced to Fc/Fc$^+$.

Across different dispersions, the absolute SWV peak currents varied moderately (−4 to −8 µA). To ensure reliable comparison of data across electrodes and experiments, all results used for limit-of-detection (LOD) calculations, selectivity studies and all comparisons between the composite material and the molecular analogue were normalized to the uninhibited peak area and expressed as the percent inhibition of the SWV peak area (see Section 4.6). This approach accounts for sample-to-sample peak current variations.

Additionally, the effect of thin-film thickness and surface coverage was examined. Increasing the amount of deposited **SWCNT-[Pyr]$_2$[Co]** led to a broadening of the anodic feature and a larger CNT background current (Figure S66), consistent with thicker films. During initial optimization studies, two-fold deposition was determined to yield the most consistent results with respect to complex-to-CNT signal ratio, feature width and absolute currents. Despite the current variations across electrodes, the half-wave potential ($E_{1/2}$) remained constant, confirming that redox behavior and film integrity are maintained.

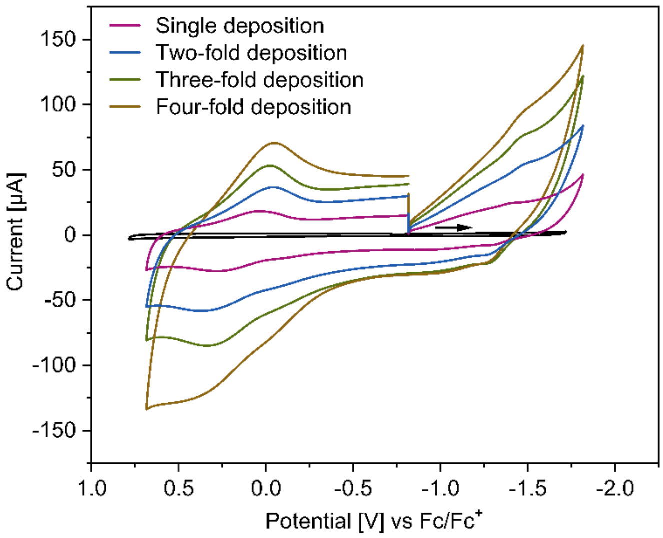

Figure S66: Cyclic voltammogram of **SWCNT-[Pyr]$_2$[Co]** thin films on individual glassy carbon working electrodes with a single (purple), double (blue), triple (green) and four-fold (bronze) deposition of the composite. All scans were recorded at a scan rate of 100 mV/s and are shown alongside the background (black) of pure supporting electrolyte. 0.10 M [(*n*-Bu)$_4$N][PF$_6$] supporting electrolyte in 1,2-DFB, r.t., referenced to Fc/Fc$^+$.

5.2 Robustness of **SWCNT-[Pyr]$_2$[Co]** Surface Functionalization under Electrochemical Cycling Conditions

The robustness of the **SWCNT-[Pyr]$_2$[Co]** surface functionalization was evaluated under repeated electrochemical cycling before and after soaking the **SWCNT-[Pyr]$_2$[Co]** electrodes in 1,2-DFB,the solvent employed for all electrochemical measurements. As shown in Figure S67, the **SWCNT-[Pyr]$_2$[Co]** film retained identical electrochemical features after soaking for 1 h, and no visible color change of the solution was observed. These results confirm that **SWCNT-[Pyr]$_2$[Co]** is highly robust in 1,2-DFB, with no detectable desorption or leaching upon solvent contact or during electrochemical operation.

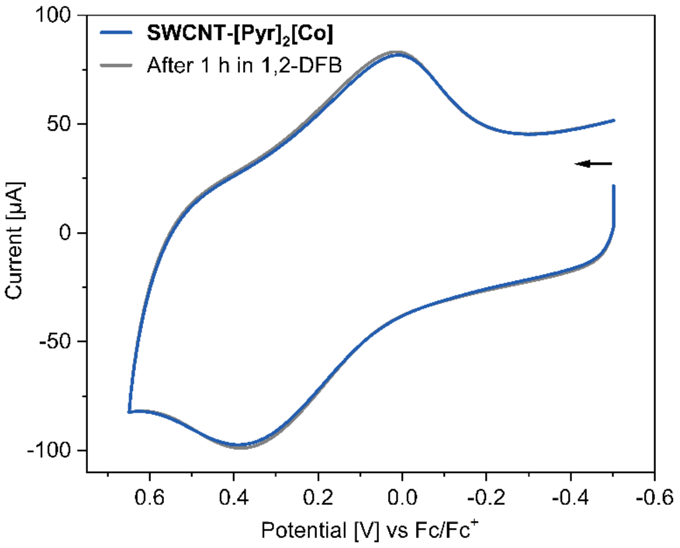

Figure S67: Cyclic voltammogram of a **SWCNT-[Pyr]$_2$[Co]** thin film in 1,2-DFB before (blue) and after (grey) 1 h of soaking. Both scans were recorded at a scan rate of 100 mV/s. 0.10 M [(n-Bu)$_4$N][PF$_6$] supporting electrolyte, r.t., referenced to Fc/Fc$^+$.

5.3 Response of **SWCNT-[Pyr]$_2$[Co]** Electrodes to Sequential and Prolonged Cyanide Exposure

To verify that the observed signal attenuation during titration originates from cyanide concentration rather than electrode degradation or repeated scanning, the stability of **SWCNT-[Pyr]$_2$[Co]** toward prolonged exposure to [(n-Bu)$_4$N][CN] was evaluated.

In the first experiment, a single **SWCNT-[Pyr]$_2$[Co]** electrode was subjected to four sequential additions of [(*n*-Bu)$_4$N][CN] while monitoring its square wave voltammetry (SWV) response (Figure S68). Each addition produced a gradual decrease in the net SWV peak current, consistent with the expected concentration-dependent inhibition of the redox process. After each cyanide addition, a second SWV scan was recorded at the same concentration to test whether further signal loss occurred in the absence of additional analyte. These repeated scans showed only minor changes in SWV peak area, all within the standard deviation determined from the limit-of-detection (LOD) studies (see Section 4.6), confirming that the inhibition response is governed by cyanide concentration and not by time- or scan-dependent degradation of the electrode.

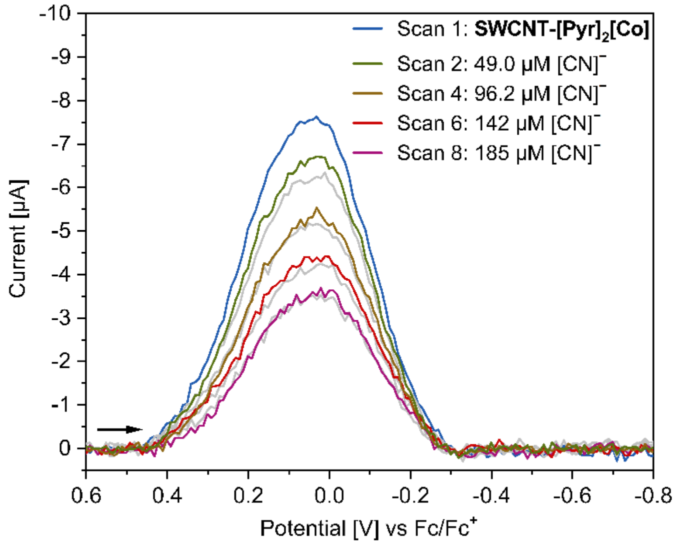

Figure S68: Square wave voltammograms of **SWCNT-[Pyr]$_2$[Co]** after four sequential additions of [(*n*-Bu)$_4$N][CN] (2.5 mM in 1,2-DFB). The first SWV scan in pure electrolyte is shown in blue; sequential [(*n*-Bu)$_4$N][CN] additions (+0.250 µmol each) are shown in green, bronze, red, and purple. The gray traces correspond to the second SWV scan recorded at each respective [(*n*-Bu)$_4$N][CN] concentration (scans 3, 5, 7, and 9). 0.10 M [(*n*-Bu)$_4$N][PF$_6$] supporting electrolyte in 1,2-DFB, r.t., glassy carbon working electrode, referenced to Fc/Fc$^+$.

In a complementary control, four independently prepared **SWCNT-[Pyr]$_2$[Co]** electrodes were each exposed directly to a defined concentration of [(*n*-Bu)$_4$N][CN] (Figure S69). The corresponding peak area inhibitions ($-\Delta A/A_0$) of 15%, 25%, 40%, and 49% for 0.250, 0.500, 0.750, and 1.00 µmol [(*n*-Bu)$_4$N][PF$_6$], respectively, closely matched those obtained in the sequential titration.

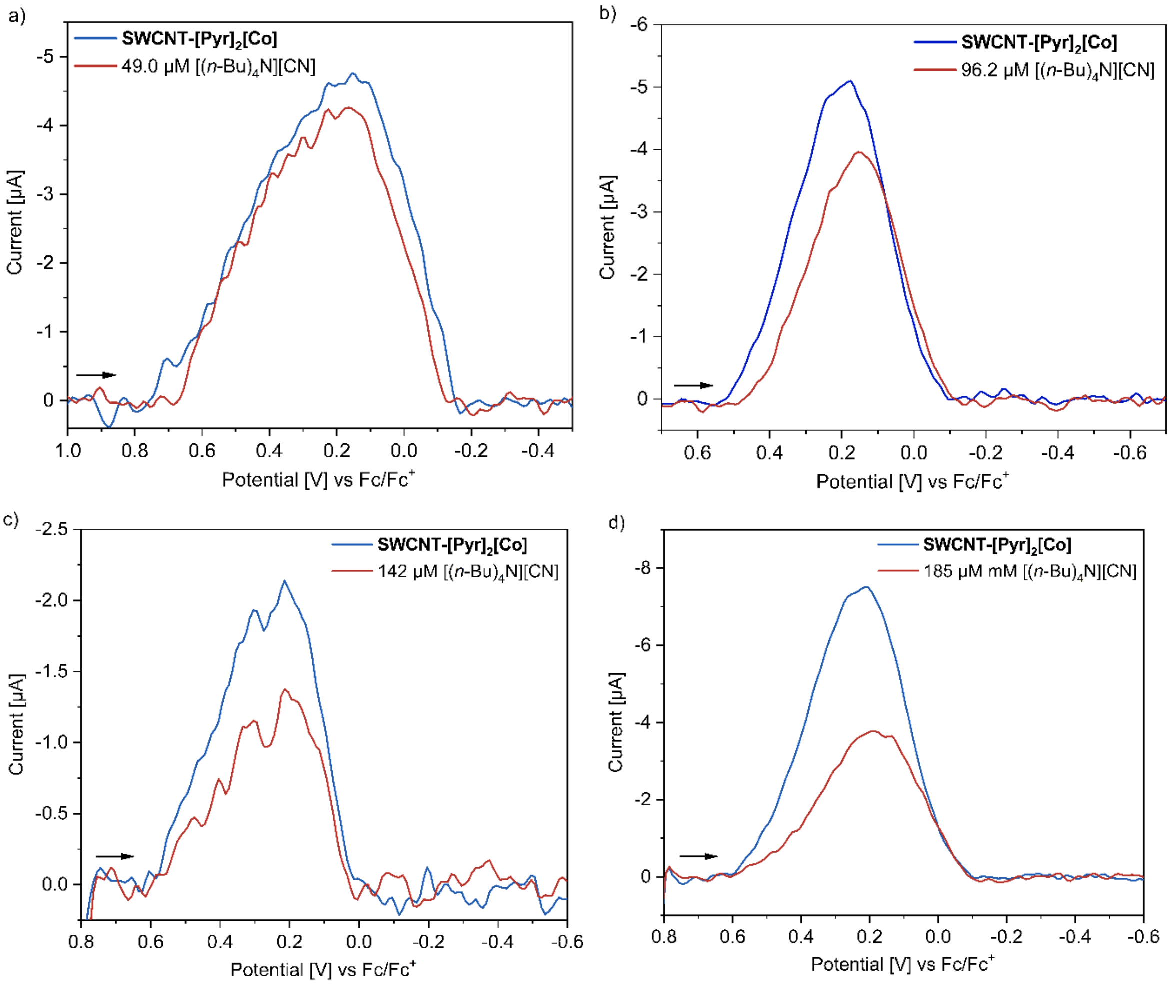

Figure S69: Square wave voltammograms of four individually prepared **SWCNT-[Pyr]$_2$[Co]** electrodes after direct addition of (a) 0.250 µmol, (b) 0.500 µmol, (c) 0.750 µmol and (d) 1.00 µmol [(*n*-Bu)$_4$N][CN]. The initial SWV is shown in blue; the SWV after [(*n*-Bu)$_4$N][CN] addition is shown in red. The relative SWV peak area inhibitions ($-\Delta A/A_0$) correspond to (a) 15%, (b) 25%, (c) 40% and (d) 49%. 0.10 M [(*n*-Bu)$_4$N][PF$_6$] supporting electrolyte in 1,2-DFB, r.t., glassy carbon working electrode, referenced to Fc/Fc$^+$.

5.4 Attempted Cyanide Sensing with **SWCNT-[Pyr]$_3$[Co(CN)$_6$]**

To test whether a closed-shell cobalt complex lacking a semiquinone radical (**[Co(CN)$_6$]**$^{3-}$) could exhibit a cyanide-dependent electrochemical response, **SWCNT-[Pyr]$_3$[Co(CN)$_6$]** electrodes were prepared and analyzed under conditions identical to those used for **SWCNT-[Pyr]$_2$[Co]**. Upon sequential addition of [(n-Bu)$_4$N][CN], no changes in SWV profiles were detected.

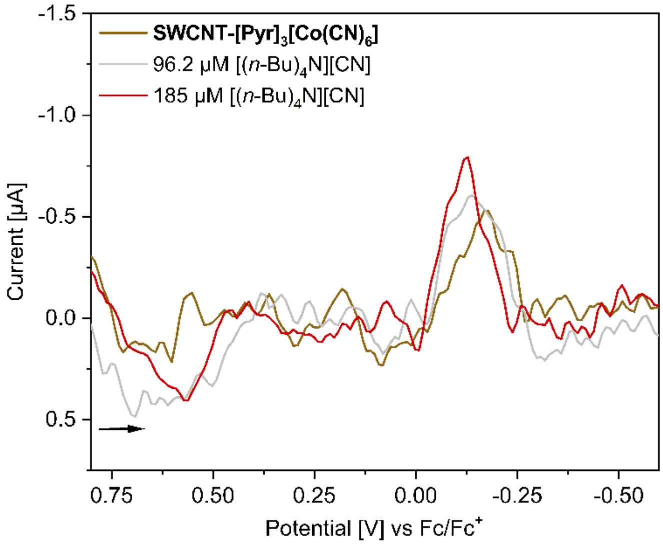

Figure S70: Square-wave voltammograms of **SWCNT-[Pyr]$_3$[Co(CN)]** after two sequential additions of [(*n*-Bu)$_4$N][CN]. The initial SWV in pure electrolyte is shown in bronze; sequential cyanide additions are shown in gray and red. 0.10 M [(*n*-Bu)$_4$N][PF$_6$] supporting electrolyte in 1,2-DFB, r.t., glassy carbon working electrode, referenced to Fc/Fc$^+$.

6 Chemical Oxidation of [Pyr]₂[Co]

6.1 Chemical Synthesis of **[Co]⁻**

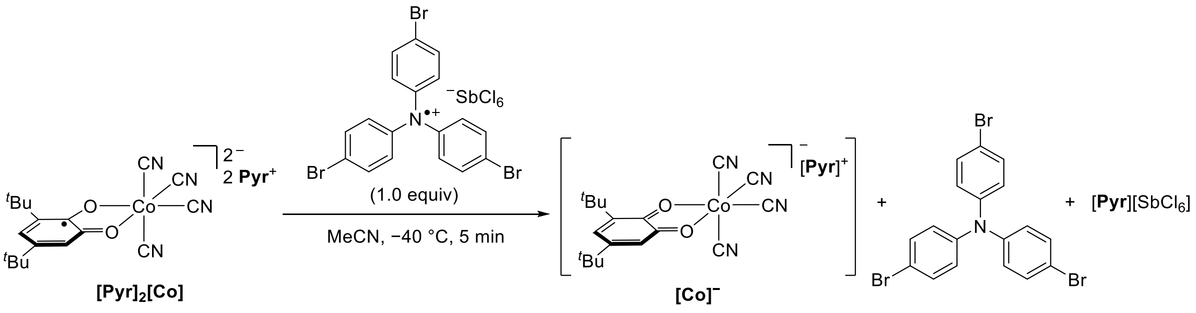

Scheme S1: Chemical synthesis of **[Co]⁻** with proposed reaction products.

To examine the cyanide sensing mechanism of **[Pyr]₂[Co],** several stoichiometric experiments were conducted with [(*n*-Bu)$_4$N][CN] under oxidizing conditions and were analyzed by IR and NMR spectroscopy.

In an argon-filled glovebox, a 10 mL scintillation vial was charged with a magnetic stir bar, **[Pyr]₂[Co]** (10.0 mg, 10.4 µmol, 1.00 equiv) and MeCN (3.0 mL) to produce a suspension. The vial was placed into a cooling well cooled to −40 °C. In the dark, under stirring, a solution of tris(4-bromophenyl)ammoniumyl hexachloroantimonate ("*Magic Blue*", 8.49 mg, 10.4 µmol, 1.00 equiv) dissolved in MeCN (2.0 mL) was added, leading to an immediate color change to deep purple with the concomitant dissolution of the cobalt complex. The mixture was allowed to stir for 5 min at −40 °C, before the solvent was removed *in vacuo* at low temperatures. The purple-brown residue was analyzed by IR spectroscopy (Figure S71) under inert conditions.

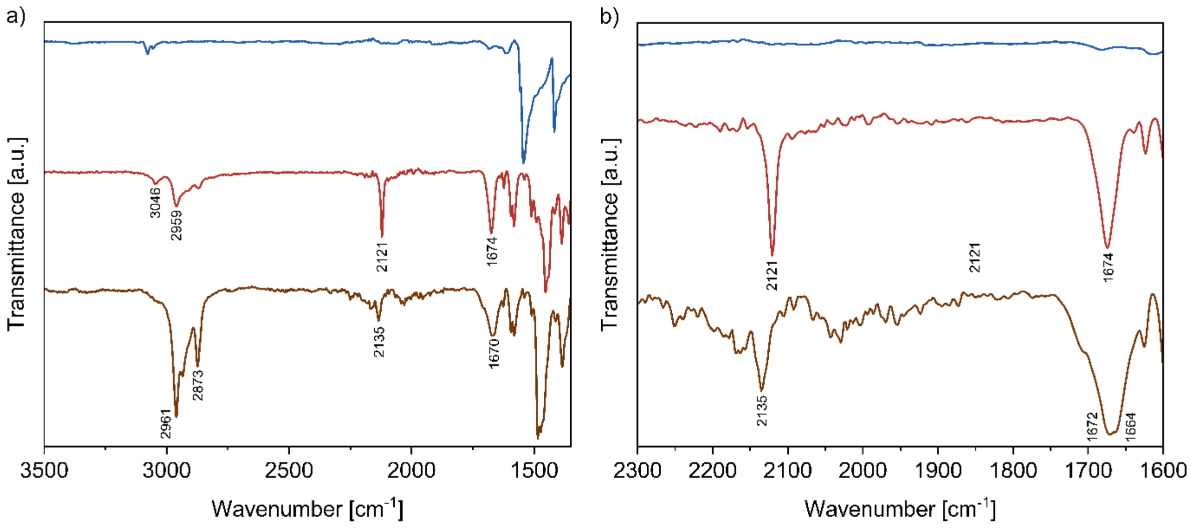

Figure S71: a) IR (ATR) spectra of the dried product mixture (brown) obtained after reaction of **[Pyr]₂[Co]** (red) with "*Magic Blue*" (blue). b) Zoomed IR (ATR) spectra highlighting the cyanide and carbonyl regions.

Attempts to reconstitute the precipitated solid into NMR solvents for further analysis categorically led to an immediate color change to yellow and the sole detection of decomposition products. Despite the pronounced reactivity of **[Co]**$^-$ and its elusive nature, the observed changes in the cyanide IR feature of the isolated solid, as well as the detection of stoichiometric amounts of tris(4-bromophenyl)amine in the ^{1}H NMR spectrum of the reaction mixture support the successful oxidation of **[Pyr]$_2$[Co]** with "*Magic Blue*".

6.2 Reaction of Chemically Generated **[Co]**$^-$ with [(n-Bu)$_4$N][CN]

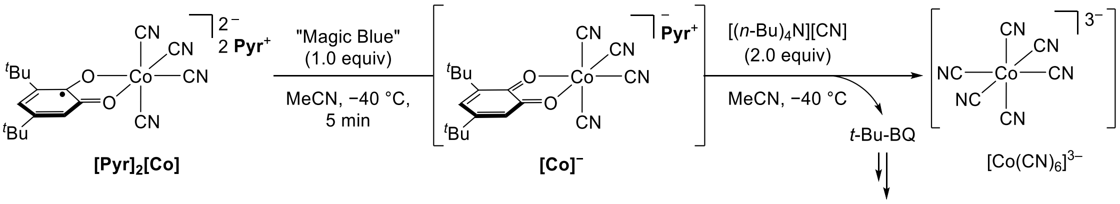

Scheme S2: Reaction of chemically generated **[Co]**$^-$ with [(n-Bu)$_4$N][CN]. "*Magic Blue*": Tris(4-bromophenyl)ammoniumyl hexachloroantimonate.

In an argon-filled glovebox, a 10 mL scintillation vial was charged with a magnetic stir bar, **[Pyr]$_2$[Co]** (10.0 mg, 10.4 µmol, 1.00 equiv) and MeCN (3.0 mL) to produce a suspension. The vial was placed into a cooling well cooled to −40 °C. In the dark, under stirring, a solution of "*Magic Blue*" (8.49 mg, 10.4 µmol, 1.00 equiv) dissolved in MeCN (2.0 mL) was added, leading to an immediate color change to deep purple with concomitant dissolution of the cobalt complex. The mixture was allowed to stir for 5 min at −40 °C, before a solution of [(n-Bu)$_4$N][CN] (5.60 mg, 20.8 µmol, 2.00 equiv) in MeCN (1.0 mL) was added, resulting in an immediate solution color change from deep purple to bright yellow. The solvent was removed *in vacuo* and the yellow-orange residue was analyzed by IR spectroscopy under inert conditions (Figure S72). MeCN-d_3 (0.7 mL) was then added to the vial and the solution was filtered over Celite® into a J. Young NMR tube. Upon addition of the deuterated solvent, the solution's color quickly changed from yellow to green-brown, with a green solid precipitating. The green-brown supernatant was analyzed by ^{1}H NMR spectroscopy (Figure S73).

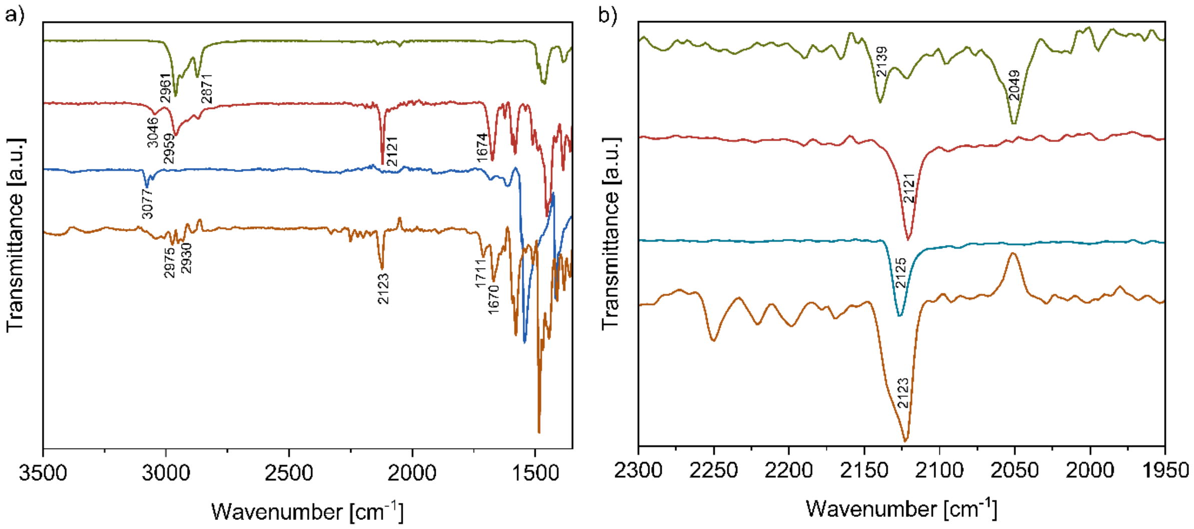

Figure S72: (a) IR (ATR) spectra of the dried product mixture (orange) obtained after reaction of **[Pyr]₂[Co]** (red) with Magic Blue (blue) and subsequent addition of 2.0 equiv of $[(n\text{-Bu})_4N][CN]$ (green). (b) IR (ATR) spectra of the dried product mixture (orange), **[Pyr]₂[Co]** (red), $[(n\text{-Bu})_4N][CN]$ (green) and $K_3[Co(CN)_6]$ (petrol) for comparison, highlighting the cyanide region.

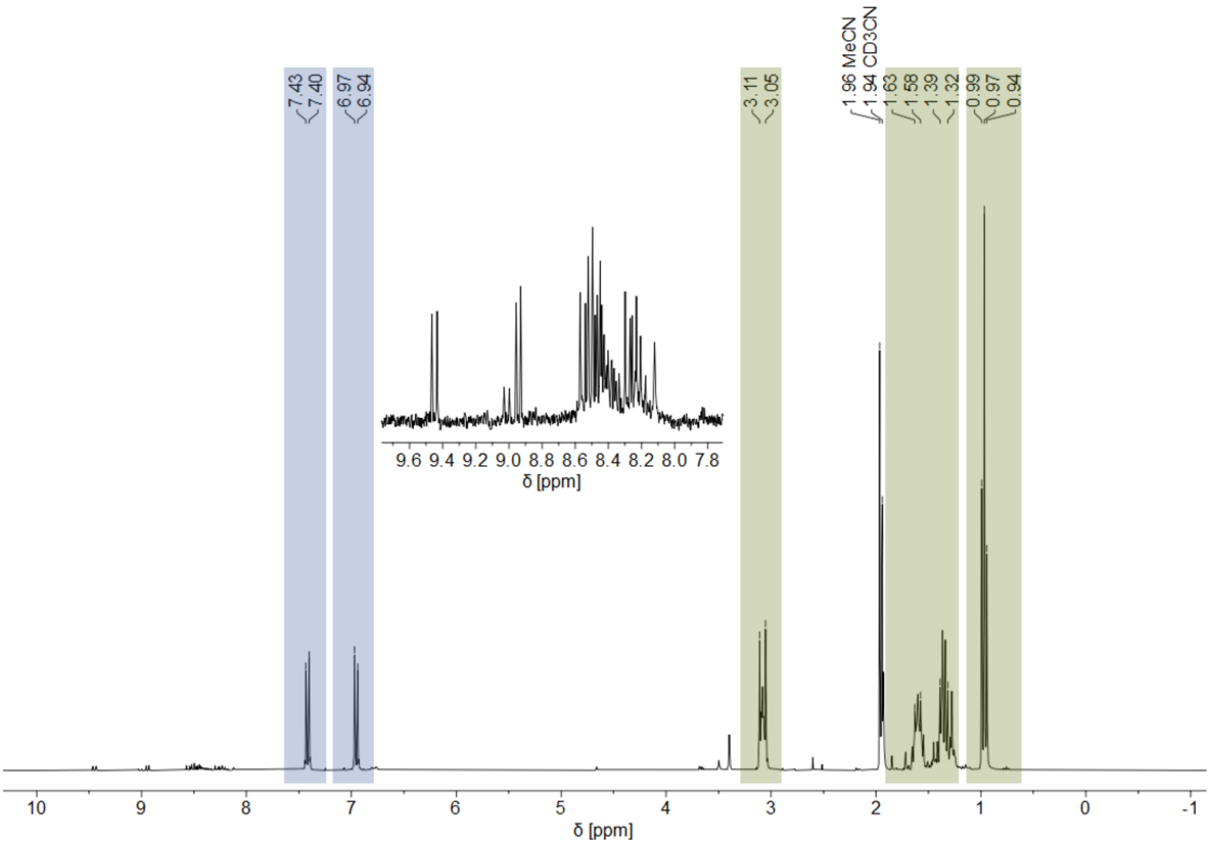

Figure S73: ^{1}H NMR (300 MHz, MeCN-d_3, 298 K) spectrum of the product mixture after reaction of **$[Pyr]_2[Co]$** with Magic Blue and subsequent addition of $[(n\text{-Bu})_4N][CN]$ (green), showing tris(4-bromophenyl)amine (blue) and decomposition products of **$[Pyr]^+$**.

Given that the ^{1}H NMR spectrum of the reaction mixture showed an apparent decomposition of the **[Pyr]⁺** cation (Figure S73), as well as of the previously isolatable, free *t*-Bu-BQ ligand, comparative NMR samples containing equivalent amounts of

I. **[Pyr]$_2$[Co]** and [(*n*-Bu)$_4$N][CN],

II. **[Pyr][Br]** and [(*n*-Bu)$_4$N][CN],

III. *t*-Bu-BQ and **[Pyr][Br]** (Figure S74a),

IV. *t*-Bu-BQ and [(*n*-Bu)$_4$N][CN] (Figure S74b),

V. and *t*-Bu-BQ, [(*n*-Bu)$_4$N][CN], and **[Pyr][Br]** (Figure S74c),

were prepared, to determine the crucial reaction partners in these decomposition processes. The addition of 1.0 equiv of [(*n*-Bu)$_4$N][CN] to *t*-Bu-BQ (Figure S74b) led to the complete decomposition of the free ligand, and partial decomposition when additional **[Pyr][Br]** was present (Figure S74c).

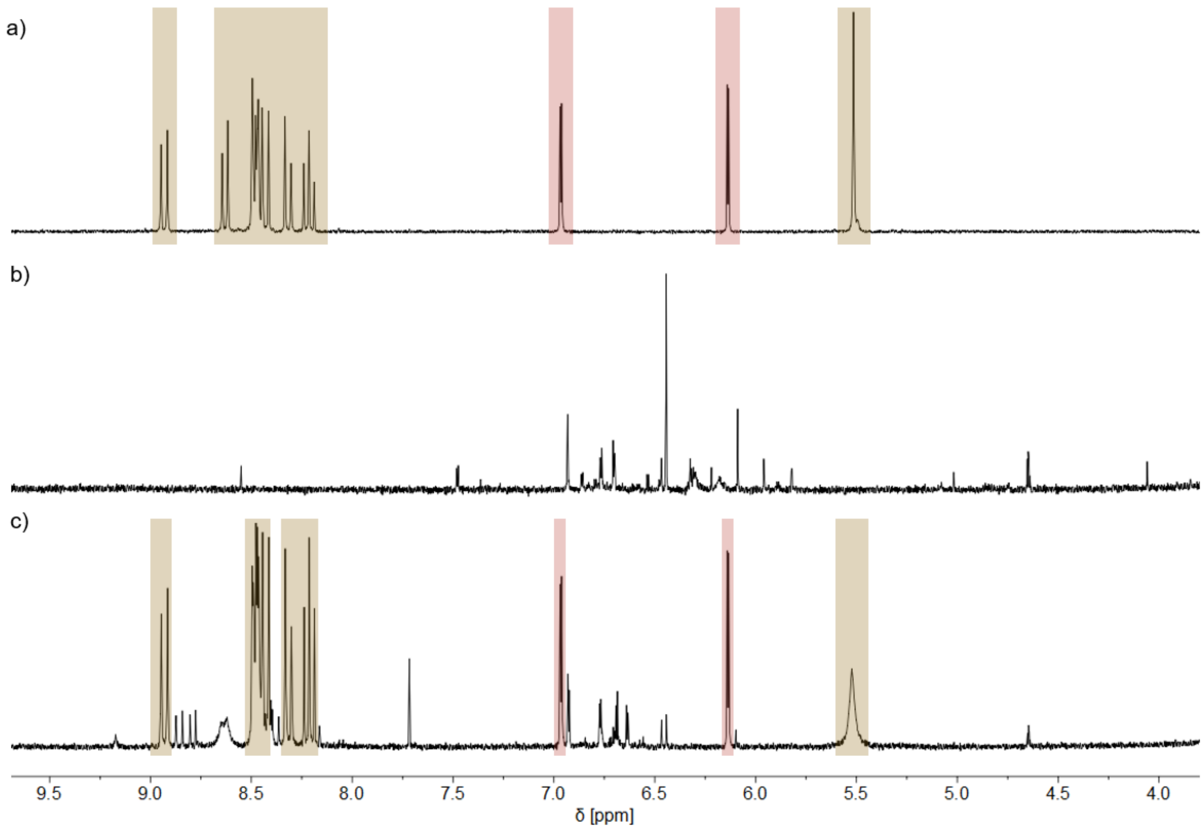

Figure S74: ^{1}H NMR (300 MHz, DMSO-*d*$_6$, 298 K) spectra 1.5 h after the addition of equivalent amounts of (a) *t*-Bu-BQ (red) and **[Pyr][Br]** (bronze), (b) *t*-Bu-BQ and [(*n*-Bu)$_4$N][CN], and (c) *t*-Bu-BQ (red), [(*n*-Bu)$_4$N][CN], and **[Pyr][Br]** (bronze).

These observations are in line with the IR spectroscopic analysis of the reaction products, wherein the recorded IR spectrum only showed few of the characteristic C–H stretching vibrations around 3000 cm^{-1} corresponding to **[Pyr]⁺** and/or *t*-Bu-BQ, but a new CN^{-}

absorption band at 2123 cm^{-1}, which is nearly identical to the one in the $K_3[Co(CN)_6]$ reference (Figure S72b). Therefore it seems plausible that some of the cyanide anions react with the *t*-Bu-BQ ligand, setting-off a cascade of reactions leading to the partial decomposition of **[Pyr]**$^+$, while the $[Co(CN)_4]^-$ moiety reacts with the rest of the CN^-present, forming the hexacyano complex.

Importantly, neither **[Pyr]$_2$[Co]**, nor **[Pyr][Br]** alone showed any reactivity towards [(*n*-Bu)$_4$N][CN], underlining the need for the oxidation of the semiquinone ligand in **[Pyr]$_2$[Co]** to unlock the reactivity towards cyanide seen in the electrochemical sensing studies. The inertness of **[Pyr]$_2$[Co]** towards [(*n*-Bu)$_4$N][CN] was further probed by EPR spectroscopy, confirming that a sample stored under ambient conditions, protected from light, will not show any signs of decomposition over an extended period (Figure S75).

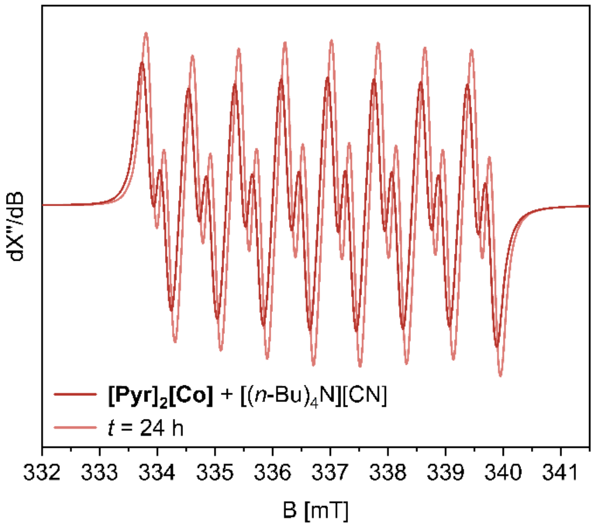

Figure S75: X-band CW EPR spectra of the reaction of **[Pyr]$_2$[Co]** with 1.0 equiv of [(*n*-Bu)$_4$N][CN] (PhMe/MeOH, 3:1), directly after addition (red) and after 24 h (rose).

7 Appendix

7.1 NMR Spectra

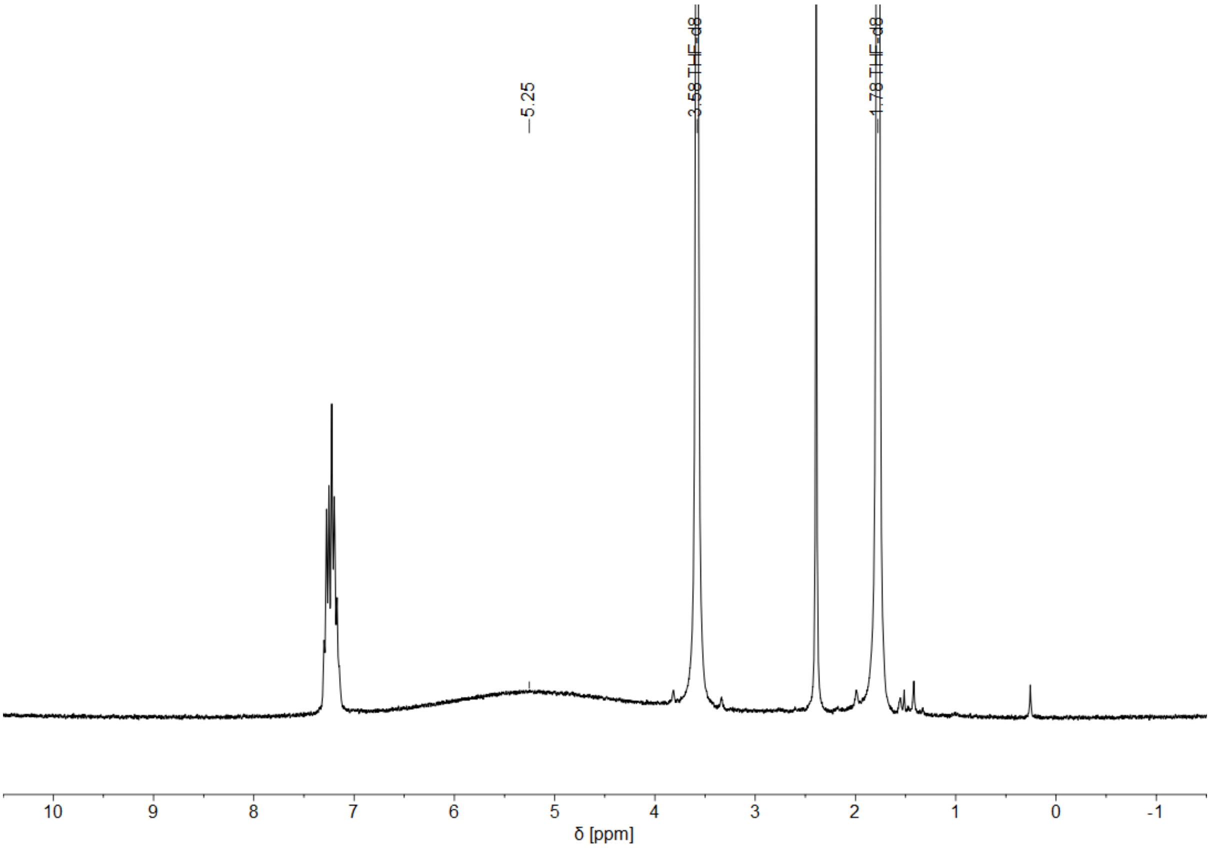

Figure S76 ^{1}H NMR spectrum (300 MHz, THF-d_8, 299 K) of [(t-Bu-SQ)$_2$Co]$_4$.

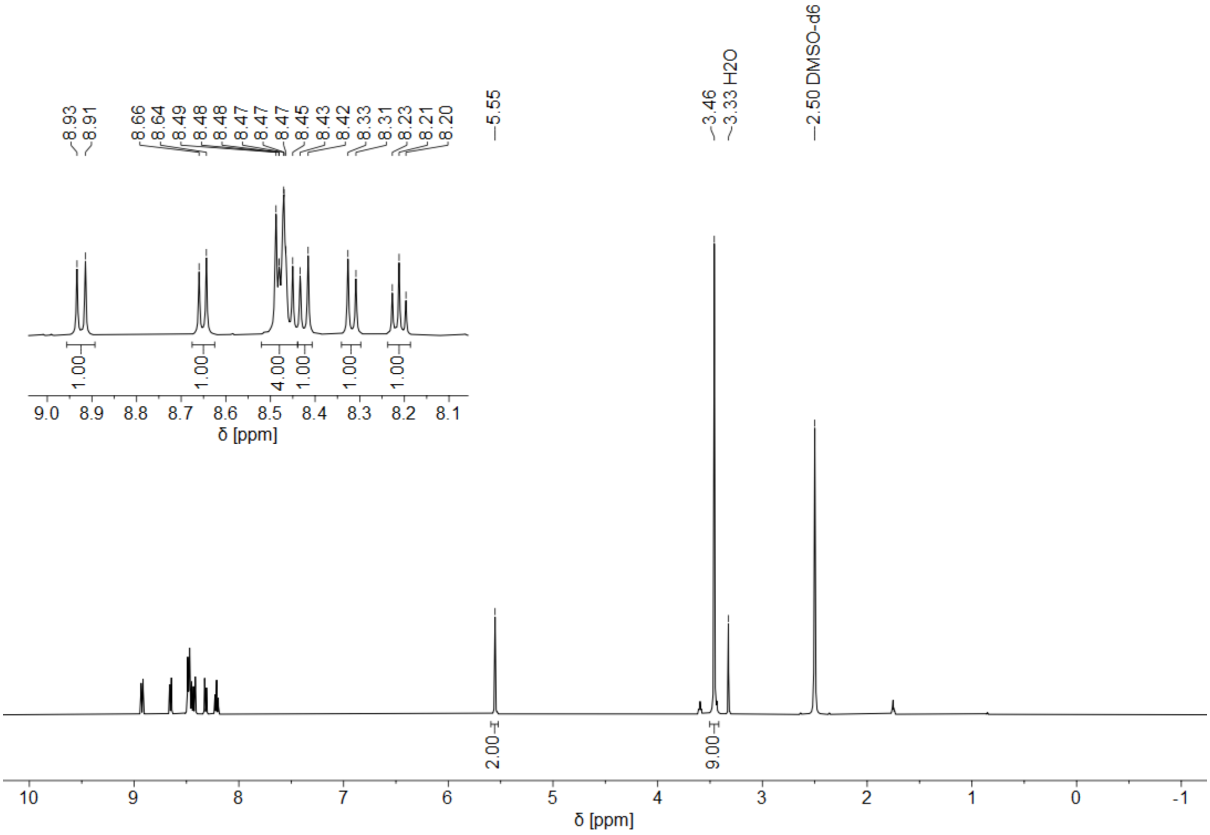

Figure S77: ^{1}H NMR spectrum (500 MHz, DMSO-d_6, 298 K) of pyrene ammonium bromide (**[Pyr][Br]**).

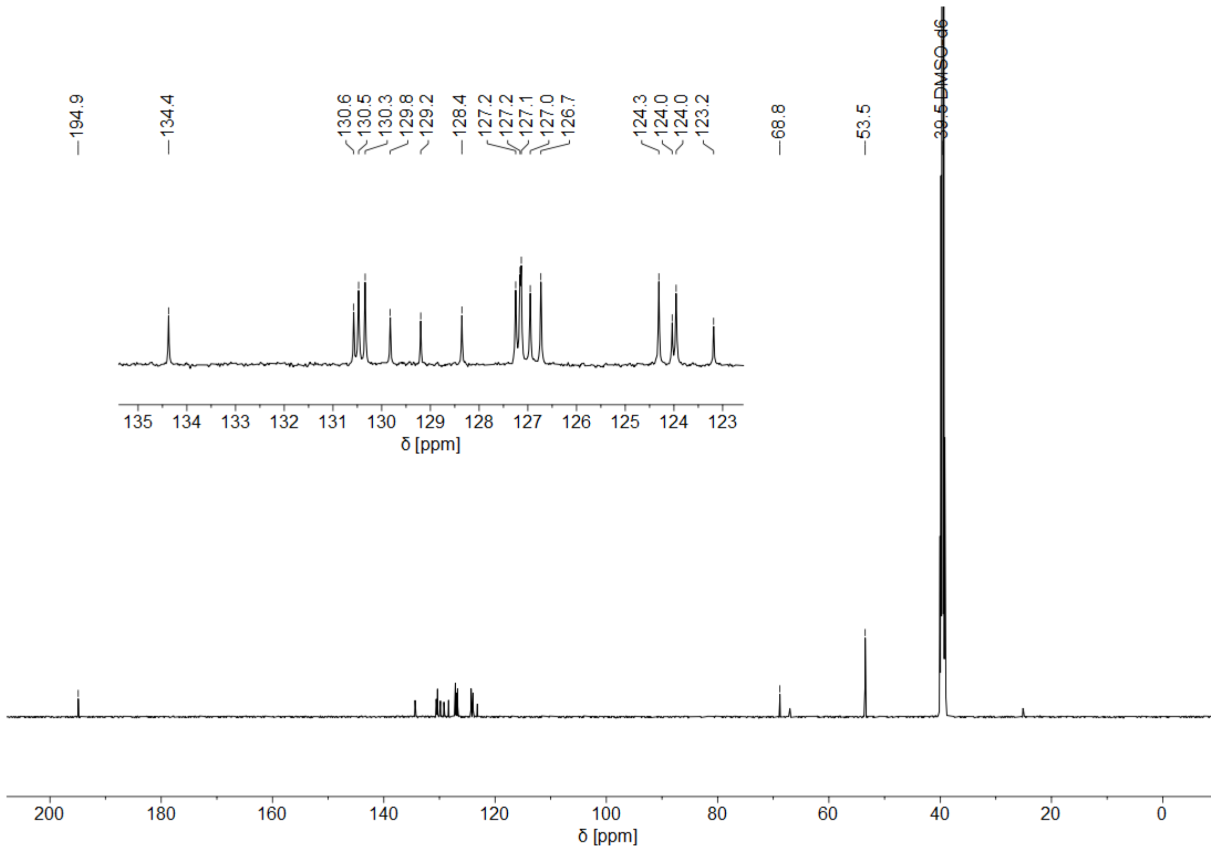

Figure S78: $^{13}C\{^{1}H\}$ NMR spectrum (126 MHz, DMSO-d_6, 298 K) of pyrene ammonium bromide (**[Pyr][Br]**).

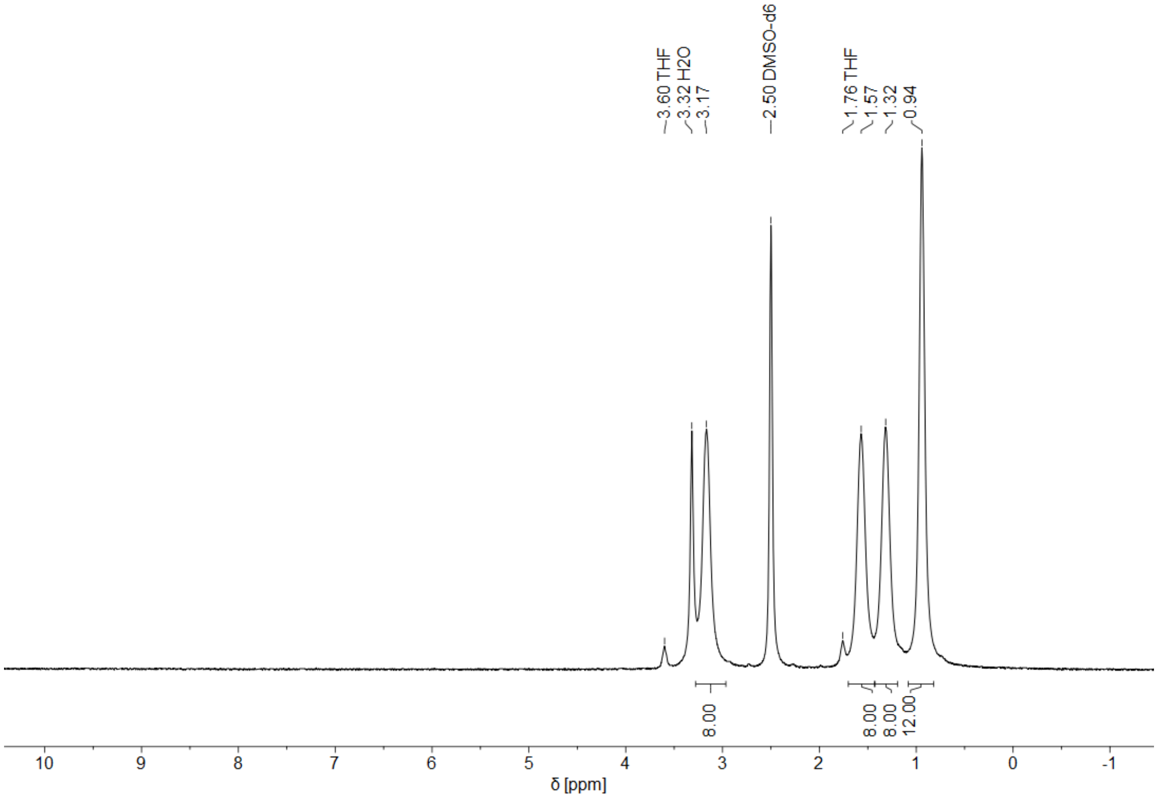

Figure S79: ^{1}H NMR spectrum (300 MHz, DMSO-d_6, 299 K) of **[(*n*-Bu)$_4$N]$_2$[Co]**.

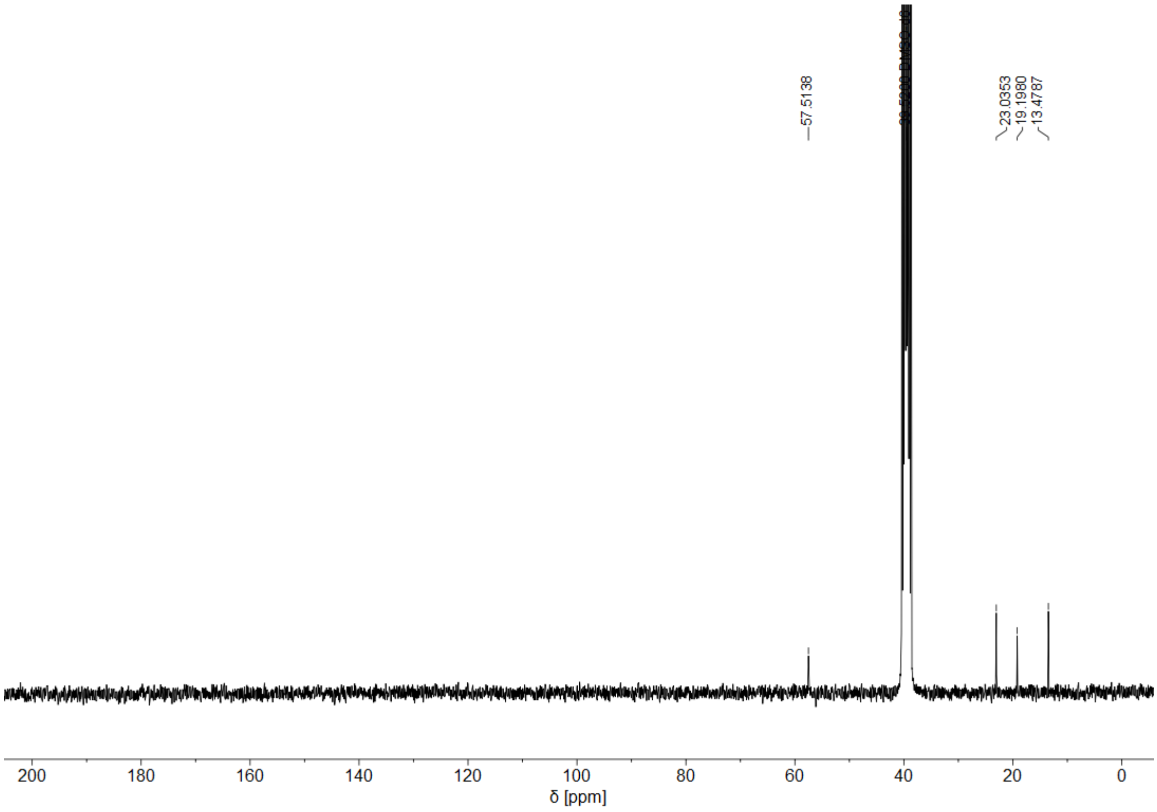

Figure S80: $^{13}C\{^{1}H\}$ NMR spectrum (126 MHz, DMSO-d_6, 298 K) of **[(*n*-Bu)$_4$N]$_2$[Co]**.

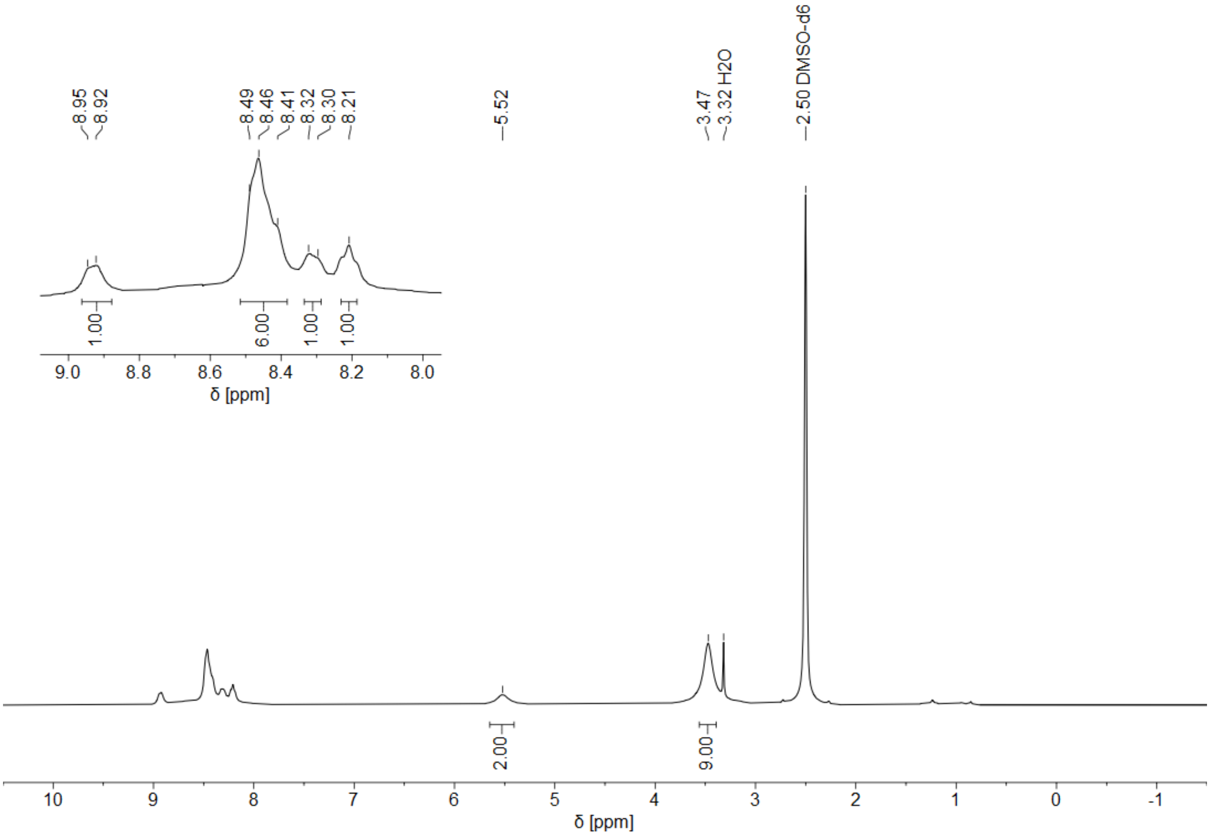

Figure S81: ^{1}H NMR spectrum (500 MHz, DMSO-d_6, 298 K) of **[Pyr]$_2$[Co]**.

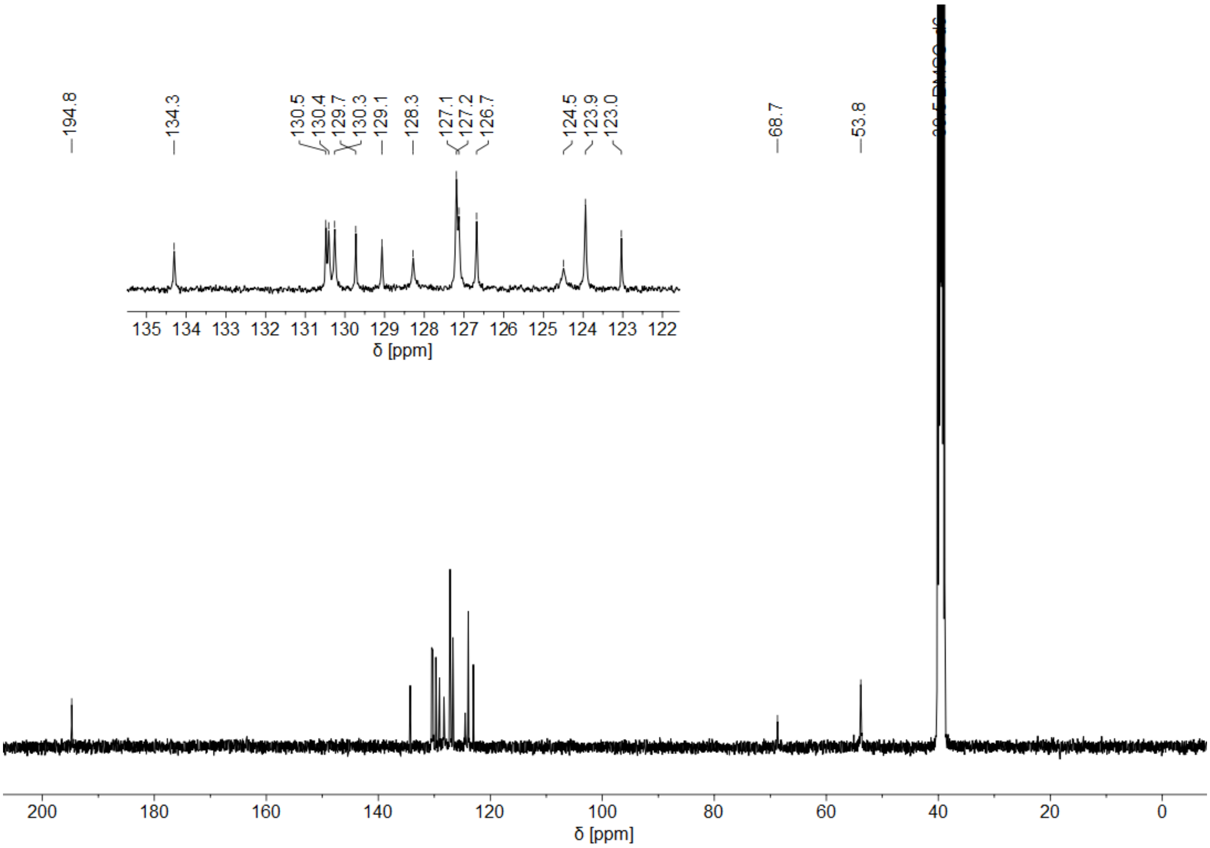

Figure S82: ^{13}C{^{1}H} NMR spectrum (101 MHz, DMSO-d_6, 298 K) of **[Pyr]$_2$[Co]**.

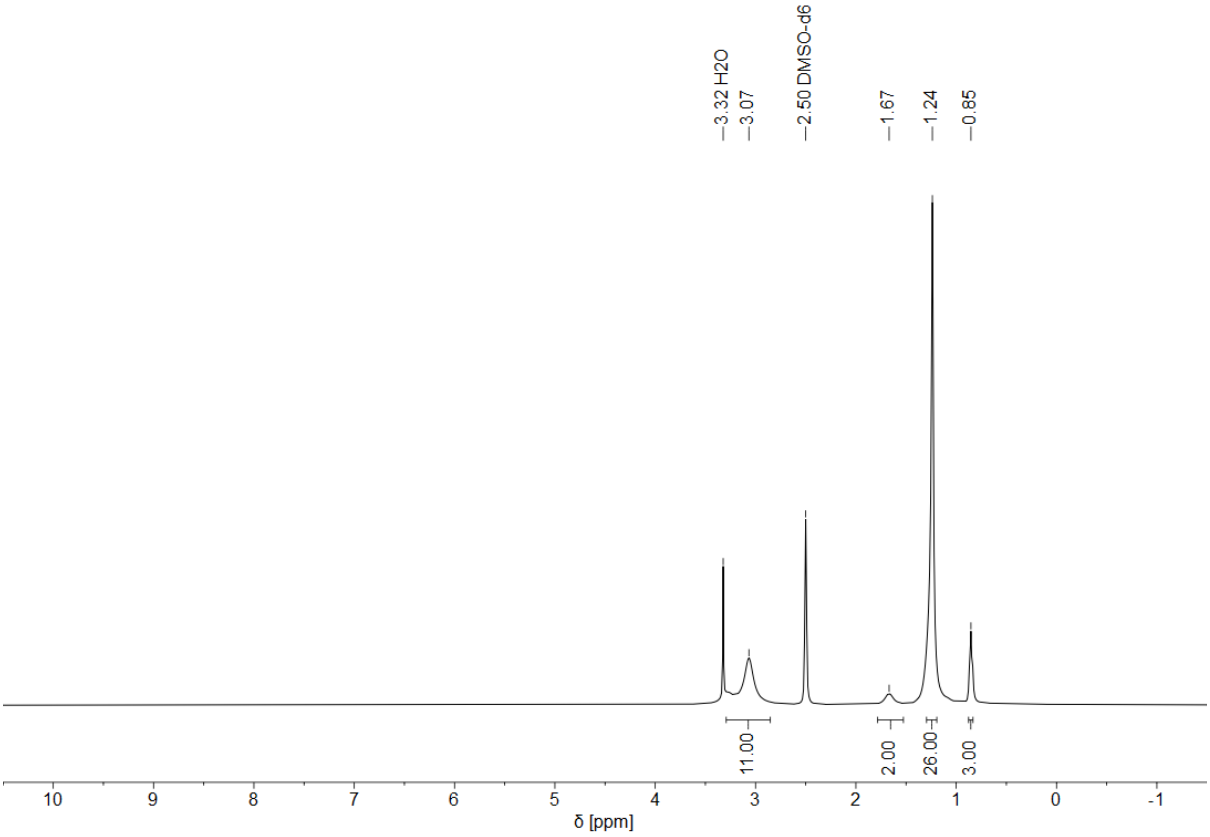

Figure S83: ^{1}H NMR spectrum (400 MHz, DMSO-d_6, 298 K) of **$[CTA]_2[Co]$**.

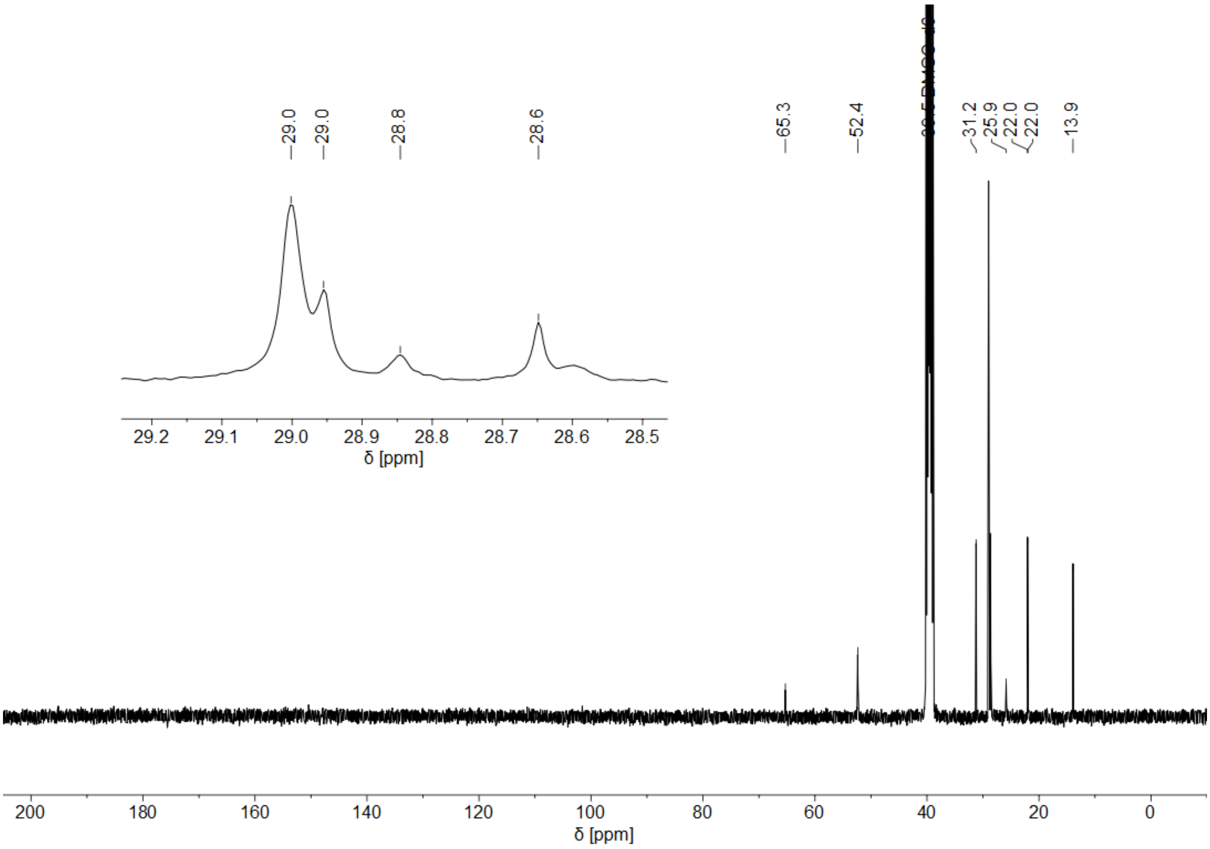

Figure S84: ^{13}C{^{1}H} NMR spectrum (101 MHz, DMSO-d_6, 298 K) of **$[CTA]_2[Co]$**.

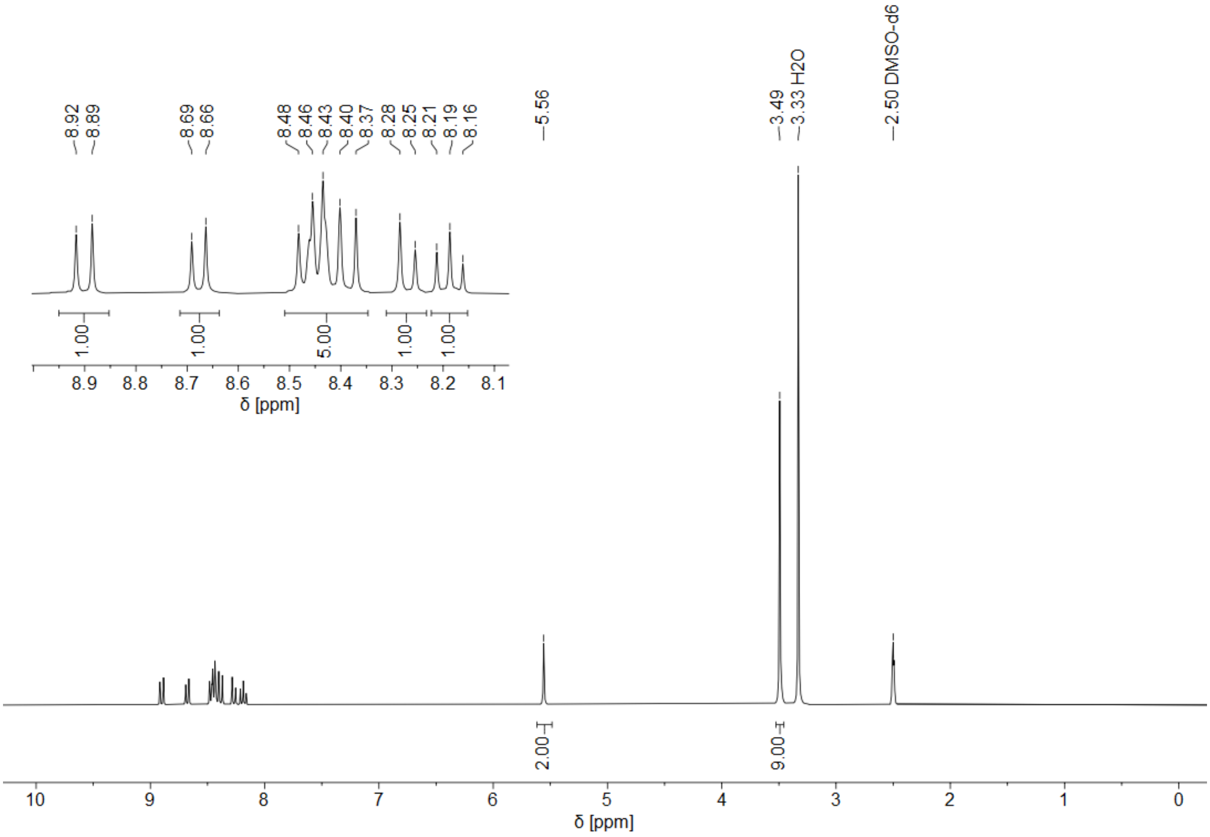

Figure S85: ^{1}H NMR spectrum (300 MHz, DMSO-d_6, 298 K) of **[Pyr]$_3$[Co(CN)$_6$]**.

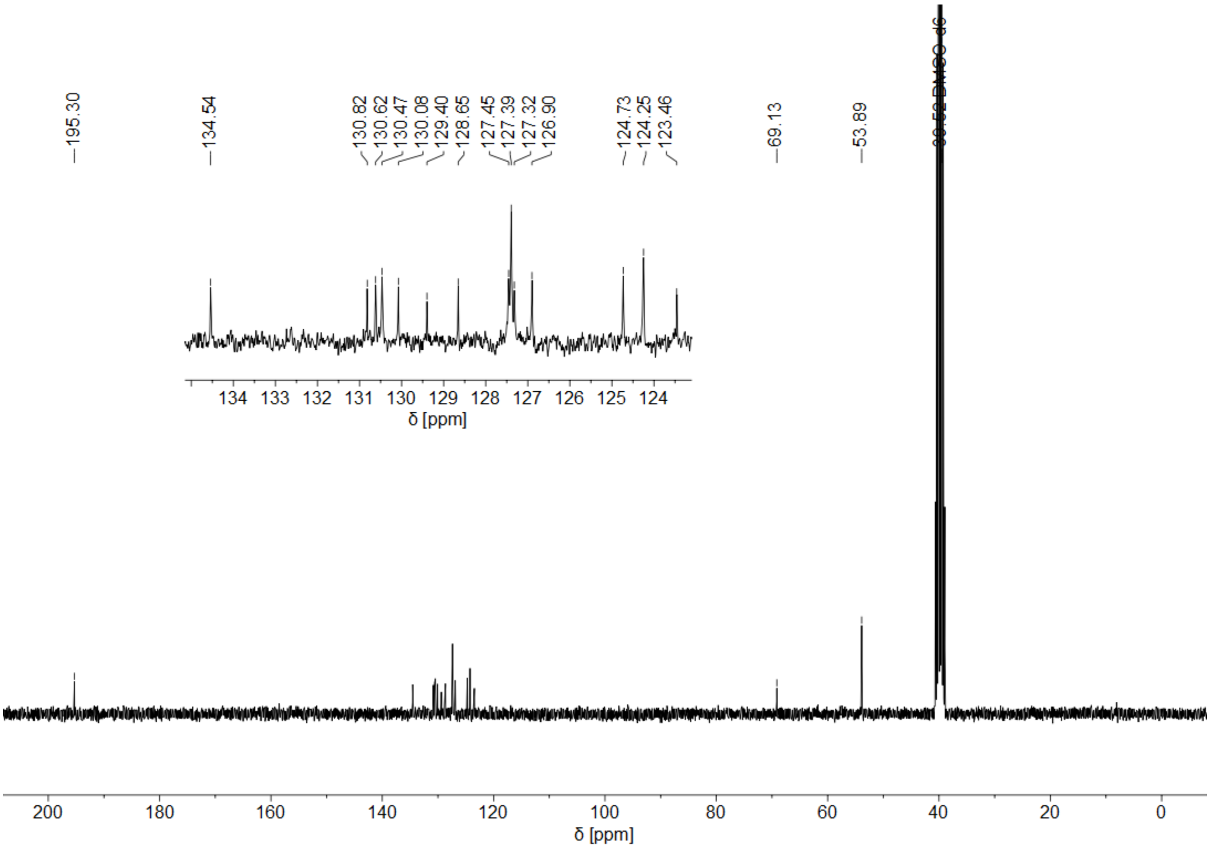

Figure S86: $^{13}C\{^{1}H\}$ NMR spectrum (101 MHz, DMSO-d_6, 298 K) of **[Pyr]$_3$[Co(CN)$_6$]**.

7.2 UV-Vis Spectra

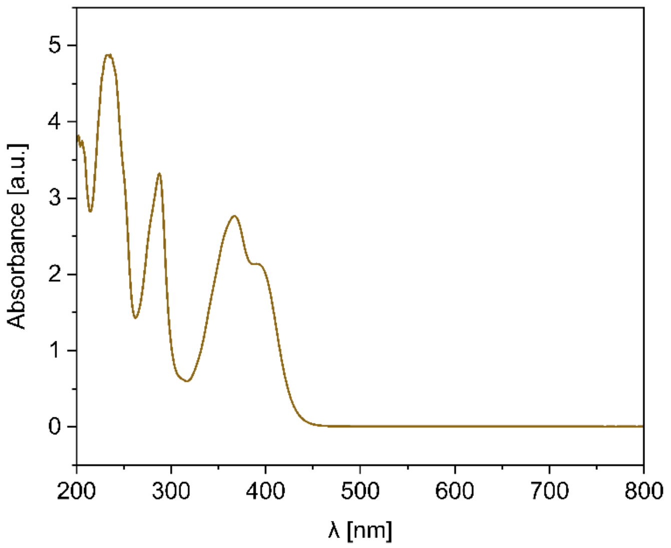

Figure S87: UV-Vis spectrum of pyrene ammonium bromide **[Pyr][Br]** (50.0 µM in MeOH).

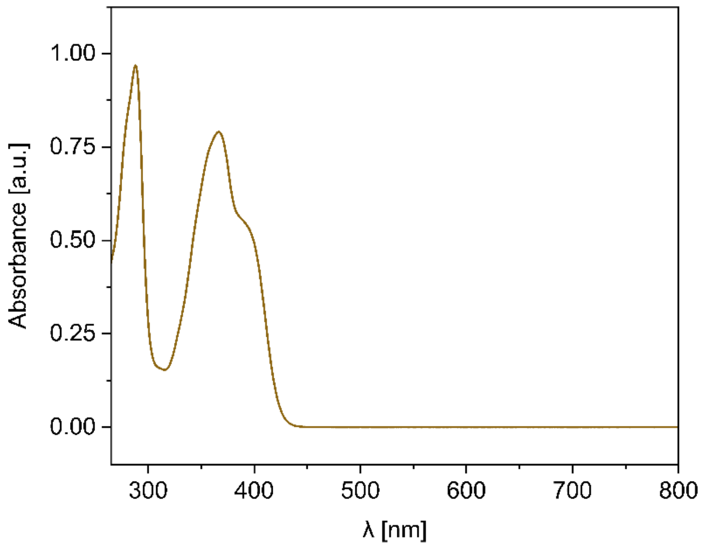

Figure S88: UV-Vis spectrum of pyrene ammonium bromide **[Pyr][Br]** (25.0 µM in DMF).

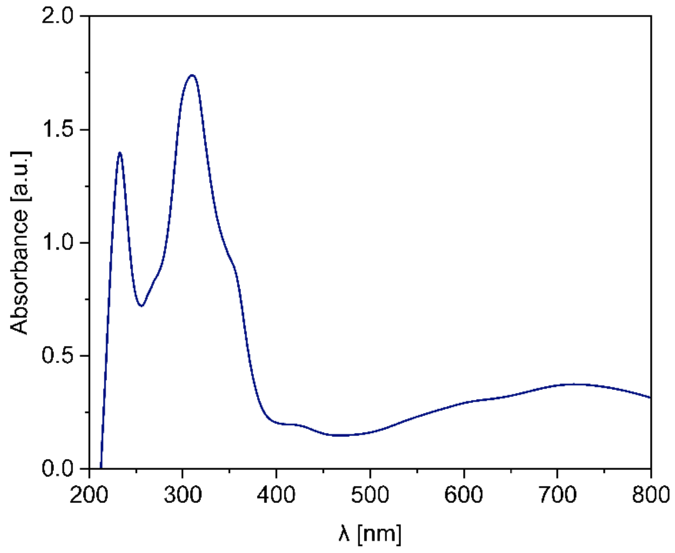

Figure S89: UV-Vis spectrum of [(*t*-Bu-SQ)$_2$Co]$_4$ (30.0 µM in THF).

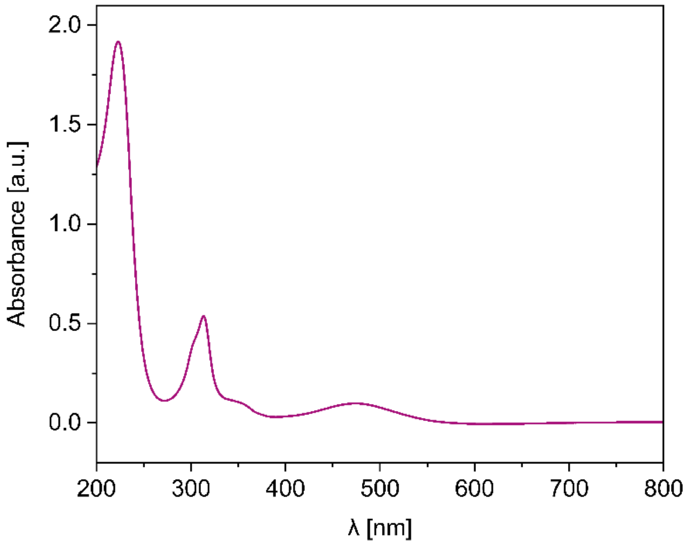

Figure S90: UV-Vis spectrum of **[(*n*-Bu)$_4$N]$_2$[Co]** (50.0 µM in MeOH).

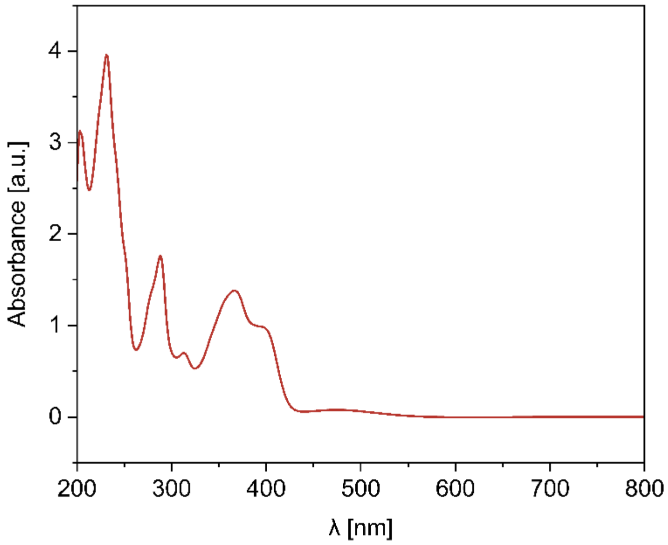

Figure S91: UV-Vis spectrum of **[Pyr]$_2$[Co]** (50.0 µM in MeOH).

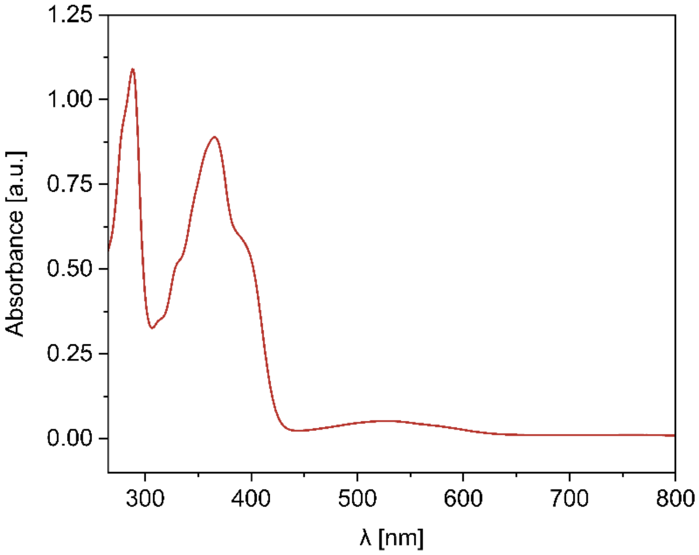

Figure S92: UV-Vis spectrum of **[Pyr]$_2$[Co]** (25.0 µM in DMF).

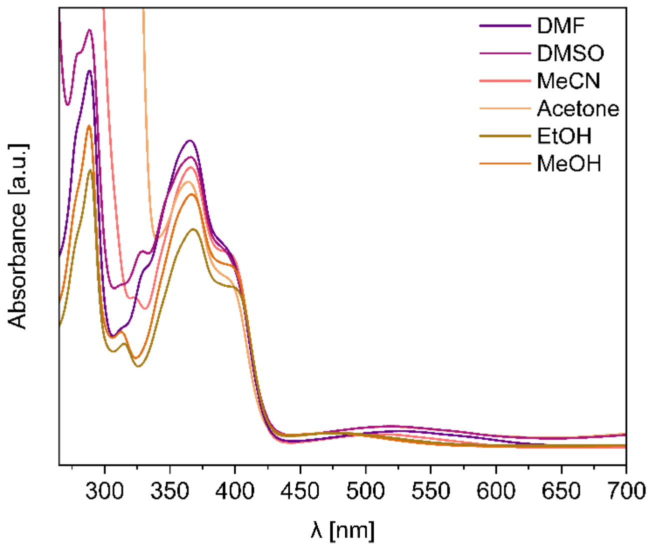

Figure S93: Overlay of UV-Vis spectra of **[Pyr]$_2$[Co]** (25 μM) in DMF (purple), DMSO (magenta), MeCN (melon), acetone (peach), EtOH (green), MeOH (orange).

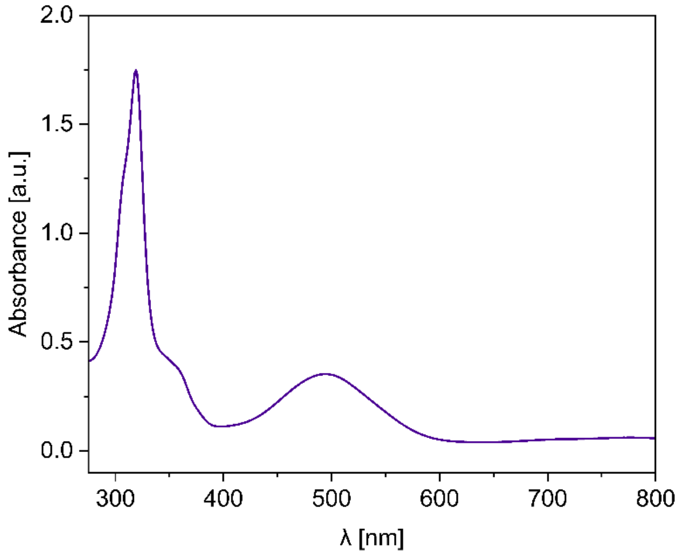

Figure 94: UV-Vis spectrum of **[CTA]$_2$[Co]** (130 μM in CH_2Cl_2).

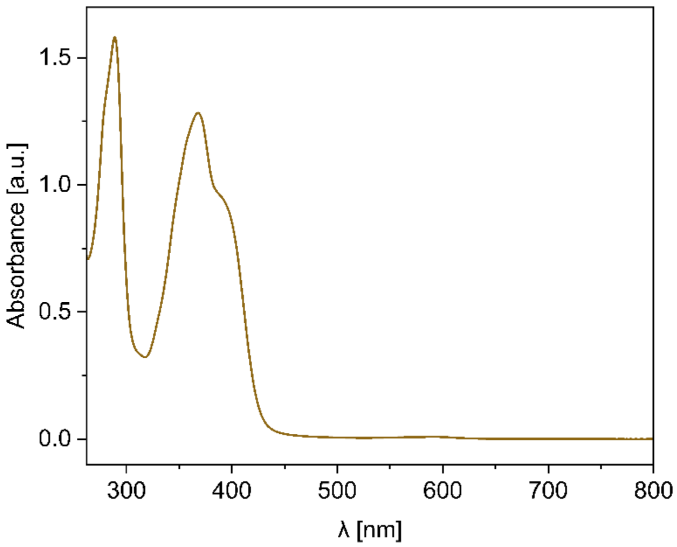

Figure S95: UV-Vis spectrum of **[Pyr]$_3$[Co(CN)$_6$]** (28 µM in DMSO).

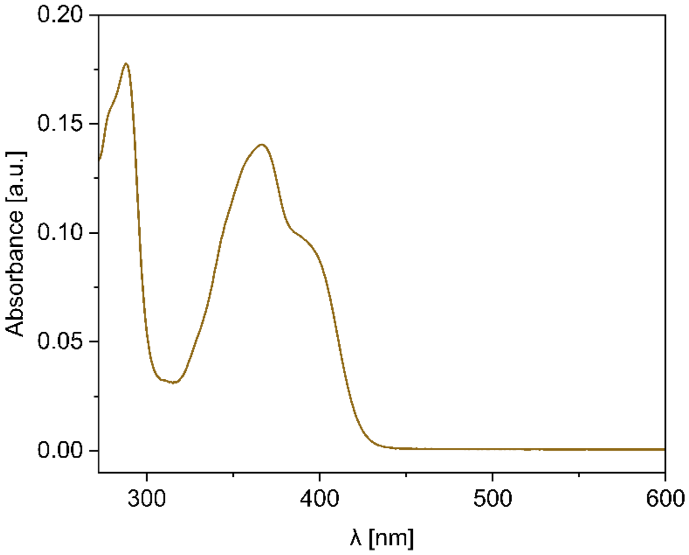

Figure S96: UV-Vis spectrum of **[Pyr]$_3$[Co(CN)$_6$]** (3.3 µM in DMF).

7.3 IR Spectra

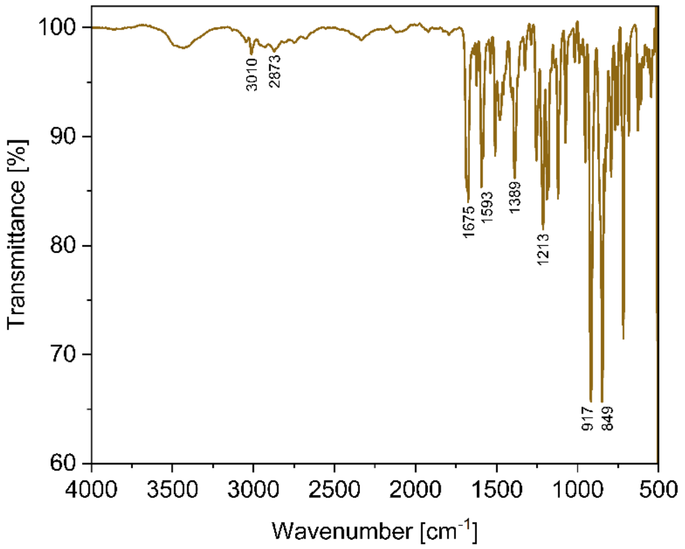

Figure S97: IR (ATR) spectrum of pyrene ammonium bromide **[Pyr][Br]**.

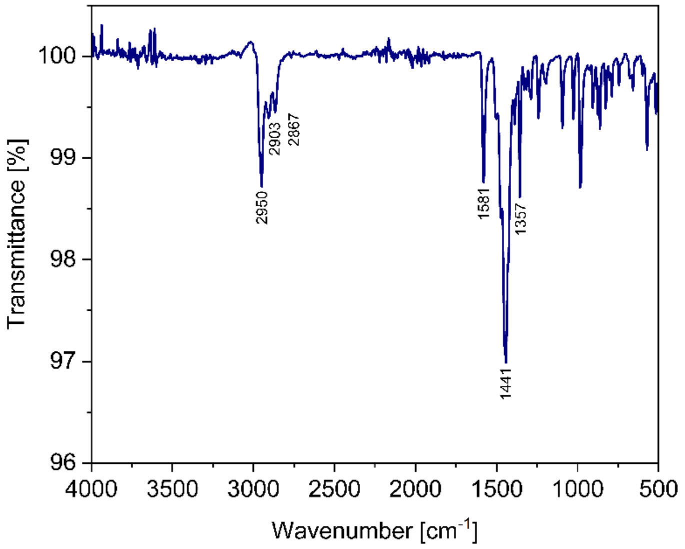

Figure S98: IR (ATR) spectrum of $[(t\text{-Bu-SQ})_2Co]_4$.

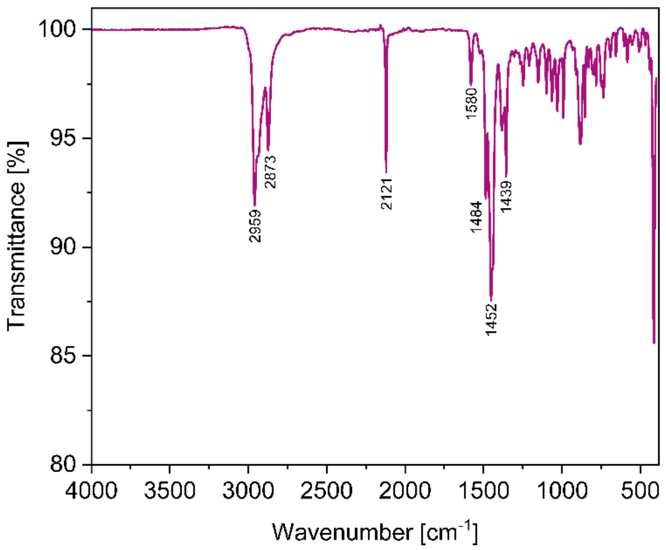

Figure S99: IR (ATR) spectrum of **[(*n*-Bu)$_4$N]$_2$[Co]**.

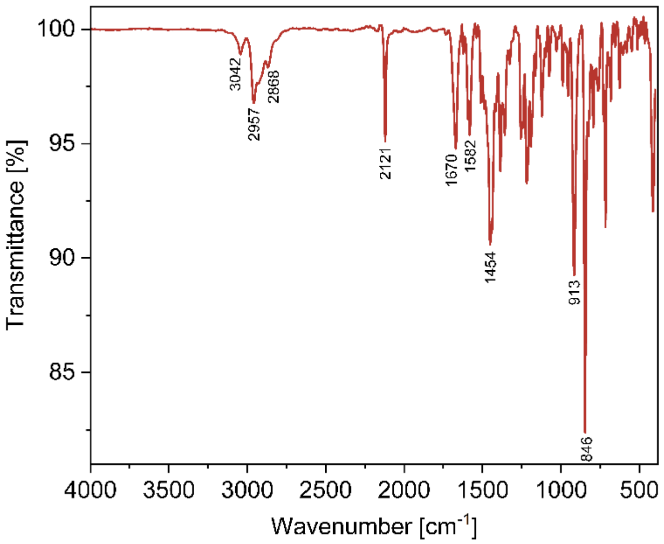

.

Figure S100: IR (ATR) spectrum of **[Pyr]$_2$[Co]**.

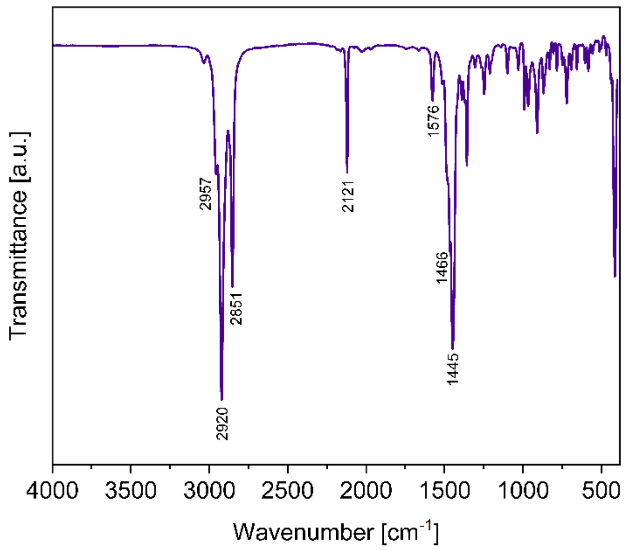

Figure S101: IR (ATR) spectrum of **[CTA]$_2$[Co]**.

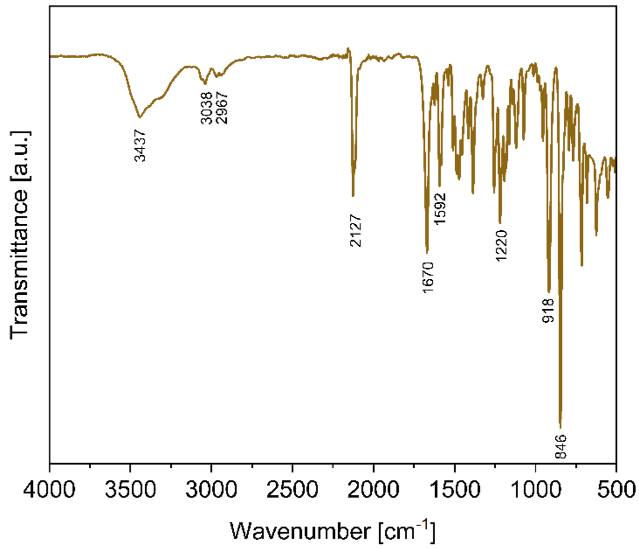

Figure S102: IR (ATR) spectrum of **[Pyr]$_3$[Co(CN)$_6$]**.

7.4 X-Ray Crystallographic Data

Crystal data and structure refinement of ***[Pyr]₂[Co]***

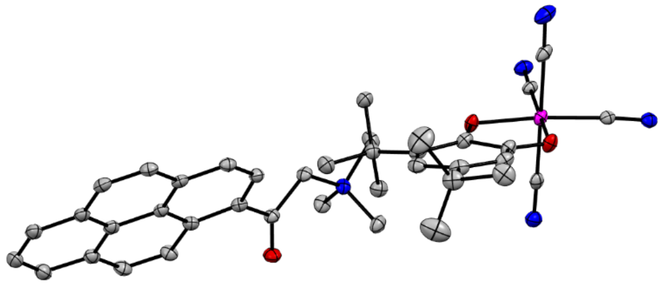

Identification code	2479193
Empirical formula	$C_{63}H_{72}CoN_6O_7$
Formula weight	1084.19
Temperature [K]	100.00
Crystal system	triclinic
Space group	P-1
a [Å]	8.6631(2)
b [Å]	18.2576(4)
c [Å]	18.4718(4)
α [°]	81.9530(10)
β [°]	84.4740(10)
γ [°]	78.2240(10)
Volume [Å^3]	2825.06(11)
Z	2
ρ_{calc} [g/cm^3]	1.275
μ [mm^{-1}]	2.851
F(000)	1150.0
Crystal size [mm^3]	0.101 × 0.075 × 0.025
Radiation	CuKα (λ = 1.54178)
2Θ range for data collection [°]	4.844 to 145.146
Index ranges	−10 ≤ h ≤ 10, -22 ≤ k ≤ 22, -22 ≤ l ≤ 22
Reflections collected	111225
Independent reflections	11185 [R_{int} = 0.1098, R_{sigma} = 0.0451]
Data/restraints/parameters	11185/57/743
Goodness-of-fit on F^2	1.059
Final R indexes [I>=2σ (I)]	R_1 = 0.0642, wR_2 = 0.1858
Final R indexes [all data]	R_1 = 0.0852, wR_2 = 0.2069
Largest diff. peak/hole [e Å^{-3}]	1.16/−0.78

Packing Diagrams

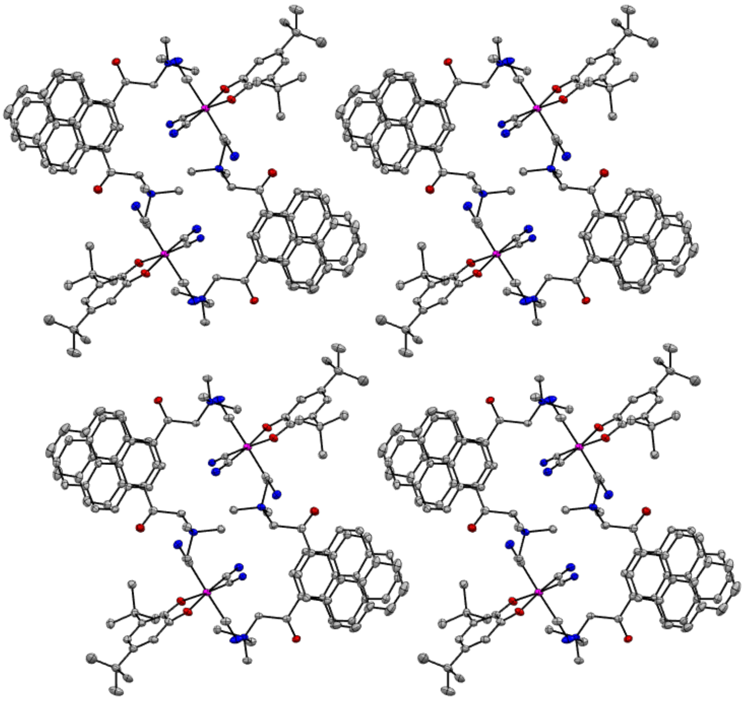

Figure S103: Packing of **[Pyr]₂[Co]** along the a axis in the solid-state with 50% probability ellipsoids. Hydrogen atoms are omitted.

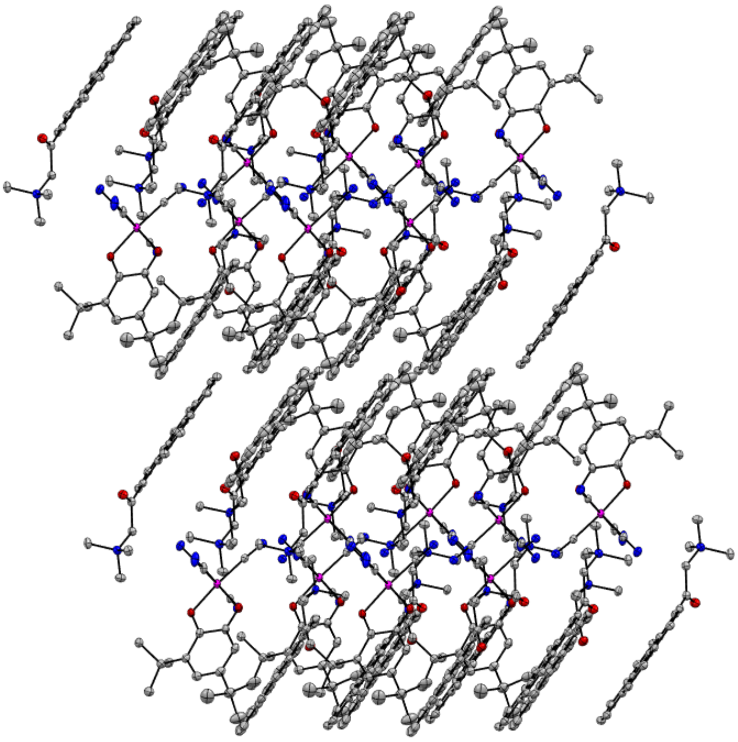

Figure S104: Packing of **[Pyr]₂[Co]** along the c axis in the solid-state with 50% probability ellipsoids. Hydrogen atoms are omitted.

*Crystal data and structure refinement of **$[Pyr]_3[Co(CN)_6]$***

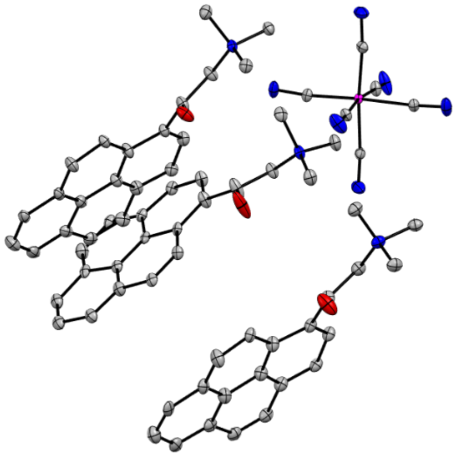

Identification code	2500980
Empirical formula	$C_{276}H_{240}Co_4N_{36}O_{12}$
Formula weight	4488.75
Temperature [K]	100.00(10)
Crystal system	orthorhombic
Space group	$Pca2_1$
a [Å]	33.6120(2)
b [Å]	14.67609(10)
c [Å]	24.7122(2)
α [°]	90
β [°]	90
γ [°]	90
Volume [Å3]	12190.36(16)
Z	2
ρcalc [g/cm3]	1.223
μ [mm^{-1}]	2.632
F(000)	4704.0
Crystal size [mm3]	0.103 × 0.089 × 0.042
Radiation	Cu Kα (λ = 1.54184)
2Θ range for data collection [°]	6.36 to 160.258
Index ranges	−42 ≤ h ≤ 40, −18 ≤ k ≤ 10, −31 ≤ l ≤ 30
Reflections collected	57417
Independent reflections	21657 [R_{int} = 0.0337, R_{sigma} = 0.0428]
Data/restraints/parameters	21657/1819/1496
Goodness-of-fit on F2	1.080
Final R indexes [I>=2σ (I)]	R_1 = 0.0930, wR_2 = 0.2739
Final R indexes [all data]	R_1 = 0.1086, wR_2 = 0.2904
Largest diff. peak/hole [e $Å^{-3}$]	0.93/−0.40

Packing Diagrams

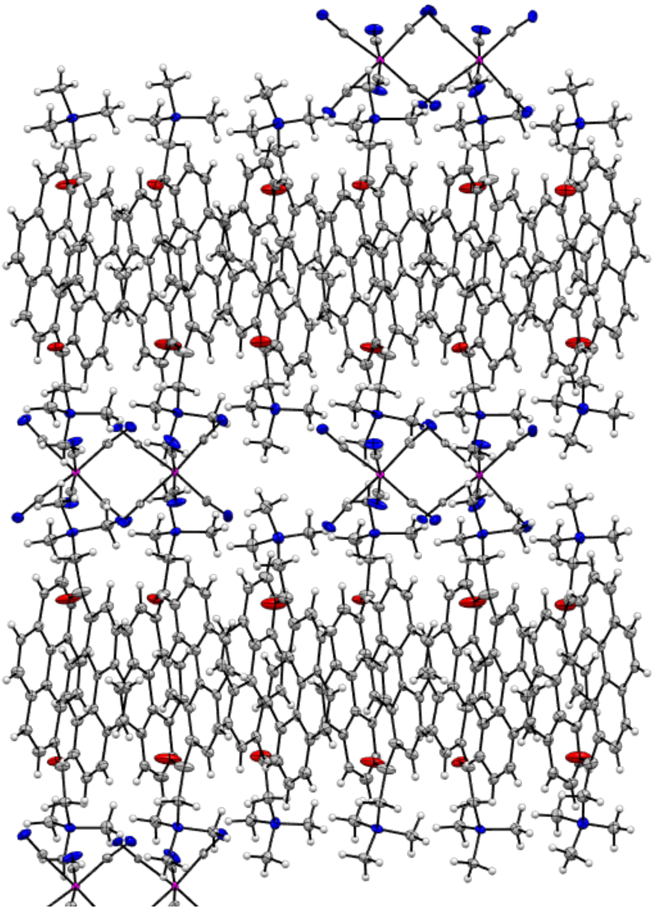

Figure S105: Packing of **[Pyr]$_3$[Co(CN)$_6$]** along the b axis in the solid-state with 50% probability ellipsoids. Hydrogen atoms are omitted.

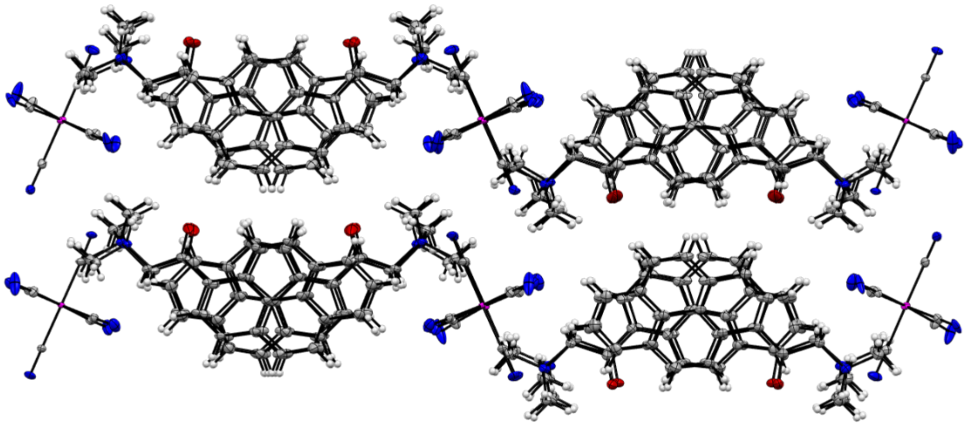

Figure S106: Packing of **[Pyr]$_3$[Co(CN)$_6$]** along the c axis in the solid-state with 50% probability ellipsoids. Hydrogen atoms are omitted.

8 DFT Computations

8.1 General Considerations

All density functional theory (DFT) calculations were computed using the *ORCA 6.0.0* program package.[25,26] Molecular orbitals, spin- and electron-density plots were visualized using *ChemCraft*.

Geometry optimizations and free energy calculations of the complex anion were carried out at the unrestricted Kohn-Sham (UKS) level using the B3LYP functional[27–29] with Grimme's D3BJ dispersion correction.[30] The def2-TZPV basis set was employed for Co, while the def2-SVP basis set was used for all lighter elements.[31] The optimization criteria followed ORCA's default tight convergence settings, and frequency calculations confirmed that all optimized geometries corresponded to true local minima (zero imaginary frequencies). Free energy corrections were obtained from vibrational frequency calculations, incorporation standard thermodynamic contributions (298 K, 1 atm).

Solvent Effects: Solvent effects were incorporated using the conductor-like polarizable continuum model (CPCM)[32] with the SMD solvation approach.[33] Calculations were performed for *N*,*N*-dimethylformamide (dielectric constant: ε = 36.7) and methanol as implemented in *ORCA*. Solvent corrections were applied to both geometry optimizations and single-point energy calculations.

TD-DFT Calculations: Time-dependent density functional theory (TD-DFT) calculations were performed using the range-separated hybrid functional ωB97X-D3[34] with the def2-TZVP basis set. The RIJCOSX[35] approximation was used with the def2/J auxiliary basis set to accelerate calculations. Solvent effects in DMF and methanol were included in the CPCM model (see above), while xyz coordinates from the geometry optimization at the TD-DFT level of theory where used. Excited-state properties and transition dipole moments were analyzed to characterize electronic transitions.

Sample ORCA Input File for Geometry Optimization and Frequency Calculations:

```
! BP86 def2-TZVP
! RI def2/J
! Opt
! Freq
! Normalprint Printbasis PrintMOs

%pal nprocs 16 end
%maxcore 8000

*xyz “charge” “spin multiplicity”
XYZ Coordinates
*
```

Sample ORCA Input File for Solvent-Corrected TD-DFT Calculations:

```
! wB97X-D3 def2-TZVP
! RIJCOSX def2/J
! CPCM

%cpcm
  epsilon 36.7 #Dielectric constant of DMF
end

%pal nprocs 16 end
%maxcore 8000

%tddft
  nroots 150
  maxdim 5
end
```

8.2 Ground-State Energies

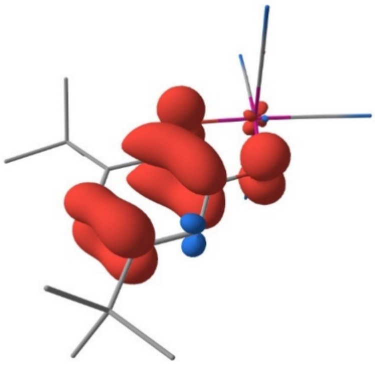

Figure S107: DFT-computed spin-density plot of **[Co]**$^{2-}$ using contour levels of +0.4% (red) and −0.4% (blue).

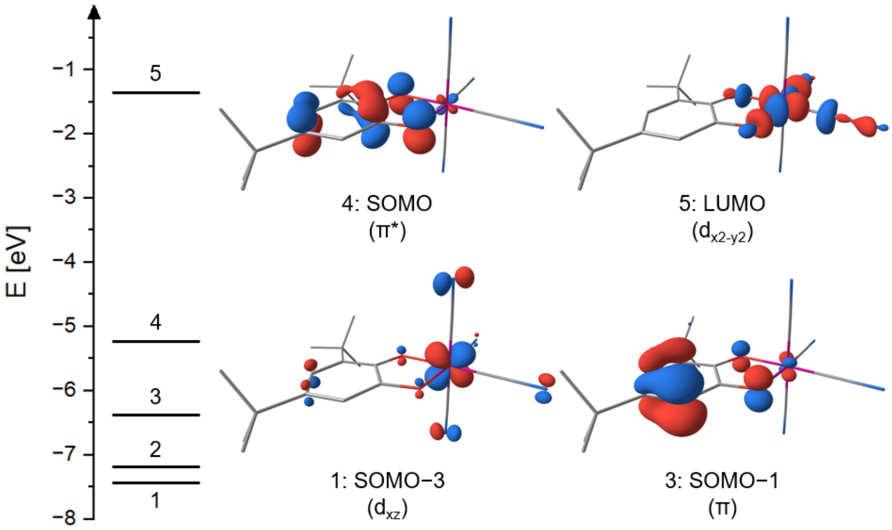

Figure S108: Energy-splitting of DFT-computed (B3LYP/def2-TZVP) frontier molecular orbitals of **[Co]**$^{2-}$ (solvent-corrected for MeOH) and their isosurfaces using contour levels of ±7%.

8.3 Time-Dependent (TD) DFT Calculations

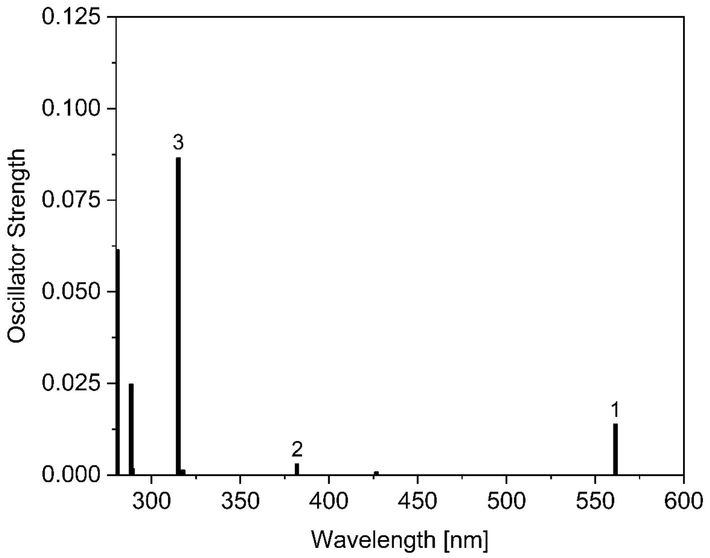

Figure S109: Data from the TD-DFT calculations of **[Co]**$^{2-}$ at the CPCM(DMF) ωB97X-D3/def2-TZVP level of theory.

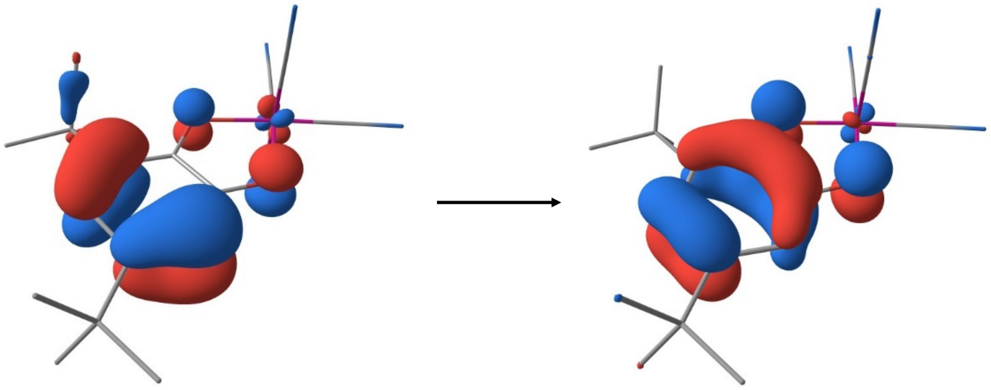

Figure S110: Molecular orbital plots of the SOMO−1 → SOMO transition (#1) at 562 nm in DMF.

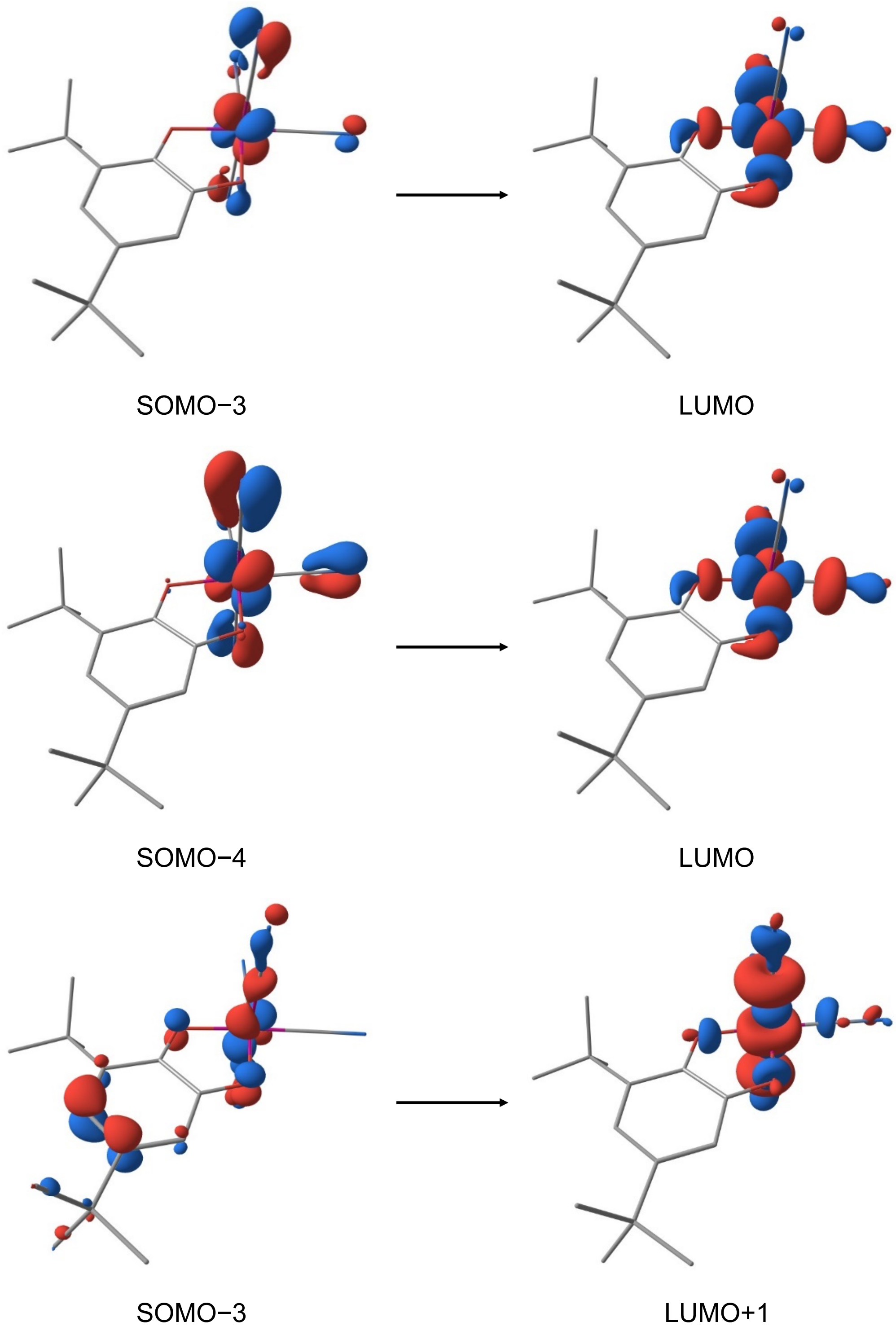

Figure S111: Molecular orbital plots of the dominant transitions (23%, 21% and 13%) of transition #2 at 382 nm in DMF.

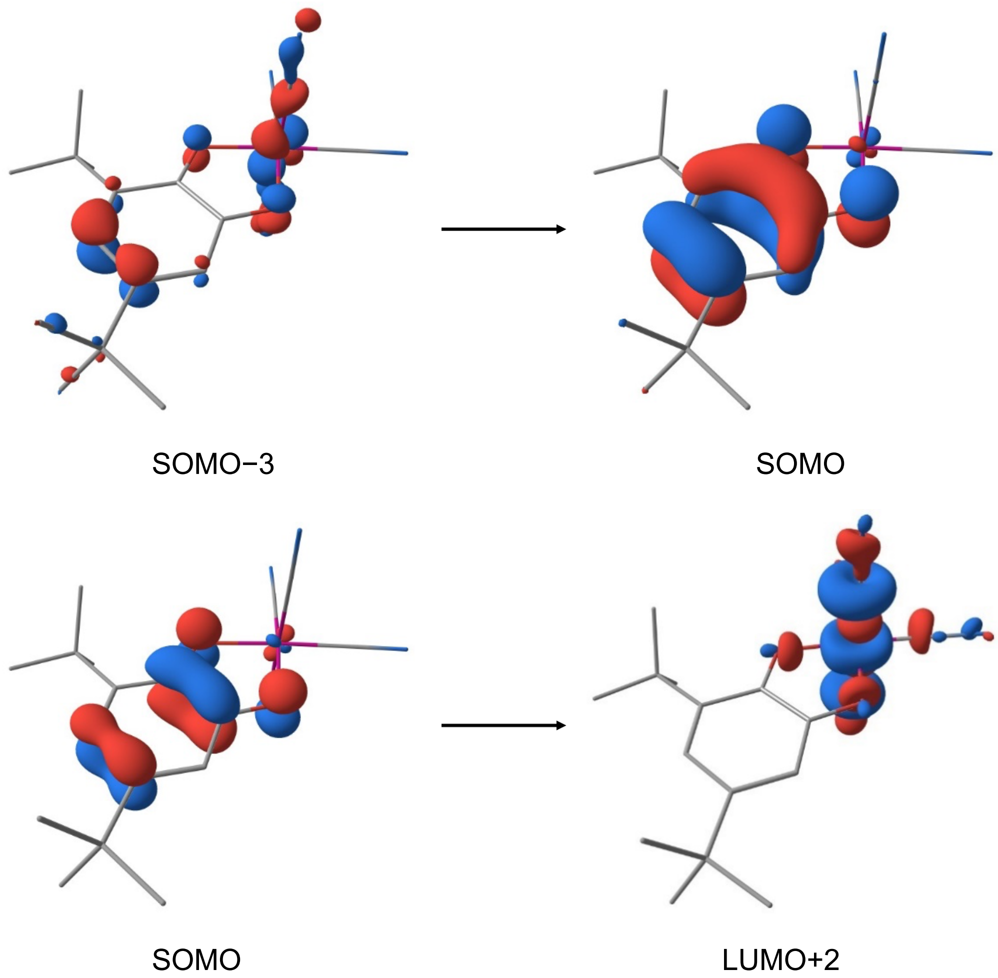

Figure S112: Molecular orbital plots of the dominant transitions (60% and 16%) of transition #3 at 315 nm in DMF.

Coordinates

Low-spin Co(III)

S = 2

Final Gibbs Free Energy: −2449.18353966 Eh

Co	-1.90526436140232	-1.56190761971554	-0.47411773381728
C	-2.66818348541481	-1.20847540209374	1.25466810058234
C	-1.89178350111120	0.27936742761788	-0.84289435270671
C	-0.18495916611453	-1.45137607351185	0.27020966302288
C	-1.12874481265314	-1.90758875888454	-2.19818021609121
N	-3.14369842462066	-1.01912170398006	2.29447270803108
N	-1.90268012277568	1.41371298669599	-1.07890623194075
N	0.87587009611068	-1.40670659762690	0.73411161926149
N	-0.67597621784071	-2.13309988275425	-3.24092246194534
O	-3.70297616231119	-1.80647488507241	-1.22392753024008
O	-2.03055338870500	-3.48681758930793	-0.13566406698656
C	-4.08677683697919	-3.03689309633551	-1.12827683715115
C	-3.15970424447641	-3.97279285579314	-0.52167074253792
C	-5.34580196869864	-3.47372081637362	-1.58451485543684
C	-3.52762118167922	-5.34830389458723	-0.38190534644347
C	-4.76926864125525	-5.70054788816833	-0.84860574694493
C	-5.69764588705719	-4.79344877883301	-1.45098086964801
C	-2.57020820858699	-6.35229581276048	0.26066994436627
C	-7.04317242999382	-5.34136190532571	-1.91764234016366
C	-7.93367324262068	-4.25421709992756	-2.52632139493074
H	-8.15494482365451	-3.46368597017090	-1.80645473916238
H	-8.88251019842865	-4.69540164693517	-2.83785990359581
H	-7.47520557474255	-3.79742382964196	-3.40580847828535
C	-7.78270194412054	-5.95828430221234	-0.71503156440328
H	-7.96228460792177	-5.20772222745975	0.05811994788523

H	-7.21922395028943	-6.77790049839087	-0.26760530542082
H	-8.74909850919719	-6.35327336884886	-1.03743624347309
C	-6.80280313099083	-6.42968014631612	-2.98122930336691
H	-6.27131330780530	-6.02086611449747	-3.84361638371473
H	-7.75978936117178	-6.82726955101010	-3.32784242029163
H	-6.21860330505615	-7.26207685940792	-2.58657607442613
C	-2.25456545486876	-5.91832326243744	1.70541452957077
H	-1.76895968534682	-4.94481058877673	1.74025091121948
H	-1.58988117900408	-6.64827395660240	2.17406896218643
H	-3.16940626518474	-5.86416816752435	2.30062464661734
C	-1.26908179182829	-6.42872523926241	-0.56194383014827
H	-0.76291782058240	-5.46641402810261	-0.61240522388660
H	-1.47850279266468	-6.75898941581798	-1.58227055684493
H	-0.58590982573400	-7.15039787526020	-0.10728325018780
C	-3.17296048143906	-7.76069154551585	0.31571811273729
H	-4.08770737856382	-7.79155867287530	0.91160636425722
H	-2.45275720182547	-8.43830219131980	0.77844334901177
H	-3.39933845601070	-8.14751108682721	-0.68010915172525
H	-5.07756977444225	-6.72958190520309	-0.75944075923132
H	-6.00116099093988	-2.73930730284479	-2.02935494360052

High-spin Co(III)

S = 4 (Broken Symmetry Calculation)

Final Gibbs Free Energy: −2450.02430442 Eh

Co	-1.86361847745065	-1.54468536852869	-0.45627507845714
C	-2.64441610130254	-1.21107738084151	1.28705509793214
C	-1.83942062295329	0.29709317512213	-0.83417743726869
C	-0.13732676648402	-1.47499843045321	0.28639449598668
C	-1.11418182779323	-1.94558204581662	-2.19918473975233
N	-3.16946914896542	-1.09553663161365	2.32397601111101

N	-1.86451705055194	1.43668043264898	-1.08563684232545
N	0.93081005009314	-1.48699044041248	0.75709868219997
N	-0.72205428964950	-2.26923683467725	-3.25071363231594
O	-3.70816505955759	-1.80903301081999	-1.20991909677517
O	-2.03590938348339	-3.50727269659198	-0.11701339032345
C	-4.08475107258013	-3.01847398972639	-1.11785986086540
C	-3.15451156529332	-3.97099006660896	-0.50785108287015
C	-5.35569423207817	-3.47850522983614	-1.57613103859307
C	-3.53723554634878	-5.35284599590057	-0.38011411963331
C	-4.78843995643096	-5.71968831590010	-0.84959117864948
C	-5.71386166698718	-4.80823441911883	-1.44910539809370
C	-2.55608310464190	-6.33992143699977	0.26209275171593
C	-7.07017996333363	-5.35188915047877	-1.92513089280259
C	-7.94840672925810	-4.25482355113228	-2.54610794852521
H	-8.16139903828866	-3.45305741482672	-1.82284582018877
H	-8.91137417675648	-4.68229454986184	-2.87467110670977
H	-7.46262433764826	-3.79745718661862	-3.42145616260106
C	-7.83502069508090	-5.95672437438452	-0.72772362086152
H	-8.01192767124215	-5.18995947438559	0.04312883081797
H	-7.26892385819218	-6.77509976618470	-0.25775410872445
H	-8.81273934563825	-6.35950643051094	-1.04907499202207
C	-6.84477227935387	-6.44619087140425	-2.99114961601368
H	-6.30324755037628	-6.03415710356495	-3.85737228769414
H	-7.80872778365322	-6.85495295790867	-3.34507135957101
H	-6.24828796128349	-7.28113833666642	-2.59351962005427
C	-2.24983973030872	-5.88616942266826	1.70749969121532
H	-1.83426513010517	-4.87007031021210	1.71608636001256
H	-1.52339031841296	-6.57321322843458	2.17751944412480
H	-3.16996251270600	-5.88845669824503	2.31545869776095
C	-1.24828539981039	-6.36882515191066	-0.56117126063772

H	-0.80172489343369	-5.36751830962988	-0.61615957612280
H	-1.44706441752965	-6.71555590150029	-1.58902677373413
H	-0.52095752996301	-7.05952467774734	-0.09797968040417
C	-3.12345377468992	-7.76753157517702	0.31472857597846
H	-4.04882020236846	-7.82040562345687	0.91191302660292
H	-2.38471155611219	-8.44043981556342	0.78171496137612
H	-3.34328480935309	-8.15931773406098	-0.69217382755886
H	-5.09278312147333	-6.76234176936582	-0.76011905303392
H	-6.00699939116884	-2.72678992802424	-2.02100602365135

Low-spin Co(III)

S = 2 (Geometry Optimization at the TD-DFT level of theory)

Final Gibbs Free Energy: −2450.37347118 Eh

Co	-1.91534501620658	-1.57364774293983	-0.47057559141057
C	-2.63812212862211	-1.18760443303043	1.26794993728857
C	-1.89144903457360	0.26395637047459	-0.86261997403899
C	-0.17966799217431	-1.46150570416637	0.24115623055791
C	-1.16370822358809	-1.94070279621176	-2.20189011469387
N	-3.08631132053681	-0.97539575450129	2.31262336154019
N	-1.89218553933810	1.39239455018922	-1.11282448801675
N	0.88774562470505	-1.41146980672856	0.68183286298323
N	-0.72414880214005	-2.17807597909648	-3.24483560525137
O	-3.71552580757574	-1.81160996588054	-1.18664200009641
O	-2.04801761574277	-3.48512780548787	-0.11095218623856
C	-4.09638960802218	-3.03005018864285	-1.09585005168523
C	-3.16939743676258	-3.96778611019400	-0.49478310175929
C	-5.35740792122414	-3.47189108276692	-1.55662377887772
C	-3.53775975774850	-5.34686761973986	-0.36271654801414
C	-4.77253210386927	-5.69761960494063	-0.83257812620745
C	-5.70190740297296	-4.78801798471403	-1.43451580187881

C	-2.57323093098036	-6.35125054840177	0.26822081843427
C	-7.03947947602913	-5.34187044568741	-1.91969552552836
C	-7.92388779577253	-4.25703295544559	-2.53381331971353
H	-8.16542039628454	-3.47312054363151	-1.81229941519895
H	-8.86439808652458	-4.70018642785813	-2.86847461028690
H	-7.45290201325392	-3.78959852331672	-3.40172553041353
C	-7.79045544830882	-5.96531983714557	-0.73363418121749
H	-7.98290580001099	-5.21998762941069	0.04224955486486
H	-7.23438278837633	-6.78845060879898	-0.28107658370088
H	-8.75263040138599	-6.35994036274740	-1.06984973258118
C	-6.78163562889313	-6.41919101695878	-2.98403342501883
H	-6.24161615617575	-6.00332147551981	-3.83838755842478
H	-7.73149311761396	-6.81945330373413	-3.34758241035180
H	-6.19930167717199	-7.25395988545737	-2.58929259246493
C	-2.25406592850291	-5.92847250692346	1.71068899434449
H	-1.78104109806357	-4.94784404193989	1.74833058797985
H	-1.57501563219162	-6.65264004282670	2.16852244775007
H	-3.16435581960359	-5.89333805828115	2.31522295491699
C	-1.27961770285349	-6.40805438390224	-0.55904684959680
H	-0.77650307891652	-5.44199576610229	-0.58527691796131
H	-1.49116527268054	-6.71156991754022	-1.58777545481442
H	-0.59186102661607	-7.14006515305264	-0.12743595568759
C	-3.16957259912359	-7.75919592665020	0.31244091864856
H	-4.08345204450122	-7.79846318446377	0.91046291956839
H	-2.44723096289268	-8.44025249411063	0.76781251604737
H	-3.39802148608104	-8.13990115368887	-0.68603586212240
H	-5.07960685207370	-6.73078940195278	-0.75243902296443
H	-6.01464069272427	-2.73642274607329	-2.00065178870740

9 References

[1] Fulmer, G. R; Miller, A. J. M.; Sherden, N. H.; Gottlieb, H. E.; Nudelman, A.; Stoltz, B. M.; Bercaw, J. E.; Goldberg, K. I. NMR Chemical Shifts of Trace Impurities: Common Laboratory Solvents, Organics, and Gases in Deuterated Solvents Relevant to the Organometallic Chemist. *Organometallics* **2010**, *29*, 2176–2179.

[2] Stoll, S.; Schweiger, A. EasySpin, a comprehensive software package for spectral simulation and analysis in EPR. *J. Magn. Reson.* **2006**, *178*, 42–55.

[3] Stoll, S. in *Methods Enzymol*, Qin, P. Z.; Warncke, K., Eds.; Academic Press, **2015**, pp. 121–142.

[4] Fairley, N.; Fernandez, V.; Richard-Plouet, M.; Guillot-Deudon, C.; Walton, J.; Smith, E.; Flahaut, D.; Greiner, M.; Biesinger, M.; Tougaard, S.; Morgan, D.; Baltrusaitis, J. Systematic and collaborative approach to problem solving using X-ray photoelectron spectroscopy. *Appl. Surf. Sci. Adv.* **2021**, *5*, 100112.

[5] Dolomanov, O. V; Bourhis, L. J.; Gildea, R. J.; Howard, J. A. K.; Puschmann, H. OLEX2: a complete structure solution, refinement and analysis program. *Appl. Cryst.* **2009**, *42*, 339–341.

[6] Sheldrick, G. M. Crystal structure refinement with SHELXL. *Acta Cryst C* **2015**, *71*, 3–8.

[7] Tomonari, Y.; Murakami, H.; Nakashima, N. Solubilization of Single-Walled Carbon Nanotubes by using Polycyclic Aromatic Ammonium Amphiphiles in Water—Strategy for the Design of High-Performance Solubilizers. *Chem. Eur. J.* **2006**, *12*, 4027–4034.

[8] Buchanan, R. M.; Fitzgerald, B. J.; Pierpont, C. G. Semiquinone radical anion coordination to divalent cobalt and nickel. Structural features of the bis (3, 5-di-tert-butyl-1, 2-semiquinone) cobalt (II) tetramer. *Inorg. Chem.* **1979**, *18*, 3439–3444.

[9] Arzberger, S.; Soper, J.; Anderson, O. P.; la Cour, A.; Wicholas, M. Synthesis and structure of an air-stable, free-radical cobalt (III) semiquinone complex. *Inorg. Chem.* **1999**, *38*, 757–761.

[10] Evans, D. F. 400. The determination of the paramagnetic susceptibility of substances in solution by nuclear magnetic resonance. *J. Chem. Soc. (Resumed)* **1959**, 2003–2005.

[11] Bain, G. A.; Berry, J. F. Diamagnetic Corrections and Pascal's Constants. *J. Chem. Educ.* **2008**, *85*, 532.

[12] McLaren, R. L.; Owen, G. R.; Morgan, D. J. Analysis induced reduction of a polyelectrolyte. *Results Surf. Interfaces* **2022**, *6*, 100032.

[13] Edwards, L.; Mack, P.; Morgan, D. J. Recent advances in dual mode charge compensation for XPS analysis. *Surf. Interface Anal.* **2019**, *51*, 925–933.

[14] Yang, D.-Q.; Rochette, J.-F.; Sacher, E. Spectroscopic Evidence for π−π Interaction between Poly(diallyl dimethylammonium) Chloride and Multiwalled Carbon Nanotubes. *J. Phys. Chem. B* **2005**, *109*, 4481–4484.

[15] Kolbeck, C.; Cremer, T.; Lovelock, K. R. J.; Paape, N.; Schulz, P. S.; Wasserscheid, P.; Maier, F.; Steinrück, H.-P. Influence of Different Anions on the Surface Composition of Ionic Liquids Studied Using ARXPS. *J. Phys. Chem. B* **2009**, *113*, 8682–8688.

[16] Moulder, J. F.; Stickle, W. F.; Sobol, P. E.; Bomben, K. D.; *Handbook of X-Ray Photoelectron Spectroscopy*, Chastain, J., Ed.; Perkin Elmer Corp., Eden Prairie, MN, **1992**.

[17] Nadurata, V. L.; Boskovic, C. Switching metal complexes via intramolecular electron transfer: connections with solvatochromism. *Inorg. Chem. Front.* **2021**, *8*, 1840–1864.

[18] Nadurata, V. L.; Hay, M. A.; Janetzki, J. T.; Gransbury, G. K.; Boskovic, C. Rich redox-activity and solvatochromism in a family of heteroleptic cobalt complexes. *Dalton Trans.* **2021**, *50*, 16631–16646.

[19] Burgess, J.; Fawcett, J.; Haines, R. I.; Singh, K.; Russell, D. R. Structure, solvatochromism, and solvation of trans-[CoIII(cyclam)(NCS)2](NCS) and the structure of [CoII(Me4cyclam) (NCS)]2[Co(NCS)4]MeOH. *Trans. Met. Chem.* **1999**, *24*, 355–361.

[20] Karthikeyan, G.; Anbalagan, K.; Elango, K. P. Solvent and chelation effects on the photoreduction of cobalt(III)–amine complexes in aqueous–organic solvent media. *Trans. Met. Chem.* **2002**, *27*, 52–57.

[21] Otsuka, F. S.; García Otaduy, M. C.; Nascimento, O. R.; Garrido Salmon, C. E.; Huber, M. Challenges of Continuous Wave EPR of Broad Signals—The Ferritin Case. *Appl. Magn. Reson.* **2024**, *55*, 1605–1620.

[22] Eaton, G. R.; Eaton, S. S.; Barr, D. P.; Weber R. T. in *Quantitative EPR: A Practitioners Guide* (Eds.: G.R. Eaton, S.S. Eaton, D.P. Barr, R.T. Weber), Springer Vienna, Vienna, **2010**, pp. 25–36.

[23] Wettstein, L.; Specht, J.; Kesselring, V.; Sieben, L.; Pan, Y.; Käch, D.; Baster, D.; Krumeich, F.; El Kazzi, M; Bezdek, M. J. A Dye-Sensitized Sensor for Oxygen Detection under Visible Light. *Adv. Sci.* **2024**, *11*, 2405694.

[24] Luo, S.-X. L.; Swager, T. M. Chemiresistive sensing with functionalized carbon nanotubes. *Nat. Rev. Methods Primers* **2023**, *3*, 73.

[25] Neese, F. The ORCA program system. *WIREs Comp. Mol. Sci.* **2012**, *2*, 73–78.

[26] Neese, F. Software update: The ORCA program system—Version 5.0. *WIREs Comp. Mol. Sci*. **2022**, *12*, e1606.

[27] Becke, A. D. Density-functional thermochemistry. III. The role of exact exchange. *J. Chem. Phys.* **1993**, *98*, 5648–5652.

[28] Lee, C.; Yang, W.; Parr, R. G. Development of the Colle-Salvetti correlation-energy formula into a functional of the electron density. *Phys. Rev. B* **1988**, *37*, 785–789.

[29] Stephens, P. J.; Devlin, F. J.; Chabalowski, C. F.; Frisch, M. J. Ab Initio Calculation of Vibrational Absorption and Circular Dichroism Spectra Using Density Functional Force Fields. *J. Phys. Chem.* **1994**, *98*, 11623–11627.

[30] Grimme, S.; Ehrlich, S.; Goerigk, L. Effect of the damping function in dispersion corrected density functional theory. *J. Comput. Chem.* **2011**, *32*, 1456–1465.

[31] Weigend, F.; Ahlrichs, R. Balanced basis sets of split valence, triple zeta valence and quadruple zeta valence quality for H to Rn: Design and assessment of accuracy. *Phys. Chem. Chem. Phys.* **2005**, *7*, 3297–3305.

[32] Barone, V.; Cossi, M. Quantum Calculation of Molecular Energies and Energy Gradients in Solution by a Conductor Solvent Model. *J. Phys. Chem. A* **1998**, *102*, 1995–2001.

[33] Marenich, A. V; Cramer, C. J.; Truhlar, D. G. Universal Solvation Model Based on Solute Electron Density and on a Continuum Model of the Solvent Defined by the Bulk Dielectric Constant and Atomic Surface Tensions. *J. Phys. Chem. B* **2009**, *113*, 6378–6396.

[34] Mardirossian, N.; Head-Gordon, M. ωB97X-V: A 10-parameter, range-separated hybrid, generalized gradient approximation density functional with nonlocal correlation, designed by a survival-of-the-fittest strategy. *Phys. Chem. Chem. Phys.* **2014**, *16*, 9904–9924.

[35] Neese, F.; Wennmohs, F.; Hansen, A.; Becker, U. Efficient, approximate and parallel Hartree–Fock and hybrid DFT calculations. A ‘chain-of-spheres’ algorithm for the Hartree–Fock exchange. *Chem. Phys.* **2009**, *356*, 98–109.
